# Quantitative Proteomic Analysis of Meningiomas for the Identification of Surrogate Protein Markers

**DOI:** 10.1038/srep07140

**Published:** 2014-11-21

**Authors:** Samridhi Sharma, Sandipan Ray, Aliasgar Moiyadi, Epari Sridhar, Sanjeeva Srivastava

**Affiliations:** 1 Department of Biosciences and Bioengineering, Indian Institute of Technology Bombay, Powai, Mumbai 400076, India; 2 Department of Neurosurgery, Advanced Center for Treatment Research and Education in Cancer, Tata Memorial Center, Kharghar, Navi Mumbai 410210, India; 3 Department of Pathology, Tata Memorial Hospital, Mumbai 400012, India

## Abstract

Meningiomas are the most common non-glial tumors of the brain and spine. Pathophysiology and definite histological grading of meningiomas are frequently found to be deceptive due to their unusual morphological features and locations. Here for the first time we report a comprehensive serum proteomic analysis of different grades of meningiomas by using multiple quantitative proteomic and immunoassay-based approaches to obtain mechanistic insights about disease pathogenesis and identify grade specific protein signatures. *In silico* functional analysis revealed modulation of different vital physiological pathways including complement and coagulation cascades, metabolism of lipids and lipoproteins, immune signaling, cell growth and apoptosis and integrin signaling in meningiomas. ROC curve analysis demonstrated apolipoprotein E and A-I and hemopexin as efficient predictors for meningiomas. Identified proteins like vimentin, alpha-2-macroglobulin, apolipoprotein B and A-I and antithrombin-III, which exhibited a sequential increase in different malignancy grades of meningiomas, could serve as potential predictive markers.

Meningiomas arising from meninges of the central nervous system account for 30% of all reported brain tumors^1^. Meningiomas exhibit diverse clinical manifestations and histological features; based on which WHO has classified human meningiomas in three different malignancy grades: benign (grade I), atypical (grade II) and anaplastic (grade III)^2^. Even though, the majority of meningiomas are generally benign in nature, higher grade tumors do exhibit a propensity for progression, recurrence and fatality^3^. Additionally, unusual locations, diversity of the histomorphological spectrum and heterogeneity of the grading criteria lead to diagnostic difficulties, especially grading^4^. Moreover, understanding of the recurrence, proliferation and resistance acquisition of these tumors is largely obscure and knowledge available regarding the mechanism for meningioma tumorgenesis is sporadic and markedly lesser studied than gliomas^5^,^6^. Often meningiomas have been found to be diagnosed as an incidental finding for unrelated clinical symptoms, when a clinical dilemma of intervention versus observation is faced.

The significant morphological variations and divergent biological features leads to multiple sub-classes within a specific type or even grade of a tumor^7^,^8^. Comprehensive proteomic characterizations of different sub-types/malignancy grades could explore the proteome level diversity within specific types of cancers similar to that achieved through genomic and other molecular level investigations^9^,^10^. Consequently, in quest of alternative diagnostic modalities for human meningiomas apart from clinical symptoms and histopathological investigation, serum protein biomarkers could be considered as promising candidates^11^. Proteomic analysis of biological specimens has been found to be informative for investigation of disease pathobiology and identification of potential surrogate markers in different types of brain tumors^12^,^13^,^14^,^15^,^16^. In contrast, few proteome level studies have been conducted to investigate pathophysiology of different grades of human meningiomas^5^. There are reported studies demonstrating comparative tissue proteomics^17^,^18^, serum autoantibody profiles^19^, and proteomic alterations in cerebrospinal fluid^20^ of meningioma subtypes. However, no comprehensive quantitative serum proteomics analysis reported hitherto to describe alterations of human serum proteins and related biological pathways in different grades of human meningiomas.

This study aims to investigate alterations in the human serum proteome in different grades of human meningiomas; grade I (benign), grade II (atypical) and grade III (anaplastic) to obtain insights about disease pathogenesis and identify grade specific surrogate protein markers. For comparative proteomic analysis three complementary quantitative proteomic approaches; 2D-differential in gel electrophoresis (2D-DIGE) followed by matrix-assisted laser desorption/ionization tandem time-of-flight mass spectrometry (MALDI-TOF/TOF MS) and isobaric tags for relative and absolute quantitation (iTRAQ)-based and label-free quantitative proteomics in combination with electrospray ionization quadrupole time-of-flight (ESI-Q-TOF) LC/MS-MS were employed. The results obtained from these complementary proteomic techniques were validated by ELISA and western blotting (Figure 1). The differentially expressed serum proteins (*p* < 0.05) identified in different grades of meningiomas were subjected to functional pathway analysis. Different software like protein analysis through evolutionary relationships (PANTHER), database for annotation, visualization and integrated discovery (DAVID), and GeneTrail functional annotation tools were employed to understand their biological context, involvement in various physiological pathways and association with disease pathophysiology. Functional pathway analysis revealed the modulation of different vital physiological pathways, including complement and coagulation cascades, metabolism of lipids and lipoproteins, signaling in immune system, cell growth and apoptosis, and integrin signaling in meningiomas. To the best of our knowledge, this is the first comprehensive investigation describing serum proteomic alterations in different grades of human meningiomas. Our findings may open up new opportunities for the early detection and prognosis of human meningiomas and provide better understanding of the underlying mechanism of the disease pathogenesis and tumour progression.

## Results

### Study population profiles

In order to minimize the pre-analytical variations and specify the disease-related alterations in meningioma patients (MGI, MGII and MGIII) compared to the healthy controls, uniform sample collection, handling and storage process was followed. Blood samples were collected from the patients diagnosed with different grades of meningioma. Fourteen grade I meningioma patients with an average age 43.28 (range 17–57), 5 grade II meningioma patients with an average age 53.8 (range 43–67), and 1 grade III meningioma patient of age 61 were enrolled for this study. For the comparative proteomic study, a healthy population of comparable age range; average age 41.4 (range 20–62) was selected (Table S1).

### Identification of differentially expressed serum proteins in meningiomas by 2D-DIGE analysis

**Figure 2:**
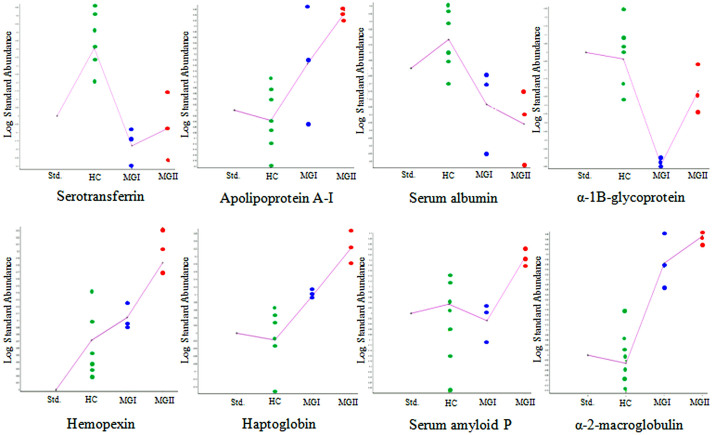
Differentially expressed serum proteins in different grades of meningiomas identified using 2D-DIGE. Trend of few selected differentially expressed serum proteins in different grades of meningiomas represented as standardized log abundance of spot intensity.

In DeCyder 2D software analysis approximately 1500 protein spots were detected on each 2D-DIGE gels. Comparative serum proteome analysis of the healthy controls and different grades of meningioma patients revealed differential expression of 41 and 24 protein spots (*p* < 0.05) in MGI and MGII, respectively (Table S2 and Table S3). In grade I meningioma, among the 41 differentially expressed protein spots, 20 spots exhibited reduced expression, while the remaining 21 spots were up-regulated (Table S2; Figure S1A). In grade II meningioma, expression level of 14 protein spots was elevated, whereas 10 spots were found to be down-regulated (Table S3; Figure S1B). Identity of 27 and 10 protein spots in MGI and MGII, respectively, was established by the subsequent MALDI-TOF/TOF MS analysis (Table S4 and Table S5). Few spots remained unidentified due to the extremely low intensity of those spots and inadequate quantity of detectable peptides. Alpha-1-antitrypsin, Ig kappa chain, serum albumin, hemopexin and haptoglobin were identified as multiple spots in 2D-DIGE gels most likely due to the presence of multiple isoforms (Table S4 and Table S5). DIGE gel images, 3D views and graphical representation of selected protein spots in MGI and MGII are shown in Figure S1. Among the identified differentially expressed proteins, 6 candidates; hemopexin, serum albumin, haptoglobin, alpha-2-macroglobulin, apolipoprotein A-I and serotransferrin were found to be commonly altered in grade I and grade II meningiomas, however levels of differential expression (fold-changes) was found to be different (Figure 2).

### Alterations in serum proteome in different grades of meningiomas revealed by iTRAQ-based and label-free quantitative proteomics analysis

**Figure 3:**
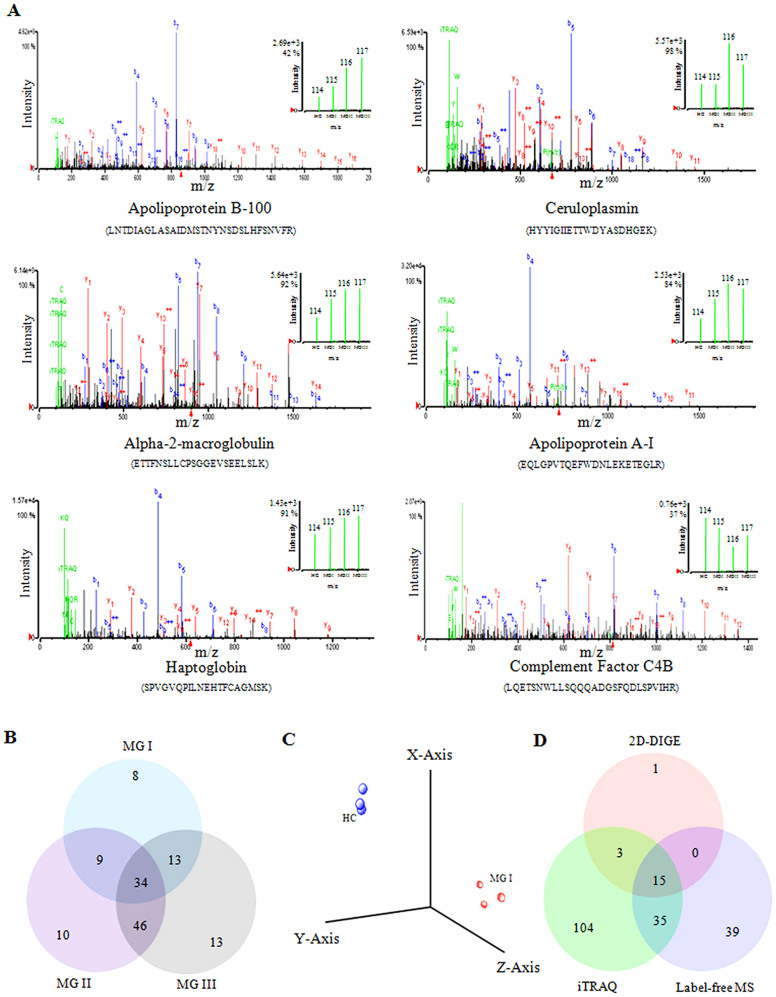
Comparative analysis of different grades of meningiomas by iTRAQ and label-free quantitative proteomics. (A) Representative MS/MS spectrum of a few selected differentially expressed proteins. Inset showing the iTRAQ reporter ion intensities for representative peptides in control and different grades of meningiomas. (B) Venn diagram showing the unique and common differentially expressed proteins in different grades of meningiomas identified in iTRAQ analysis. (C) Discrimination of meningioma grade I patients from healthy controls on the basis of protein expression profile obtained in label-free MS analysis. (D) Venn diagram showing the unique and common differentially expressed proteins in meningiomas identified by 2D-DIGE, iTRAQ and label-free MS.

In this study, two LC-based quantitative proteomics approaches; iTRAQ and label-free were applied. Tryptic digested fractions of healthy control, meningioma grade I (MGI), grade II (MGII) and grade III (MGIII) samples were labelled with 114, 115, 116 and 117 isobaric reagents, respectively for differential quantitative proteomic analysis. A total of 157 significant proteins were identified based on the relative intensity with and 1% FDR. Quantitative proteomic analysis based on the iTRAQ ratios indicated differential expression (fold-change ≥ 1.5) of 64 proteins in MGI (31 up-regulated and 33 down-regulated), 99 proteins in MGII (72 up-regulated and 27 down-regulated) and 106 proteins in MGIII (78 up-regulated and 28 down-regulated) (Figure S2). MS/MS spectra of a few selected proteins with inset depicting the iTRAQ reporter ion intensities for representative peptides in healthy controls and different grades of meningiomas (MGI, MGII and MGIII) are provided in the Figure 3A. Among the differentially expressed proteins (fold-change ≥ 1.5; FDR 1%), 9 proteins were common between MGI and MGII, 13 proteins were common between MGI and MGIII, and 46 proteins were common between MGII and MGIII, while 34 proteins were found to be overlapping among all the three grades of meningiomas (Figure 3B). The iTRAQ ratios of entire protein list along with sequence coverage, protein score and unique peptide information are provided in supplementary information (Table S6).

Comparative analysis of serum proteome profiles of healthy subjects and grade I meningioma patients was also performed by using label-free MS/MS analysis. A total of 363 proteins were identified with high confidence in the label-free MS/MS analysis. Among the identified proteins 61 proteins were found to be with two or more unique peptides (Table S7). Identification of the differentially expressed proteins in meningioma patients was performed by measuring the mean peak intensity of all peptides identified for any protein common to both control and diseased samples. In MPP analysis over 95% accuracy was obtained for discrimination of the meningioma grade I patients from the healthy controls on the basis of protein expression profiles obtained in label-free MS analysis (Figure 3C).

Further comparative analysis of the findings obtained from the three complementary proteomic approaches; 2D-DIGE, iTRAQ and label-free MS analysis indicated that almost all of the differentially expressed proteins, except plasma retinol binding protein, visualized in 2D-DIGE were also identified in the LC-based quantitative proteomics approaches (Table 1).Among the identified differentially expressed candidate proteins, 50 were found to be common between iTRAQ and label-free analysis (Figure 3D). The overlap between the differentially expressed proteins identified in iTRAQ and label-free LC-based quantitative proteomics was not very significant; overlap was 57% and 32% of the total proteins identified by label-free and iTRAQ, respectively. Although for majority of the proteins, which were identified by more than one approach, the trends of differential expressions (up-/down-regulation) were found to be similar, while the magnitude of alterations in expression level observed in different analytical platforms were not exactly same.

### Modulation of different biological process and physiological pathways in meningiomas

**Figure 4:**
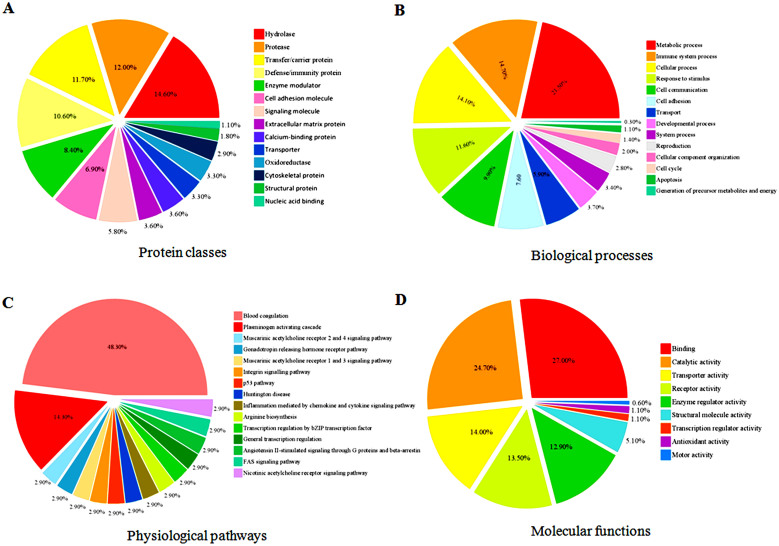
Functional clustering and physiological pathways associated with the differentially expressed proteins identified in meningiomas. Pie charts showing the protein classes (A), protein classes (B), biological process (C), and physiological pathways (D) molecular functions.

The differentially expressed serum proteins identified in meningioma grade I, II and III samples using DIGE, iTRAQ and label-free MS data were subjected to pathway analysis using DAVID, PANTHER and GeneTrail functional annotation tools for demonstrating their biological context, association with diverse physiological pathways and connection with disease pathophysiology (Table 2). Protein class analysis using PANTHER indicated differentially expressed candidates in meningiomas are mostly hydrolases, transfer proteins, proteases, immunity proteins, enzyme modulators and signaling molecules (Figure 4A). Cellular component analysis indicated the major portion of the identified differentially expressed proteins belongs to the extracellular region, while some were intracellular, and a few reside as protein complex (Table S8A). PANTHER analysis revealed the association of proteins in different biological processes, including response to immune system process, cellular processes, metabolic processes, developmental processes, cell communication, cell adhesion, apoptosis and cell cycle (Figure 4B; Table S8A).

Functional pathway analysis by DAVID revealed association of the differentially expressed proteins in complement and coagulation cascades, ECM-receptor interaction and PPAR signaling pathway under the KEGG (Kyoto Encyclopedia of Genes and Genomes) category; while hemostasis, signaling in immune system and metabolism of lipids and lipoproteins were identified as the major pathways under the REACTOME category (Figure S3; Table S8A). Extrinsic prothrombin activation pathway and complement cascades were the major candidates from BIOCARTA (Table S8A). Details of functional annotation clustering are provided under the supplementary information (Table S8B). In PANTHER analysis, 15 different pathways were identified, among which blood coagulation, plasminogen activating cascade, angiotensin II-stimulated signaling through G proteins and beta-arrestin, FAS signaling, p53 pathway and integrin signaling pathway were found to be statistically significant (*p* < 0.05) (Figure 4C; Table S8A). According to the molecular function analysis, most of the differentially expressed proteins were related to catalytic and binding activities. Few candidates were found to be involved in transport and enzyme regulated activities as well (Figure 4D). The list of differentially expressed proteins was also analyzed using GeneTrail software, which provided some overlapping information regarding the pathways obtained from KEGG category. Cellular and molecular sub-trees associated with the differentially expressed proteins identified in meningiomas generated by GeneTrail analysis are depicted in the Figure S4.

### Immunoassay-based validations of selected differentially expressed proteins and evaluation of the accuracy of the proteins in prediction of different grades of meningiomas

**Figure 5:**
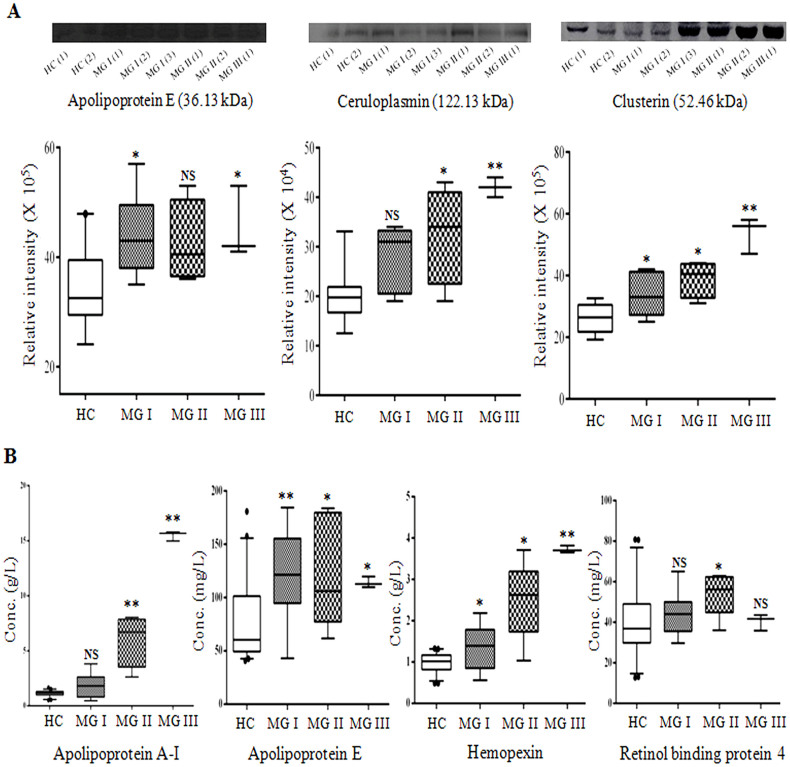
Immunoassay-based validation of selected differentially expressed proteins. (A) Western blot analysis of Apo E, CP, and CLU in serum samples of different grades of meningioma patients and healthy controls. (B) Estimation of serum levels of Apo A-I, Apo E, HPX, and RBP4 in HC (n = 45) and grade I (n = 14), grade II (n = 5) and grade III (n = 1) ^#^ meningioma patients by ELISA assay (# three technical replicates were analyzed). NS indicates *p* > 0.05; * indicates *p* < 0.05, ** indicates *p* < 0.005 in a Mann Whitney U-test.

Differential expressions of a few selected serum proteins identified in quantitative proteomics analysis were validated further using two immunoassay-based methods; western blotting and ELISA. Selection of the proteins for validation was performed on the basis of fold-changes (from 2D-DIGE and iTRAQ-based mass spectrometric experiments), possible functional association of the proteins with meningioma pathobiology, and availability of the required antibodies/ELISA kits. Western blot analyses of three differentially expressed targets proteins; apolipoprotein E, ceruloplasmin and clusterin were carried out using a subset of healthy control and meningiomas samples (MGI, MGII and MGIII). Equal loading of the samples in each lane was confirmed by CBB staining of the SDS-PAGE gels and Ponceau staining of the transferred blots containing the resolved proteins (Figure S5). Western blot results indicated up-regulation of Apo E, CLU and CP in meningioma patients compared to the healthy controls (*p* < 0.005 in a Mann Whitney U-test) (Figure 5A). However, differential expression of Apo E in MGII patients and CP in MGI patients was found to be statistically insignificant.

Hemopexin (HPX), apolipoprotein E and A-I (Apo E and A-I), and plasma retinol binding protein (RBP4) concentrations were directly measured in the serum samples of healthy controls and meningiomas patients (MGI, MGII and MGIII) using ELISA. Compared to the healthy controls, all the three grades of meningioma patients found to have higher serum level of Apo E and HPX (*p* < 0.01 in a Mann-Whitney test). (Table S9) (Table S9). The serum levels of Apo A-I was also found to be significantly (*p* < 0.001 in a Mann-Whitney test) increased in the Grade II and III meningioma patients compared to the HC population (Figure 5B and Figure S6). On the other hand, differential expression of RBP4 was found to be significant only in the grade II meningioma patients (Figure 5B). Results acquired from the immunoassay-based analyses confirmed the findings of the discovery-phase proteomic analysis.

Receiver operating characteristic (ROC) curve analysis was carried out to evaluate the individual performance of 4 proteins; HPX, Apo E and A-I, RBP4 for prediction of different grades of meningiomas. The area under the ROC curve (AUC) signifies the accuracy of the differentially expressed proteins to distinguish meningiomas from HC. ROC curves demonstrate Apo E (AUC = 0.794) as an effective predictor proteins for MGI (Figure S7A), while Apo A-I (AUC = 0.937) and HPX (AUC = 0.906) exhibited better efficiency for discrimination of MGII patients from the healthy population (Figure S7B). A cut off value > 93.93 mg/L for Apo E, demonstrated 79% sensitivity and 71% specificity for MGI. At a threshold value > 1.44 g/L for Apo A-I, 95% sensitivity and 91% specificity were obtained for the MGII patients; while HPX at a cut off value > 1.295 g/L exhibited 85% sensitivity and 93% specificity in predicting MGII (Table S10).

## Discussion

Meningiomas are the most common non-glial tumors of the brain and spine^21^. Their pathophysiology is still unclear and often diagnosis can be misleading especially when radiology is equivocal and locations of occurrence are unusual^4^. There are certain challenges for comprehensive molecular characterization and comparison between different malignancy grades of meningiomas. The scarcity of grade II and III meningiomas (<10% of all of meningiomas, but are more invasive and often fatal in nature with an increased risk of recurrence and poor prognosis) compared to the grade I benign meningiomas hampers comparative studies^3^. Another limitation in conducting disease *vs* control studies is age related differences among the clinical cohorts which may be responsible for the variation in human plasma proteome profiles to some extent, as ageing causes physiological dysfunction^22^. Also, it is often challenging to obtain suitable diseased and control samples of exactly comparable socio-epidemiological background within a very narrow range. Another factor is lack of homogeneity in the WHO grade I meningioma group itself, which is histologically divided into different subtypes with variable biological behaviour^2^. In addition, intratumoral heterogeneity, which might be spatial (due to the genetic aberrations in the different geographical regions of the same tumor) or temporal (between the primary tumor and the local recurrence), has not been studied adequately in meningiomas. Investigation of global and differential proteome expression profiles of cancer patients has been found to be proficient for tumor classification and identification of potential diagnostic and predictive markers^23^. Moreover comprehensive proteome analysis of protein–protein interactions involved in signaling networks are extremely informative for investigating the molecules controlling the vital processes involved in cancer^24^.

Previously reported studies have used CSF and tissue samples for comprehensive proteome analysis in different grades of meningioma^7^,^19^,^20^. However this is the first in-depth analysis unraveling the serum proteomic alterations amongst different grades of meningiomas using three complementary quantitative proteomic approaches; 2D-DIGE, iTRAQ and label-free MS/MS. Multiple complementary proteomics techniques are crucial to obtain the maximum coverage and identification of the differentially expressed candidates with high confidence. With multiple proteomic approaches we were able to identify higher number of differentially expressed serum proteins in comparison to the earlier studies performed using tissue and CSF samples^17^,^20^. For the first time we have revealed modulation of complement and coagulation cascades, metabolism of lipids and lipoproteins and integrin signaling in meningiomas through proteomic investigation.

Our quantitative proteomic analysis revealed differential expression of multiple proteins involved in coagulation cascades indicating the apparent coagulation disorders in the meningioma patients (Table 2). Meningiomas are associated with the risk of perioperative thromboembolic events^25^ and coagulopathy during neurosurgery^26^. Similarly frequent association of blood coagulation activation, thrombosis and hemorrhage have been reported in different cancers^27^,^28^. The activation of blood coagulation leads to peritumoral fibrin deposition and altered hemostatic factors which is imperative for tumor development and progression^27^,^29^. Alterations in hemostatic system in brain tumor patients might be caused by injured endothelial cells, which are vital for controlling the usual hemostatic conditions^30^. In our study, expression levels of few important candidates of coagulation system and hemostasis including antithrombin-III, alpha-2-antiplasmin, vitamin K-dependent protein S, fibrinogen alpha chain, plasminogen, alpha-2-macroglobulin and coagulation factor XII were found to be elevated in the different grades of meningiomas (Table 1 and S6). Thrombospondin-1 (TSP-1) plays an important role in regulation of tumor growth and angiogenesis, and can inhibit the proliferation and metastasis of different types of solid tumors^31^. Down-regulation of TSP-1 identified in the meningioma patients, might be an outcome of activated oncogenes such as Myc and Ras which promotes tumor growth and metastasis. Anti-thrombins – such as antithrombin-III, argatroban, serpin antithrombin having anti-angiogenic activity were found to be elevated which could reduce tumor mass thereby extending the survival time of the meningioma patients. These coagulation abnormalities are generated at an early stage of tumorigenesis which is crucial for tumor progression. Hence in- depth analysis of the biological process could be informative for identification of risk factors involved in tumor malignancy and may act as potential prophylactic and therapeutic targets.

Complement cascades stimulate cellular proliferation and regeneration, and therefore affect tumor growth. Comparative serum proteomic analysis of healthy subjects and meningioma patients indicated activation of complement cascades in meningiomas with up-regulation of quite a few complement factors including C5, C8 beta chain, C6, C4-B (Table 2). The exact mechanism through which complement proteins influence cancer growth is obscure; however multiple possible mechanisms have been postulated including dysregulation of mitogenic signaling pathways, constant cellular proliferation, angiogenesis, resistance to apoptosis, and escape from the immune-system^32^. Whereas, some studies indicate that malignant tumor cells escape complement attack by the expression of membrane-bound complement regulatory proteins (mCRPs)^33^. Another interesting finding is the modulation of integrin signaling pathway and integrin cell surface interactions in meningioma (Table 2). Integrins play a central role in cell migration and invasion, thus affect tumour growth and metastasis and regulate tumor cell survival and malignancy^34^. Additionally, integrin-associated signaling makes cells more resistant to genotoxic anti-cancer agents like ionizing radiation and chemotherapeutic materials^35^ and matrix cross linking facilitates tumor progression by inducing integrin signaling^36^. Consequently, integrin signaling has been extensively investigated as therapeutic targets, and integrins can be promising biomarkers for evaluating the effectiveness of anti-angiogenic and anti-tumour agents^34^,^37^.

Apart from the members of complement and coagulation cascades and integrin signaling pathway, expression levels of quite a few serum proteins including apolipoprotein A-I, A-II, A-IV, B-100, C-II and E, and serum albumin associated with metabolism of lipids and lipoproteins were found to be altered in the meningioma patients; particularly in atypical and anaplastic meningiomas (Table 2). Interestingly, earlier studies have introduced lipid metabolism as a promising target for brain cancer therapy^38^; and demonstrated that covalent connection of apolipoprotein A-I and B-100 to albumin nanoparticles can facilitate drug transport into the brain^39^. Elevated expression level of Apoliprotein A-I in meningioma tumor tissue samples was reported previously by Okamoto et al.^17^, which supports our findings. However, isoforms of Apo A-I and A-II have been reported also as potential markers for other cancers such as ovarian^40^ and prostate cancer^41^.

Discrimination between the generic and specific responses in various human cancers is very crucial, since comparative analyses of different types of tumors often indicate modulation of similar serum proteins and physiological pathways associated with the complex processes of tumorigenesis^42^. In this context, differentially expressed proteins identified in meningiomas in this study, were compared further with the list of differentially expressed serum proteins reported earlier in another class of clinically relevant adult brain tumors; glioblastomas^13^,^14^. Expectedly, quite a few of the identified proteins were found to be commonly modulated in both meningiomas and gliomas; however, the magnitude of alteration in expression levels was different in these two types of brain tumors. For instance apolipoprotein A-I & A-II, alpha-1-acid glycoprotein 2, hemoglobin subunit beta/alpha, leucine-rich alpha-2-glycoprotein and vimentin exhibited very high level of differential expression in the meningioma patients (compared to the healthy subjects) (Table S6), but alteration in the expression levels of those candidates were subtle in gliomas^13^. Interestingly, expression levels of many serum proteins including thrombispondin-I, serotransferrin, and alpha-2-macroglobulin were found to be altered in meningioma patients, but not reported in glioblastomas (GBMs). Some of our identified differentially expressed proteins like apolipoprotein E, carbonic anhydrase 1, leucine-rich α-2-glycoprotein, afamin etc. which showed detectable alteration in expression levels in the benign meningima (grade I) may act as potential candidate markers for meningiomas at their early stages of development. Proteins such as vimentin, α-2-macroglobulin, apolipoprotein B and A-I, antithrombin-III etc., which exhibited sequential changes in expression level in different malignancy grades, therefore significant alterations in expression level between the benign and atypical or anaplastic meningiomas, can be considered as potential disease monitoring markers (Table S6). Protein exhibiting differential expression in a specific grade of meningiomas could serve as promising grade specific signatures.

In comparative proteomic study of disease *vs*. healthy controls, sometimes differentially expressed candidates are a result of the generic acute phase reactions which should not be misinterpreted as promising markers. Hence it has been comprehended that identification of a panel of serum biomarkers is much more effective for detection of specific cancer and its discrimination from the other types of cancers. To this end, development of for multiplexed multiple reaction monitoring (MRM) assays for simultaneous absolute quantitation of different interesting target proteins in serum/plasma will be useful. There are few proteomic studies which have shown aberration in the expression of the different tissue and cerebrospinal fluid proteins in meningioma^17^,^20^. Using classical 2D gel electrophoresis Okamoto et al. reported differential expression of 24 proteins, including apolipoprotein E, serum albumin, apolipoprotein A-I and alpha-1 antitrypsin, which are also identified in our data^17^. Wiemels et al. screened meningioma patients and healthy subjects for autoantibody reactivity against enolase 1 (ENO1), NK-tumor recognition protein (NKTR), and nuclear mitotic apparatus protein 1 (NUMA1)^19^(Table S11; Figure S8).

In the present study, quantitative proteomics analysis of different grades of meningiomas (benign, atypical, and anaplastic) and healthy controls was performed using three complementary quantitative proteomic approaches; 2D-DIGE, iTRAQ-based and label-free LC/MS-MS. Our investigation identified alterations in the expression levels of multiple serum proteins associated with diverse physiological pathways, including complement and coagulation cascades, metabolism of lipids and lipoproteins, signaling in immune system, FAS and integrin signaling and plasminogen activator cascade in meningiomas. Some of the modulated pathways including integrin signaling, hemostasis and coagulation cascade and apoptosis are promising targets for new cancer drug development. Further, in-depth investigation of the functional involvement of these differentially expressed proteins in tumor development and progression will enhance our understanding regarding meningioma pathobiology and identification of novel therapeutic targets. Meningiomas are routinely diganosed using magnetic resonance imaging (MRI) and computed tomography (CT), which are adequately robust and efficient for detection of the meningiomas but the tumors with atypical locations and misleading morphological features often leads to clinical dilemmas. To this end, measurement of the identified serum protein biomarkers could be considered as a promising approach, complementary to the analysis of clinical symptoms and radiological parameters and can be used for diagnosis and prognosis of meningiomas. Additionally, the identified classifier proteins may aid in comprehensive characterizations of different sub-types of meningiomas, existing even within a specific malignancy grade, and prediction of transition from their benign to malignant manifestations, which are not easily achievable by the conventional diagnostic approaches. However, prior to any clinical implication, further validation of the identified proteins in bigger cohorts of meningioma patients and healthy controls is obligatory. Further valuable information regarding the correlation of the identified markers with disease progression on large patient cohorts and their utility as disease monitoring or prognostic markers can be obtained from the longitudinal investigations involving repeated analysis of the patients after therapeutic interventions or after surgical removal, which could be an exciting future continuation of the present study. Additionally, comprehensive analysis of changes in proteome profiles in other biological specimens like CSF or tumor tissue lysates can provide a comprehensive glimpse of the overall proteomic alterations and pathobiology of meningiomas.

## Methods

### Ethics statement

This study has been approved by the Institutional Ethics Committee of Advanced Centre for Treatment Research and Education in Cancer (ACTREC) and Tata Memorial Hospital (TMH), Mumbai, India and all experiments were performed in accordance with relevant guidelines and regulations. Pre-informed written consents of all the subjects enrolled for this study were taken before sample collection process.

### Subject selection and sample collection

Twenty subjects with radiologically suspected meningioma undergoing surgery were enrolled for this study from Advanced Centre for Treatment Research and Education in Cancer (ACTREC) and Tata Memorial Hospital (TMH), Mumbai, India. Out of these 20 meningioma patients; 14 were histologically diagnosed as grade I, five as grade II, and one as grade III according to the criteria of 2007 WHO classification for primary Central Nervous System tumours. For the comparative analysis, blood samples from 45 age-matched healthy subjects were collected from the antecubital vein of the subjects using serum separation tubes (BD Vacutainer®; BD Biosciences). Sample collection, processing and storage were performed as previously described^43^.

### Sample processing, 2D-DIGE and software analysis

Protein extraction and 2D-DIGE were performed as described previously^43^. Protein extraction from depleted serum samples was performed using TCA/acetone precipitation method^43^. The two most high abundance serum proteins (albumin and IgG) were depleted using Albumin & IgG Depletion SpinTrap (GE Healthcare) following the manufacturer's instructions. Samples (meningiomas grade I/II and control) were labeled with Cy3 and Cy5, while a mixture of equal amounts of all samples to be analyzed in the experiment, regarded as internal standard, was labeled with the third fluorescent dye; Cy2 according to the manufacturer's instructions (GE Healthcare). DIGE experiments were performed in three technical replicates and dye-swapping was performed while labeling the meningioma and control samples to avoid any type of labeling effects. Image acquisition and data analysis was performed as described previously^42^. In brief, comparative analysis was performed using two different modules, differential in-gel analysis (DIA) and biological variation analysis (BVA) of DeCyder 2D software; *version* 7.0 (GE Healthcare). Statistical significance of the average ratio of expressions was analyzed by Student's t-test and ANOVA (*p* < 0.05).

### In-gel digestion, MALDI-TOF/TOF analysis and protein identification

In-gel digestion of the statistically significant (*p* < 0.05) differentially expressed protein spots identified in 2D-DIGE experiment and subsequent enrichment of digested peptides using Zip-Tip C18 pipette tips (Millipore, USA) were performed as described previously^43^. An Autoflex MALDI–TOF/TOF mass spectrometer (Autoflex; Bruker Daltonics, MA, USA) equipped with a pulsed N_2_ laser (337 nm) was used for MS and MS/MS analysis. Mass spectra were detected in a reflectron positive mode with the following settings: ion source 1- 19.02 kV; ion source 2-16.65 kV; reflector 1- 21.14 kV; reflector 2- 9.57 kV; lens- 8.00 kV; pulsed ion extraction- 120 ns and nitrogen pressure- 2.5 × 10^5^ Pa. Protein identification was carried out by MS/MS ion search using MASCOT *version* 2.1 against the Swiss-Prot database specifying the following settings; taxonomy: human, trypsin digestion with one missed cleavage, fixed modifications: carbamidomethylation of cysteine residues, variable modifications: oxidation of methionine residues, mass tolerance 100 ppm for MS and 0.4 Da for MS/MS. Identified proteins having at least two unique matched peptides were selected for further analysis. Only those proteins with a protein identification confidence interval of ≥ 95% are reported.

### In-solution digestion, iTRAQ labeling and OFFGEL fractionation

Protein samples (control and different grades of meningiomas) in rehydration solution were exchanged to TEAB buffer using Amicon Ultra 0.5 mL centrifugal 3 kDa filters (Millipore, Watford, UK). Similar sample pooling strategy for control and different grades of meningiomas, which was used for 2D-DIGE analysis, was followed. After buffer exchange, quantification of protein content in each sample was performed using QuickStart Bradford reagent (BioRad, USA). Prior to the iTRAQ labeling, in-solution digestion was performed (75 μg proteins from each sample) following the manufacturer's instructions. Trypsin (Trypsin Gold, mass spectrometry grade, Promega, Madison, WI, USA) was used at a 1:20 trypsin: protein ratio. After in-solution digestion, iTRAQ labeling of the peptides was performed following the manufacturer's instructions (AB Sciex UK Limited, UK). Following labeling strategy was implemented for differential proteomic analysis; control-114, MGI-115, MGII-116 and MGIII-117. All the labeled samples were pooled and concentrated using a speed vac. OFFGEL fractionation of the labeled peptides was performed using a 3100 OFFGEL fractionator (Agilent Technologies, Santa Clara, CA) with high resolution (pH 4–7, 24 cm) IPG strips.

### LC-MS/MS analysis for the protein identification and quantitation

Quantitative proteome analysis of the off-gel fractionated samples was done using 1260 Infinity HPLC-nano-chip linked to Agilent 6550 iFunnel Q-TOF instrument (Agilent technology, USA) equipped with a Polaris C18A chip (150 mm × 0.075 mm) with 160 nL trap column. For elution of the peptides from the analytical column, a linear gradient of 7–35% ACN was used for a run of 60 min at flow rate of 2 μL/min for the capillary pump and 0.5 μL/min for the nano pump. Chip-Cube controlled by the Mass hunter Acquisition software was set to perform data acquisition in a positive ion mode. MS was scanned from 300-2000 and MS/MS from 50–3000. The instrument was operated in a data-dependent manner using AutoMS/MS, selecting max 4 precursors with intensity over 1000 for each cycle. MS/MS was done with a gas pressure of 2 × 10^−2^ bar in the collision cell.

Data files were processed by Spectrum Mill Protein Identification software (Agilent Technologies, USA). The Paragon algorithm was used as the default method for search with trypsin as a digesting agent with up to two allowed miss cleavages, and All searches were performed and protein identification was executed against the UniProt database (Swiss-Prot and NCBI databases). Data was extracted between MH+ 600 and 4000. 20 ppm precursor mass tolerance and 50 ppm fragment mass error tolerance was specified. Only peptides identified with confidence interval (C.I.) values above 95% was used for protein identification and quantification. The iTRAQ report peak areas (RPAs) corresponding to quantification ions m/z 114-117 were extracted from the raw spectra and corrected for isotopic carryover using GPS Explorer. A decoy database search was used for calculation of the false discovery rate (FDR) and a cut-off of 1% was used to report identifications.

### Label-free relative quantitation of proteins

Label-free relative quantitation of in-solution digested serum protein extracts from healthy subjects and meningioma patients (MGI) was performed using Agilent 6550 iFunnel Q-TOF LC MS/MS instrument (Agilent Technologies, USA). 3 μg of each trypsin digested sample was diluted in 10 μL of 0.2% FA/5%ACN and 5 μL was injected in triplicate. Molecular Feature Extraction (MFE) algorithm automatically located all sample components down to the lowest level of abundance and extracted all relevant spectral and chromatographic information. 120 min linear gradient ranging from 5% to 45% B was used for the separation of the peptides for label-free analysis. In the acquisition mode the following parameters were used; MS min range (m/z): 100, MS max range (m/z): 3200, and MS and MS/MS scan rate (spectra/sec): 5. While for the precursor selection following parameters were specified; max precursor selection precursors per cycle: 15, threshold (Abs): 1000, threshold (Rel) (%): 0.010, and target (counts/spectrum): 25000. The instrument was operated in a positive ionization mode. Relative quantitation was performed in three technical replicates. Mass Profiler Professional (MPP) software (Agilent Technologies, USA) was used for visualization and identification of statistically significant differences between the two sample groups.

### Proteins networks and functional analysis

The differentially expressed serum proteins (*p* < 0.05) identified in different grades of meningiomas were analyzed using PANTHER system, *version* 7 (http://www.pantherdb.org)^44^, DAVID database *version* 6.7 (http://david.abcc.ncifcrf.gov/home.jsp)^45^ and GeneTrail (http://genetrail.bioinf.uni-sb.de/)^46^. The list of UniProt Accession from each dataset were uploaded and mapped against reference *Homo sapiens* dataset to extract and summarize functional annotation associated with individual or group of genes and proteins.

### Western blot analysis

Serum proteins extracted from grade I (n = 12), grade II (n = 5) and grade III (n = 1) meningiomas and healthy controls (n = 12) were separated by using 12% SDS-PAGE (50 μg per track) and then transferred onto PVDF membranes under semidry conditions by using an ECL semi-dry transfer unit (GE Healthcare). Equal loading of the samples in each lane was confirmed by CBB staining of the SDS-PAGE gels and Ponceau staining of the transferred blots containing the resolved proteins. Western blot was performed by using monoclonal/polyclonal antibody against apolipoprotein E (Santacruz Biotechnology, sc-6383), ceruloplasmin (Santacruz biotechnology, sc-365206) and clusterin (Santacruz biotechnology, sc-56079) and appropriate secondary antibody conjugated with HRP (GeNei (MERCK)-621140380011730 or 621140680011730. Quantitation of signal intensity of the bands in western blots was performed using ImageQuant software version 5.0 (GE Healthcare).

### ELISA

Quantification of four interesting targets; hemopexin (HPX), apolipoprotein E and A-I (Apo E and A-I), and plasma retinol binding protein (RBP4) was performed by using ELISA. Concentrations of the candidate proteins in serum samples of different grades of meningiomas; grade I (n = 14), grade II (n = 5) and grade III (n = 1) and healthy controls (n = 45) were measured using AssayMax ELISA kits following the manufacturer's instructions (AssayPro, USA). Optical densities were measured at 450 nm and 570 nm using a SpectraMax M2^e^ (Molecular Devices, USA). Statistical significance of differential expression of the serum proteins was analyzed by Mann Whitney U-test.

### Receiver operating characteristic (ROC) curve analysis

Efficiency of the four differentially expressed proteins; HPX, Apo E and A-I, RBP4 for prediction of different grades of meningiomas was analyzed using receiver operating characteristic (ROC) curves [plot of true positives (sensitivity) *vs.* false positives (1- specificity) for each possible cut off] using GraphPad Prism software package (*version* 6) following the protocol as described previously^43^.

## Supplementary Material

Supplementary Information Supplementary information

## Figures and Tables

**Figure 1:**
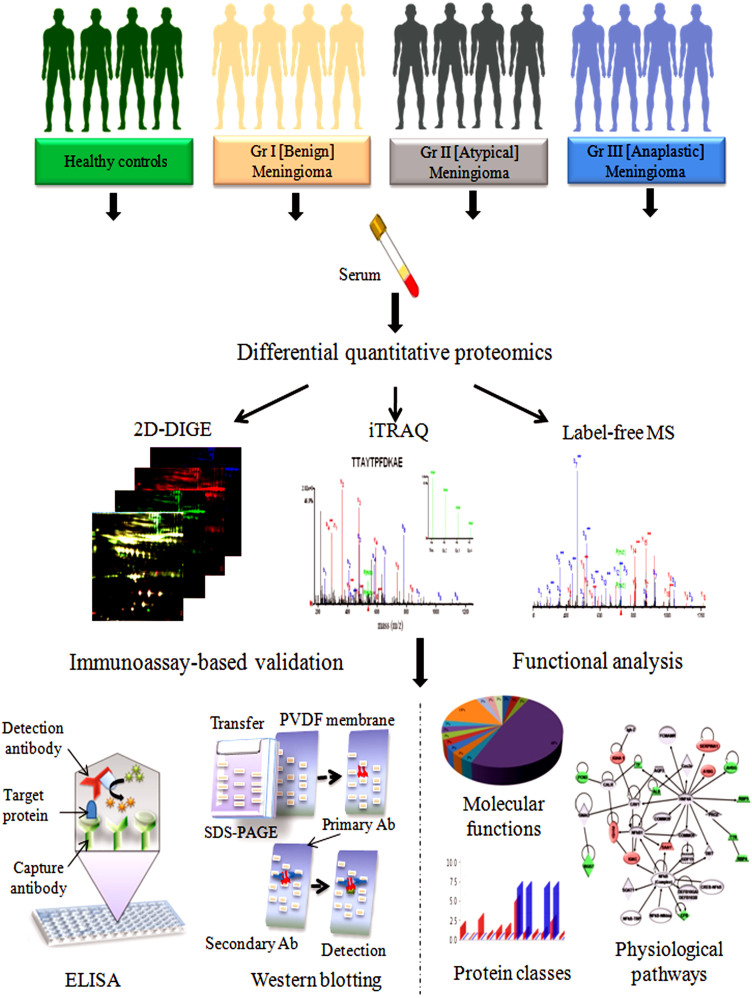
Schematic representation of the experimental strategy for proteomic analysis of alterations in the human serum proteome in different grades of human meningiomas (Drawn by the authors: S.S^1^ & S.R.).

**Table 1:** Partial list of the differentially expressed proteins identified in different grades of meningiomas*

Sl No	Protein Name	Gene name	UniProt Accession number	Num Unique Peps	Score	Sequence coverage (%)	Fold-change (HC *vs.* MGI)	Fold-change (HC *vs.* MGII)	Fold-change (HC *vs.* MGIII)	Molecular Functions #
1	Apolipoprotein B-100^(d)^	APOB	P04114	100	1797.28	32.7	1.79	3.72	6.58	(i),(ii),(iii),(iv)
2	Complement C3^(e)^	C3	P01024	75	1344.36	54.9	1.24	2.50	3.01	(v),(vi)
3	Alpha-2-macroglobulin^(e)^	A2M	P01023	52	954.16	47.8	1.45	3.45	3.12	(vii),(viii),(ix)
4	Apolipoprotein A-I^(e)^	APOA1	P02647	29	539.32	70.4	2.28	7.09	13.26	(i),(iv),(x),(xi),(xiii),(xiv),(xv)
5	Ceruloplasmin^(e)^	CP	P00450	27	564.51	37.8	1.03	2.04	1.43	(xvi), (xvii)
6	Fibronectin^(d)^	FN1	P02751	22	400.36	18.2	0.91	1.42	2.25	(xviii),(ii),(xix)
7	Haptoglobin^(e)^	HP	P00738	22	389.03	48.2	1.67	2.10	1.87	(viii),(xx),(xxi),(xxii)
8	Vitamin D-binding protein^(e)^	GC	P02774	22	382.45	50.6	0.85	1.20	0.78	(xxiii), (xxiv)
9	Antithrombin-III^(d)^	SERPINC1	P01008	22	372.13	51.2	0.93	1.67	2.87	(ii),(viii)
10	Hemopexin^(e)^	HPX	P02790	19	365.85	46.7	1.15	3.09	3.47	(xxv), (xxvi)
11	Plasminogen^(d)^	PLG	P00747	19	336.5	39.8	0.82	1.66	1.86	(viii)
12	Inter-alpha-trypsin inhibitor heavy chain H4^(d)^	ITIH4	Q14624	17	320.84	31.1	1.69	3.12	3.76	(v), (xxvii)
13	Zinc-alpha-2-glycoprotein^(d)^	AZGP1	P25311	13	236.8	44.9	1.39	3.05	3.44	(xii),(xxvii),(xxviii),(xxix)
14	Hemoglobin subunit beta^(d)^	HBB	P68871	10	204.75	78.2	2.53	19.62	8.47	(xxii),(xxx),(xxxi),(xxxii),(xxxiii)
15	Gelsolin^(d)^	GSN	P06396	10	189.08	26.7	0.50	0.48	0.68	(xxxiv)
16	Afamin^(d)^	AFM	P43652	9	169.19	21	1.65	1.83	3.21	(xxxv)
17	Alpha-2-antiplasmin^(d)^	SERPINF2	P08697	9	154.97	31.7	1.44	2.39	3.35	(viii)
18	Transthyretin^(d)^	TTR	P02766	7	152.35	72.7	1.15	1.42	1.86	(xxxvi)
19	Apolipoprotein E^(c)^	APOE	P02649	8	143.68	35.6	2.49	1.48	2.03	(i),(ii),(iv),(x),(xv),(xx),(xxxvii),(xxxviii),(xxxix),(xl)
20	Clusterin^(e)^	CLU	P10909	8	129.38	23.6	1.09	1.18	1.79	(xli),(xlii)
21	Angiotensinogen^(a)^	AGT	P01019	4	89.49	9.8	1.08	3.53	4.40	(viii),(liii),(liv),(lv)
22	Vimentin precursor^(a)^	VIM	P08670.2	5	69.93	13.7	2.07	3.96	2.95	(xliii), (xliv)
23	Vitamin K-dependent protein S^(a)^	PROS1	P07225	3	46.74	5.6	0.73	1.50	1.84	(v),(xlv)
24	Ficolin-3^(a)^	FCN3	O75636	2	40.28	13.7	0.81	0.56	1.73	(xlvi)
25	Serum amyloid P-component^(c)^	APCS	P02743	2	40.02	7.1	1.23	0.18	0.29	(xlvii),(xxv)
26	Leucine-rich alpha-2-glycoprotein^(c)^	LRG1	P02750	1	32.08	7.4	2.98	10.19	4.90	
27	Hyaluronan-binding protein 2^(a)^	HABP2	Q14520	2	28.95	7.1	1.58	1.92	2.54	(viii)(xlviii)
28	Carboxypeptidase N subunit 2^(a)^	CPN2	P22792	2	27.28	4.7	0.65	1.06	1.13	(lii)
29	Glutathione peroxidase 3^(a)^	GPX3	P22352	2	25.48	8.8	1.29	2.42	2.93	(xlix),(l),(li)
30	Plasma retinol binding protein^(b)^	RBP4	P02753	11	637	21.4	1.76	NS	NS	(lvi)

*This is a partial list having few selected candidates identified in DIGE, iTRAQ and label-free MS/MS analysis; complete lists are provided in supplementary information (Table S4–7).

(a) Identified only in iTRAQ, (b) Identified only in DIGE, (c) Identified in DIGE and iTRAQ (d) Identified in label-free MS and iTRAQ, and (e) Identified in DIGE, iTRAQ and label-free MS.

NS, indicates differential expression is not statistically significant.

# Gene ontology terms obtained from UniProt database.

(i) Cholesterol transporter activity; (ii) Heparin binding; (iii) Low density lipoprotein particle receptor binding; (iv) Phospholipid binding; (v) Endopeptidase inhibitor activity; (vi) C5L2 anaphlyatoxin chemotactic receptor binding; (vii) Interleukin-1 binding; (viii) Serine-type endopeptidase inhibitor activity; (ix) Tumor-necrosis factor binding; (x) Beta-amyloid binding; (xi) Cholesterol binding; (xii) Antigen activity; (xiii) High-density lipoprotein particle binding; (xiv) Lipase inhibitor activity; (xv) Phosphatidylcholine-sterol O-acyltransferase activator activity; (xvi) Copper ion binding; (xvii) Ferroxidase activity; (xviii) Collagen binding; (xix) Extracellular matrix structural constituent; (xx) Antioxidant activity; (xxi) Catalytic activity; (xxii) Hemoglobin binding; (xxiii) Calcidiol binding; (xxiv) Vitamin transporter activity; (xxv) Metal ion-binding (xxvi) Heme transporter activity; (xxvii) Peptide antigen binding; (xxviii) Protein transmembrane transporter activity; (xxix) Ribonuclease activity; (xxx) Heme binding; (xxxi) Iron ion binding; (xxxii) Oxygen transportor binding; (xxxiii) Oxygen binding; (xxxiv) Calcium ion binding; (xxxv) Vitamin E binding; (xxxvi) Hormone binding; (xxxvii) Very-low-density lipoprotein particle receptor binding; (xxxviii) Protein homodimerisation activity; (xxxix) Metal chelating activity; (xl) Lipoprotein particle binding; (xli) Misfolded protein binding; (xlii) Ubiquitin protein ligase binding; (xliii) Structural constituent of eye lens; (xliv) Structural constituent of the cytoskeleton; (xlv) Calcium ion binding; (xlvi) Carbohydrate binding; (xlvii) Unfolded protein binding; (xlviii) Glycosaminoglycan binding enzyme regulator activity; (xlix) Selenium binding; (l) Glutathione peroxidase activity; (li)Transcription factor binding; (lii) Enzyme regulator activity; (liii) Acetyltransferase activator activity; (liv) Growth factor activity; (lv) Hormone activity; (lvi) Retinal binding and transporter activity.

**Table 2:** Modulation of essential physiological pathways in meningiomas*

Pathways	*P* -value	Observed number of candidates	Gene IDs of test set in subcategory	Category
Complement and coagulation cascades	1.45E^−55^	36	P01031, P07357, P07358, P04003, P05546, P04070, P08603, P09871, P13671, P10643, P00751, P01008, P01009, P08697, P01024, P05160, P00734, P01023, P07225, P06681, P02671, P01042, P02748, P07360, P20851, P00742, P05154, P05155, P00740, P05156, P04275, P03952, P00748, P00747, P0C0L5, Q96IY4	KEGG, BIOCARTA, PANTHER
ECM-receptor interaction	0.015	4	P02751, P04275, P04004, P07996	KEGG
PPAR signaling pathway	0.044	3	P02647, P02656, P02652	KEGG
Extrinsic prothrombin activation pathway	1.41E-^06^	6	P02671, P00742, P04070, P01008, P00734, P07225	BIOCARTA
Plasminogen activating cascade	1.45E^−06^	6	P02671, P36955, P08697, P00747, P08519, Q96IY4	PANTHER
FAS signaling pathway	0.045	1	P06396	PANTHER
Integrin signaling pathway	0.039	1	P02751	PANTHER
Hemostasis	3.08E^−17^	27	P04196, P02751, P04114, P04070, P02775, P01008, P09486, P01009, P08697, P05160, P07996, P00734, P01023, P07225, P02671, P02787, P01042, P02768, P00742, P05155, P00740, P10909, P04275, P03952, P00748, P00747, P02647	REACTOME
Signaling in immune system	3.55E-^06^	17	P02751, P01031, P07357, P07358, P04114, P02748, P04070, P07360, P09871, P13671, P10643, P01834, P00751, P01024, P00734, P06681, P07225	REACTOME
Integrin cell surface interactions	0.0362	5	P02671, P02751, P04275, P04004, P07996	REACTOME
Metabolism of lipids and lipoproteins	0.007	8	P04114, P02649, P02768, P02655, P06727, P02656, P02652, P02647	REACTOME

*Partial list of the different pathways associated with the differential expressed proteins identified in different grades of meningiomas in analysis using DAVID, PANTHER and GeneTrail functional annotation tools. Complete list is provided under the supplementary information (Table S8).

## References

[b1] CBTRUS (2005) Primary Brain Tumors in the United States: Statistical Report 1998–2002 Hinsdale, Ill, Central Brain Tumor Registry of the United States, 2005. (http://www.cbtrus.org/reports/2005-2006/2006report.pdf) [Accessed 31st January 2014].

[b2] Riemenschneider M. J., Perry A. & Reifenberger G. Histological classification and molecular genetics of meningiomas. Lancet Neurol. 5, 1045–1054 (2006). 17110285 10.1016/S1474-4422(06)70625-1

[b3] Mawrin C. & Perry A. Pathological classification and molecular genetics of meningiomas. J. Neurooncol. 99, 379–391 (2010). 20809251 10.1007/s11060-010-0342-2

[b4] Hallinan J. T., Hegde A. N. & Lim W. E. Dilemmas and diagnostic difficulties in meningioma. Clin. Radiol. 68, 837–844 (2013). 23623578 10.1016/j.crad.2013.03.007

[b5] Herrmann A. *et al.* Proteomic data in meningiomas: post-proteomic analysis can reveal novel pathophysiological pathways. J. Neurooncol. 104, 401–410 (2011). 21222216 10.1007/s11060-010-0526-9

[b6] Wiemels J., Wrensch M. & Claus E. B. Epidemiology and etiology of meningioma. J. Neurooncol. 99, 307–314 (2010). 20821343 10.1007/s11060-010-0386-3 PMC2945461

[b7] Bedard P. L., Hansen A. R., Ratain M. J. & Siu L. L. Tumour heterogeneity in the clinic. Nature 501, 355–364 (2013). 24048068 10.1038/nature12627 PMC5224525

[b8] Fisher R., Pusztai L. & Swanton C. Cancer heterogeneity: implications for targeted therapeutics. Br. J. Cancer 108, 479–485 (2013). 23299535 10.1038/bjc.2012.581 PMC3593543

[b9] Network T. C. Corrigendum: Comprehensive genomic characterization defines human glioblastoma genes and core pathways. Nature 494, 506 (2013). 10.1038/nature11903 23389443

[b10] Comprehensive molecular characterization of human colon and rectal cancer. Nature 487, 330–337 (2012). 22810696 10.1038/nature11252 PMC3401966

[b11] Ray S. *et al.* Proteomic technologies for the identification of disease biomarkers in serum: advances and challenges ahead. Proteomics. 11, 2139–2161 (2011). 21548090 10.1002/pmic.201000460

[b12] Khalil A. A. Biomarker discovery: a proteomic approach for brain cancer profiling. Cancer Sci. 98, 201–213 (2007). 17233837 10.1111/j.1349-7006.2007.00374.x PMC11158801

[b13] Gautam P. *et al.* Proteins with altered levels in plasma from glioblastoma patients as revealed by iTRAQ-based quantitative proteomic analysis. PLoS. One. 7, e46153 (2012) 23029420 10.1371/journal.pone.0046153 PMC3461020

[b14] Gollapalli K. *et al.* Investigation of serum proteome alterations in human glioblastoma multiforme. Proteomics. 12, 2378–2390 (2012). 22684992 10.1002/pmic.201200002

[b15] Whittle I. R. *et al.* Proteomic analysis of gliomas. Br. J. Neurosurg. 21, 576–582 (2007). 18071984 10.1080/02688690701721691

[b16] Polisetty R. V. *et al.* LC-MS/MS analysis of differentially expressed glioblastoma membrane proteome reveals altered calcium signaling and other protein groups of regulatory functions. Mol. Cell Proteomics. 11, M111 (2012). 10.1074/mcp.M111.013565 PMC3433906 22219345

[b17] Okamoto H. *et al.* Comparative proteomic profiles of meningioma subtypes. Cancer Res. 66, 10199–10204 (2006). 17047085 10.1158/0008-5472.CAN-06-0955

[b18] Wibom C. *et al.* Proteomic profiles differ between bone invasive and noninvasive benign meningiomas of fibrous and meningothelial subtype. J. Neurooncol. 94, 321–331 (2009). 19350207 10.1007/s11060-009-9865-9

[b19] Wiemels J. L. *et al.* Assessment of autoantibodies to meningioma in a population-based study. Am. J. Epidemiol. 177, 75–83 (2013). 23221727 10.1093/aje/kws221 PMC3590036

[b20] Kim J. H. *et al.* Proteome analysis of human cerebrospinal fluid as a diagnostic biomarker in patients with meningioma. Med. Sci. Monit. 18, BR450–BR460 (2012). 23111736 10.12659/MSM.883538 PMC3560610

[b21] Harrison M. J. *et al.*Radiation-induced meningiomas experience at the mount Sinai Hospital and review of the literature. J Neurosurg. 75, 564–74 (1991). 1885974 10.3171/jns.1991.75.4.0564

[b22] Ignjatovic V. *et al.* Age-related differences in plasma proteins: How plasma poteins change from neonates to adults. PloS. One. 6, e17213 (2011). 21365000 10.1371/journal.pone.0017213 PMC3041803

[b23] Alaiya A. A., Franzen B., Auer G. & Linder S. Cancer proteomics: from identification of novel markers to creation of artifical learning models for tumor classification. Electrophoresis 21, 1210–1217 (2000). 10786893 10.1002/(SICI)1522-2683(20000401)21:6<1210::AID-ELPS1210>3.0.CO;2-S

[b24] Simpson R. J. & Dorow D. S. Cancer proteomics: from signaling networks to tumor markers. Trends Biotechnol. 19, S40–S48 (2001). 11780970 10.1016/S0167-7799(01)01801-7

[b25] Constantini S. *et al.* Thromboembolic phenomena in neurosurgical patients operated upon for primary and metatstatic brain tumors. Acta neurochir. 109, 93–7 (1991). 1858538 10.1007/BF01403001

[b26] Hamilton M. G., Hull R. D. & Pineo G. F. Venous thromboembolism in neurosurgery and neurology patients: a review. Neurosurgery. 34, 280–96 (2001). 10.1227/00006123-199402000-00012 8177390

[b27] Boccaccio C. & Medico E. Cancer and blood coagulation. Cell Mol. Life Sci. 63, 1024–1027 (2006). 16612563 10.1007/s00018-005-5570-9 PMC11135989

[b28] Rickles F. R. & Levine M. N. Epidemiology of thrombosis in cancer. Acta Haematol. 106, 6–12 (2001). 11549771 10.1159/000046583

[b29] Falanga A., Marchetti M. & Vignoli A. Coagulation and cancer: biological and clinical aspects. J. Thromb. Haemost. 11, 223–233 (2013). 23279708 10.1111/jth.12075

[b30] Thoron L. & Arbit E. Hemostatic changes in patients with brain tumors. J. Neurooncol. 22, 87–100 (1994). 7745471 10.1007/BF01052885

[b31] Ren B., Yee K. O., Lawler J. & Khosravi-Far R. Regulation of tumor angiogenesis by thrombospondin-1. Biochim. Biophys. Acta 1765, 178–188 (2006). 16406676 10.1016/j.bbcan.2005.11.002

[b32] Rutkowski M. J., Sughrue M. E., Kane A. J., Mills S. A. & Parsa A. T. Cancer and the complement cascade. Mol. Cancer Res. 8, 1453–1465 (2010). 20870736 10.1158/1541-7786.MCR-10-0225

[b33] Gorter A. & Meri S. Immune evasion of tumor cells using membrane-bound complement regulatory proteins. Immunol. Today 20, 576–582 (1999). 10562709 10.1016/s0167-5699(99)01537-6

[b34] Desgrosellier J. S. & Cheresh D. A. Integrins in cancer: biological implications and therapeutic opportunities. Nat. Rev. Cancer 10, 9–22 (2010). 20029421 10.1038/nrc2748 PMC4383089

[b35] Hehlgans S., Haase M. & Cordes N. Signalling via integrins: implications for cell survival and anticancer strategies. Biochim. Biophys. Acta 1775, 163–180 (2007). 17084981 10.1016/j.bbcan.2006.09.001

[b36] Levental K. R. *et al.* Matrix crosslinking forces tumor progression by enhancing integrin signaling. Cell 139, 891–906 (2009). 19931152 10.1016/j.cell.2009.10.027 PMC2788004

[b37] Giancotti F. G. & Ruoslahti E. Integrin signaling. Science 285, 1028–1032 (1999). 10446041 10.1126/science.285.5430.1028

[b38] Prasanna P., Thibault A., Liu L. & Samid D. Lipid metabolism as a target for brain cancer therapy: synergistic activity of lovastatin and sodium phenylacetate against human glioma cells. J. Neurochem. 66, 710–716 (1996). 8592143 10.1046/j.1471-4159.1996.66020710.x

[b39] Kreuter J. *et al.* Covalent attachment of apolipoprotein A-I and apolipoprotein B-100 to albumin nanoparticles enables drug transport into the brain. J. Control Release 118, 54–58 (2007). 17250920 10.1016/j.jconrel.2006.12.012

[b40] Moore L. E. *et al.* Evaluation of apolipoprotein A1 and posttranslationally modified forms of transthyretin as biomarkers for ovarian cancer detection in an independent study population. Cancer Epidemiol. Biomarkers Prev. 15, 1641–1646 (2006). 16985025 10.1158/1055-9965.EPI-05-0980

[b41] Malik G. *et al.* Serum levels of an isoform of apolipoprotein A-II as a potential marker for prostate cancer. Clin. Cancer Res. 11, 1073–1085 (2005). 15709174

[b42] Petrak J. *et al.* Deja vu in proteomics. A hit parade of repeatedly identified differentially expressed proteins. Proteomics. 8, 1744–1749 (2008). 18442176 10.1002/pmic.200700919

[b43] Ray S. *et al.* Proteomic investigation of falciparum and vivax malaria for identification of surrogate protein markers. PLoS. One. 7, e41751 (2012). 22912677 10.1371/journal.pone.0041751 PMC3415403

[b44] Thomas P. D. *et al.* Applications for protein sequence-function evolution data: mRNA/protein expression analysis and coding SNP scoring tools. Nucleic Acids Res. 34, W645–W650 (2006). 16912992 10.1093/nar/gkl229 PMC1538848

[b45] Huang D. W., Sherman B. T. & Lempicki R. A. Systematic and integrative analysis of large gene lists using DAVID bioinformatics resources. Nat. Protoc. 4, 44–57 (2009). 19131956 10.1038/nprot.2008.211

[b46] Backes C. *et al.*GeneTrail--advanced gene set enrichment analysis. Nucleic. Acids. Res. 35 (Web Server issue) (2007). 10.1093/nar/gkm323 PMC1933132 17526521

