# Supplementary material for: Quantitative Proteomic Analysis of Meningiomas for the Identification of Surrogate Protein Markers
Source: Sci Rep. 2014 Nov 21;4:7140. doi: 10.1038/srep07140 (PMC5382771; doi:10.1038/srep07140)
Supplement: Supplementary Information [file srep07140-s1.docx]

**Original Article (Supplementary Information)**

**Quantitative Proteomic Analysis of Meningiomas for the Identification of Surrogate Protein Markers**

Samridhi Sharma1†, Sandipan Ray 1†, Aliasgar Moiyadi 2, Epari Sridhar 3

and Sanjeeva Srivastava1*****

1 Wadhwani Research Center for Biosciences and Bioengineering, Department of Biosciences and Bioengineering, Indian Institute of Technology Bombay, Powai, Mumbai 400076, India

2 Department of Neurosurgery, Advanced Center for Treatment Research and Education in

Cancer, Tata Memorial Center, Kharghar, Navi Mumbai 410210, India

3 Department of Pathology, Tata Memorial Hospital, Mumbai 400012, India.

† Both authors contributed equally to the preparation of this manuscript

*Correspondence: Dr. Sanjeeva Srivastava, Department of Biosciences and Bioengineering, IIT Bombay, Mumbai 400 076, India: E-mail: [sanjeeva@iitb.ac](mailto:sanjeeva@iitb.ac.in).in

Phone: +91-22-2576-7779, Fax: +91-22-2572-3480

**Running Title:** Proteomic Analysis of Different Grades of Meningioma

**
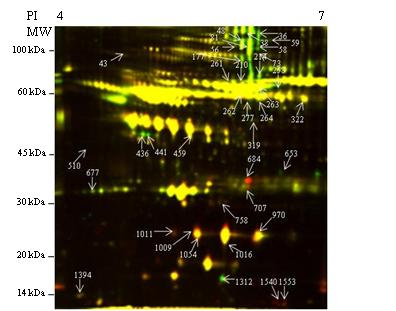
**

**A**


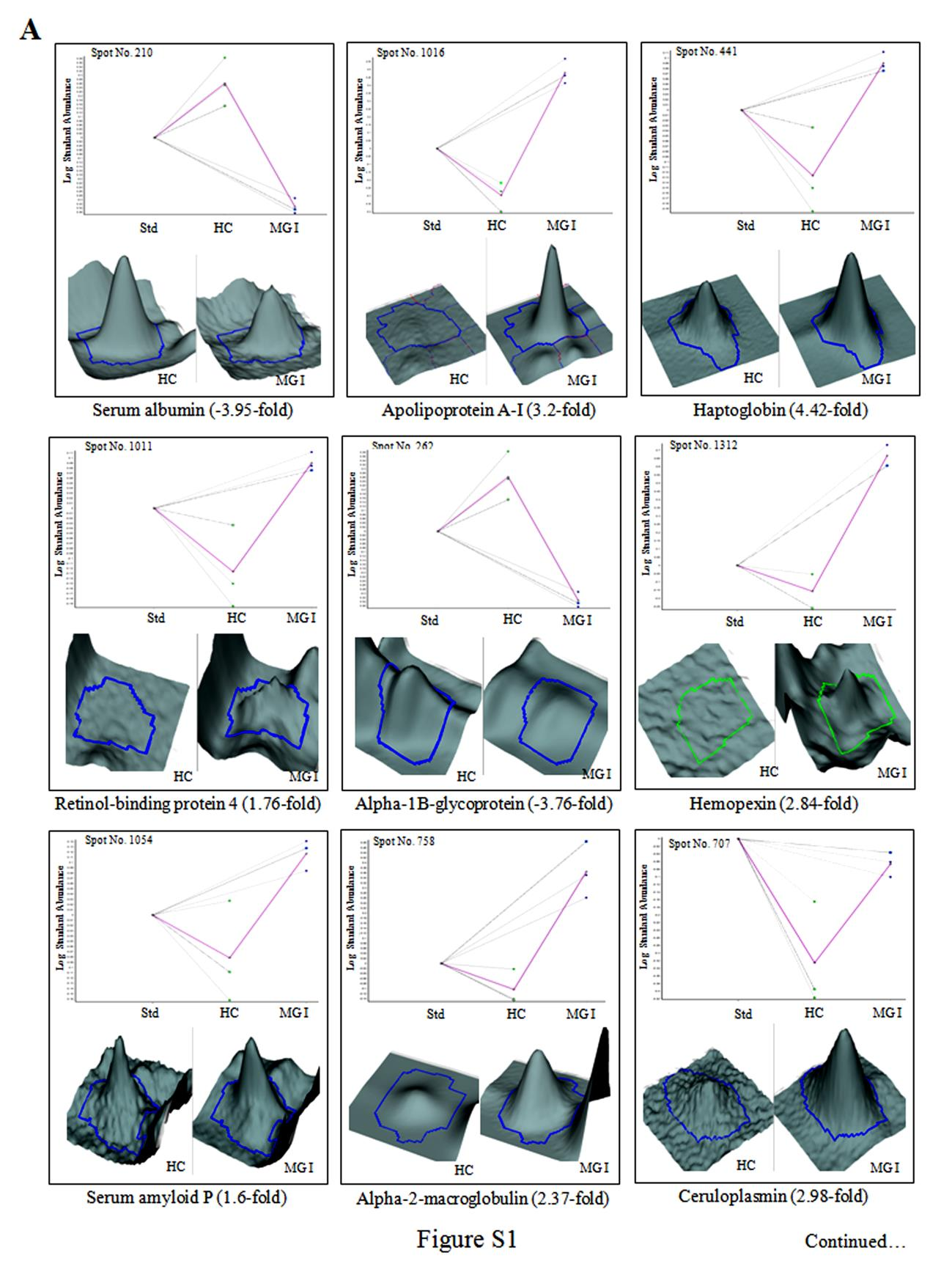


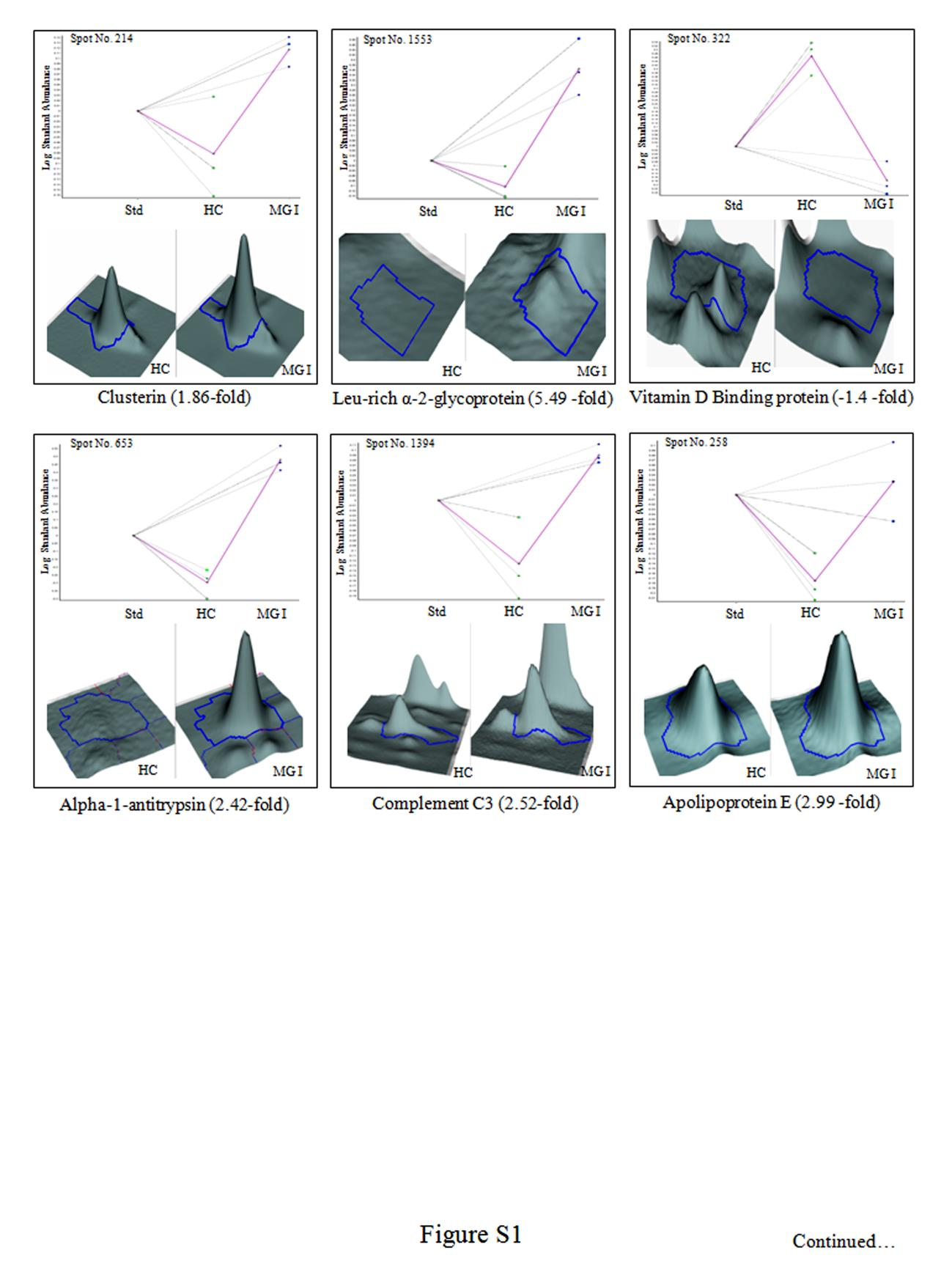


**B**


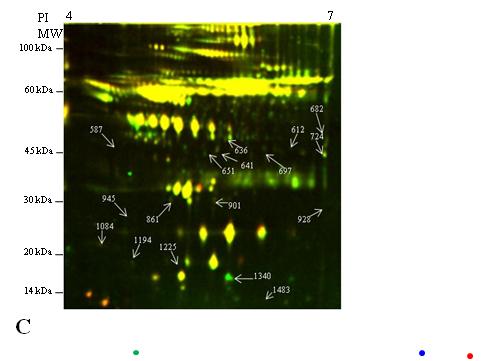


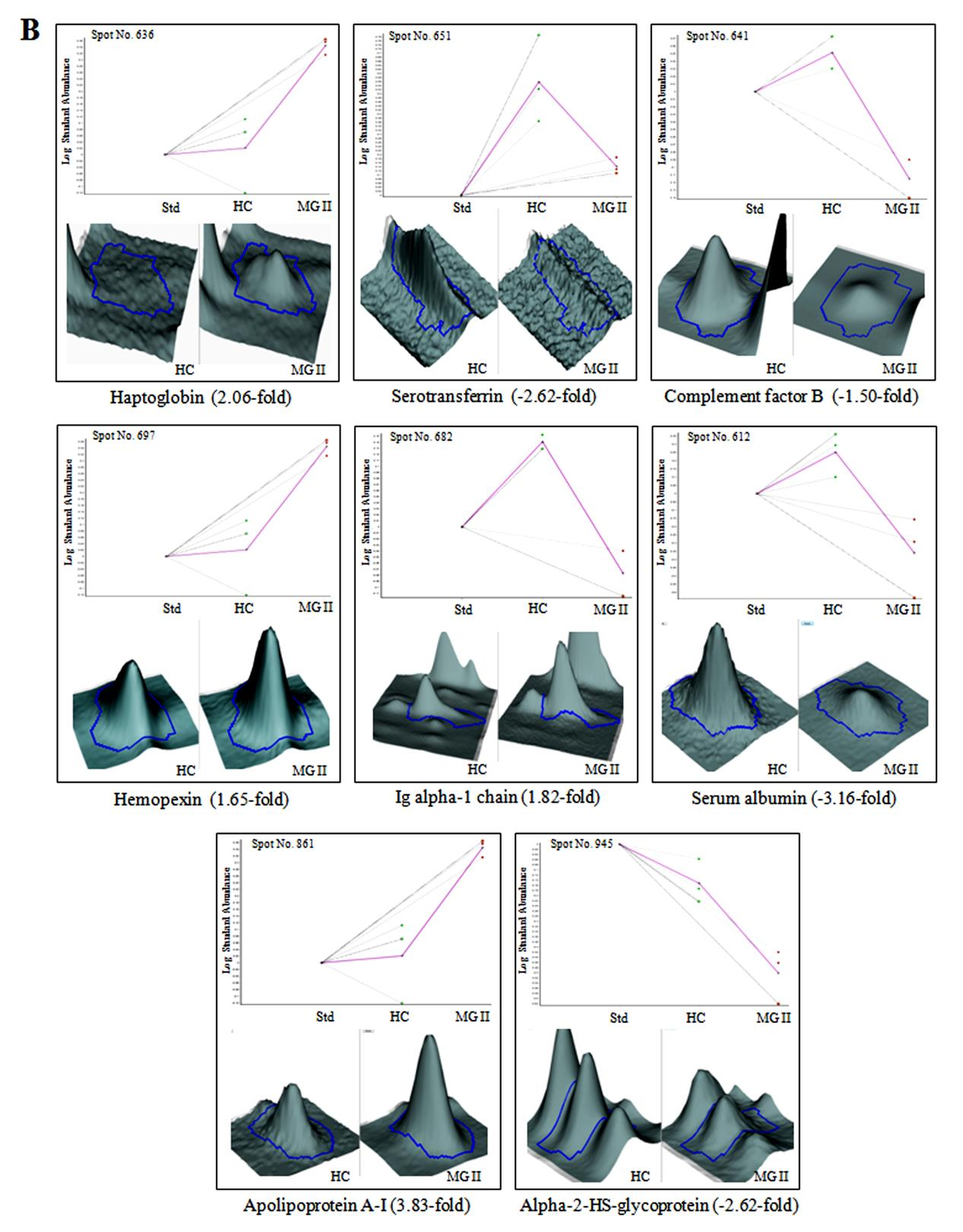


**Figure S1.** (A) Representative 2D- DIGE image (Cy3 and Cy5 channel overlap) and trends of differentially expressed proteins in meningioma grade I (compared to healthy controls) visualized in 2D-DIGE. (B) Representative 2D- DIGE image (Cy3 and Cy5 channel overlap) Trends of differentially expressed proteins in meningioma grade II (compared to healthy controls) visualized in 2D-DIGE.


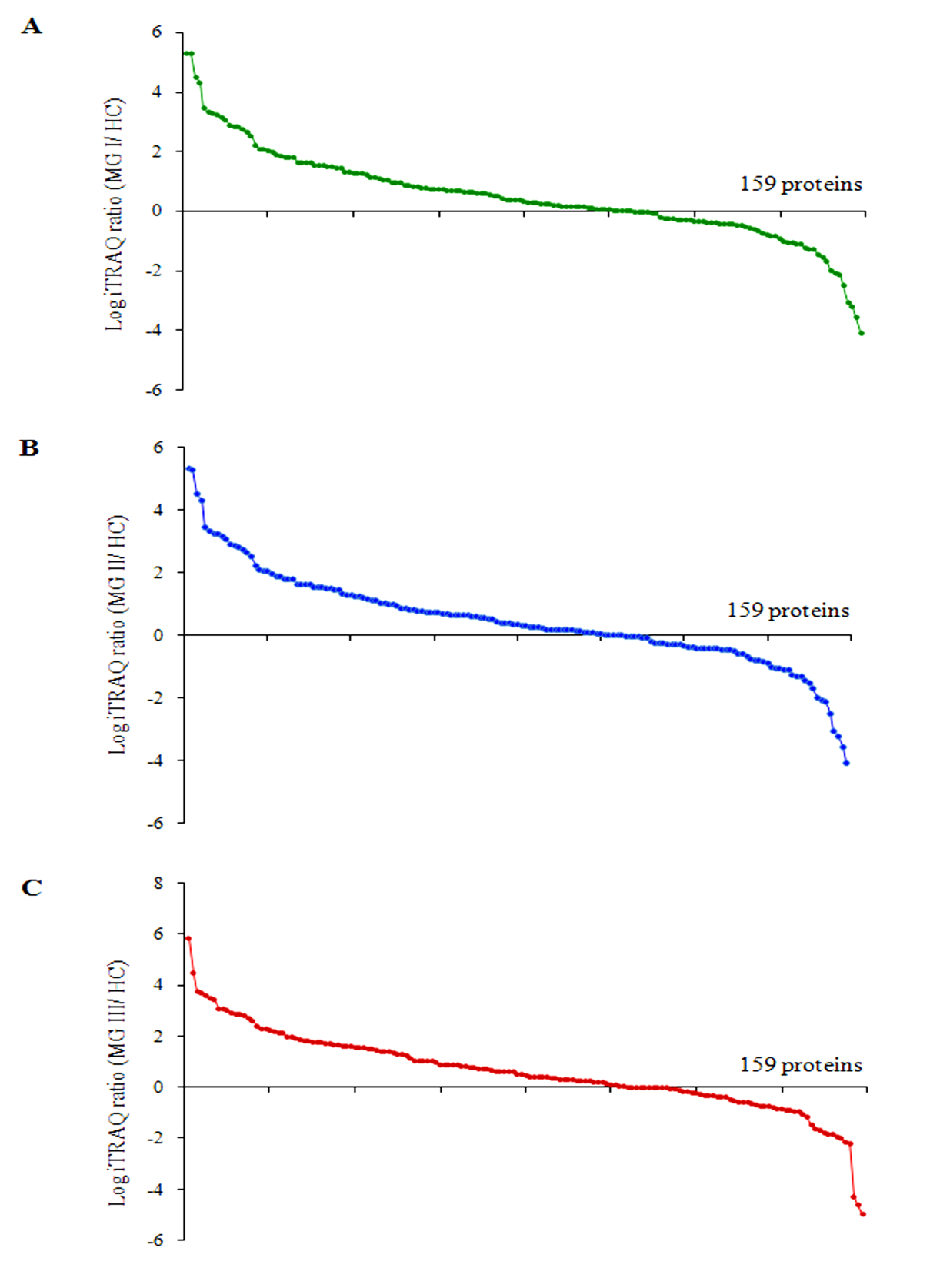


157 proteins

157 proteins

157 proteins

**Figure S2.** The distribution of the differentially expressed proteins in different grades of meningiomas**;**

grade I (A), II (B) and III (C) identified in iTRAQ**.**


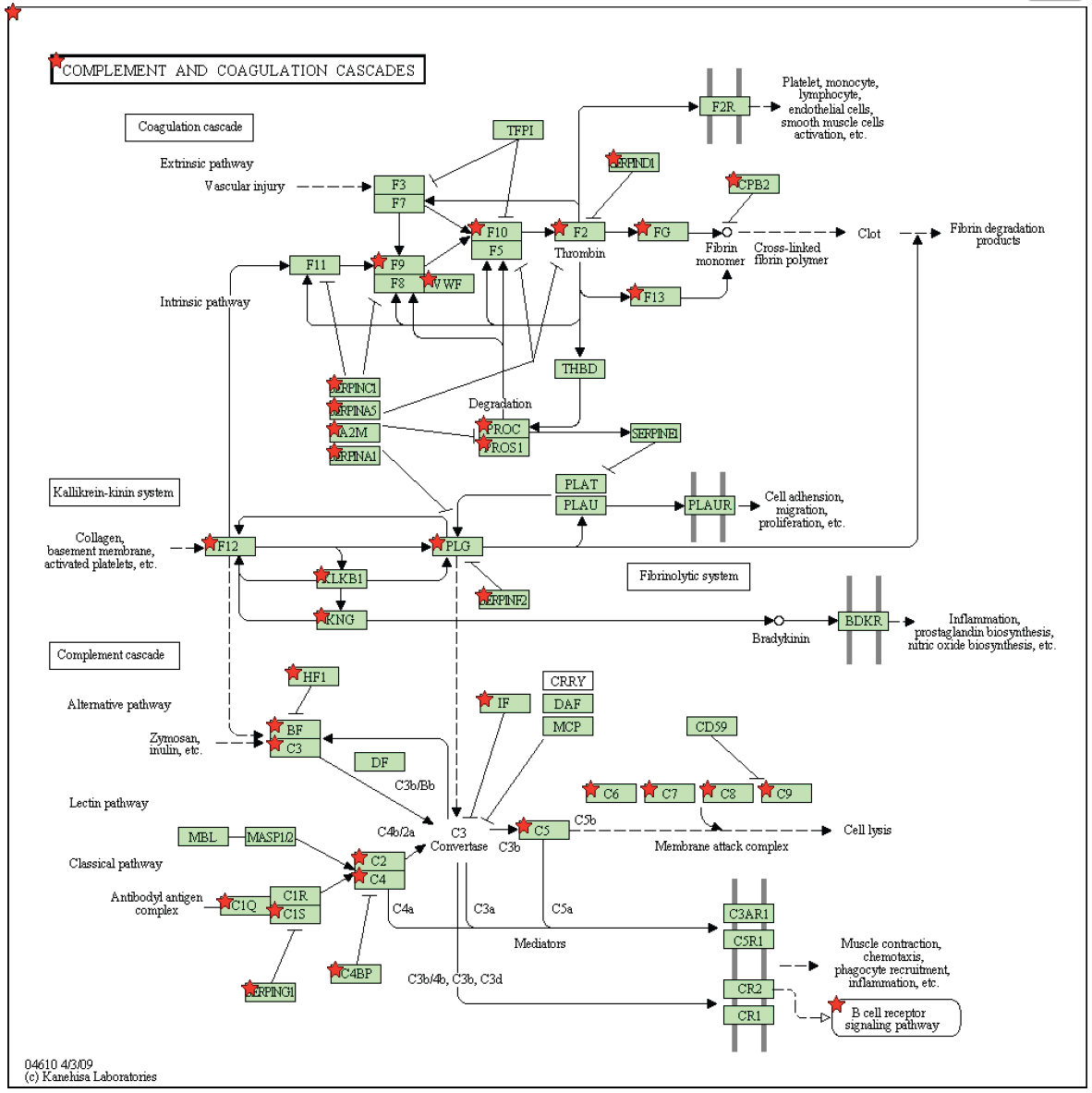


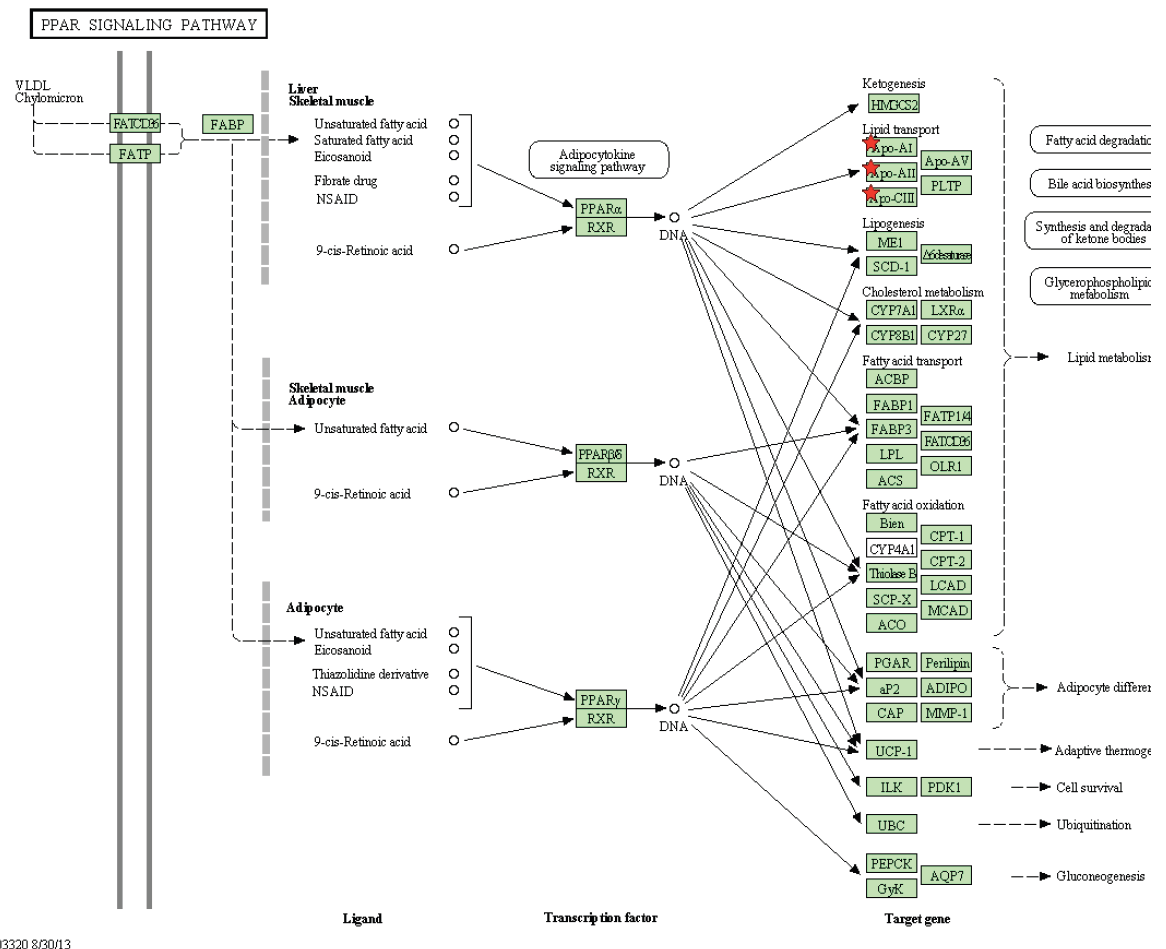

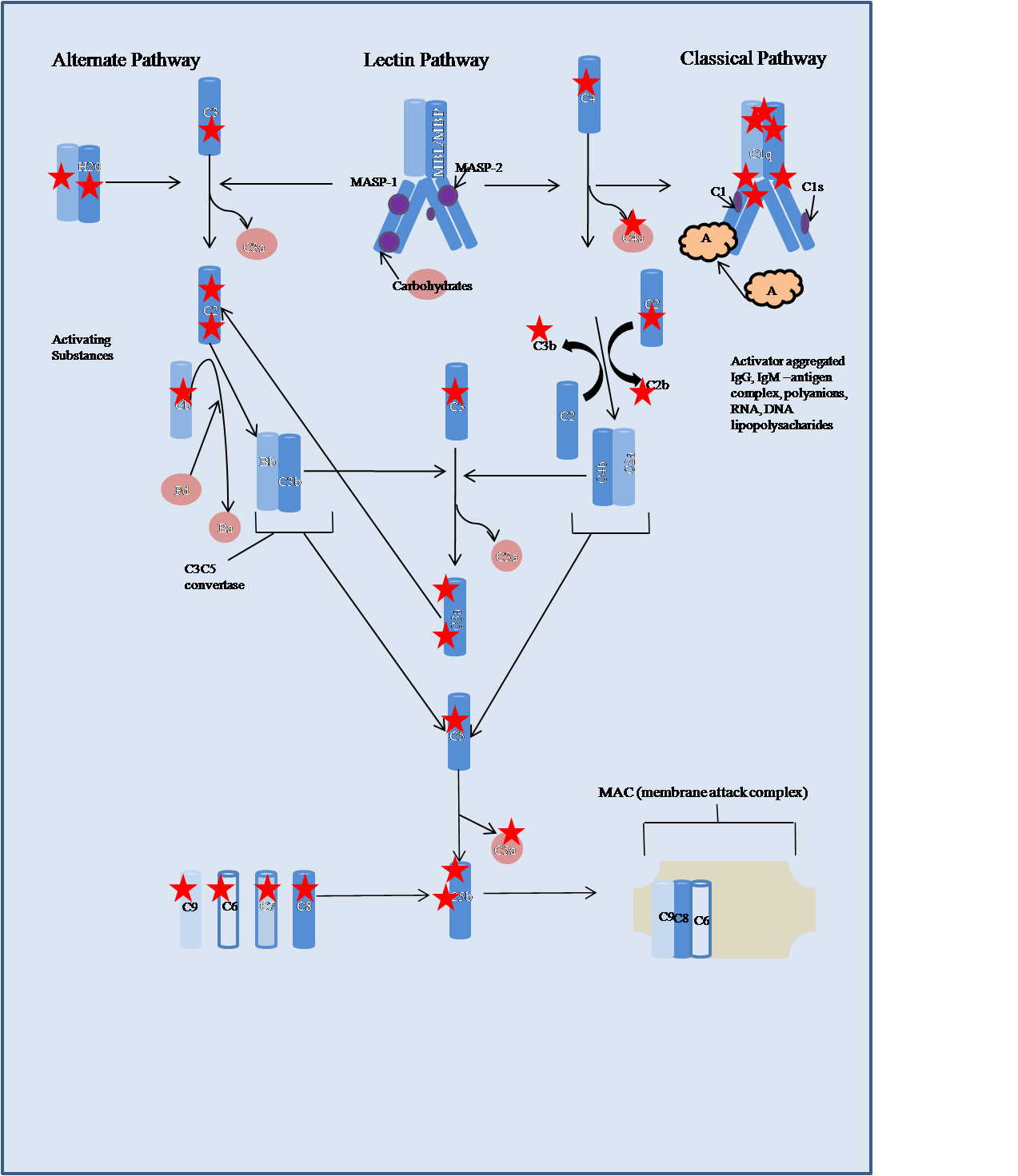


Continued…


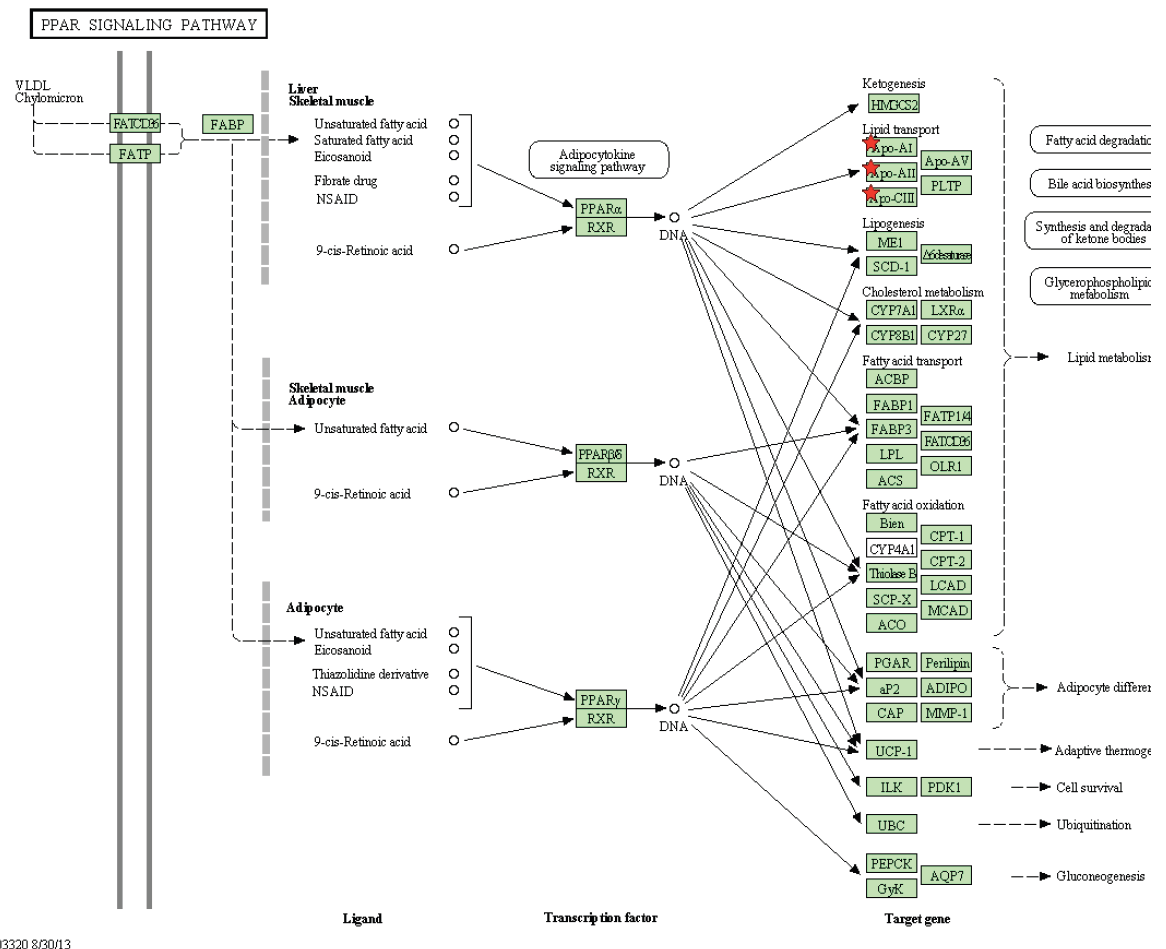
 Continued…

**D. ECM signalling**


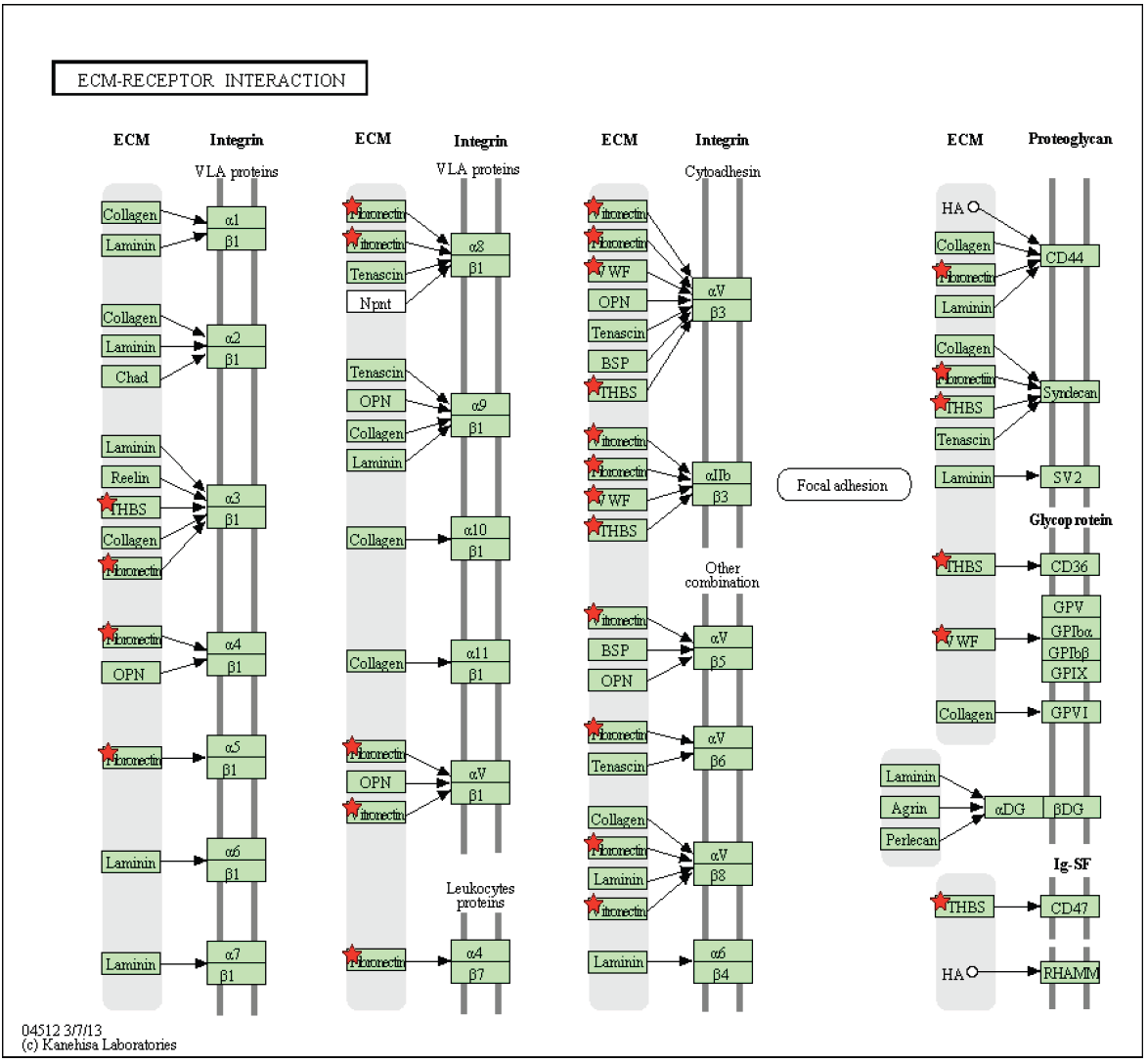


**Figure S3.** Different biological pathways modulated in meningiomas obtained in DAVID analysis.


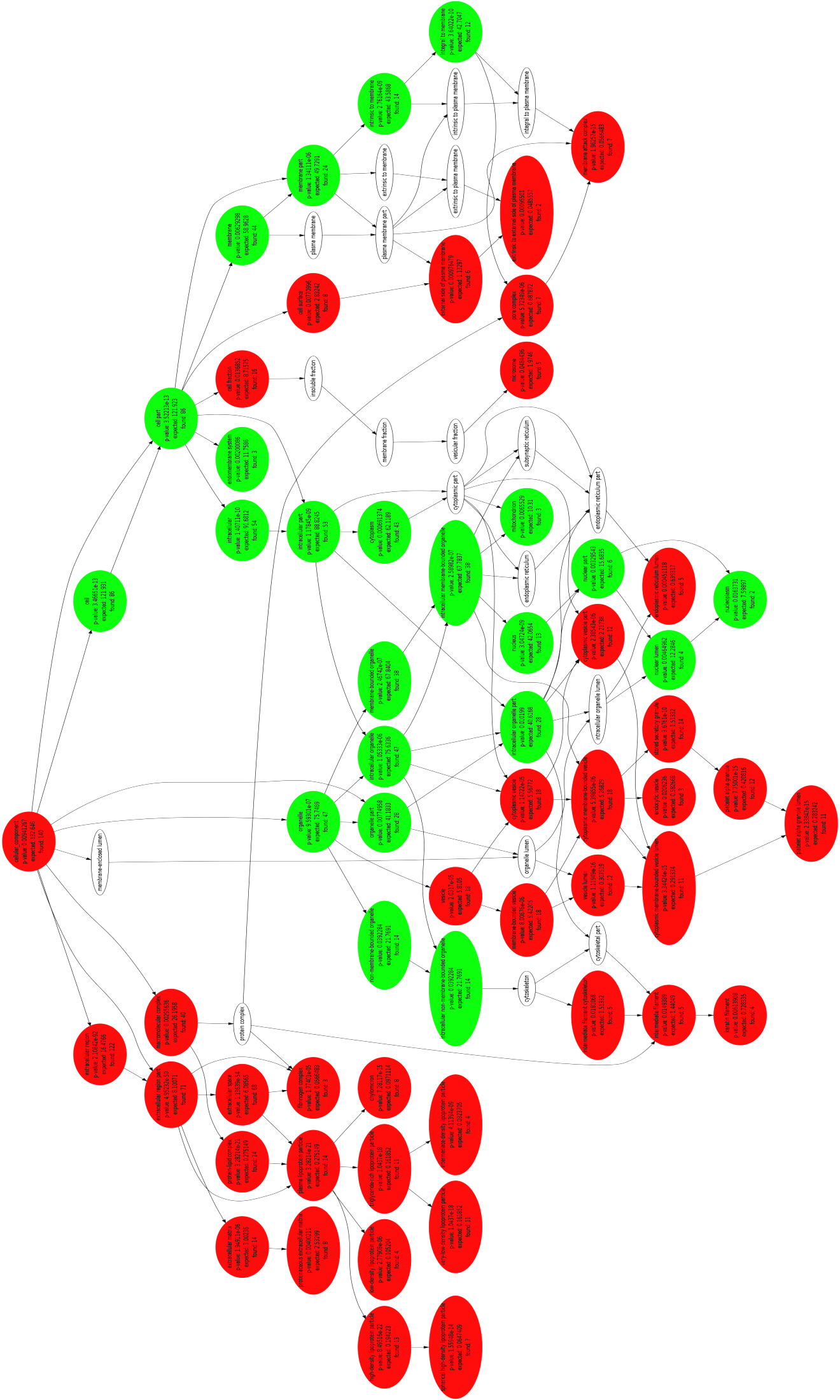
**A**

**B**


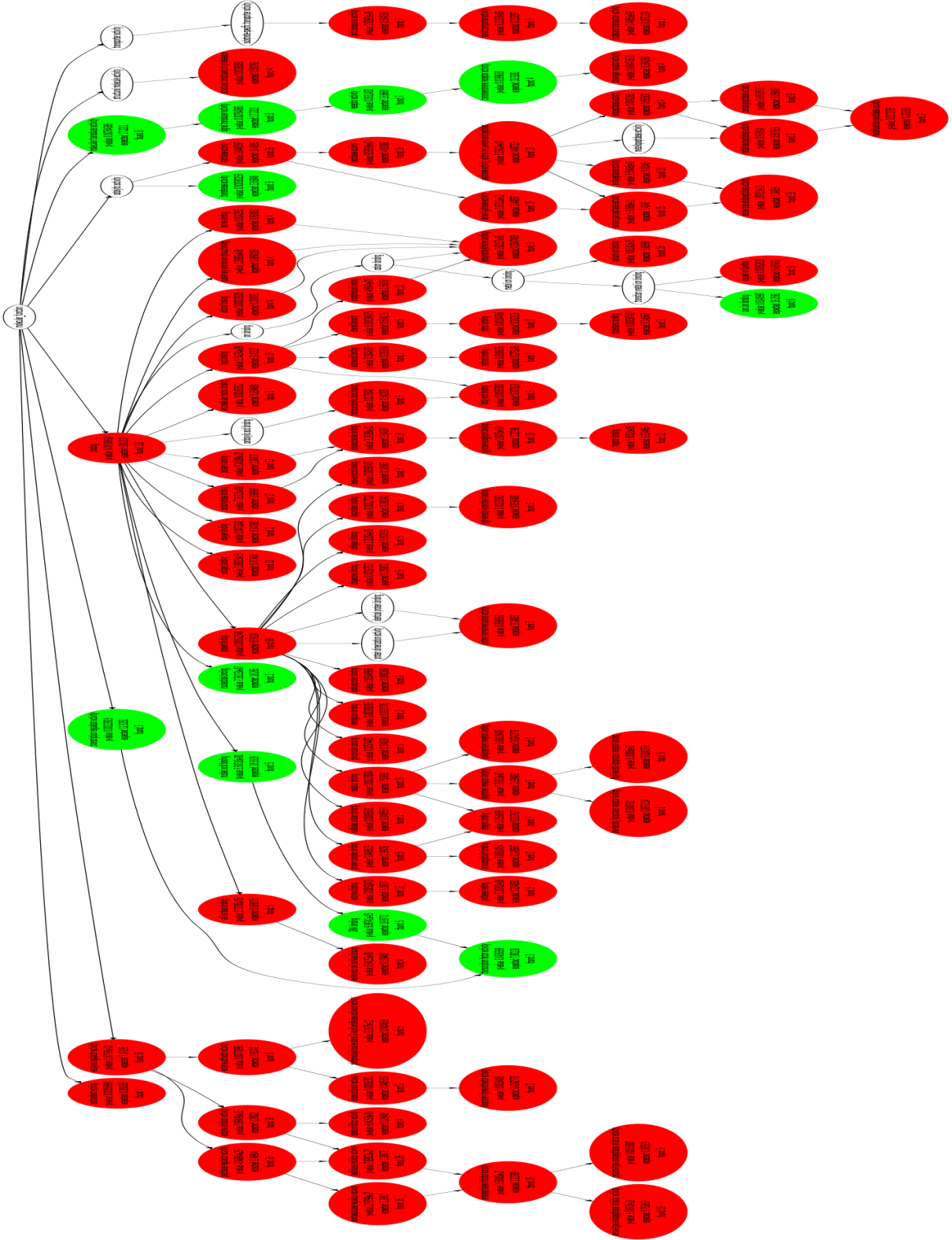


**Figure S4.** Cellular and molecular sub-trees associated with the differentially expressed proteins identified in meningiomas.


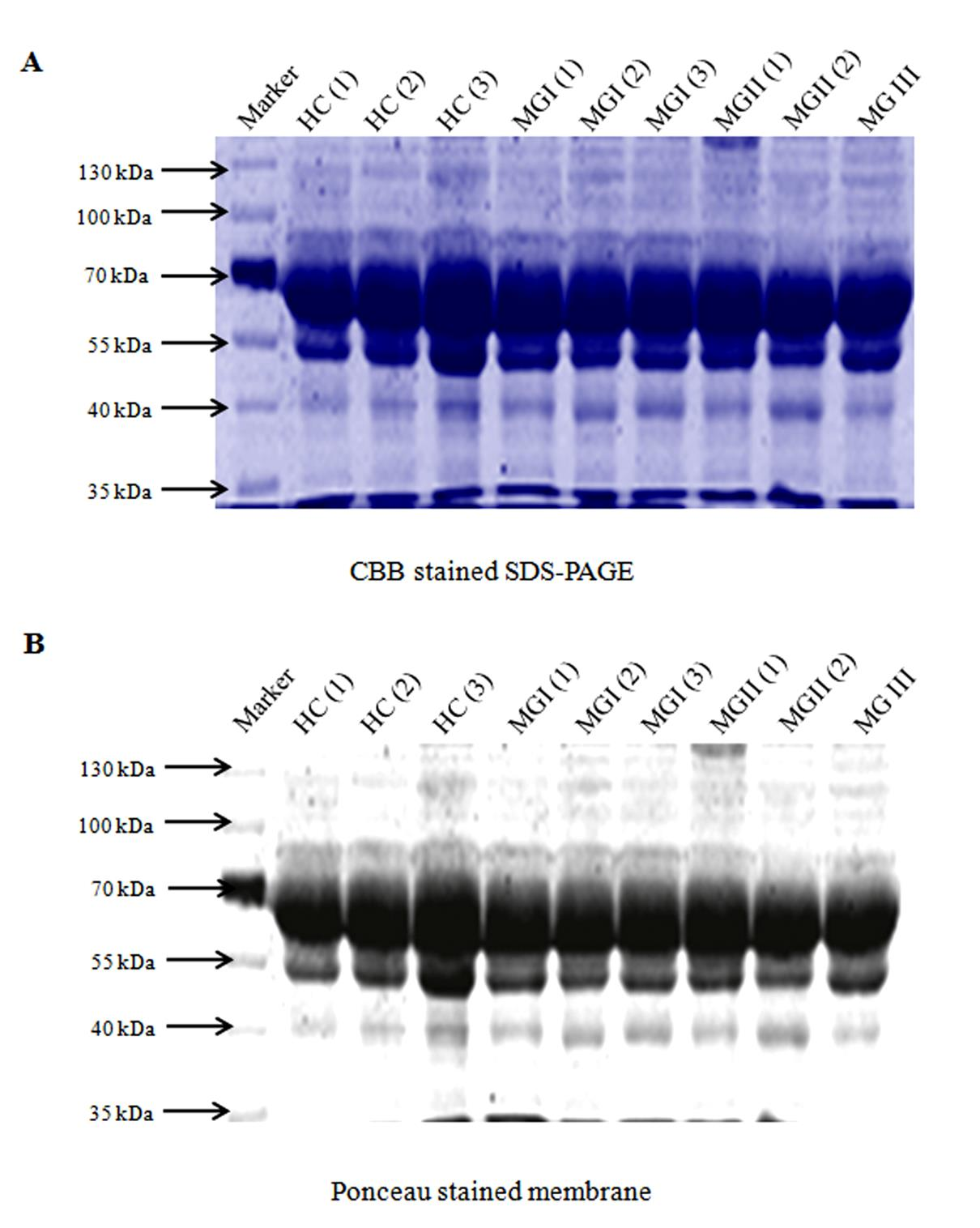


**Figure S5.** Equal loading of protein samples during western blot experiment. Representative CBB stained SDS-PAGE gel (A) and Ponceau stained blot (B) containing the resolved proteins depicting equal loading (50 µg) of the samples (meningioma patients and healthy subjects) in every lane during western blot experiment.


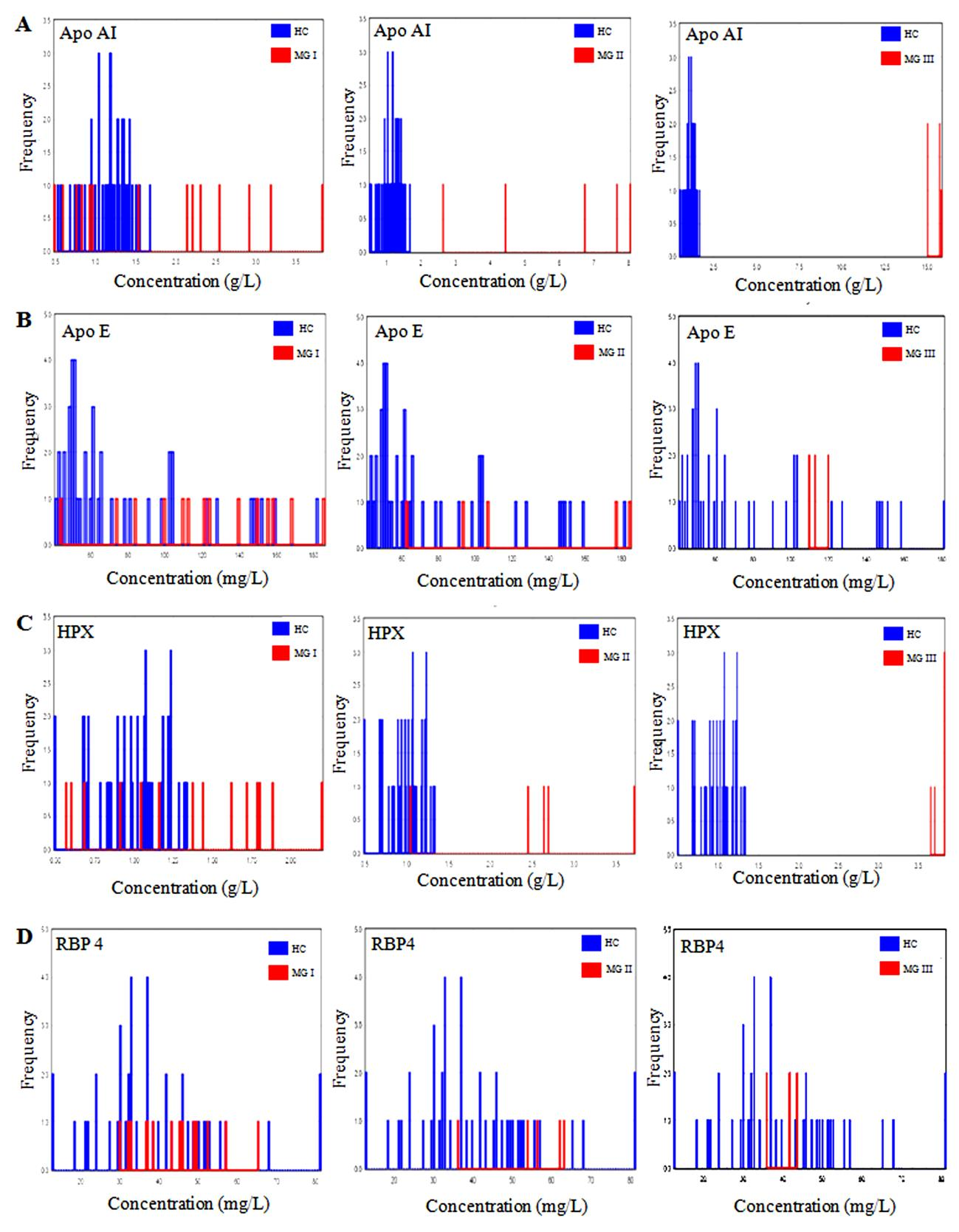


**Figure S6.** Frequency distribution of serum concentrations of Apo E, HPX, Apo A1 and RBP4 in healthy controls and different grades of meningioma patients measured by ELISA.


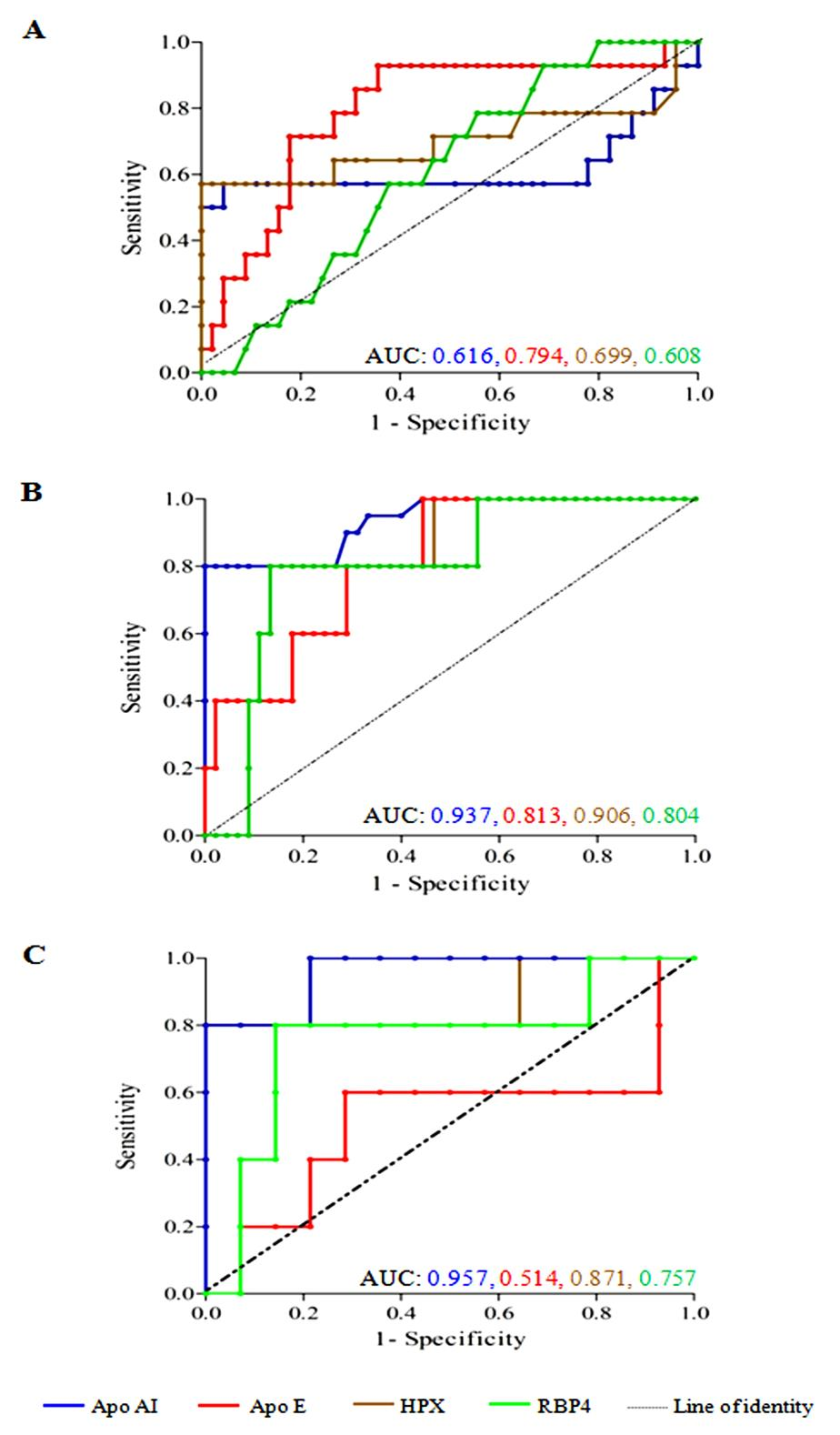


**Figure S7.** Receiver operating characteristic (ROC) curves depicting accuracy of 4 classifier proteins; Apo E, HPX, Apo A1 and RBP4 for prediction of grade I and grade II meningiomas

**
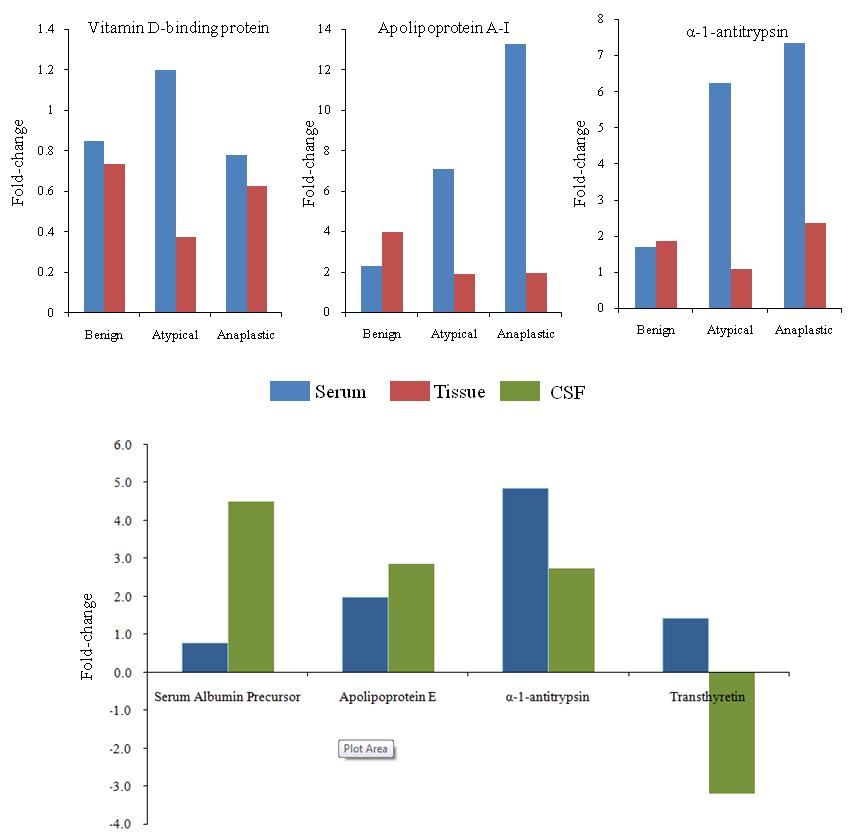
**

**Figure S8.** Comparison of the fold changes of the differentially expressed proteins identified from iTRAQ data with proteins reported in published literature in tissue and CSF samples. Differential expression of proteins in tissue and CSF samples are obtained from reference number 17 and 20 respectively.

**Supplementary Tables**

**Table S1.** Demographics and clinical details of the meningioma patients and healthy subjects

| **Sl.No.** | **Patient ID** | **Diagnosis** | **Grade** | **Age** | **Sex** |
| --- | --- | --- | --- | --- | --- |
| **1** | CJ 14742 | Meningothelial Meningioma | Gr. I | 29 | M |
| **2** | CJ4231 | Transitional Meningioma | Gr. I | 35 | F |
| **3** | CH 30545 | Transitional meningioma | Gr. I | 17 | M |
| **4** | CF 4450 | MeningothelialMeningioma | Gr. I | 55 | F |
| **5** | CH 24953 | Transitional meningioma | Gr. I | 41 | F |
| **6** | CJ 20717 | MeningothelialMeningioma | Gr. I | 39 | F |
| **7** | CJ 20619 | MeningothelialMeningioma | Gr. I | 41 | F |
| **8** | CJ 29822 | Transitional meningioma | Gr. I | 50 | F |
| **9** | CJ 29452 | Transitional meningioma | Gr. I | 42 | F |
| **10** | CJ 29583 | Meningothelial meningioma | Gr. I | 44 | F |
| **11** | CJ 9179 | Transitional meningioma | Gr. I | 57 | F |
| **12** | CJ 26538 | Lipomatous meningioma | Gr. I | 45 | F |
| **13** | CK 726 | MeningothelialMeningioma | Gr. I | 56 | F |
| **14** | CK 985 | Angiomatous meningioma | Gr. I | 55 | F |
| **15** | CJ 15491 | Atypical meningioma | Gr. II | 67 | M |
| **16** | CJ 15753 | Atypical meningiom | Gr. II | 46 | F |
| **17** | CH 17967 | Atypical meningioma | Gr. II | 58 | M |
| **18** | CJ 3577 | Atypical meningioma | Gr. II | 43 | F |
| **19** | CK 7710 | Atypical meningioma | Gr. II | 55 | F |
| **20** | CJ6540 | Pappalary Meningioma | Gr. III | 61 | M |

in meningioma grade I (compared to healthy controls) visualized in 2D-DIGE

| **Sl. No.** | **Master No.** | **Appearance** | **T-test** | **Av. Ratio** |
| --- | --- | --- | --- | --- |
| 1 | 684 | 9 (9) | 0.00039 | -6.23 |
| 2 | 441 | 9 (9) | 0.00089 | 4.42 |
| 3 | 263 | 9 (9) | 0.0011 | -3.88 |
| 4 | 277 | 9 (9) | 0.0011 | -4.04 |
| 5 | 262 | 9 (9) | 0.0013 | -3.76 |
| 6 | 264 | 9 (9) | 0.0019 | -3.4 |
| 7 | 707 | 9 (9) | 0.0036 | 2.98 |
| 8 | 1312 | 9 (9) | 0.0054 | 2.84 |
| 9 | 653 | 9 (9) | 0.0084 | 2.42 |
| 10 | 677 | 9 (9) | 0.012 | -2.67 |
| 11 | 758 | 9 (9) | 0.012 | 2.37 |
| 12 | 1009 | 9 (9) | 0.012 | 1.63 |
| 13 | 261 | 9 (9) | 0.013 | -2.02 |
| 14 | 214 | 9 (9) | 0.019 | 1.86 |
| 15 | 657 | 9 (9) | 0.019 | -2.7 |
| 16 | 436 | 9 (9) | 0.02 | 2.08 |
| 17 | 1054 | 9 (9) | 0.021 | 1.6 |
| 18 | 58 | 9 (9) | 0.028 | 1.56 |
| 19 | 1011 | 9 (9) | 0.035 | 1.76 |
| 20 | 1553 | 9 (9) | 0.00042 | 5.49 |
| 21 | 1016 | 9 (9) | 0.0011 | 3.2 |
| 22 | 322 | 6 (9) | 0.0015 | -1.4 |
| 23 | 266 | 6 (9) | 0.0045 | -2.55 |
| 24 | 56 | 6 (9) | 0.0057 | -2.31 |
| 25 | 510 | 6 (9) | 0.0075 | -6.23 |
| 26 | 210 | 6 (9) | 0.018 | -3.95 |
| 27 | 43 | 6 (9) | 0.019 | -1.62 |
| 28 | 1595 | 6 (9) | 0.021 | 6.57 |
| 29 | 590 | 6 (9) | 0.022 | 3.08 |
| 30 | 319 | 6 (9) | 0.027 | -1.63 |
| 31 | 177 | 6 (9) | 0.032 | -2.93 |
| 32 | 1394 | 6 (9) | 0.032 | 2.52 |
| 33 | 36 | 6 (9) | 0.034 | -4.13 |
| 34 | 459 | 6 (9) | 0.034 | 1.56 |
| 35 | 48 | 6 (9) | 0.037 | -3.46 |
| 36 | 59 | 6 (9) | 0.037 | -2.73 |

| 37 | 81 | 6 (9) | 0.037 | -3.99 |
| --- | --- | --- | --- | --- |
| 38 | 38 | 6 (9) | 0.043 | 3.96 |
| 39 | 73 | 6 (9) | 0.044 | 4.17 |
| 40 | 970 | 6 (9) | 0.046 | 2.43 |
| 41 | 258 | 6 (9) | 0.047 | 2.99 |

spots in meningioma grade II (compared to healthy controls) visualized in 2D-DIGE

| **Sl. No.** | **Master No.** | **Appearance** | **T-test** | **Av. Ratio** |
| --- | --- | --- | --- | --- |
| 1 | 1084 | 9 (9) | 0.00067 | 1.74 |
| 2 | 632 | 6 (9) | 0.0039 | 1.49 |
| 3 | 1340 | 9 (9) | 0.0071 | 6.19 |
| 4 | 1483 | 9 (9) | 0.0083 | -2.01 |
| 5 | 1435 | 9 (9) | 0.011 | -2.23 |
| 6 | 636 | 9 (9) | 0.012 | 2.06 |
| 7 | 612 | 9 (9) | 0.016 | -3.16 |
| 8 | 641 | 6 (9) | 0.019 | -1.50 |
| 9 | 861 | 9 (9) | 0.019 | 3.83 |
| 10 | 945 | 9 (9) | 0.024 | -2.62 |
| 11 | 1194 | 9 (9) | 0.024 | -1.9 |
| 12 | 682 | 6 (9) | 0.025 | 1.82 |
| 13 | 928 | 9 (9) | 0.027 | 1.55 |
| 14 | 724 | 9 (9) | 0.029 | 2.83 |
| 15 | 915 | 9 (9) | 0.029 | 1.86 |
| 16 | 697 | 6 (9) | 0.031 | 1.65 |
| 17 | 492 | 6 (9) | 0.033 | -1.71 |
| 18 | 901 | 9 (9) | 0.035 | 1.53 |
| 19 | 582 | 6 (9) | 0.037 | 1.47 |
| 20 | 251 | 6 (9) | 0.042 | 2.09 |
| 21 | 651 | 9 (9) | 0.042 | -2.62 |
| 22 | 993 | 6 (9) | 0.042 | -1.52 |
| 23 | 921 | 9 (9) | 0.049 | 1.62 |
| 24 | 587 | 9 (9) | 0.05 | -2.02 |

visualized in 2D-DIGE#

[Analysis Type: Combined (MS+MS/MS); Database: SwissProt; Taxonomy: Human]

| **Sl**  **No.** | **Mast**  **er**  **ID** | **Fold-**  **change** | Name of protein with  UniProt  **accession number** | **MW**  **(kDa)** | **Protein**  **score** | **No. of**  **matched peptides** | **Peptide sequence** |
| --- | --- | --- | --- | --- | --- | --- | --- |
| 1 | 210 | -3.95 | (P02768) Serum albumin | 69.32 | 456 | 30 | CCKADDK YLYEIAR LCTVATLR DDNPNLPR LKCASLQK FQNALLVR LVNEVTEFAK FKDLGEENFK HPDYSVVLLLR AVMDDFAAFVEK RHPDYSVVLLLR CCAAADPHECYAK DVFLGMFLYEYAR  KVPQVSTPTLVEVSR QEPERNECFLQHK HPYFYAPELLFFAK RHPYFYAPELLFFAK HPYFYAPELLFFAKR RPCFSALEVDETYVPK VFDEFKPLVEEPQNLIK EFNAETFTFHADICTLSEK ALVLIAFAQYLQQCPFEDHVK TCVADESAENCDKSLHTLFGDK MPCAEDYLSVVLNQLCVLHEK EFNAETFTFHADICTLSEKER QNCELFEQLGEYKFQNALLVR RMPCAEDYLSVVLNQLCVLHEK LVRPEVDVMCTAFHDNEETFLK RMPCAEDYLSVVLNQLCVLHEK LVRPEVDVMCTAFHDNEETFLKK |
| 2 | 459 | 1.56 | (P00738) Haptoglobin | 45.17 | 217 | 13 | DYAEVGR  QLVEIEK  GSFPWQAK ILGGHLDAK VGYVSGWGR VMPICLPSK VTSIQDWVQK DIAPTLTLYVGK SCAVAEYGVYVK YVMLPVADQDQCIR VSVNERVMPICLPSK VMPICLPSKDYAEVGR  SPVGVQPILNEHTFCAGMSK |
| 3 | 1016 | 3.2 | (P02647) Apolipoprotein A-  I (Apo-AI) | 30.75 | 486 | 20 | QKVEPLR  AELQEGAR LHELQEK AKPALEDLR LSPLGEEMR QKLHELQEK |

|  |  |  |  |  |  |  | QGLLPVLESFK  DLATVYVDVLK VQPYLDDFQK WQEEMELYR THLAPYSDELR LSPLGEEMRDR VQPYLDDFQKK VSFLSALEEYTK DYVSQFEGSALGK KWQEEMELYR VEPLRAELQEGAR LLDNWDSVTSTFSK DSGRDYVSQFEGSALGK LREQLGPVTQEFWDNLEK |
| --- | --- | --- | --- | --- | --- | --- | --- |
| 4 | 1011 | 1.76 | (P02753) Plasma retinol-  binding protein (RBP4) | 23.02 | 637 | 11 | QEELCLAR  FSGTWYAMAK DPNGLPPEAQK YWGVASFLQK QRQEELCLAR LIVHNGYCDGR LLNLDGTCADSYSFVFSR  LLNNWDVCADMVGTFTDTEDPAK GNDDHWIVDTDYDTYAVQYSCR  *DPEGLFLQDNIVAEFSVDETGQMSATA K*  *KDPEGLFLQDNIVAEFSVDETGQMSAT AK* |
| 5 | 262 | -3.76 | (P04217) Alpha-1B-  glycoprotein precursor  (Alpha-1-BN glycoprotein) | 54.3 | 900 | 16 | GVTFLLR  LLELTGPK CLAPLEGAR ATWSGAVLAGR  LETPDFQLFK  SGLSTGWTQLSK HQFLLTGDTQGR CEGPIPDVTFELLR NGVAQEPVHLDSPAIK LELHVDGPPPRPQLR VTLTCVAPLSGVDFQLR IFFHLNAVALGDGGHYTCR SLPAPWLSMAPVSWITPGLK TPGAAANLELIFVGPQHAGNYR SWVPHTFESELSDPVELLVAES  *LHDNQNGWSGDSAPVELILSDETLPAP EFSPEPESGR* |
| 6 | 1312 | 2.84 | (P02790) Hemopexin  precursor (Beta-1B- glycoprotein) | 51.64 | 239 | 14 | LHIMAGR  LWWLDLK RLWWLDLK DYFMPCPGR VWVYPPEKK FDPVRGEVPPR YYCFQGNQFLR GECQAEGVLFFQGDR ALPQPQNVTSLLGCTH LYLVQGTQVYVFLTK SGAQATWTELPWPHEK  LLQDEFPGIPSPLDAAVECHR EVGTPHGIILDSVDAAFICPGSSR  *SLGPNSCSANGPGLYLIHGPNLYCYSD VEK* |
| 7 | 1054 | 1.6 | (P02743) Serum amyloid P-  component precursor (SAP) | 25.37 | 179 | 7 | AYSDLSR  VGEYSLYIGR QGYFVEAQPK GYVIIKPLVWV IVLGQEQDSYGGK AYSLFSYNTQGR IVLGQEQDSYGGKFDR |

| 8 | 758 | 2.37 | (P01023) Alpha-2-  macroglobulin | 163.17 | 318 | 39 | YGAATFTR  FQVDNNNR  QGIPFFGQVR LPPNVVEESAR YDVENCLANK AIGYLNTGYQR VGFYESDVMGR VTAAPQSVCALR NALFCLESAWK HYDGSYSTFGER NQGNTWLTAFVLK LVHVEEPHTETVR ALLAYAFALAGNQDK IAQWQSFQLEGGLK DNSVHWERPQKPK TEHPFTVEEFVLPK SSSNEEVMFLTVQVK ALLAYAFALAGNQDKR AHTSFQISLSVSYTGSR LLIYAVLPTGDVIGDSAK QFSFPLSSEPFQGSYK VSVQLEASPAFLAVPVEK GHFSISIPVKSDIAPVAR FSGQLNSHGCFYQQVK GNRIAQWQSFQLEGGLK AFQPFFVELTMPYSVIR LLLQQVSLPELPGEYSMK VDLSFSPSQSLPASHAHLR AFQPFFVELTMPYSVIR MCPQLQQYEMHGPEGLR  AGAFCLSEDAGLGISSTASLR MCPQLQQYEMHGPEGLR LHTEAQIQEEGTVVELTGR QQNAQGGFSSTQDTVVALHALSK SLFTDLEAENDVLHCVAFAVPK NALFCLESAWKTAQEGDHGSHVYTK VVSMDENFHPLNELIPLVYIQDPK  *YDVENCLANKVDLSFSPSQSLPASHAH LR*  *SPCYGYQWVSEEHEEAHHTAYLVFSPS K* |
| --- | --- | --- | --- | --- | --- | --- | --- |
| 9 | 707 | 2.98 | (P00450) Ceruloplasmin | 122.13 | 460 | 36 | KGSLHANGR  QYTDSTFR  EYTDASFTNR EYTDASFTNRK GAYPLSIEPIGVR EVGPTNADPVCLAK QSEDSTFYLGER DIASGLIGPLIICK ALYLQYTDETFR VNKDDEEFIESNK DLYSGLIGPLIVCR RQSEDSTFYLGER QYTDSTFRVPVER KALYLQYTDETFR NNEGTYYSPNYNPQSR TTIEKPVWLGFLGPIIK TYYIAAVEVEWDYSPQR NLASRPYTFHSHGITYYK LISVDTEHSNIYLQNGPDR |

|  |  |  |  |  |  |  | MHSMNGFMYGNQPGLTMCK  KAEEEHLGILGPQLHADVGDK FNKNNEGTYYSPNYNPQSR KLISVDTEHSNIYLQNGPDR MYYSAVDPTKDIFTGLIGPMK HYYIGIIETTWDYASDHGEK MYSVNGYTFGSLPGLSMCAEDR MFTTAPDQVDKEDEDFQESNK GPEEEHLGILGPVIWAEVGDTIR SGAGTEDSACIPWAYYSTVDQVK HYYIGIIETTWDYASDHGEKK SVPPSASHVAPTETFTYEWTVPK GVYSSDVFDIFPGTYQTLEMFPR ERGPEEEHLGILGPVIWAEVGDTIR WYLFGMGNEVDVHAAFFHGQALTNK  *ADDKVYPGEQYTYMLLATEEQSPGEG DGNCVTR*  *NMATRPYSIHAHGVQTESSTVTPTLPG ETLTYVWK* |
| --- | --- | --- | --- | --- | --- | --- | --- |
| 10 | 214 | 1.86 | (P10909) Clusterin  precursor (Complement- associated protein SP-40) | 52.46 | 151 | 8 | VCRSGSGLVGR  EGDDDRTVCR ELDESLQVAER NPKFMETVAEK  ASSIIDELFQDR  EILSVDCSTNNPSQAK LFDSDPITVTVPVEVSR VTTVASHTSDSDVPSGVTEVVVK |
| 11 | 1553 | 5.49 | (P02750) Leucine-rich  alpha-2-glycoprotein (LRG) | 38.15 | 637 | 10 | GPLQLER  DCQVFR  VAAGAFQGLR ALGHLDLSGNR DLLLPQPDLR ENQLEVLEVSWLHGLK TLDLGENQLETLPPDLLR  NALTGLPPGLFQASATLDTLVLK DGFDISGNPWICDQNLSDLYR LQELHLSSNGLESLSPEFLRPVPQLR |
| 12 | 970 | 2.43 | (P02790) Hemopexin  precursor (Beta-1B- glycoprotein) | 52.38 | 207 | 9 | RLWWLDLK  DYFMPCPGR  DYFM*PCPGR NFPSPVDAAFR FDPVRGEVPPR YYCFQGNQFLR GECQAEGVLFFQGDR LLQDEFPGIPSPLDAAVECHR  EVGTPHGIILDSVDAAFICPGSSR |
| 13 | 322 | -1.4 | (P02774) Vitamin D  Binding protein | 54.52 | 199 | 3 | SCESNSPFPVHPGTAECCTK  EVVSLTEACCAEGADPDCYDTR HQPQEFPTYVEPTNDEICEAFR |
| 14 | 653 | 2.42 | (P01009) Alpha-1-  antitrypsin | 46.87 | 355 | 5 | GKWERPFEVK  ITPNLAEFAFSLYR FNKPFVFLMIEQNTK  ELDRDTVFALVNYIFFK LYHSEAFTVNFGDTEEAKK |
| 15 | 1394 | 2.52 | (P01024) Complement C3 | 188.56 | 275 | 8 | GYTQQLAFR  DFDFVPPVVR  KGYTQQLAFR AGDFLEANYM*NLQR SYTVAIAGYALAQMGR SYTVAIAGYALAQMGR ILLQGTPVAQMTEDAVDAER EGVQKEDIPPADLSDQVPDTESETR |
| 16 | 258 | 2.99 | (P02649) Apolipoprotein E  precursor | 36.24 | 234 | 6 | LAVYQAGAR  LGPLVEQGR LQAEAFQAR AKLEEQAQQIR |

|  |  |  |  |  |  |  | AATVGSLAGQPLQER  SELEEQLTPVAEETR |
| --- | --- | --- | --- | --- | --- | --- | --- |
| 17 | 319 | -1.63 | (P02768) Serum albumin  precursor | 69.32 | 663 | 26 | CCKADDK  YLYEIAR D  DNPNLPR FQNALLVR CASLQKFGER HPDYSVVLLLR AVMDDFAAFVEK RHPDYSVVLLLR CCAAADPHECYAK DVFLGMFLYEYAR KVPQVSTPTLVEVSR QNCELFEQLGEYK QEPERNECFLQHK HPYFYAPELLFFAK RPCFSALEVDETYVPK  NECFLQHKDDNPNLPR VFDEFKPLVEEPQNLIK VHTECCHGDLLECADDR EFNAETFTFHADICTLSEK MPCAEDYLSVVLNQLCVLHEK EFNAETFTFHADICTLSEKER VHTECCHGDLLECADDRADLAK LVRPEVDVMCTAFHDNEETFLK RMPCAEDYLSVVLNQLCVLHEK LVRPEVDVMCTAFHDNEETFLKK CCTESLVNRRPCFSALEVDETYVPK |
| 18 | 436 | 2.08 | (P00738) Haptoglobin  precursor | 45.17 | 248 | 12 | NYYKLR  NPANPVQR VMPICLPSK YQCKNYYK LPECEAVCGKPK TEGDGVYTLNDKK LRTEGDGVYTLNDK YVMLPVADQDQCIR LRTEGDGVYTLNNEK  AVGDKLPECEAVCGKPK LPECEADDGCPKPPEIAHGYVEHSVR  *AVGDKLPECEADDGCPKPPEIAHGYVE HSVR* |
| 19 | 441 | 4.42 | (P00738) Haptoglobin  precursor | 45.17 | 713 | 14 | DYAEVGR  GSFPWQAK  VGYVSGWGR VTSIQDWVQK DIAPTLTLYVGK TEGDGVYTLNDK SCAVAEYGVYVK YVMLPVADQDQCIR VSVNERVMPICLPSK VVLHPNYSQVDIGLIK VMPICLPSKDYAEVGR SPVGVQPILNEHTFCAGMSK  YVMLPVADQDQCIRHYEGSTVPEK  *YQEDTCYGDAGSAFAVHDLEEDTWY ATGILSFDK* |
| 20 | 1009 | 1.63 | (P10909) Clusterin  precursor (Complement- associated protein SP-40) | 52.46 | 179 | 8 | IDSLLENDR  RPHFFFPK QTCMKFYAR TLLSNLEEAKK  NPKFMETVAEK  ASSIIDELFQDR QQTHMLDVMQDHFSR EPQDTYHYLPFSLPHR |
| 21 | 261 | -2.02 | (P02768) Serum albumin  precursor | 69.3 | 674 | 23 | YLYEIAR  FQNALLVR QTALVELVK CASLQKFGER ECCEKPLLEK |

|  |  |  |  |  |  |  | HPDYSVVLLLR  AVMDDFAAFVEK RHPDYSVVLLLR CCAAADPHECYAK VPQVSTPTLVEVSR DVFLGMFLYEYAR YICENQDSISSKLK KVPQVSTPTLVEVSR QNCELFEQLGEYK QEPERNECFLQHK RPCFSALEVDETYVPK VPQVSTPTLVEVSRNLGK SLHTLFGDKLCTVATLR AAFTECCQAADKAACLLPK VFDEFKPLVEEPQNLIK EFNAETFTFHADICTLSEK MPCAEDYLSVVLNQLCVLHEK RMPCAEDYLSVVLNQLCVLHEK |
| --- | --- | --- | --- | --- | --- | --- | --- |

# Alterations in protein expression levels in MG I patients were measured using healthy subjects as controls

* Indicates the continuation of peptide sequence in the next line

**Table S5.** Master tables for all MALDI-TOF/TOF identified proteins in meningioma grade II

visualized in 2D-DIGE #

[Analysis Type: Combined (MS+MS/MS); Database: SwissProt; Taxonomy: Human]

| **Sl**  **No.** | **Mast**  **er**  **ID** | **Fold-**  **change** | **Name of protein with**  **UniProt accession number** | **MW**  **(kDa)** | **Protein**  **score** | **No. of**  **matched peptide s** | **Peptide sequence** |
| --- | --- | --- | --- | --- | --- | --- | --- |
| 1 | 636 | 2.06 | (P00738) Haptoglobin | 45.17 | 713 | 14 | DYAEVGR  GSFPWQAK VGYVSGWGR VTSIQDWVQK DIAPTLTLYVGK TEGDGVYTLNDK SCAVAEYGVYVK YVMLPVADQDQCIR VSVNERVMPICLPSK VVLHPNYSQVDIGLIK VMPICLPSKDYAEVR  SPVGVQPILNEHTFCAGMSK YVMLPVADQDQCIRHYEGSTVPK  *YQEDTCYGDAGSAFAVHDLEEDTWYA  TGILSFDK* |
| 2 | 641 | -1.50 | (P00751) Complement  factor B precursor (EC  3.4.21.47) | 85.4 | 717 | 26 | ISVIRPSK  STGSWSTLK VASYGVKPR DISEVVTPR EELLPAQDIK ALFVSEEEKK VKDISEVVTPR YGLVTYATYPK LPPTTTCQQQK EKLQDEDLGFL LEDSVTYHCSR QLNEINYEDHK DFHINLFQVLPWLK FIQVGVISWGVVDVCK EAGIPEFYDYDVALIK FLCTGGVSPYADPNTCR KEAGIPEFYDYDVALIK  SRFIQVGVISWGVVDVK DLEIEVVLFHPNYNINGK YGQTIRPICLPCTEGTTR  AIHCPRPHDFENGEYWPR  LPPTTTCQQQKEELLPAQDIK WSGQTAICDNGAGYCSNPGIPIGTR LLQEGQALEYVCPSGFYPYPVQTR EDYLDVYVFGVGPLVNQVNINALASK  *GHESCMGAVVSEYFVLTAAHCFTVDD KEHSIK* |
| 3 | 651 | -2.62 | (P02787) Serotransferrin  precursor (Transferrin) | 76.99 | 1160 | 42 | NPDPWAK  SCHTAVGR APNHAVVTR ASYLDCIR YLGEEYVK KASYLDCIR WCALSHHER DSGFQMNQLR SASDLTWDNK HSTIFENLANK EFQLFSSPHGK EGYYGYTGAR WCAVSEHEATK |

|  |  |  |  |  |  |  | KDSGFQMNQLR  DYELLCLDGTR KSASDLTWDNLK SVIPSDGPSVACVK MYLGYEYVTAIR SKEFQLFSSPHGK LKCDEWSVNSVGK CSTSSLLEACTFR DQYELLCLDNTR DLLFRDDTVCLAK FDEFFSEGCAPGSK TAGWNIPMGLLYNK KPVEEYANCHLAR EDPQTFYYAVAVVK KCSTSSLLEACTFR DCHLAQVPSHTVVAR FDEFFSEGCAPGSKK IECVSAETTEDCIAK EGTCPEAPTDECKPVK WCAVSEHEATKCQSFR ADRDQYELLCLDNTR NLNEKDYELLCLDGTR EDLIWELLNQAQEHFGK SDNCEDTPEAGYFAVAVVK SAGWNIPIGLLYCDLPEPR NLREGTCPEAPTDECKPVK KPVDEYKDCHLAQVPSHTVVAR  LCMGSGLNLCEPNNKEGYYGYTGAFR  *AIAANEADAVTLDAGLVYDAYLAPNN LKPVVAEFYGSK* |
| --- | --- | --- | --- | --- | --- | --- | --- |
| 4 | 1084 | 1.74 | (P00738) Haptoglobin | 45.17 | 306 | 9 | DYAEVGR  NYYKLR  GSFPWQAK VGYVSGWGR VMPICLPSK YQCKNYYK VTSIQDWVQK SCAVAEYGVYVK YVMLPVADQDQCIR |
| 5 | 697 | 1.65 | (P02790) Hemopexin (Beta-  1B-glycoprotein) | 51.64 | 596 | 21 | LHIMAGR  LWWLDLK GEVPPRYPR RLWWLDLK  DYFMPCPGR  VWVYPPEKK FDPVRGEVPPR GDKVWVYPPEK SWPAVGNCSSALR YYCFQGNQFLR EWFWDLATGTMK GECQAEGVLFFQGDR LYLVQGTQVYVFLTK SGAQATWTELPWPHEK SWPAVGNCSSALRWLGR LLQDEFPGIPSPLDAAVECHR  EVGTPHGIILDSVDAAFICPGSSR CSPHLVLSALTSDNHGATYAFSGTHYWR GECQAEGVLFFQGDREWFWDLATGTM  K  *DGWHSWPIAHQWPQGPSAVDAAFSWE EK*  *SLGPNSCSANGPGLYLIHGPNLYCYSDV  EK* |
| 6 | 682 | 1.82 | (P01876) Ig alpha-1 chain C  region | 37.63 | 491 | 14 | VAAEDWK  YLTWASR SAVQGPPER EKYLTWASR WLQGSQELPR TFTCTAAYPESK |

|  |  |  |  |  |  |  | DASGVTFTWTPSSGK  QEPSQGTTTFAVTSILR GDTFSCMVGHEALPLAFTQK TFTCTAAYPESKTPLTATLSK KGDTFSCMVGHEALPLAFTQK DLCGCYSVSSVLPGCAEPWNHGK  *NFPPSQDASGDLYTTSSQLTLPATQCLA  GK*  *SGNTFRPEVHLLPPPSEELALNELVTLT CLAR* |
| --- | --- | --- | --- | --- | --- | --- | --- |
| 7 | 612 | -3.16 | (P02768) Serum albumin | 69.3 | 1410 | 26 | YLYEIAR  LCTVATLR DDNPNLPR FQNALLVR NECFLQHK CCTESLVNR LVNEVTEFAK ECCEKPLLEK HPDYSVVLLLR AVMDDFAAFVEK AAFTECCQAADK RHPDYSVVLLLR CCAAADPHECYAK VPQVSTPTLVEVSR DVFLGMFLYEYAR KVPQVSTPTLVEVSR QNCELFEQLGEYK HPYFYAPELLFFAK RPCFSALEVDETYVPK  VHTECCHGDLLECADDR VFDEFKPLVEEPQNLIK EFNAETFTFHADICTLSEK ALVLIAFAQYLQQCPFEDHVK MPCAEDYLSVVLNQLCVLHEK LVRPEVDVMCTAFHDNEETFLK SHCIAEVENDEMPADLPSLAADFVESK |
| 8 | 861 | 3.83 | (P02647) Apolipoprotein A-  I (Apo-AI) | 30.75 | 486 | 20 | QKVEPLR  AELQEGAR LHELQEK AKPALEDLR  LSPLGEEMR  QKLHELQEK QGLLPVLESFK DLATVYVDVLK VQPYLDDFQK WQEEMELYR THLAPYSDELR LSPLGEEMRDR VQPYLDDFQKK VSFLSALEEYTK DYVSQFEGSALGK KWQEEMELYR VEPLRAELQEGAR LLDNWDSVTSTFSK DSGRDYVSQFEGSALGK LREQLGPVTQEFWDNLEK |
| 9 | 587 | -2.02 | (P02768) Serum albumin | 69.32 | 456 | 30 | CCKADDK  YLYEIAR LCTVATLR DDNPNLPR LKCASLQK FQNALLVR LVNEVTEFAK FKDLGEENFK HPDYSVVLLLR AVMDDFAAFVEK RHPDYSVVLLLR CCAAADPHECYAK DVFLGMFLYEYAR |

|  |  |  |  |  |  |  | KVPQVSTPTLVEVSR  QEPERNECFLQHK HPYFYAPELLFFAK RHPYFYAPELLFFAK HPYFYAPELLFFAKR RPCFSALEVDETYVPK VFDEFKPLVEEPQNLIK EFNAETFTFHADICTLSEK ALVLIAFAQYLQQCPFEDHVK TCVADESAENCDKSLHTLFGDK MPCAEDYLSVVLNQLCVLHEK EFNAETFTFHADICTLSEKER QNCELFEQLGEYKFQNALLVR RMPCAEDYLSVVLNQLCVLHEK LVRPEVDVMCTAFHDNEETFLK RMPCAEDYLSVVLNQLCVLHEK LVRPEVDVMCTAFHDNEETFLKK |
| --- | --- | --- | --- | --- | --- | --- | --- |
| 10 | 945 | -2.62 | (P02765) Alpha-2-HS-  glycoprotein | 39.29 | 495 | 10 | QYGFCK  FSVVYAK  HTLNQIDEVK CDSSPDSAEDVRK EHAVEGDCDFQLLK HTFMGVVSLGSPSGEVSHPR HTFMGVVSLGSPSGEVSHPRK  AQLVPLPPSTYVEFTVSGTDCVAK  *QPNCDDPETEEAALVAIDYINQNLPWG YK*  *VWPQQPSGELFEIEIDTLETTCHVLDPTP VAR* |

# Alterations in protein expression levels in MG II patients were measured using healthy subjects as controls

* Indicates the continuation of peptide sequence in the next line

**Table S6.** Complete details of protein identification and quantitative iTRAQ data for different grades of meningiomas.

| **Group Num** | **Num Spectra** | **Num Peps Unique** | **Score Unique** | **% Coverage** | **log2_iTRAQ_115_114_median** | **Fold-change (HC vs. MG1)** | **log2_iTRAQ_116_114_median** | **Fold-change (HC vs. MG2)** | **log2_iTRAQ_117_114_median** | **Fold-change (HC vs. MG3)** | **Accession_number** | **Protein_name** | **Peptide sequence** |
| --- | --- | --- | --- | --- | --- | --- | --- | --- | --- | --- | --- | --- | --- |
| **1** | 320 | 100 | 1797.28 | 32.7 | 0.843 | 1.79 | 1.895 | 3.72 | 2.718 | 6.58 | P04114.2 | Apolipoprotein B-100 | AALTELSLGSAYQAMILGVDSK ADSVVDLLSYNVQGSGETTYDHK AEPLAFTFSHDYK AHLDIAGSLEGHLR ALVEQGFTVPEIK AQNLYQELLTQEGQASFQGLK ATFQTPDFIVPLTDLR ATGVLYDYVNK ATVAVYLESLQDTK AVSMPSFSILGSDVR DAVEKPQEFTIVAFVK DFSLWEK DKAQNLYQELLTQEGQASFQGLK DKDQEVLLQTFLDDASPGDKR DLKVEDIPLAR DNVFDGLVR EELCTMFIR EFNLQNMGLPDFHIPENLFLK EFQVPTFTIPK ESQLPTVMDFR EVGTVLSQVYSK EVYGFNPEGK EYSGTIASEANTYLNSK FDHTNSLNIAGLSLDFSSK FPEVDVLTK FSDEGTHESQISFTIEGPLTSFGLSNK GFEPTLEALFGK GIISALLVPPETEEAK GMALFGEGK HSITNPLAVLCEFISQSIK IADFELPTIIVPEQTIEIPSIK IAELSATAQEIIK IDDIWNLEVK IEFEWNTGTNVDTK IEGNLIFDPNNYLPK IEIPLPFGGK IGQDGISTSATTNLK ILGEELGFASLHDLQLLGK INCKVELEVPQLCSFILK ITENDIQIALDDAK ITLPDFR IVQILPWEQNEQVK KMTSNFPVDLSDYPK KYTYNYEAESSSGVPGTADSR LDFSSQADLR LEIQSQVDSQHVGHSVLTAK LELELRPTGEIEQYSVSATYELQR LELELRPTGEIEQYSVSATYELQREDR LIDVISMYR LLLQMDSSATAYGSTVSK LNDLNSVLVMPTFHVPFTDLQVPSCK LNTDIAGLASAIDMSTNYNSDSLHFSNVFR LPQQANDYLNSFNWER LPYTIITTPPLKDFSLWEK LSLESLTSYFSIESSTK LSLESLTSYFSIESSTKGDVK LSLPDFK LSNDMMGSYAEMK LTLDIQNK LVGFIDDAVK MGLAFESTK MTSNFPVDLSDYPK MYQMDIQQELQR NFVASHIANILNSEELDIQDLK NFVASHIANILNSEELDIQDLKK NIQEYLSILTDPDGK NLQDLLQFIFQLIEDNIK NLQNNAEWVYQGAIR NLTDFAEQYSIQDWAK NSLFFSAQPFEITASTNNEGNLK QTIIVVLENVQR QVFLYPEKDEPTYILNIKR SGSSTASWIQNVDTK SLWDFLK SVSDGIAALDLNAVANK SVSLPSLDPASAK TEHGSEMLFFGNAIEGK TEVIPPLIENR TFQIPGYTVPVVNVEVSPFTIEMSAFGYVFPK TIHDLHLFIENIDFNK TILGTMPAFEVSLQALQK TLADLTLLDSPIK TLADLTLLDSPIKVPLLLSEPINIIDALEMR TLQGIPQMIGEVIR TSSFALNLPTLPEVK VEDIPLAR VELEVPQLCSFILK VHANPLLIDVVTYLVALIPEPSAQQLREIFNMAR VIGNMGQTMEQLTPELK VLLDQLGTTISFER VNWEEEAASGLLTSLK VNWEEEAASGLLTSLKDNVPK VPLLLSEPINIIDALEMR VPQTDMTFR VPSYTLILPSLELPVLHVPR VSALLTPAEQTGTWK YDKNQDVHSINLPFFETLQEYFER YENYELTLK YSQPEDSLIPFFEITVPESQLTVSQFTLPK YTYNYEAESSSGVPGTADSR |
| **2** | 393 | 75 | 1344.36 | 54.9 | 0.316 | 1.24 | 1.323 | 2.50 | 1.591 | 3.01 | P01024.2 | Complement C3 | ACEPGVDYVYK ACEPGVDYVYKTR ADIGCTPGSGKDYAGVFSDAGLTFTSSSGQQTAQR AEDLVGKSLYVSATVILHSGSDMVQAER AGDFLEANYMNLQR APSTWLTAYVVKVFSLAVNLIAIDSQVLCGAVK AYYENSPQQVFSTEFEVK DAPDHQELNLDVSLQLPSR DFDFVPPVVR DICEEQVNSLPGSITK DSCVGSLVVK DSITTWEILAVSMSDK DSITTWEILAVSMSDKK DYAGVFSDAGLTFTSSSGQQTAQR ENEGFTVTAEGK EPGQDLVVLPLSITTDFIPSFR EVVADSVWVDVK EVVADSVWVDVKDSCVGSLVVK EYVLPSFEVIVEPTEK FISLGEACK FVTVQATFGTQVVEK FYYIYNEK GLEVTITAR ILLQGTPVAQMTEDAVDAER IPIEDGSGEVVLSR ISLPESLK KQELSEAEQATR KVEGTAFVIFGIQDGEQR KVFLDCCNYITELR KVLLDGVQNPRAEDLVGK LDKACEPGVDYVYK LDKACEPGVDYVYKTR LESEETMVLEAHDAQGDVPVTVTVHDFPGK LESEETMVLEAHDAQGDVPVTVTVHDFPGKK NNNEKDMALTAFVLISLQEAK NTLIIYLDK NTMILEICTR QDSLSSQNQLGVLPLSWDIPELVNMGQWK QGALELIK QKPDGVFQEDAPVIHQEMIGGLR QLYNVEATSYALLALLQLKDFDFVPPVVR QVREPGQDLVVLPLSITTDFIPSFR RIPIEDGSGEVVLSR SDDKVTLEERLDK SEETKENEGFTVTAEGK SEETKENEGFTVTAEGKGQGTLSVVTMYHAK SEFPESWLWNVEDLK SEFPESWLWNVEDLKEPPK SGIPIVTSPYQIHFTK SGQSEDRQPVPGQQMTLK SGQSEDRQPVPGQQMTLKIEGDHGAR SLYVSATVILHSGSDMVQAER SNLDEDIIAEENIVSR SSLSVPYVIVPLK SSLSVPYVIVPLKTGLQEVEVK TELRPGETLNVNFLLR TGLQEVEVKAAVYHHFISDGVR TKKQELSEAEQATR TVMVNIENPEGIPVK VELLHNPAFCSLATTK VFLDCCNYITELR VFLDCCNYITELRR VHQYFNVELIQPGAVK VLLDGVQNPR VLLDGVQNPRAEDLVGK VPVAVQGEDTVQSLTQGDGVAK VQLSNDFDEYIMAIEQTIK VQLSNDFDEYImAIEQTIK VQLSNDFDEYIMAIEQTIKSGSDEVQVGQQR VSHSEDDCLAFK VTIKPAPETEK VVLVAVDK VYAYYNLEESCTR WLILEK YFKPGMPFDLMVFVTNPDGSPAYR YISKYELDK |
| **3** | 317 | 52 | 954.16 | 47.8 | 0.54 | 1.45 | 1.788 | 3.45 | 1.641 | 3.12 | P01023.3 | Alpha-2-macroglobulin | AFQPFFVELTMPYSVIR AFQPFFVELTmPYSVIR AGAFCLSEDAGLGISSTASLR ALLAYAFALAGNQDK AVDQSVLLMKPDAELSASSVYNLLPEK AYIFIDEAHITQALIWLSQR DMYSFLEDMGLK DTVIKPLLVEPEGLEK EEFPFALGVQTLPQTCDEPK ETTFNSLLCPSGGEVSEELSLK ETTFNSLLCPSGGEVSEELSLKLPPNVVEESAR FEVQVTVPK GGVEDEVTLSAYITIALLEIPLTVTHPVVR HNVYINGITYTPVSSTNEK HNVYINGITYTPVSSTNEKDMYSFLEDMGLK HYDGSYSTFGER IAQWQSFQLEGGLK IAQWQSFQLEGGLKQFSFPLSSEPFQGSYK KDTVIKPLLVEPEGLEK KYSDASDCHGEDSQAFCEK LHTEAQIQEEGTVVELTGR LLIYAVLPTGDVIGDSAK LLLQQVSLPELPGEYSMK LPPNVVEESAR LVHVEEPHTETVR MCPQLQQYEMHGPEGLR NALFCLESAWK NEDSLVFVQTDK QFSFPLSSEPFQGSYK QGIPFFGQVR QQNAQGGFSSTQDTVVALHALSK QTVSWAVTPK SASNMAIVDVK SGGRTEHPFTVEEFVLPK SGGRTEHPFTVEEFVLPKFEVQVTVPK SLFTDLEAENDVLHCVAFAVPK SPCYGYQWVSEEHEEAHHTAYLVFSPSK SSSNEEVMFLTVQVK TEHPFTVEEFVLPK TEVSSNHVLIYLDK TTVMVKNEDSLVFVQTDK VDLSFSPSQSLPASHAHLR VGFYESDVMGR VSVQLEASPAFLAVPVEK VTAAPQSVCALR VTGEGCVYLQTSLK VVSMDENFHPLNELIPLVYIQDPK VVSMDENFHPLNELIPLVYIQDPKGNR VYDYYETDEFAIAEYNAPCSK YDVENCLANK YDVENCLANKVDLSFSPSQSLPASHAHLR YNILPEKEEFPFALGVQTLPQTCDEPK YSDASDCHGEDSQAFCEK |
| **4** | 382 | 46 | 887.4 | 59.1 | -0.573 | 0.67 | 0.095 | 1.07 | 0.332 | 1.26 | P02787.3 | Serotransferrin | ADRDQYELLCLDNTR ASYLDCIR CDEWSVNSVGK CLKDGAGDVAFVK CLVEKGDVAFVK CSTSSLLEACTFR DGAGDVAFVK DLLFK DLLFKDSAHGFLK DLLFRDDTVCLAK DSGFQMNQLR EDLIWELLNQAQEHFGK EDLIWELLNQAQEHFGKDK EDPQTFYYAVAVVK EDPQTFYYAVAVVKK EFQLFSSPHGK EGTCPEAPTDECKPVK EGTCPEAPTDECKPVKWCALSHHER EGYYGYTGAFR FDEFFSEGCAPGSK FDEFFSEGCAPGSKK GDVAFVK HSTIFENLANK HSTIFENLANKADRDQYELLCLDNTR IECVSAETTEDCIAK INHCRFDEFFSEGCAPGSK KPVDEYKDCHLAQVPSHTVVAR KPVEEYANCHLAR KSASDLTWDNLK LCMGSGLNLCEPNNK LCMGSGLNLCEPNNKEGYYGYTGAFR LKCDEWSVNSVGK LKCDEWSVNSVGKIECVSAETTEDCIAK MYLGYEYVTAIR mYLGYEYVTAIR NLNEKDYELLCLDGTR NLNEKDYELLCLDGTRKPVEEYANCHLAR SAGWNIPIGLLYCDLPEPR SASDLTWDNLK SASDLTWDNLKGK SETKDLLFRDDTVCLAK SMGGKEDLIWELLNQAQEHFGK SmGGKEDLIWELLNQAQEHFGK SMGGKEDLIWELLNQAQEHFGKDK SmGGKEDLIWELLNQAQEHFGKDK SVIPSDGPSVACVK TAGWNIPMGLLYNK WCAVSEHEATK YLGEEYVK |
| **5** | 186 | 42 | 774.94 | 31 | 0.245 | 1.19 | 0.67 | 1.59 | 0.727 | 1.66 | P0C0L5.1 | Complement C4-B | AEFQDALEK AEFQDALEKLNMGITDLQGLR AEMADQASAWLTR DFALLSLQVPLK DFALLSLQVPLKDAK DSSTWLTAFVLK ECVGFEAVQEVPVGLVQPASATLYDYYNPER ECVGFEAVQEVPVGLVQPASATLYDYYNPERR EELVYELNPLDHR EMSGSPASGIPVK EPFLSCCQFAESLR FGLLDEDGK FGLLDEDGKK FGLLDEDGKKTFFR GLEEELQFSLGSK GPEVQLVAHSPWLK GSFEFPVGDAVSK ITPGKPYILTVPGHLDEMQLDIQAR KEVYMPSSIFQDDFVIPDISEPGTWK KKEVYMPSSIFQDDFVIPDISEPGTWK LELSVDGAK LHLETDSLALVALGALDTALYAAGSK LLATLCSAEVCQCAEGK LNMGITDLQGLR LQETSNWLLSQQQADGSFQDLSPVIHR LTVAAPPSGGPGFLSIERPDSRPPR SFFPENWLWR STQDTVIALDALSAYWIASHTTEER TEQWSTLPPETK VDFTLSSER VDFTLSSERDFALLSLQVPLKDAK VDVQAGACEGK VEYGFQVK VGDTLNLNLR VLQIEKEGAIHREELVYELNPLDHR VLSLAQEQVGGSPEK VQQPDCREPFLSCCQFAESLR VQQPDCREPFLSCCQFAESLRK VTASDPLDTLGSEGALSPGGVASLLR YLDKTEQWSTLPPETK YVLPNFEVK YVSHFETEGPHVLLYFDSVPTSR |
| **6** | 108 | 30 | 615.64 | 67.4 | -0.659 | 0.63 | 0.075 | 1.05 | -0.723 | 0.61 | P02768.2 | Serum albumin | ADDKETCFAEEGKK AEFAEVSKLVTDLTK ALVLIAFAQYLQQCPFEDHVK AVMDDFAAFVEK DVFLGMFLYEYAR EFNAETFTFHADICTLSEK EFNAETFTFHADICTLSEKER KVPQVSTPTLVEVSR LKECCEKPLLEK LVAASQAALGL LVNEVTEFAK LVRPEVDVMCTAFHDNEETFLK LVRPEVDVMCTAFHDNEETFLKK LVTDLTK MPCAEDYLSVVLNQLCVLHEK MPCAEDYLSVVLNQLCVLHEKTPVSDR NYAEAKDVFLGMFLYEYAR QNCELFEQLGEYK QTALVELVK RMPCAEDYLSVVLNQLCVLHEK RMPCAEDYLSVVLNQLCVLHEKTPVSDR RPCFSALEVDETYVPK SHCIAEVENDEMPADLPSLAADFVESK SHCIAEVENDEMPADLPSLAADFVESKDVCK VFDEFKPLVEEPQNLIK VHTECCHGDLLECADDR VHTECCHGDLLECADDRADLAK VPQVSTPTLVEVSR YICENQDSISSK YLYEIAR |
| **7** | 197 | 29 | 539.32 | 70.4 | 1.19 | 2.28 | 2.825 | 7.09 | 3.729 | 13.26 | P02647.1 | Apolipoprotein A-I | AKPALEDLR AKVQPYLDDFQK ATEHLSTLSEK ATEHLSTLSEKAKPALEDLR DLATVYVDVLK DLATVYVDVLKDSGRDYVSQFEGSALGK DLEEVK DSGRDYVSQFEGSALGK DYVSQFEGSALGK EQLGPVTQEFWDNLEK EQLGPVTQEFWDNLEKETEGLR KWQEEMELYR LLDNWDSVTSTFSK LLDNWDSVTSTFSKLR LREQLGPVTQEFWDNLEK LREQLGPVTQEFWDNLEKETEGLR LSPLGEEMR LSPLGEEMRDR QGLLPVLESFK QGLLPVLESFKVSFLSALEEYTK QKVEPLRAELQEGAR THLAPYSDELR VEPLRAELQEGAR VKDLATVYVDVLK VKDLATVYVDVLKDSGR VQPYLDDFQK VQPYLDDFQKKWQEEMELYR VSFLSALEEYTK WQEEMELYR |
| **8** | 152 | 27 | 564.51 | 37.8 | 0.048 | 1.03 | 1.028 | 2.04 | 0.52 | 1.43 | P00450.1 | Ceruloplasmin | ADDKVYPGEQYTYMLLATEEQSPGEGDGNCVTR AEEEHLGILGPQLHADVGDK AGLQAFFQVQECNK ALYLQYTDETFR DIASGLIGPLIICK DIFTGLIGPMK DLYSGLIGPLIVCR ERGPEEEHLGILGPVIWAEVGDTIR EVGPTNADPVCLAK GAYPLSIEPIGVR GPEEEHLGILGPVIWAEVGDTIR GVYSSDVFDIFPGTYQTLEMFPR HYYIAAEEIIWNYAPSGIDIFTK HYYIGIIETTWDYASDHGEKK KAEEEHLGILGPQLHADVGDK KAEEEHLGILGPQLHADVGDKVK KERGPEEEHLGILGPVIWAEVGDTIR MFTTAPDQVDKEDEDFQESNK MYYSAVDPTK MYYSAVDPTKDIFTGLIGPMK NNEGTYYSPNYNPQSR QKDVDKEFYLFPTVFDENESLLLEDNIR SGAGTEDSACIPWAYYSTVDQVK SVPPSASHVAPTETFTYEWTVPK TYCSEPEKVDKDNEDFQESNR VNKDDEEFIESNK VYPGEQYTYMLLATEEQSPGEGDGNCVTR |
| **9** | 153 | 27 | 485.96 | 32.8 | -0.147 | 0.90 | 0.2 | 1.15 | -0.034 | 0.98 | P08603.4 | Complement factor H | AGEQVTYTCATYYK AVYTCNEGYQLLGEINYR CFEGFGIDGPAIAK CTLKPCDYPDIK DGEKVSVLCQENYLIQEGEEITCKDGR DGWSAQPTCIK DTSCVNPPTVQNAYIVSR EIMENYNIALR EKTKEEYGHSEVVEYYCNPR GDAVCTESGWRPLPSCEEK IDVHLVPDR IVSSAMEPDREYHFGQAVR LGYVTADGETSGSITCGK LSYTCEGGFR NTEILTGSWSDQTYPEGTQAIYK RPCGHPGDTPFGTFTLTGGNVFEYGVK SCDIPVFMNAR SIDVACHPGYALPK SITCIHGVWTQLPQCVAIDK SSNLIILEEHLK TDCLSLPSFENAIPMGEK TGESVEFVCK TKEEYGHSEVVEYYCNPR VSVLCQENYLIQEGEEITCK VSVLCQENYLIQEGEEITCKDGR WQSIPLCVEK WSSPPQCEGLPCK |
| **10** | 126 | 23 | 392.79 | 53.1 | 0.761 | 1.69 | 2.641 | 6.24 | 2.874 | 7.33 | P01009.3 | Alpha-1-antitrypsin | AVLTIDEK AVLTIDEKGTEAAGAMFLEAIPMSIPPEVK DTEEEDFHVDQVTTVK DTEEEDFHVDQVTTVKVPMMK ELDRDTVFALVNYIFFK FLEDVK GTEAAGAMFLEAIPMSIPPEVK ITPNLAEFAFSLYR IVDLVK IVDLVKELDRDTVFALVNYIFFK KLYHSEAFTVNFGDTEEAK KLYHSEAFTVNFGDTEEAKK LGMFNIQHCK LQHLENELTHDIITK LSITGTYDLK LVDKFLEDVK LVDKFLEDVKK LYHSEAFTVNFGDTEEAK QINDYVEK SPLFMGK SVLGQLGITK TDTSHHDQDHPTFNKITPNLAEFAFSLYR WERPFEVK |
| **11** | 83 | 22 | 400.36 | 18.2 | -0.143 | 0.91 | 0.507 | 1.42 | 1.168 | 2.25 | P02751.4 | Fibronectin | DDKESVPISDTIIPAVPPPTDLR DLEVVAATPTSLLISWDAPAVTVR DLQFVEVTDVK EESPLLIGQQSTVSDVPR EINLAPDSSSVVVSGLMVATK EYLGAICSCTCFGGQR GEWTCIAYSQLR GFNCESKPEAEETCFDK ITYGETGGNSPVQEFTVPGSK NLQPASEYTVSLVAIK NTFAEVTGLSPGVTYYFK QDGHLWCSTTSNYEQDQK RPGGEPSPEGTTGQSYNQYSQR SSPVVIDASTAIDAPSNLR SYTITGLQPGTDYK TDELPQLVTLPHPNLHGPEILDVPSTVQK TNTNVNCPIECFMPLDVQADREDSRE VDVIPVNLPGEHGQR VTIMWTPPESAVTGYR VTWAPPPSIDLTNFLVR VVTPLSPPTNLHLEANPDTGVLTVSWER WCGTTQNYDADQK |
| **12** | 182 | 22 | 389.03 | 48.2 | -0.28 | 1.67 | 0.133 | 2.10 | -0.212 | 1.87 | P00738.1 | Haptoglobin | AVGDKLPECEADDGCPKPPEIAHGYVEHSVR AVGDKLPECEAVCGKPK DIAPTLTLYVGK DYAEVGR GSFPWQAK LPECEADDGCPKPPEIAHGYVEHSVR LPECEAVCGKPK LRTEGDGVYTLNDK LRTEGDGVYTLNDKK LRTEGDGVYTLNNEK LRTEGDGVYTLNNEKQWINK QLVEIEK SCAVAEYGVYVK SPVGVQPILNEHTFCAGMSK TEGDGVYTLNDKKQWINK TEGDGVYTLNNEK TEGDGVYTLNNEKQWINK VGYVSGWGR VMPICLPSK VMPICLPSKDYAEVGR VTSIQDWVQK YVMLPVADQDQCIR |
| **13** | 65 | 22 | 382.45 | 50.6 | -0.237 | 0.85 | 0.264 | 1.20 | -0.357 | 0.78 | P02774.1 | Vitamin D-binding protein | EDFTSLSLVLYSR EFSHLGKEDFTSLSLVLYSR ELPEHTVK ELSSFIDK ELSSFIDKGQELCADYSENTFTEYK ELSSFIDKGQELCADYSENTFTEYKK EYANQFMWEYSTNYGQAPLSLLVSYTK GQELCADYSENTFTEYK HQPQEFPTYVEPTNDEICEAFR HQPQEFPTYVEPTNDEICEAFRK HQPQEFPTYVEPTNDEICEAFRKDPK KELSSFIDKGQELCADYSENTFTEYK LAQKVPTADLEDVLPLAEDITNILSK SCESNSPFPVHPGTAECCTK SLGECCDVEDSTTCFNAK TAMDVFVCTYFMPAAQLPELPDVELPTNK THLPEVFLSK VCSQYAAYGEK VLEPTLK VMDKYTFELSR VPTADLEDVLPLAEDITNILSK YTFELSR |
| **14** | 95 | 22 | 372.13 | 51.2 | -0.112 | 0.93 | 0.742 | 1.67 | 1.521 | 2.87 | P01008.1 | Antithrombin-III | ADGESCSASMMYQEGK AFLEVNEEGSEAAASTAVVIAGR DDLYVSDAFHK DIPMNPMCIYR ELFYKADGESCSASMMYQEGK ELTPEVLQEWLDELEEMMLVVHMPR EQLQDMGLVDLFSPEK EVPLNTIIFMGR FRIEDGFSLKEQLQDMGLVDLFSPEK GDDITMVLILPKPEK IEDGFSLK ITDVIPSEAINELTVLVLVNTIYFK LPGIVAEGRDDLYVSDAFHK LQPLDFK LQPLDFKENAEQSR NDNDNIFLSPLSISTAFAMTK RVAEGTQVLELPFKGDDITMVLILPKPEK SKLPGIVAEGRDDLYVSDAFHK VAEGTQVLELPFK VAEGTQVLELPFKGDDITMVLILPKPEK VEKELTPEVLQEWLDELEEMMLVVHMPR VWELSK |
| **15** | 64 | 21 | 368.56 | 43 | -0.131 | 0.91 | 0.17 | 1.13 | 0.262 | 1.20 | P00734.2 | Prothrombin | DKLAACLEGNCAEGLGTNYR ELLESYIDGR ENLDRDIALMK HQDFNSAVQLVENFCR ISMLEK ITDNMFCAGYKPDEGK ITDNMFCAGYKPDEGKR IVEGSDAEIGMSPWQVMLFR KPVAFSDYIHPVCLPDRETAASLLQAGYK KSPQELLCGASLISDR LAACLEGNCAEGLGTNYR NPDSSTTGPWCYTTDPTVR RQECSIPVCGQDQVTVAMTPR SEGSSVNLSPPLEQCVPDR SEGSSVNLSPPLEQCVPDRGQQYQGR SGIECQLWR SPQELLCGASLISDR TATSEYQTFFNPR TFGSGEADCGLRPLFEK WYQMGIVSWGEGCDR WYQMGIVSWGEGCDRDGK |
| **16** | 161 | 19 | 365.85 | 46.7 | 0.203 | 1.15 | 1.627 | 3.09 | 1.794 | 3.47 | P02790.2 | Hemopexin | DGWHSWPIAHQWPQGPSAVDAAFSWEEK DVRDYFMPCPGR DYFMPCPGR EVGTPHGIILDSVDAAFICPGSSR EWFWDLATGTMK FDPVRGEVPPR GECQAEGVLFFQGDR GECQAEGVLFFQGDREWFWDLATGTMK GECQAEGVLFFQGDREWFWDLATGTMKER GEFVWK LEKEVGTPHGIILDSVDAAFICPGSSR LLQDEFPGIPSPLDAAVECHR LWWLDLK NFPSPVDAAFR RLEKEVGTPHGIILDSVDAAFICPGSSR SGAQATWTELPWPHEK SGAQATWTELPWPHEKVDGALCMEK VDGALCMEK VWVYPPEK |
| **17** | 71 | 19 | 336.5 | 39.8 | -0.285 | 0.82 | 0.728 | 1.66 | 0.892 | 1.86 | P00747.2 | Plasminogen | ATTVTGTPCQDWAAQEPHR DVVLFEK EAQLPVIENK FGMHFCGGTLISPEWVLTAAHCLEK FSPATHPSEGLEENYCR FVTWIEGVMR KLYDYCDVPQCAAPSFDCGKPQVEPK LFLEPTR LSSPAVITDK NPDADKGPWCFTTDPSVR NPDGDVGGPWCYTTNPR NPDNDPQGPWCYTTDPEKR TECFITGWGETQGTFGAGLLK TMSGLECQAWDSQSPHAHGYIPSK TPENYPNAGLTMNYCR VILGAHQEVNLEPHVQEIEVSR VIPACLPSPNYVVADR VQSTELCAGHLAGGTDSCQGDSGGPLVCFEK WELCDIPR |
| **18** | 72 | 17 | 320.84 | 31.1 | 0.759 | 1.69 | 1.643 | 3.12 | 1.909 | 3.76 | Q14624.4 | Inter-alpha-trypsin inhibitor heavy chain H4 | ATTVTGTPCQDWAAQEPHR DVVLFEK EAQLPVIENK FGMHFCGGTLISPEWVLTAAHCLEK FSPATHPSEGLEENYCR FVTWIEGVMR KLYDYCDVPQCAAPSFDCGKPQVEPK LFLEPTR LSSPAVITDK NPDADKGPWCFTTDPSVR NPDGDVGGPWCYTTNPR NPDNDPQGPWCYTTDPEKR TECFITGWGETQGTFGAGLLK TMSGLECQAWDSQSPHAHGYIPSK TPENYPNAGLTMNYCR VILGAHQEVNLEPHVQEIEVSR VIPACLPSPNYVVADR VQSTELCAGHLAGGTDSCQGDSGGPLVCFEK WELCDIPR |
| **19** | 53 | 17 | 274.73 | 36.4 | 0.075 | 1.05 | 0.979 | 1.97 | 1.42 | 2.68 | P01011.2 | Alpha-1-antichymotrypsin | ADLSGITGAR AKWEMPFDPQDTHQSR AVLDVFEEGTEASAATAVK AVLDVFEEGTEASAATAVKITLLSALVETR DEELSCTVVELK EIGELYLPK EQLSLLDR EQLSLLDRFTEDAK GTHVDLGLASANVDFAFSLYK ITLLSALVETR LINDYVK LYGSEAFATDFQDSAAAK LYGSEAFATDFQDSAAAKK MEEVEAMLLPETLK MEEVEAMLLPETLKR RLYGSEAFATDFQDSAAAK WEMPFDPQDTHQSR |
| **20** | 45 | 16 | 254.97 | 45.4 | -0.095 | 0.94 | 0.686 | 1.61 | 1.318 | 2.49 | P06727.3 | Apolipoprotein A-IV | ALVQQMEQLR ENADSLQASLRPHADELK IDQNVEELK IDQTVEELR LAPLAEDVR LEPYADQLR LGEVNTYAGDLQK LKEEIGKELEELR LTPYADEFK LTPYADEFKVK LVPFATELHER RVEPYGENFNKALVQQMEQLR SELTQQLNALFQDK SLAELGGHLDQQVEEFR SLAELGGHLDQQVEEFRR SLAPYAQDTQEKLNHQLEGLTFQMK |
| **21** | 53 | 15 | 250.09 | 36.3 | -0.547 | 0.68 | -0.227 | 0.85 | -0.725 | 0.60 | P04003.2 | C4b-binding protein alpha chain | CEWETPEGCEQVLTGK EDVYVVGTVLR FSAICQGDGTWSPR GVGWSHPLPQCEIVK KPDVSHGEMVSGFGPIYNYKDTIVFK LMQCLPNPEDVK LSLEIEQLELQR MALEVYK MALEVYKLSLEIEQLELQR QSSSYSFFKEEIIYECDK SHSTQTLTCNSDGEWVYNTFCIYK SRPANHCVYFYGDEISFSCHETSR TPSCGDICNFPPK TWYPEVPKCEWETPEGCEQVLTGKR WTPYQGCEALCCPEPK |
| **22** | 23 | 14 | 241.85 | 14 | 1.381 | 2.60 | 2.044 | 4.12 | 2.917 | 7.55 | P01031.4 | Complement C5 | AFTECCVVASQLR ALVEGVDQLFTDYQIK DGHVILQLNSIPSSDFLCVR DVFLEMNIPYSVVR FSDASYQSINIPVTQNMVPSSR IDTQDIEASHYR IPLDLVPK LSMDIDVSYK MSAVEGICTSESPVIDHQGTK MVETTAYALLTSLNLK QLPGGQNPVSYVYLEVVSK SYFPESWLWEVHLVPR TSTSEEVCSFYLK VSITSITVENVFVK |
| **23** | 179 | 14 | 259.83 | 56.6 | 0.125 | 1.09 | 0.165 | 1.12 | -0.868 | 0.55 | P01876.2 | Ig alpha-1 chain C region | DASGVTFTWTPSSGK DLCGCYSVSSVLPGCAEPWNHGK GDTFSCMVGHEALPLAFTQK KGDTFSCMVGHEALPLAFTQK LAGKPTHVNVSVVMAEVDGTCY NFPPSQDASGDLYTTSSQLTLPATQCLAGK QEPSQGTTTFAVTSILR SGNTFRPEVHLLPPPSEELALNELVTLTCLAR TFTCTAAYPESK TPLTATLSK VAAEDWK VAAEDWKKGDTFSCMVGHEALPLAFTQK WLQGSQELPR WLQGSQELPREK |
| **24** | 41 | 13 | 246.37 | 25.9 | -0.507 | 0.70 | -1.449 | 0.37 | -0.932 | 0.52 | P00751.2 | Complement factor B | ALFVSEEEK DAQYAPGYDKVKDISEVVTPR EAGIPEFYDYDVALIK EELLPAQDIK EKLQDEDLGFL FLCTGGVSPYADPNTCR HVIILMTDGLHNMGGDPITVIDEIR KEAGIPEFYDYDVALIK LLQEGQALEYVCPSGFYPYPVQTR QLNEINYEDHKLK VKDISEVVTPR VSEADSSNADWVTK WSGQTAICDNGAGYCSNPGIPIGTR |
| **25** | 35 | 13 | 236.8 | 44.9 | 0.472 | 1.39 | 1.611 | 3.05 | 1.783 | 3.44 | P25311.2 | Zinc-alpha-2-glycoprotein | AGEVQEPELR AYLEEECPATLR DYIEFNK EDIFMETLK EIPAWVPFDPAAQITK HVEDVPAFQALGSLNDLQFFR NILDRQDPPSVVVTSHQAPGEK QDPPSVVVTSHQAPGEK QKWEAEPVYVQR QVEGMEDWK QVEGMEDWKQDSQLQK WEAEPVYVQR YYYDGKDYIEFNK |
| **26** | 55 | 12 | 212.34 | 23.8 | -0.245 | 0.84 | -0.446 | 0.73 | 0.278 | 1.21 | P19823.2 | Inter-alpha-trypsin inhibitor heavy chain H2 | AEDHFSVIDFNQNIR ETAVDGELVVLYDVKREEK FLHVPDTFEGHFDGVPVISK HLEVDVWVIEPQGLR IQPSGGTNINEALLR MLADAPPQDPSCCSGALYYGSK NVQFNYPHTSVTDVTQNNFHNYFGGSEIVVAGK SILQMSLDHHIVTPLTSLVIENEAGDER SLPGESEEMMEEVDQVTLYSYKVQSTITSR SSALDMENFR TILDDLR TILDDLRAEDHFSVIDFNQNIR |
| **27** | 34 | 12 | 205.15 | 20.9 | 0.38 | 1.30 | 0.389 | 1.31 | 0.624 | 1.54 | P19827.3 | Inter-alpha-trypsin inhibitor heavy chain H1 | ADVQAHGEGQEFSITCLVDEEEMK ADVQAHGEGQEFSITCLVDEEEMKK EVAFDLEIPK GMADQDGLKPTIDKPSEDSPPLEMLGPR GSLVQASEANLQAAQDFVR LDAQASFLPK LWAYLTIQELLAK QAVDTAVDGVFIR QLVHHFEIDVDIFEPQGISK QYYEGSEIVVAGR TAFISDFAVTADGNAFIGDIKDKVTAWK VTFQLTYEEVLK |
| **28** | 73 | 11 | 199.84 | 36.5 | -1.059 | 0.48 | 0.678 | 1.60 | 0.322 | 1.25 | P01871.3 | Ig mu chain C region | DVMQGTDEHVVCK EGKQVGSGVTTDQVQAEAK ESATITCLVTGFSPADVFVQWMQR FTCTVTHTDLPSPLK GQPLSPEKYVTSAPMPEPQAPGR GVALHRPDVYLLPPAR NVPLPVIAELPPK QVGSGVTTDQVQAEAK STGKPTLYNVSLVMSDTAGTCY YAATSQVLLPSKDVMQGTDEHVVCK YVTSAPMPEPQAPGR |
| **29** | 49 | 11 | 184.25 | 21.1 | -0.317 | 0.80 | 0.306 | 1.24 | 0.19 | 1.14 | P01042.2 | Kininogen-1 | DFVQPPTK DIPTNSPELEETLTHTITK ENFLFLTPDCK FKLDDDLEHQGGHVLDHGHK IASFSQNCDIYPGKDFVQPPTK LGQSLDCNAEVYVVPWEK TVGSDTFYSFK TVGSDTFYSFKYEIK TWQDCEYKDAAK YEIKEGDCPVQSGK YFIDFVAR |
| **30** | 16 | 11 | 156.93 | 16.7 | 0.145 | 1.11 | 1.876 | 3.67 | 1.754 | 3.37 | P04264.6 | Keratin, type II cytoskeletal 1 | DYQELMNTK IEISELNR NMQDMVEDYR QISNLQQSISDAEQR SKAEAESLYQSK SKAEAESLYQSKYEELQITAGR SLDLDSIIAEVK SLDLDSIIAEVKAQYEDIAQK TNAENEFVTIK WELLQQVDTSTR YEELQITAGR |
| **31** | 72 | 10 | 204.75 | 78.2 | 1.339 | 2.53 | 4.294 | 19.62 | 3.083 | 8.47 | P68871.2 | Hemoglobin subunit beta | EFTPPVQAAYQK FFESFGDLSTPDAVMGNPK FFESFGDLSTPDAVmGNPK GTFATLSELHCDK GTFATLSELHCDKLHVDPENFR LHVDPENFR LLVVYPWTQR SAVTALWGK VLGAFSDGLAHLDNLK VNVDEVGGEALGR VVAGVANALAHKYH |
| **32** | 25 | 10 | 189.08 | 26.7 | -0.989 | 0.50 | -1.061 | 0.48 | -0.561 | 0.68 | P06396.1 | Gelsolin | AQPVQVAEGSEPDGFWEALGGK DSQEEEKTEALTSAK EVQGFESATFLGYFK NWRDPDQTDGLGLSYLSSHIANVER QTQVSVLPEGGETPLFK SEDCFILDHGK TPSAAYLWVGTGASEAEK VHVSEEGTEPEAMLQVLGPKPALPAGTEDTAK VPEARPNSMVVEHPEFLK VPFDAATLHTSTAMAAQHGMDDDGTGQK |
| **33** | 23 | 9 | 169.19 | 21 | 0.722 | 1.65 | 0.873 | 1.83 | 1.682 | 3.21 | P43652.1 | Afamin | AESPEVCFNEESPK ESLLNHFLYEVAR HELTDEELQSLFTNFANVVDK HPDLSIPELLR IAPQLSTEELVSLGEK KSDVGFLPPFPTLDPEEK LKHELTDEELQSLFTNFANVVDK SDVGFLPPFPTLDPEEK TYVPPPFSQDLFTFHADMCQSQNEELQR |
| **34** | 19 | 9 | 154.97 | 31.7 | 0.528 | 1.44 | 1.256 | 2.39 | 1.746 | 3.35 | P08697.3 | Alpha-2-antiplasmin | DSFHLDEQFTVPVEMMQAR ELKEQQDSPGNKDFLQSLK EQQDSPGNKDFLQSLK GFPIKEDFLEQSEQLFGAKPVSLTGK HQMDLVATLSQLGLQELFQAPDLR IQEFLSGLPEDTVLLLLNAIHFQGFWR LCQDLGPGAFR QEDDLANINQWVK WFLLEQPEIQVAHFPFK |
| **35** | 37 | 8 | 164.39 | 34.8 | -0.721 | 0.61 | -0.451 | 0.73 | -0.717 | 0.61 | P02765.1 | Alpha-2-HS-glycoprotein | AQLVPLPPSTYVEFTVSGTDCVAK EHAVEGDCDFQLLK EHAVEGDCDFQLLKLDGK HTFMGVVSLGSPSGEVSHPR TVVQPSVGAAAGPVVPPCPGR VWPQQPSGELFEIEIDTLETTCHVLDPTPVAR |
| **36** | 20 | 8 | 153.51 | 20.4 | 0.08 | 1.06 | 0.422 | 1.34 | 0.632 | 1.55 | P10643.2 | Complement component C7 | ELSHLPSLYDYSAYR GGGAGFISGLSYLELDNPAGNKR LLEPHCFPLSLVPTEFCPSPPALK LTPLYELVK SCVGETTESTQCEDEELEHLR SVAVYGQYGGQPCVGNAFETQSCEPTR VLFYVDSEK VTVSCSGGMSLEGPSAFLCGSSLK |
| **37** | 30 | 8 | 156.9 | 36.8 | -0.053 | 0.96 | 2.741 | 6.69 | 2.19 | 4.56 | P02763.1 | Alpha-1-acid glycoprotein 1 /orosomucoid 1 | EQLGEFYEALDCLR NWGLSVYADKPETTK SDVVYTDWK SDVVYTDWKK TEDTIFLR TYMLAFDVNDEK TYMLAFDVNDEKNWGLSVYADKPETTK YVGGQEHFAHLLILR |
| **38** | 23 | 8 | 143.68 | 35.6 | 1.318 | 2.49 | 0.562 | 1.48 | 1.019 | 2.03 | P02649.1 | Apolipoprotein E | AATVGSLAGQPLQER ALMDETMKELK AYKSELEEQLTPVAEETR FWDYLR GEVQAMLGQSTEELR SWFEPLVEDMQR VQAAVGTSAAPVPSDNH WVQTLSEQVQEELLSSQVTQELR |
| **39** | 22 | 8 | 143.1 | 30.8 | -0.857 | 0.55 | -0.4 | 0.76 | -0.68 | 0.62 | O43866.1 | CD5 antigen-like | EATLQDCPSGPWGK ELGCGAASGTPSGILYEPPAEK ELGCGAASGTPSGILYEPPAEKEQK FWGFHDCTHQEDVAVICSG GQWGTVCDDGWDIK GQWGTVCDDGWDIKDVAVLCR GVWGSVCDDNWGEKEDQVVCK IWLDNVR |
| **40** | 14 | 8 | 134.58 | 22.4 | 0.099 | 1.07 | 0.982 | 1.98 | 1.68 | 3.20 | P36955.4 | Pigment epithelium-derived factor | ALYYDLISSPDIHGTYK ALYYDLISSPDIHGTYKELLDTVTAPQK EIPDEISILLLGVAHFK ELLDTVTAPQK KTSLEDFYLDEER LAAAVSNFGYDLYR LQSLFDSPDFSK YGLDSDLSCK |
| **41** | 42 | 8 | 133.48 | 31.8 | 0.066 | 1.05 | 0.375 | 1.30 | 0.436 | 1.35 | P02749.3 | Beta-2-glycoprotein 1 | ATFGCHDGYSLDGPEEIECTK CSYTEDAQCIDGTIEVPK CTEEGKWSPELPVCAPIICPPPSIPTFATLR DKATFGCHDGYSLDGPEEIECTK KCSYTEDAQCIDGTIEVPK TCPKPDDLPFSTVVPLK TFYEPGEEITYSCKPGYVSR WSPELPVCAPIICPPPSIPTFATLR |
| **42** | 25 | 8 | 129.38 | 23.6 | 0.119 | 1.09 | 0.238 | 1.18 | 0.839 | 1.79 | P10909.1 | Clusterin | ASSIIDELFQDR EILSVDCSTNNPSQAK KTLLSNLEEAK LFDSDPITVTVPVEVSR QQTHMLDVMQDHFSR RELDESLQVAER TLLSNLEEAK VTTVASHTSDSDVPSGVTEVVVK |
| **43** | 48 | 7 | 152.35 | 72.7 | 0.207 | 1.15 | 0.509 | 1.42 | 0.892 | 1.86 | P02766.1 | Transthyretin | AADDTWEPFASGK ALGISPFHEHAEVVFTANDSGPR GSPAINVAVHVFR KAADDTWEPFASGK TSESGELHGLTTEEEFVEGIYK TSESGELHGLTTEEEFVEGIYKVEIDTK YTIAALLSPYSYSTTAVVTNPKE |
| **44** | 34 | 7 | 128.73 | 23.8 | -0.213 | 0.86 | 0.175 | 1.13 | 0.022 | 1.02 | P04217.4 | Alpha-1B-glycoprotein | CEGPIPDVTFELLR LELHVDGPPPRPQLR LLELTGPK SLPAPWLSMAPVSWITPGLK SWVPHTFESELSDPVELLVAES TPGAAANLELIFVGPQHAGNYR VTLTCVAPLSGVDFQLR |
| **45** | 20 | 7 | 120.08 | 13.8 | 0.698 | 1.62 | 2.23 | 4.69 | 2.817 | 7.05 | P05155.2 | Plasma protease C1 inhibitor | GVTSVSQIFHSPDLAIR IKVTTSQDMLSIMEK LEDMEQALSPSVFK LLDSLPSDTR TNLESILSYPK VTTSQDMLSIMEK |
| **46** | 50 | 7 | 112.67 | 85.8 | 0.356 | 1.28 | 1.266 | 2.40 | 0.362 | 1.29 | P0CG05.1 | Ig lambda-2 chain C regions | AAPSVTLFPPSSEELQANK ADSSPVKAGVETTTPSK AGVETTTPSK AGVETTTPSKQSNNKYAASSYLSLTPEQWK ATLVCLISDFYPGAVTVAWK SYSCQVTHEGSTVEK YAASSYLSLTPEQWK |
| **47** | 12 | 7 | 111.61 | 11.5 | -0.227 | 0.85 | 1.483 | 2.80 | 1.061 | 2.09 | P13671.3 | Complement component C6 | DLTSLGHNENQQGSFSSQGGSSFSVPIFYSSK IGESIELTCPK SEYGAALAWEK TECIKPVVQEVLTITPFQR TFSEWLESVK VPANLENVGFEVQTAEDDLK VPANLENVGFEVQTAEDDLKTDFYK |
| **48** | 27 | 7 | 108.78 | 19.2 | -0.396 | 0.76 | -0.19 | 0.88 | 0.219 | 1.16 | P04004.1 | Vitronectin | DWHGVPGQVDAAMAGR GQYCYELDEK LIRDVWGIEGPIDAAFTR MDWLVPATCEPIQSVFFFSGDK RVDTVDPPYPR SIAQYWLGCPAPGHL VDTVDPPYPR |
| **49** | 26 | 7 | 105.71 | 49.2 | 0.778 | 1.71 | 2.857 | 7.25 | 1.029 | 2.04 | P69905.2 | Hemoglobin subunit alpha | KVADALTNAVAHVDDMPNALSALSDLHAHK KVADALTNAVAHVDDMPNALSALSDLHAHKLR TYFPHFDLSHGSAQVK VADALTNAVAHVDDMPNALSALSDLHAHK VADALTNAVAHVDDMPNALSALSDLHAHKLR VDPVNFK VGAHAGEYGAEALER |
| **50** | 22 | 6 | 114.22 | 30.3 | 0.563 | 1.48 | 3.444 | 10.88 | 3.097 | 8.56 | P19652.2 | Alpha-1-acid glycoprotein 2 | EQLGEFYEALDCLCIPR NWGLSFYADKPETTK SDVMYTDWK TEDTIFLR TLMFGSYLDDEK TLMFGSYLDDEKNWGLSFYADKPETTK |
| **51** | 23 | 6 | 110.95 | 21.5 | -0.121 | 0.92 | 0.276 | 1.21 | 0.334 | 1.26 | P02760.1 | Protein AMBP | AFIQLWAFDAVK EDSCQLGYSAGPCMGMTSR ETLLQDFR GECVPGEQEPEPILIPR KEDSCQLGYSAGPCMGMTSR VVAQGVGIPEDSIFTMADR |
| **52** | 12 | 6 | 92.51 | 26.4 | -0.779 | 0.58 | -0.471 | 0.72 | 0.414 | 1.33 | P27169.3 | Serum paraoxonase/arylesterase 1 | EVQPVELPNCNLVK FDVSSFNPHGISTFTDEDNAMYLLVVNHPDAK GIETGSEDLEILPNGLAFISSGLKYPGIK IFFYDSENPPASEVLR ILLMDLNEEDPTVLELGITGSK IQNILTEEPK |
| **53** | 52 | 5 | 93.68 | 79.2 | 0.006 | 1.00 | 1.529 | 2.89 | 0.753 | 1.69 | P01834.1 | Ig kappa chain C region | DSTYSLSSTLTLSK SGTASVVCLLNNFYPR TVAAPSVFIFPPSDEQLK VDNALQSGNSQESVTEQDSK VYACEVTHQGLSSPVTK |
| **54** | 9 | 5 | 85.93 | 14 | -0.264 | 0.83 | 0.869 | 1.83 | -0.298 | 0.81 | P07357.2 | Complement component C8 alpha chain | ALDQYLMEFNACR AMAVEDIISR FGGTICSGDIWDQASCSSSTTCVR LGSLGAACEQTQTEGAK YNPVVIDFEMQPIHEVLR |
| **55** | 12 | 5 | 72.06 | 10.2 | -0.211 | 0.86 | 0.308 | 1.24 | 0.186 | 1.14 | P05156.2 | Complement factor I | AQLGDLPWQVAIK GLETSLAECTFTK IVIEYVDR TMGYQDFADVVCYTQK VFSLQWGEVK |
| **56** | 9 | 5 | 71.22 | 10.5 | -0.022 | 0.98 | -0.283 | 0.82 | 0.613 | 1.53 | P06681.2 | Complement C2 | AVISPGFDVFAK KNQGILEFYGDDIALLK LDVDWR LLGMETMAWQEIR QPYSYDFPEDVAPALGTSFSHMLGATNPTQK |
| **57** | 7 | 5 | 69.93 | 13.7 | 1.051 | 2.07 | 1.987 | 3.96 | 1.562 | 2.95 | P08670.2 | Vimentin precursor | MSTRSVSSSS EEMLQREEAE RKLLEGEESR ISLPLPNFSS TLLIKTVETR |
| **58** | 7 | 5 | 69.14 | 14.6 | 1.098 | 2.14 | 2.895 | 7.44 | 3.015 | 8.08 | P05546.3 | Heparin cofactor 2 | GGETAQSADPQWEQLNNK HQGTITVNEEGTQATTVTTVGFMPLSTQVR IAIDLFK NYNLVESLK QFPILLDFK |
| **59** | 36 | 5 | 73.15 | 37.3 | 0.216 | 1.16 | 0.759 | 1.69 | 1.834 | 3.57 | P02656.1 | Apolipoprotein C-III | DALSSVQESQVAQQAR DYWSTVK GWVTDGFSSLK GWVTDGFSSLKDYWSTVK TAKDALSSVQESQVAQQAR |
| **60** | 32 | 4 | 89.49 | 9.8 | 0.117 | 1.08 | 1.82 | 3.53 | 2.138 | 4.40 | P01019.1 | Angiotensinogen | ALQDQLVLVAAK DPTFIPAPIQAK SLDFTELDVAAEK SLDFTELDVAAEKIDR |
| **61** | 22 | 4 | 76.36 | 12.3 | 1.212 | 2.32 | 1.145 | 2.21 | 0.544 | 1.46 | P04196.1 | Histidine-rich glycoprotein | DGYLFQLLR GEVLPLPEANFPSFPLPHHK GGEGTGYFVDFSVR YKEENDDFASFRVDR |
| **62** | 5 | 4 | 71.51 | 11.7 | -0.061 | 0.96 | 1.442 | 2.72 | 1.427 | 2.69 | P35908.2 | Keratin, type II cytoskeletal 2 epidermal | IEISELNR NLDLDSIIAEVK SKEEAEALYHSKYEELQVTVGR VDLLNQEIEFLK |
| **63** | 42 | 4 | 71.67 | 42 | 0.95 | 1.93 | 3.061 | 8.35 | 3.757 | 13.52 | P02652.1 | Apolipoprotein A-II | EPCVESLVSQYFQTVTDYGK EPCVESLVSQYFQTVTDYGKDLMEK EPCVESLVSQYFQTVTDYGKDLmEK EQLTPLIK SPELQAEAK |
| **64** | 9 | 4 | 71.13 | 14.4 | -0.588 | 0.67 | -1.253 | 0.42 | -0.359 | 0.78 | Q96PD5.1 | N-acetylmuramoyl-L-alanine amidase | EFTEAFLGCPAIHPR EGKEYGVVLAPDGSTVAVEPLLAGLEAGLQGR GCPDVQASLPDAK GSQTQSHPDLGTEGCWDQLSAPR |
| **65** | 11 | 4 | 69.71 | 17.7 | 0.279 | 1.21 | 1.562 | 2.95 | 2.39 | 5.24 | P08185.1 | Corticosteroid-binding globulin | GTWTQPFDLASTR HYYESEVLAMNFQDWATASR IVDLFSGLDSPAILVLVNYIFFK WSAGLTSSQVDLYIPK |
| **66** | 8 | 4 | 68.08 | 9.7 | 0.486 | 1.40 | 2.058 | 4.16 | 2.259 | 4.79 | P13645.6 | Keratin, type I cytoskeletal 10 | IEISELNR NLDLDSIIAEVK SKEEAEALYHSKYEELQVTVGR VDLLNQEIEFLK |
| **67** | 8 | 4 | 66.79 | 23.2 | -0.295 | 0.82 | 0.641 | 1.56 | 1.573 | 2.98 | P05090.1 | Apolipoprotein D | CPNPPVQENFDVNK MTVTDQVNCPK NPNLPPETVDSLK WYEIEK |
| **68** | 8 | 4 | 65.72 | 43.5 | 0.582 | 1.50 | 0.618 | 1.53 | 1.72 | 3.29 | P02655.1 | Apolipoprotein C-II | ESLSSYWESAK STAAMSTYTGIFTDQVLSVLK STAAMSTYTGIFTDQVLSVLKGEE TYLPAVDEK |
| **69** | 10 | 3 | 68.38 | 14.4 | -0.056 | 0.96 | 0.839 | 1.79 | 1.445 | 2.72 | P35527.3 | Keratin, type I cytoskeletal 9 | MTLDDFR SDLEMQYETLQEELMALK TLLDIDNTR |
| **70** | 7 | 3 | 61.94 | 11.6 | 0.233 | 1.18 | -0.402 | 0.76 | 0.018 | 1.01 | P02748.2 | Complement component C9 | AIEDYINEFSVR FEGIACEISK RQCVPTEPCEDAEDDCGNDFQCSTGR |
| **71** | 7 | 3 | 54.01 | 16.8 | -0.514 | 0.70 | -0.74 | 0.60 | -0.604 | 0.66 | P07360.3 | Complement component C8 gamma chain | VQEAHLTEDQIFYFPK YGFCEAADQFHVLDEVR YGFCEAADQFHVLDEVRR |
| **72** | 6 | 3 | 51.01 | 8.2 | -0.094 | 0.94 | 0.36 | 1.28 | 0.782 | 1.72 | P07358.3 | Complement component C8 beta chain | DTMVEDLVVLVR IPGIFELGISSQSDR VKVEPLYELVTATDFAYSSTVR |
| **73** | 5 | 3 | 50.09 | 14.8 | -1.661 | 0.32 | -0.514 | 0.70 | -1.986 | 0.25 | P01880.2 | Ig delta chain C region | DSYYMTSSQLSTPLQQWR TPECPSHTQPLGVYLLTPAVQDLWLR VPTGGVEEGLLER |
| **74** | 4 | 3 | 49.63 | 8.1 | -0.145 | 0.90 | -0.237 | 0.85 | 0.724 | 1.65 | P09871.1 | Complement C1s subcomponent | LLEVPEGR MGPTVSPICLPGTSSDYNLMDGDLGLISGWGR SNALDIIFQTDLTGQK |
| **75** | 5 | 3 | 48.97 | 13.6 | -0.486 | 0.71 | -0.024 | 0.98 | 0.426 | 1.34 | P51884.2 | Lumican | NIPTVNENLENYYLEVNQLEK SLEDLQLTHNK SLEYLDLSFNQIAR |
| **76** | 3 | 3 | 46.74 | 5.6 | -0.455 | 0.73 | 0.587 | 1.50 | 0.876 | 1.84 | P07225.1 | Vitamin K-dependent protein S | FSAEFDFR NIPGDFECECPEGYR SQDILLSVENTVIYR |
| **77** | 5 | 3 | 44.46 | 6.1 | 0.288 | 1.22 | -0.01 | 0.99 | 0.103 | 1.07 | P03952.1 | Plasma kallikrein | GGDVASMYTPNAQYCQMR VAEYMDWILEK VLTPDAFVCR |
| **78** | 3 | 3 | 44.16 | 9.1 | 0.459 | 1.37 | 1.226 | 2.34 | 1.276 | 2.42 | P00748.3 | Coagulation factor XII | AEEHTVVLTVTGEPCHFPFQYHR LHEAFSPVSYQHDLALLR NPDNDIRPWCFVLNR |
| **79** | 3 | 3 | 39.68 | 8.3 | -0.46 | 0.73 | -0.065 | 0.96 | 0.874 | 1.83 | P05154.3 | Plasma serine protease inhibitor | ADFTFDLYR GFQQLLQELNQPR MQILEGLGLNLQK |
| **80** | 5 | 3 | 34.62 | 17.8 | 0.89 | 1.85 | 1.503 | 2.83 | 0.264 | 1.20 | P20851.1 | C4b-binding protein beta chain | EVEGQILGTYVCIK NLCEAMENFMQQLK SQCLEDHTWAPPFPICK |
| **81** | 8 | 2 | 69.93 | 12.8 | 0.121 | 1.09 | 0.73 | 1.66 | 0.649 | 1.57 | P05160.3 | Coagulation factor XIII B chain | GDTYPAELYITGSILR WSSPPVCLEPCTVNVDYMNR |
| **82** | 8 | 2 | 49.53 | 17.4 | 0.155 | 1.11 | 0.649 | 1.57 | 0.482 | 1.40 | P36980.1 | Complement factor H-related protein 2 | TGDIVEFVCK YKPFSQVPTGEVFYYSCEYNFVSPSK |
| **83** | 14 | 2 | 41.95 | 30.2 | -0.459 | 0.73 | -0.079 | 0.95 | -0.326 | 0.80 | P04206.1 | Ig kappa chain V-III region GOL | EIVLTQSPGTLSLSPGER FSGSGSGTDFTLTISR |
| **84** | 6 | 2 | 40.28 | 13.7 | -0.296 | 0.81 | -0.831 | 0.56 | 0.79 | 1.73 | O75636.2 | Ficolin-3 | ALPVFCDMDTEGGGWLVFQR ELLSQGATLSGWYHLCLPEGR |
| **85** | 7 | 2 | 40.02 | 7.1 | 0.303 | 1.23 | -2.501 | 0.18 | -1.798 | 0.29 | P02743.2 | Serum amyloid P-component | IVLGQEQDSYGGK IVLGQEQDSYGGKFDR |
| **86** | 8 | 2 | 38.38 | 31.8 | -1.137 | 0.45 | 0.201 | 1.15 | -0.192 | 0.88 | P01617.1 | Ig kappa chain V-II region TEW | DIVMTQSPLSLPVTPGEPASISCR FSGSGSGTDFTLK |
| **87** | 4 | 2 | 35.37 | 10.9 | -1.378 | 0.38 | -0.897 | 0.54 | -0.822 | 0.57 | P01857.1 | Ig gamma-1 chain C region | TPEVTCVVVDVSHEDPEVK TTPPVLDSDGSFFLYSK |
| **88** | 5 | 2 | 34.94 | 10.3 | 0.108 | 1.08 | 1.05 | 2.07 | 1.304 | 2.47 | P05543.2 | Thyroxine-binding globulin | EGQMESVEAAMSSK GTEAAAVPEVELSDQPENTFLHPIIQIDR |
| **89** | 3 | 2 | 32.73 | 7 | 0.362 | 1.29 | 1.105 | 2.15 | 1.99 | 3.97 | P29622.3 | Kallistatin | EIEEVLTPEMLMR VPMMLQDQEHHWYLHDR |
| **90** | 4 | 2 | 30.17 | 6.5 | 0.27 | 1.21 | -0.58 | 0.67 | 0.363 | 1.29 | O14791.5 | Apolipoprotein L1 | VNEPSILEMSR VTEPISAESGEQVER |
| **91** | 2 | 2 | 29.91 | 5.7 | -0.626 | 0.65 | -0.994 | 0.50 | -0.959 | 0.51 | Q9BXR6.1 | Complement factor H-related protein 5 | EIMENYNIALR ITCTEEGWSPTPK YKPFSQVPTGEVFYYSCEYNFVSPSK |
| **92** | 3 | 2 | 28.95 | 7.1 | 0.658 | 1.58 | 0.94 | 1.92 | 1.342 | 2.54 | Q14520.1 | Plasma hyaluronan-binding protein | NPDADEKPWCFIK TVCLPDGSFPSGSECHISGWGVTETGK |
| **93** | 4 | 2 | 27.77 | 18.7 | 0.134 | 1.10 | -0.338 | 0.79 | 0.397 | 1.32 | P02775.3 | Platelet basic protein | GKEESLDSDLYAELR ICLDPDAPR |
| **94** | 2 | 2 | 27.67 | 4.6 | -0.002 | 1.00 | 0.063 | 1.04 | 0.605 | 1.52 | P35858.1 | Insulin-like growth factor-binding protein complex acid labile subunit | DLHFLEELQLGHNR LAELPADALGPLQR |
| **95** | 4 | 2 | 27.28 | 4.7 | -0.618 | 0.65 | 0.087 | 1.06 | 0.173 | 1.13 | P22792.3 | Carboxypeptidase N subunit 2 | AGGSWDLAVQER DHLGFQVTWPDESK |
| **96** | 5 | 2 | 25.48 | 8.8 | 0.369 | 1.29 | 1.276 | 2.42 | 1.551 | 2.93 | P22352.2 | Glutathione peroxidase 3 | FLVGPDGIPIMR MDILSYMR |
| **97** | 5 | 1 | 32.08 | 7.4 | 1.574 | 2.98 | 3.349 | 10.19 | 2.292 | 4.90 | P02750.2 | Leucine-rich alpha-2-glycoprotein | DLLLPQPDLR |
| **98** | 3 | 1 | 28.77 | 4.9 | -0.662 | 0.63 | -0.354 | 0.78 | -0.007 | 1.00 | Q06033.2 | Inter-alpha-trypsin inhibitor heavy chain H3 | STSIVIMLTDGDANVGESRPEK |
| **99** | 3 | 1 | 25 | 5.7 | 0.297 | 1.23 | -0.413 | 0.75 | 0.302 | 1.23 | Q96KN2.4 | Beta-Ala-His dipeptidase | AIHLDLEEYR |
| **100** | 12 | 1 | 23.86 | 15 | 0.485 | 1.40 | 0.12 | 1.09 | -0.172 | 0.89 | P01766.1 | Ig heavy chain V-III region BRO | EVQLVESGGGLVQPGGSLR |
| **101** | 4 | 1 | 22.6 | 15.7 | -0.303 | 0.81 | -0.294 | 0.82 | -0.46 | 0.73 | P01598.1 | Ig kappa chain V-I region EU | DIQMTQSPSTLSASVGDR |
| **102** | 6 | 1 | 20.6 | 15.7 | -0.303 | 0.81 | -0.017 | 0.99 | -0.511 | 0.70 | P01593.1 | Ig kappa chain V-I region AG | DIQMTQSPSSLSASVGDR |
| **103** | 1 | 1 | 20.58 | 2.9 | -0.997 | 0.50 | -0.01 | 0.99 | 1.064 | 2.09 | P80108.3 | Phosphatidylinositol-glycan-specific phospholipase D | FGGVLHLSDLDDDGLDEIIMAAPLR |
| **104** | 1 | 1 | 20.35 | 2.3 | 0.24 | 1.18 | 0.751 | 1.68 | 0.047 | 1.03 | P02671.2 | Fibrinogen alpha chain | TFPGFFSPMLGEFVSETESR |
| **105** | 1 | 1 | 20.17 | 15.7 | -0.466 | 0.72 | 0.175 | 1.13 | -0.303 | 0.81 | P01611.1 | Ig kappa chain V-I region Wes | DIQMTQSPSSVSASVGDR |
| **106** | 2 | 1 | 19.01 | 0.2 | -0.779 | 0.58 | 0.078 | 1.06 | -1.914 | 0.27 | P08519.1 | Apolipoprotein(a) | EWFSETFQK |
| **107** | 1 | 1 | 18.97 | 3.4 | -1.914 | 0.27 | -0.28 | 0.82 | -0.859 | 0.55 | P06276.1 | Cholinesterase | AILQSGSFNAPWAVTSLYEAR |
| **108** | 6 | 1 | 18.57 | 15.1 | -0.452 | 0.73 | 0.608 | 1.52 | -0.115 | 0.92 | P01774.1 | Ig heavy chain V-III region POM | EVQLLESGGGLVQPGGSL |
| **109** | 2 | 1 | 18.3 | 14.9 | -2.438 | 0.18 | -0.424 | 0.75 | -0.6 | 0.66 | P01625.2 | Ig kappa chain V-IV region Len | DIVMTQSPDSLAVSLGER |
| **110** | 5 | 1 | 17.59 | 9.4 | -0.465 | 0.72 | -0.294 | 0.82 | -0.165 | 0.89 | P01591.4 | Immunoglobulin J chain | CYTAVVPLVYGGETK |
| **111** | 2 | 1 | 17.31 | 15.7 | 0.32 | 1.25 | -1.093 | 0.47 | 0.113 | 1.08 | P01714.1 | Ig lambda chain V-III region SH | SELTQDPAVSVALGQTVR |
| **112** | 1 | 1 | 16.92 | 3.9 | 0 | 1.00 | 0 | 1.00 | 0 | 1.00 | P01854.1 | Ig epsilon chain C region | AAPEVYAFATPEWPGS |
| **113** | 2 | 1 | 16.69 | 10.8 | 0.593 | 1.51 | 5.316 | 39.84 | 5.85 | 57.68 | P02654.1 | Apolipoprotein C-I | WIYHLTEGSTDLR |
| **114** | 3 | 1 | 16.17 | 16.8 | 1.542 | 2.91 | -0.776 | 0.58 | -0.286 | 0.82 | P01717.1 | Ig lambda chain V-IV region Hil | SYELTQPPSVSVSPGQTAR |
| **115** | 2 | 1 | 16.03 | 4.8 | -0.731 | 0.60 | -0.426 | 0.74 | 0.059 | 1.04 | P15169.1 | Carboxypeptidase N catalytic chain | HLYVLEFSDHPGIHEPLEPEV |
| **116** | 2 | 1 | 15.76 | 16.3 | 1.213 | 2.32 | 1.303 | 2.47 | 1.018 | 2.03 | P0DJI8.1 | Serum amyloid A-1 protein | FFGHGAEDSLADQAANEWG |
| **117** | 2 | 1 | 15.68 | 3.7 | -0.355 | 0.78 | 0.782 | 1.72 | 0.888 | 1.85 | Q96IY4.2 | Carboxypeptidase B2 | * |
| **118** | 1 | 1 | 15.59 | 7.8 | -0.601 | 0.66 | -0.353 | 0.78 | -1.48 | 0.36 | P04433.1 | Ig kappa chain V-III region VG | DIQMTQSPSSLSASVGDR |
| **119** | 1 | 1 | 15.46 | 20.5 | -3.216 | 0.11 | -3.216 | 0.11 | 0 | 1.00 | P01616.1 | Ig kappa chain V-II region MIL | DIVLTQSPLSLPVTPGEPASISCR |
| **120** | 1 | 1 | 15.22 | 1 | -0.79 | 0.58 | -1.11 | 0.46 | -1.058 | 0.48 | P07996.2 | Thrombospondin-1 | FVFGTTPEDILR |
| **121** | 2 | 1 | 15.18 | 3.6 | -0.633 | 0.64 | 0.248 | 1.19 | 0.675 | 1.60 | P27918.2 | Properdin | * |
| **122** | 1 | 1 | 14.72 | 5.1 | -0.768 | 0.59 | 0.173 | 1.13 | 1.389 | 2.62 | P01861.1 | Ig gamma-4 chain C region | TTPPVLDSDGSFFLYSR |
| **123** | 1 | 1 | 14 | 5.2 | -1.191 | 0.44 | -0.393 | 0.76 | -2.173 | 0.22 | P01859.2 | Ig gamma-2 chain C region | TTPPMLDSDGSFFLYSK |
| **124** | 1 | 1 | 13.71 | 11.8 | 1.079 | 2.11 | 0.809 | 1.75 | 1.519 | 2.87 | P05109.1 | Protein S100-A8 | LLETECPQYIR |
| **125** | 1 | 1 | 13.49 | 10.8 | -0.316 | 0.80 | 1.645 | 3.13 | 2.141 | 4.41 | P31151.4 | Protein S100-A7 | GTNYLADVFEK |
| **126** | 1 | 1 | 13.47 | 11.5 | 0.476 | 1.39 | -1.972 | 0.25 | -1.698 | 0.31 | P35542.2 | Serum amyloid A-4 protein | SFFKEALQGVGDMGR |
| **127** | 4 | 1 | 13.33 | 4.5 | -1.809 | 0.29 | -4.079 | 0.06 | -4.277 | 0.05 | P50897.1 | Palmitoyl-protein thioesterase 1 | CPGESSHICDFIRK |
| **128** | 2 | 1 | 13.27 | 7.7 | 1.182 | 2.27 | 3.267 | 9.63 | 2.284 | 4.87 | P01781.1 | Ig heavy chain V-III region GAL | GLEWVANIK |
| **129** | 3 | 1 | 13.05 | 2.7 | -0.041 | 0.97 | 2.091 | 4.26 | 3.573 | 11.90 | P50213.1 | Isocitrate dehydrogenase [NAD] subunit alpha, mitochondrial | IAEFAFEYAR |
| **130** | 2 | 1 | 12.97 | 6.9 | -0.502 | 0.71 | 1.137 | 2.20 | 1.853 | 3.61 | O95445.2 | Apolipoprotein M | WIYHLTEGSTDLR |
| **131** | 1 | 1 | 12.74 | 15.7 | -0.377 | 0.77 | -1.688 | 0.31 | -2.158 | 0.22 | P04430.1 | Ig kappa chain V-I region BAN | DIQLTQSPSSLSASVGDR |
| **132** | 1 | 1 | 12.6 | 7.5 | -0.26 | 0.84 | -1.288 | 0.41 | 0.82 | 1.77 | P04070.1 | Vitamin K-dependent protein C | STTDNDIALLHLAQPATLSQTIVPICLPDSGLAER |
| **133** | 1 | 1 | 12.39 | 13.1 | 1.091 | 2.13 | 4.508 | 22.75 | 4.485 | 22.39 | P06702.1 | Protein S100-A9 | LLETECPQYIR |
| **134** | 1 | 1 | 12.32 | 0.6 | -1.459 | 0.36 | -1.528 | 0.35 | -1.132 | 0.46 | Q5XPI4.1 | E3 ubiquitin-protein ligase RNF123 | FLQENASGR |
| **135** | 1 | 1 | 11.84 | 4.5 | -0.161 | 0.89 | -1.043 | 0.49 | 0.017 | 1.01 | P00742.2 | Coagulation factor X | ITVVAGEHNIEETEHTEQKR |
| **136** | 1 | 1 | 11.6 | 2.3 | -0.465 | 0.72 | 2.534 | 5.79 | 2.859 | 7.26 | P43251.2 | Biotinidase | VDLITFDTPFAGR |
| **137** | 1 | 1 | 11.49 | 4.3 | -0.789 | 0.58 | -2.115 | 0.23 | -1.821 | 0.28 | P00740.2 | Coagulation factor IX | ITVVAGEHNIEETEHTEQKR |
| **138** | 2 | 1 | 11.36 | 1.5 | 0.306 | 1.24 | 0.67 | 1.59 | 0.39 | 1.31 | O15265.1 | Ataxin-7 | IPPVPSTTSPISTR |
| **139** | 1 | 1 | 11.26 | 3.9 | -0.606 | 0.66 | 0.643 | 1.56 | -0.032 | 0.98 | Q7Z353.1 | Highly divergent homeobox | KNYGNSSVQASEMTVPQKPSVCHRPCK |
| **140** | 1 | 1 | 11.23 | 2.4 | 1.952 | 3.87 | -1.295 | 0.41 | 2.614 | 6.12 | P01833.4 | Polymeric immunoglobulin receptor | NADLQVLKPEPELVYEDLR |
| **141** | 2 | 1 | 11.16 | 10.9 | -1.142 | 0.45 | -3.046 | 0.12 | -4.978 | 0.03 | Q6ZN03.1 | Uncharacterized protein encoded by LINC00322 | * |
| **142** | 1 | 1 | 11.14 | 0.4 | 0.117 | 1.08 | 0 | 1.00 | -1.596 | 0.33 | Q96M86.2 | Dynein heavy chain domain-containing protein 1 | * |
| **143** | 1 | 1 | 11.13 | 4.7 | -0.191 | 0.88 | -2.062 | 0.24 | -1.817 | 0.28 | Q8N336.3 | ELMO domain-containing protein 1 | GMGLLGLYNLQYFAER |
| **144** | 2 | 1 | 11.12 | 5.2 | 0.039 | 1.03 | 1.466 | 2.76 | 1.978 | 3.94 | P09486.1 | SPARC | YIPPCLDSELTEFPLR |
| **145** | 1 | 1 | 10.91 | 2.9 | -0.682 | 0.62 | -0.815 | 0.57 | -0.56 | 0.68 | Q04756.1 | Hepatocyte growth factor activator | VQLSPDLLATLPEPASPGR |
| **146** | 1 | 1 | 10.89 | 11 | 2.137 | 4.40 | 3.163 | 8.96 | 0.979 | 1.97 | P02745.2 | Complement C1q subcomponent subunit A | GLFQVVSGGMVLQLQQGDQVWVEKDPK |
| **147** | 1 | 1 | 10.69 | 2 | 0.828 | 1.78 | 3.24 | 9.45 | 3.489 | 11.23 | Q9C0A0.3 | Contactin-associated protein-like 4 | RSENVDSAEAVLKSELNIQNAVNENQK |
| **148** | 1 | 1 | 10.54 | 4.5 | 2.535 | 5.80 | 5.277 | 38.77 | 3.418 | 10.69 | P00915.2 | Carbonic anhydrase 1 | ADGLAVIGVLMK |
| **149** | 1 | 1 | 10.51 | 0.7 | -0.839 | 0.56 | -0.232 | 0.85 | -0.346 | 0.79 | Q9P2R6.2 | Arginine-glutamic acid dipeptide repeats protein | TDLYFMPLAGSK |
| **150** | 1 | 1 | 10.47 | 6.9 | 0 | 1.00 | 0 | 1.00 | 0 | 1.00 | P05452.3 | Tetranectin | NWETEITAQPDGGK |
| **151** | 1 | 1 | 10.3 | 11.8 | 1.345 | 2.54 | 0.399 | 1.32 | 0 | 1.00 | Q16514.1 | Transcription initiation factor TFIID subunit 12 | * |
| **152** | 1 | 1 | 10.27 | 6.4 | 0.952 | 1.93 | 1.558 | 2.94 | 1.605 | 3.04 | Q9HBK9.3 | Arsenite methyltransferase | YGFQASNVTFIHGYIEKLGEAGIK |
| **153** | 2 | 1 | 10.21 | 1.3 | 0.085 | 1.06 | -3.562 | 0.08 | -4.584 | 0.04 | Q8WVM7.3 | Cohesin subunit SA-1 | CLKALQSLYTNRELFPK |
| **154** | 1 | 1 | 10.21 | 0.6 | -0.74 | 0.60 | -0.598 | 0.66 | -0.877 | 0.54 | Q9P1Q0.2 | Vacuolar protein sorting-associated protein 54 | * |
| **155** | 1 | 1 | 10.2 | 4.7 | 0.526 | 1.44 | -0.657 | 0.63 | -0.765 | 0.59 | Q08380.1 | Galectin-3-binding protein | AAFGQGSGPIMLDEVQCTGTEASLADCK |
| **156** | 1 | 1 | 10.18 | 15.7 | 0 | 1.00 | 0 | 1.00 | 0 | 1.00 | P01763.1 | Ig heavy chain V-III region WEA | QVQLVDSGGGLVEPGGSLR |
| **157** | 1 | 1 | 10.12 | 0 | -0.445 | 0.73 | 1.795 | 3.47 | 1.804 | 3.49 | Q8WZ42.4 | Titin | NTADLKWTVPEKDGGSPITNYIVEK |

**Table S7.** Complete details of protein identification and label-free MS data for differentially expressed proteins in meningioma grade I (compared to healthy controls).

| **Sl No.** | **Num Spectra** | **Unique Peptides** | **Score** | **Sequence Coverage (%)** | **HC I (Set 1)** | **HC I (Set 2)** | **HC (Set 3)** | **Average** | **MG I (Set 1)** | **MG I (Set 2)** | **MG I (Set 3)** | **Average** | **Fold-change** | **Accession number** | **Protein Name** |
| --- | --- | --- | --- | --- | --- | --- | --- | --- | --- | --- | --- | --- | --- | --- | --- |
| 1 | 415 | 75 | 1188.19 | 52.9 | 7.83E+07 | 8.14E+07 | 9.94E+07 | 86366667 | 1.78E+08 | 2.09E+08 | 2.26E+08 | 204333333 | 2.37 | P01024.2 | RecName: Full=Complement C3: AltName: Full=C3 and PZP-like alpha-2-macroglobulin domain-containing protein 1: Contains: RecName: Full=Complement C3 beta chain: Contains: RecName: Full=Complement C3 alpha chain: Contains: RecName: Full=C3a anaphylatoxin: Contains: RecName: Full=Acylation stimulating protein: Short=ASP: AltName: Full=C3adesArg: Contains: RecName: Full=Complement C3b alpha' chain: Contains: RecName: Full=Complement C3c alpha' chain fragment 1: Contains: RecName: Full=Complement C3dg fragment: Contains: RecName: Full=Complement C3g fragment: Contains: RecName: Full=Complement C3d fragment: Contains: RecName: Full=Complement C3f fragment: Contains: RecName: Full=Complement C3c alpha' chain fragment 2: Flags: Precursor |
| 2 | 695 | 67 | 1091.36 | 74 | 3.23E+08 | 3.52E+08 | 4.20E+08 | 365000000 | 6.00E+08 | 7.29E+08 | 7.63E+08 | 697333333 | 1.91 | P02787.3 | RecName: Full=Serotransferrin: Short=Transferrin: AltName: Full=Beta-1 metal-binding globulin: AltName: Full=Siderophilin: Flags: Precursor |
| 3 | 389 | 60 | 982.32 | 50 | 4.32E+07 | 4.16E+07 | 4.72E+07 | 44000000 | 2.39E+08 | 2.95E+08 | 3.03E+08 | 279000000 | 6.34 | A5A6I6.1 | RecName: Full=Serotransferrin: Short=Transferrin: AltName: Full=Beta-1 metal-binding globulin: AltName: Full=Siderophilin: Flags: Precursor |
| 4 | 269 | 36 | 600.5 | 74.5 | 4.29E+07 | 5.12E+07 | 5.44E+07 | 49500000 | 1.73E+08 | 2.06E+08 | 2.13E+08 | 197333333 | 3.99 | P01023.3 | RecName: Full=Alpha-2-macroglobulin: Short=Alpha-2-M: AltName: Full=C3 and PZP-like alpha-2-macroglobulin domain-containing protein 5: Flags: Precursor |
| 5 | 294 | 33 | 501.32 | 71.9 | 1.56E+07 | 1.43E+08 | 1.22E+08 | 93533333 | 9.86E+07 | 1.59E+08 | 3.01E+08 | 186200000 | 1.99 | P02647.1 | RecName: Full=Apolipoprotein A-I: Short=Apo-AI: Short=ApoA-I: AltName: Full=Apolipoprotein A1: Contains: RecName: Full=Truncated apolipoprotein A-I: AltName: Full=Apolipoprotein A-I(1-242): Flags: Precursorgi\|380876859\|sp\|G3QY98.1\|APOA1_GORGO RecName: Full=Apolipoprotein A-I: Short=Apo-AI: Short=ApoA-I: AltName: Full=Apolipoprotein A1: Contains: RecName: Full=Truncated apolipoprotein A-I: Flags: Precursorgi\|385178604\|sp\|P0DJG0.1\|APOA1_PANTR RecName: Full=Apolipoprotein A-I: Short=Apo-AI: Short=ApoA-I: AltName: Full=Apolipoprotein A1: Contains: RecName: Full=Truncated apolipoprotein A-I: Flags: Precursor |
| 6 | 237 | 30 | 448.76 | 56.6 | 2.31E+08 | 1.77E+08 | 1.92E+08 | 200000000 | 2.81E+08 | 3.64E+08 | 5.50E+08 | 398333333 | 1.99 | P0DJG1.1 | RecName: Full=Apolipoprotein A-I: Short=Apo-AI: Short=ApoA-I: AltName: Full=Apolipoprotein A1: Contains: RecName: Full=Truncated apolipoprotein A-I: Flags: Precursor |
| 7 | 108 | 29 | 439.72 | 26.8 | 1.61E+07 | 1.07E+07 | 1.07E+07 | 12500000 | 4.63E+07 | 4.79E+07 | 3.98E+07 | 44666667 | 3.57 | P00738.1 | RecName: Full=Haptoglobin: AltName: Full=Zonulin: Contains: RecName: Full=Haptoglobin alpha chain: Contains: RecName: Full=Haptoglobin beta chain: Flags: Precursor |
| 8 | 153 | 26 | 416.22 | 48.7 | 1.39E+07 | 1.12E+07 | 1.47E+07 | 13266667 | 7.00E+07 | 1.04E+08 | 1.04E+08 | 92666667 | 6.98 | P00739.2 | RecName: Full=Haptoglobin-related protein: Flags: Precursor |
| 9 | 159 | 21 | 362.25 | 48 | 5.23E+07 | 7.18E+07 | 5.70E+07 | 60366667 | 6.07E+07 | 7.00E+07 | 9.71E+07 | 75933333 | 1.26 | P01009.3 | RecName: Full=Alpha-1-antitrypsin: AltName: Full=Alpha-1 protease inhibitor: AltName: Full=Alpha-1-antiproteinase: AltName: Full=Serpin A1: Contains: RecName: Full=Short peptide from AAT: Short=SPAAT: Flags: Precursor |
| 10 | 210 | 20 | 335.36 | 59.7 | 8.36E+07 | 9.92E+07 | 9.28E+07 | 91866667 | 1.67E+08 | 1.97E+08 | 2.82E+08 | 215333333 | 2.34 | Q5RCW5.1 | RecName: Full=Alpha-1-antitrypsin: AltName: Full=Alpha-1 protease inhibitor: AltName: Full=Alpha-1-antiproteinase: AltName: Full=Serpin A1: Flags: Precursor |
| 11 | 76 | 18 | 303.55 | 30.6 | 1.81E+07 | 8.96E+06 | 1.02E+07 | 12420000 | 3.17E+07 | 3.27E+07 | 3.18E+07 | 32066667 | 2.58 | P0C0L4.1 | RecName: Full=Complement C4-A: AltName: Full=Acidic complement C4: AltName: Full=C3 and PZP-like alpha-2-macroglobulin domain-containing protein 2: Contains: RecName: Full=Complement C4 beta chain: Contains: RecName: Full=Complement C4-A alpha chain: Contains: RecName: Full=C4a anaphylatoxin: Contains: RecName: Full=C4b-A: Contains: RecName: Full=C4d-A: Contains: RecName: Full=Complement C4 gamma chain: Flags: Precursor |
| 12 | 117 | 18 | 302.11 | 45.6 | 1.56E+07 | 1.25E+07 | 2.27E+07 | 16933333 | 6.34E+07 | 7.58E+07 | 8.22E+07 | 73800000 | 4.36 | P02768.2 | RecName: Full=Serum albumin: Flags: Precursor |
| 13 | 100 | 18 | 271.11 | 48.1 | 1.67E+07 | 9.17E+06 | 6.46E+06 | 10776667 | 7.03E+07 | 5.86E+07 | 7.27E+07 | 67200000 | 6.24 | P01871.3 | RecName: Full=Ig mu chain C region |
| 14 | 130 | 17 | 278.6 | 57.9 | 4.17E+07 | 6.11E+07 | 5.26E+07 | 51800000 | 1.16E+08 | 1.19E+08 | 1.39E+08 | 124666667 | 2.41 | P04220.1 | RecName: Full=Ig mu heavy chain disease protein: AltName: Full=BOT |
| 15 | 111 | 16 | 218.42 | 32.7 | 3.52E+07 | 4.43E+07 | 4.55E+07 | 41666667 | 4.93E+07 | 6.70E+07 | 1.38E+08 | 84766667 | 2.03 | P01876.2 | RecName: Full=Ig alpha-1 chain C region |
| 16 | 57 | 15 | 197.1 | 20.3 | 3.67E+06 | 4.01E+06 | 4.89E+06 | 4190000 | 9.17E+06 | 3.52E+06 | 4.31E+06 | 5666666.7 | 1.35 | P01877.3 | RecName: Full=Ig alpha-2 chain C region |
| 17 | 109 | 14 | 243.23 | 49.3 | 5.14E+07 | 5.42E+07 | 5.23E+07 | 52633333 | 3.48E+07 | 5.22E+07 | 6.74E+07 | 51466667 | 0.98 | P00450.1 | RecName: Full=Ceruloplasmin: AltName: Full=Ferroxidase: Flags: Precursor |
| 18 | 82 | 14 | 232.59 | 44.6 | 1.41E+07 | 1.04E+07 | 7.95E+06 | 10816667 | 2.56E+07 | 1.70E+07 | 2.09E+07 | 21166667 | 1.96 | P02790.2 | RecName: Full=Hemopexin: AltName: Full=Beta-1B-glycoprotein: Flags: Precursor |
| 19 | 142 | 13 | 207.07 | 35.1 | 4.70E+07 | 4.59E+07 | 3.80E+07 | 43633333 | 3.91E+07 | 4.66E+07 | 5.79E+07 | 47866667 | 1.10 | P02774.1 | RecName: Full=Vitamin D-binding protein: Short=DBP: Short=VDB: AltName: Full=Gc-globulin: AltName: Full=Group-specific component: Flags: Precursor |
| 20 | 123 | 12 | 225.55 | 77.5 | 7.46E+07 | 9.03E+07 | 7.47E+07 | 79866667 | 5.89E+07 | 7.16E+07 | 8.74E+07 | 72633333 | 0.91 | P08603.4 | RecName: Full=Complement factor H: AltName: Full=H factor 1: Flags: Precursor |
| 21 | 83 | 12 | 194.08 | 49.5 | 1.97E+07 | 3.09E+07 | 3.69E+07 | 29166667 | 4.89E+07 | 7.29E+07 | 1.05E+08 | 75600000 | 2.59 | P01008.1 | RecName: Full=Antithrombin-III: Short=ATIII: AltName: Full=Serpin C1: Flags: Precursor |
| 22 | 31 | 12 | 133.57 | 19.7 | 3.22E+06 | 1.73E+06 | 1.84E+06 | 2263333.3 | 5.88E+06 | 8.03E+06 | 7.99E+06 | 7300000 | 3.23 | P04217.4 | RecName: Full=Alpha-1B-glycoprotein: AltName: Full=Alpha-1-B glycoprotein: Flags: Precursor |
| 23 | 30 | 11 | 168.91 | 24.7 | 2.79E+06 | 4.37E+06 | 1.51E+06 | 2890000 | 1.44E+07 | 1.91E+07 | 2.06E+07 | 18033333 | 6.24 | P02766.1 | RecName: Full=Transthyretin: AltName: Full=ATTR: AltName: Full=Prealbumin: AltName: Full=TBPA: Flags: Precursor |
| 24 | 42 | 11 | 159.52 | 4.5 | 6.53E+05 | 1.31E+06 | 1.08E+06 | 1014333.3 | 4.64E+06 | 4.54E+06 | 3.07E+06 | 4083333.3 | 4.03 | P02765.1 | RecName: Full=Alpha-2-HS-glycoprotein: AltName: Full=Alpha-2-Z-globulin: AltName: Full=Ba-alpha-2-glycoprotein: AltName: Full=Fetuin-A: Contains: RecName: Full=Alpha-2-HS-glycoprotein chain A: Contains: RecName: Full=Alpha-2-HS-glycoprotein chain B: Flags: Precursor |
| 25 | 120 | 9 | 155.12 | 92.4 | 3.41E+07 | 4.64E+07 | 4.60E+07 | 42166667 | 6.89E+07 | 9.70E+07 | 8.10E+07 | 82300000 | 1.95 | Q9N2D0.1 | RecName: Full=Alpha-2-HS-glycoprotein: AltName: Full=Fetuin-A: Contains: RecName: Full=Alpha-2-HS-glycoprotein chain A: Contains: RecName: Full=Alpha-2-HS-glycoprotein chain B: Flags: Precursor |
| 26 | 90 | 9 | 150.39 | 74 | 6.31E+07 | 7.04E+07 | 4.29E+07 | 58800000 | 1.74E+08 | 1.90E+08 | 1.77E+08 | 180333333 | 3.07 | P01042.2 | RecName: Full=Kininogen-1: AltName: Full=Alpha-2-thiol proteinase inhibitor: AltName: Full=Fitzgerald factor: AltName: Full=High molecular weight kininogen: Short=HMWK: AltName: Full=Williams-Fitzgerald-Flaujeac factor: Contains: RecName: Full=Kininogen-1 heavy chain: Contains: RecName: Full=T-kinin: AltName: Full=Ile-Ser-Bradykinin: Contains: RecName: Full=Bradykinin: AltName: Full=Kallidin I: Contains: RecName: Full=Lysyl-bradykinin: AltName: Full=Kallidin II: Contains: RecName: Full=Kininogen-1 light chain: Contains: RecName: Full=Low molecular weight growth-promoting factor: Flags: Precursor |
| 27 | 51 | 7 | 128.7 | 59.1 | 1.38E+07 | 2.29E+07 | 2.65E+07 | 21066667 | 1.13E+07 | 2.31E+06 | 4.79E+06 | 6133333.3 | 0.29 | P00751.2 | RecName: Full=Complement factor B: AltName: Full=C3/C5 convertase: AltName: Full=Glycine-rich beta glycoprotein: Short=GBG: AltName: Full=PBF2: AltName: Full=Properdin factor B: Contains: RecName: Full=Complement factor B Ba fragment: Contains: RecName: Full=Complement factor B Bb fragment: Flags: Precursor |
| 28 | 21 | 7 | 110.17 | 17.5 | 8.27E+05 | 3.09E+06 | 4.38E+06 | 2765666.7 | 2.80E+06 | 4.58E+06 | 1.23E+06 | 2870000 | 1.04 | P04114.2 | RecName: Full=Apolipoprotein B-100: Short=Apo B-100: Contains: RecName: Full=Apolipoprotein B-48: Short=Apo B-48: Flags: Precursor |
| 29 | 35 | 7 | 108.84 | 37.3 | 1.67E+07 | 1.94E+07 | 1.34E+07 | 16500000 | 1.44E+07 | 1.89E+07 | 2.06E+07 | 17966667 | 1.09 | P01834.1 | RecName: Full=Ig kappa chain C region |
| 30 | 17 | 7 | 105.07 | 16 | 4.77E+05 | 8.54E+05 | 2.39E+05 | 523333.33 | 1.05E+06 | 1.77E+06 | 3.66E+06 | 2160000 | 4.13 | P02652.1 | RecName: Full=Apolipoprotein A-II: Short=Apo-AII: Short=ApoA-II: AltName: Full=Apolipoprotein A2: Contains: RecName: Full=Truncated apolipoprotein A-II: AltName: Full=Apolipoprotein A-II(1-76): Flags: Precursorgi\|385178606\|sp\|P0DJG2.1\|APOA2_GORGO RecName: Full=Apolipoprotein A-II: Short=Apo-AII: Short=ApoA-II: AltName: Full=Apolipoprotein A2: Contains: RecName: Full=Truncated apolipoprotein A-II: Flags: Precursor |
| 31 | 44 | 7 | 96.93 | 16.7 | 1.14E+07 | 6.57E+06 | 8.33E+06 | 8766666.7 | 5.52E+06 | 5.30E+06 | 2.04E+07 | 10406667 | 1.19 | P68871.2 | RecName: Full=Hemoglobin subunit beta: AltName: Full=Beta-globin: AltName: Full=Hemoglobin beta chain: Contains: RecName: Full=LVV-hemorphin-7gi\|56749857\|sp\|P68872.2\|HBB_PANPA RecName: Full=Hemoglobin subunit beta: AltName: Full=Beta-globin: AltName: Full=Hemoglobin beta chaingi\|56749858\|sp\|P68873.2\|HBB_PANTR RecName: Full=Hemoglobin subunit beta: AltName: Full=Beta-globin: AltName: Full=Hemoglobin beta chain |
| 32 | 18 | 7 | 82.89 | 12.2 | 1.95E+06 | 1.13E+06 | 1.85E+06 | 1643333.3 | 1.17E+06 | 8.22E+05 | 1.50E+06 | 1164000 | 0.71 | P04004.1 | RecName: Full=Vitronectin: Short=VN: AltName: Full=S-protein: AltName: Full=Serum-spreading factor: AltName: Full=V75: Contains: RecName: Full=Vitronectin V65 subunit: Contains: RecName: Full=Vitronectin V10 subunit: Contains: RecName: Full=Somatomedin-B: Flags: Precursor |
| 33 | 16 | 7 | 81.46 | 5.8 | 0.00E+00 | 2.52E+05 | 1.99E+05 | 150333.33 | 3.74E+06 | 4.67E+06 | 4.54E+06 | 4316666.7 | 28.71 | P02763.1 | RecName: Full=Alpha-1-acid glycoprotein 1: Short=AGP 1: AltName: Full=Orosomucoid-1: Short=OMD 1: Flags: Precursor |
| 34 | 20 | 6 | 80.1 | 22.8 | 1.32E+06 | 0.00E+00 | 1.11E+06 | 810000 | 4.00E+06 | 5.97E+06 | 1.13E+07 | 7090000 | 8.75 | P19652.2 | RecName: Full=Alpha-1-acid glycoprotein 2: Short=AGP 2: AltName: Full=Orosomucoid-2: Short=OMD 2: Flags: Precursor |
| 35 | 37 | 6 | 102.86 | 31.3 | 9.30E+06 | 1.21E+07 | 7.83E+06 | 9743333.3 | 7.88E+06 | 7.87E+06 | 1.33E+07 | 9683333.3 | 0.99 | P04003.2 | RecName: Full=C4b-binding protein alpha chain: Short=C4bp: AltName: Full=Proline-rich protein: Short=PRP: Flags: Precursor |
| 36 | 84 | 6 | 100.97 | 79.2 | 4.34E+07 | 5.29E+07 | 4.92E+07 | 48500000 | 3.57E+07 | 7.80E+07 | 5.89E+07 | 57533333 | 1.19 | P10909.1 | RecName: Full=Clusterin: AltName: Full=Aging-associated gene 4 protein: AltName: Full=Apolipoprotein J: Short=Apo-J: AltName: Full=Complement cytolysis inhibitor: Short=CLI: AltName: Full=Complement-associated protein SP-40,40: AltName: Full=Ku70-binding protein 1: AltName: Full=NA1/NA2: AltName: Full=Testosterone-repressed prostate message 2: Short=TRPM-2: Contains: RecName: Full=Clusterin beta chain: AltName: Full=ApoJalpha: AltName: Full=Complement cytolysis inhibitor a chain: Contains: RecName: Full=Clusterin alpha chain: AltName: Full=ApoJbeta: AltName: Full=Complement cytolysis inhibitor b chain: Flags: Precursor |
| 37 | 20 | 6 | 98.79 | 28.1 | 6.67E+05 | 1.25E+06 | 1.81E+06 | 1242333.3 | 3.32E+06 | 2.23E+06 | 2.76E+06 | 2770000 | 2.23 | Q14624.4 | RecName: Full=Inter-alpha-trypsin inhibitor heavy chain H4: Short=ITI heavy chain H4: Short=ITI-HC4: Short=Inter-alpha-inhibitor heavy chain 4: AltName: Full=Inter-alpha-trypsin inhibitor family heavy chain-related protein: Short=IHRP: AltName: Full=Plasma kallikrein sensitive glycoprotein 120: Short=Gp120: Short=PK-120: Contains: RecName: Full=70 kDa inter-alpha-trypsin inhibitor heavy chain H4: Contains: RecName: Full=35 kDa inter-alpha-trypsin inhibitor heavy chain H4: Flags: Precursor |
| 38 | 19 | 6 | 95.94 | 14.6 | 6.59E+05 | 6.93E+05 | 7.32E+05 | 694666.67 | 4.41E+06 | 2.60E+06 | 1.11E+07 | 6036666.7 | 8.69 | P02751.4 | RecName: Full=Fibronectin: Short=FN: AltName: Full=Cold-insoluble globulin: Short=CIG: Contains: RecName: Full=Anastellin: Contains: RecName: Full=Ugl-Y1: Contains: RecName: Full=Ugl-Y2: Contains: RecName: Full=Ugl-Y3: Flags: Precursor |
| 39 | 36 | 6 | 95.42 | 19.2 | 4.68E+06 | 1.98E+06 | 1.60E+07 | 7553333.3 | 1.34E+07 | 1.70E+07 | 2.90E+07 | 19800000 | 2.62 | P02749.3 | RecName: Full=Beta-2-glycoprotein 1: AltName: Full=APC inhibitor: AltName: Full=Activated protein C-binding protein: AltName: Full=Anticardiolipin cofactor: AltName: Full=Apolipoprotein H: Short=Apo-H: AltName: Full=Beta-2-glycoprotein I: Short=B2GPI: Short=Beta(2)GPI: Flags: Precursor |
| 40 | 10 | 6 | 87.92 | 15.9 | 0.00E+00 | 0.00E+00 | 1.52E+05 | 50666.667 | 5.32E+05 | 4.17E+06 | 4.08E+06 | 2927333.3 | 57.78 | P0CG05.1 | RecName: Full=Ig lambda-2 chain C regions |
| 41 | 18 | 6 | 75.86 | 11.5 | 6.22E+05 | 0.00E+00 | 0.00E+00 | 207333.33 | 6.50E+06 | 5.69E+06 | 5.36E+06 | 5850000 | 28.22 | P25311.2 | RecName: Full=Zinc-alpha-2-glycoprotein: Short=Zn-alpha-2-GP: Short=Zn-alpha-2-glycoprotein: Flags: Precursor |
| 42 | 15 | 6 | 73.75 | 14.8 | 0.00E+00 | 0.00E+00 | 0.00E+00 | 0 | 3.44E+06 | 4.06E+06 | 3.06E+06 | 3520000 | #DIV/0! | P05155.2 | RecName: Full=Plasma protease C1 inhibitor: Short=C1 Inh: Short=C1Inh: AltName: Full=C1 esterase inhibitor: AltName: Full=C1-inhibiting factor: AltName: Full=Serpin G1: Flags: Precursor |
| 43 | 38 | 5 | 73.61 | 49.2 | 1.68E+07 | 1.48E+07 | 1.43E+07 | 15300000 | 2.75E+06 | 1.73E+06 | 3.49E+06 | 2656666.7 | 0.17 | P00734.2 | RecName: Full=Prothrombin: AltName: Full=Coagulation factor II: Contains: RecName: Full=Activation peptide fragment 1: Contains: RecName: Full=Activation peptide fragment 2: Contains: RecName: Full=Thrombin light chain: Contains: RecName: Full=Thrombin heavy chain: Flags: Precursor |
| 44 | 23 | 4 | 71.38 | 8 | 3.80E+06 | 2.69E+06 | 2.32E+06 | 2936666.7 | 1.25E+07 | 1.17E+07 | 1.42E+07 | 12800000 | 4.36 | P06727.3 | RecName: Full=Apolipoprotein A-IV: Short=Apo-AIV: Short=ApoA-IV: AltName: Full=Apolipoprotein A4: Flags: Precursor |
| 45 | 13 | 4 | 70.74 | 10.4 | 4.55E+05 | 3.38E+06 | 1.62E+06 | 1818333.3 | 4.28E+06 | 4.48E+06 | 1.67E+07 | 8486666.7 | 4.67 | P06396.1 | RecName: Full=Gelsolin: AltName: Full=AGEL: AltName: Full=Actin-depolymerizing factor: Short=ADF: AltName: Full=Brevin: Flags: Precursor |
| 46 | 16 | 4 | 52.19 | 11 | 1.48E+06 | 8.83E+05 | 3.41E+05 | 901333.33 | 2.76E+06 | 9.73E+05 | 3.21E+06 | 2314333.3 | 2.57 | P01011.2 | RecName: Full=Alpha-1-antichymotrypsin: Short=ACT: AltName: Full=Cell growth-inhibiting gene 24/25 protein: AltName: Full=Serpin A3: Contains: RecName: Full=Alpha-1-antichymotrypsin His-Pro-less: Flags: Precursor |
| 47 | 3 | 3 | 20.06 | 10.4 | 5.76E+05 | 1.26E+06 | 0.00E+00 | 612000 | 1.73E+08 | 2.06E+08 | 0.00E+00 | 126333333 | 206.43 | P69905.2 | RecName: Full=Hemoglobin subunit alpha: AltName: Full=Alpha-globin: AltName: Full=Hemoglobin alpha chaingi\|57013851\|sp\|P69906.2\|HBA_PANPA RecName: Full=Hemoglobin subunit alpha: AltName: Full=Alpha-globin: AltName: Full=Hemoglobin alpha chaingi\|57013852\|sp\|P69907.2\|HBA_PANTR RecName: Full=Hemoglobin subunit alpha: AltName: Full=Alpha-globin: AltName: Full=Hemoglobin alpha chain |
| 48 | 15 | 3 | 59.38 | 16.7 | 2.56E+06 | 4.83E+06 | 2.94E+06 | 3443333.3 | 3.98E+06 | 2.78E+06 | 7.89E+05 | 2516333.3 | 0.73 | P19823.2 | RecName: Full=Inter-alpha-trypsin inhibitor heavy chain H2: Short=ITI heavy chain H2: Short=ITI-HC2: Short=Inter-alpha-inhibitor heavy chain 2: AltName: Full=Inter-alpha-trypsin inhibitor complex component II: AltName: Full=Serum-derived hyaluronan-associated protein: Short=SHAP: Flags: Precursor |
| 49 | 9 | 3 | 43.69 | 24 | 1.04E+06 | 7.15E+05 | 0.00E+00 | 585000 | 1.47E+06 | 5.62E+05 | 4.97E+05 | 843000 | 1.44 | P00747.2 | RecName: Full=Plasminogen: Contains: RecName: Full=Plasmin heavy chain A: Contains: RecName: Full=Activation peptide: Contains: RecName: Full=Angiostatin: Contains: RecName: Full=Plasmin heavy chain A, short form: Contains: RecName: Full=Plasmin light chain B: Flags: Precursor |
| 50 | 5 | 3 | 42.86 | 8.5 | 0.00E+00 | 0.00E+00 | 0.00E+00 | 0 | 6.26E+05 | 2.51E+06 | 2.28E+05 | 1121333.3 | #DIV/0! | P04196.1 | RecName: Full=Histidine-rich glycoprotein: AltName: Full=Histidine-proline-rich glycoprotein: Short=HPRG: Flags: Precursor |
| 51 | 5 | 3 | 37.97 | 18.2 | 9.41E+05 | 0.00E+00 | 5.99E+05 | 513333.33 | 1.64E+05 | 0.00E+00 | 7.83E+05 | 315666.67 | 0.61 | P02760.1 | RecName: Full=Protein AMBP: Contains: RecName: Full=Alpha-1-microglobulin: Short=Protein HC: AltName: Full=Alpha-1 microglycoprotein: AltName: Full=Complex-forming glycoprotein heterogeneous in charge: Contains: RecName: Full=Inter-alpha-trypsin inhibitor light chain: Short=ITI-LC: AltName: Full=Bikunin: AltName: Full=EDC1: AltName: Full=HI-30: AltName: Full=Uronic-acid-rich protein: Contains: RecName: Full=Trypstatin: Flags: Precursor |
| 52 | 6 | 2 | 30.2 | 22.8 | 0.00E+00 | 0.00E+00 | 0.00E+00 | 0 | 1.30E+06 | 8.10E+05 | 0.00E+00 | 703333.33 | #DIV/0! | P18136.2 | RecName: Full=Ig kappa chain V-III region HIC: Flags: Precursor |
| 53 | 8 | 2 | 25.33 | 12.1 | 2.00E+07 | 1.94E+07 | 2.16E+07 | 20333333 | 9.33E+06 | 6.79E+07 | 4.79E+07 | 41710000 | 2.05 | P43652.1 | RecName: Full=Afamin: AltName: Full=Alpha-albumin: Short=Alpha-Alb: Flags: Precursor |
| 54 | 4 | 2 | 24.91 | 1.9 | 0.00E+00 | 0.00E+00 | 6.09E+04 | 20300 | 1.06E+05 | 1.38E+05 | 1.28E+05 | 124000 | 6.11 | P01591.4 | RecName: Full=Immunoglobulin J chain: Flags: Precursor |
| 55 | 10 | 2 | 24.13 | 19.1 | 5.09E+05 | 0.00E+00 | 5.92E+05 | 367000 | 4.14E+06 | 2.36E+05 | 4.65E+06 | 3008666.7 | 8.20 | P01625.2 | RecName: Full=Ig kappa chain V-IV region Len |
| 56 | 2 | 2 | 19.45 | 2.4 | 3.37E+05 | 0.00E+00 | 0.00E+00 | 112333.33 | 0.00E+00 | 0.00E+00 | 2.19E+05 | 73000 | 0.65 | P00761.1 | RecName: Full=Trypsin: Flags: Precursor |
| 57 | 3 | 2 | 18.25 | 5.7 | 0.00E+00 | 4.47E+05 | 5.20E+05 | 322333.33 | 0.00E+00 | 0.00E+00 | 1.26E+06 | 420000 | 1.30 | P01031.4 | RecName: Full=Complement C5: AltName: Full=C3 and PZP-like alpha-2-macroglobulin domain-containing protein 4: Contains: RecName: Full=Complement C5 beta chain: Contains: RecName: Full=Complement C5 alpha chain: Contains: RecName: Full=C5a anaphylatoxin: Contains: RecName: Full=Complement C5 alpha' chain: Flags: Precursor |
| 58 | 3 | 2 | 15.39 | 11.6 | 7.94E+05 | 7.15E+05 | 0.00E+00 | 503000 | 3.17E+04 | 0.00E+00 | 0.00E+00 | 10566.667 | 0.02 | P01766.1 | RecName: Full=Ig heavy chain V-III region BRO |
| 59 | 2 | 2 | 14.31 | 0.6 | 0.00E+00 | 0.00E+00 | 3.84E+05 | 128000 | 0.00E+00 | 0.00E+00 | 0.00E+00 | 0 | 0.00 | P05156.2 | RecName: Full=Complement factor I: AltName: Full=C3B/C4B inactivator: Contains: RecName: Full=Complement factor I heavy chain: Contains: RecName: Full=Complement factor I light chain: Flags: Precursor |
| 60 | 3 | 2 | 12.62 | 7.1 | 0.00E+00 | 0.00E+00 | 8.39E+05 | 279666.67 | 8.65E+05 | 0.00E+00 | 2.77E+06 | 1211666.7 | 4.33 | O43866.1 | RecName: Full=CD5 antigen-like: AltName: Full=CT-2: AltName: Full=IgM-associated peptide: AltName: Full=SP-alpha: Flags: Precursor |
| 61 | 2 | 2 | 11.96 | 4.7 | 0.00E+00 | 0.00E+00 | 5.69E+05 | 189666.67 | 1.31E+07 | 0.00E+00 | 0.00E+00 | 4366666.7 | 23.02 | P04207.2 | RecName: Full=Ig kappa chain V-III region CLL: AltName: Full=Rheumatoid factor: Flags: Precursor |
| 62 | 1 | 1 | 8.77 | 2.2 | 0.00E+00 | 0.00E+00 | 0.00E+00 | 0 | 0.00E+00 | 3.16E+05 | 0.00E+00 | 105333.33 | #DIV/0! | O75037.2 | RecName: Full=Kinesin-like protein KIF21B |
| 63 | 1 | 1 | 5.38 | 2.6 | 0.00E+00 | 0.00E+00 | 0.00E+00 | 0 | 2.34E+06 | 0.00E+00 | 0.00E+00 | 780000 | #DIV/0! | Q3SV33.1 | RecName: Full=Sulfite reductase [NADPH] hemoprotein beta-component: Short=SiR-HP: Short=SiRHP |
| 64 | 1 | 1 | 8.44 | 5.7 | 0.00E+00 | 0.00E+00 | 0.00E+00 | 0 | 0.00E+00 | 4.00E+04 | 0.00E+00 | 13333.333 | #DIV/0! | Q86VV4.2 | RecName: Full=Ran-binding protein 3-like |
| 65 | 5 | 1 | 24.82 | 38.8 | 0.00E+00 | 0.00E+00 | 0.00E+00 | 0 | 9.69E+07 | 9.14E+06 | 0.00E+00 | 35346667 | #DIV/0! | P32081.1 | RecName: Full=Cold shock protein CspB: AltName: Full=Major cold shock protein |
| 66 | 5 | 1 | 22.18 | 39.3 | 0.00E+00 | 0.00E+00 | 0.00E+00 | 0 | 4.88E+07 | 0.00E+00 | 0.00E+00 | 16266667 | #DIV/0! | P51777.1 | RecName: Full=Cold shock protein CspD |
| 67 | 7 | 1 | 18.89 | 4.9 | 1.55E+06 | 1.31E+06 | 1.31E+06 | 1390000 | 8.95E+05 | 1.21E+06 | 5.52E+06 | 2541666.7 | 1.83 | P27485.2 | RecName: Full=Retinol-binding protein 4: AltName: Full=Plasma retinol-binding protein: Short=PRBP: Short=RBP: Flags: Precursor |
| 68 | 8 | 1 | 18.77 | 7 | 1.74E+06 | 7.49E+05 | 3.80E+06 | 2096333.3 | 0.00E+00 | 2.15E+06 | 0.00E+00 | 716666.67 | 0.34 | P18978.1 | RecName: Full=Hemoglobin subunit alpha: AltName: Full=Alpha-globin: AltName: Full=Hemoglobin alpha chain |
| 69 | 1 | 1 | 18.4 | 3 | 0.00E+00 | 0.00E+00 | 0.00E+00 | 0 | 2.54E+05 | 0.00E+00 | 0.00E+00 | 84666.667 | #DIV/0! | Q5RAD0.1 | RecName: Full=Complement component C7: Flags: Precursor |
| 70 | 7 | 1 | 17.46 | 4.7 | 1.94E+06 | 1.05E+06 | 2.37E+06 | 1786666.7 | 2.53E+05 | 0.00E+00 | 0.00E+00 | 84333.333 | 0.05 | P07415.1 | RecName: Full=Hemoglobin subunit beta: AltName: Full=Beta-globin: AltName: Full=Hemoglobin beta chain |
| 71 | 5 | 1 | 16.14 | 1.2 | 0.00E+00 | 0.00E+00 | 0.00E+00 | 0 | 6.10E+05 | 7.10E+05 | 4.22E+05 | 580666.67 | #DIV/0! | P01029.3 | RecName: Full=Complement C4-B: Contains: RecName: Full=Complement C4 beta chain: Contains: RecName: Full=Complement C4 alpha chain: Contains: RecName: Full=C4a anaphylatoxin: Contains: RecName: Full=Complement C4 gamma chain: Flags: Precursor |
| 72 | 1 | 1 | 15.79 | 3.4 | 0.00E+00 | 0.00E+00 | 0.00E+00 | 0 | 8.34E+05 | 0.00E+00 | 0.00E+00 | 278000 | #DIV/0! | P19134.4 | RecName: Full=Serotransferrin: Short=Transferrin: AltName: Full=Beta-1 metal-binding globulin: AltName: Full=Siderophilin: Flags: Precursor |
| 73 | 1 | 1 | 14.86 | 2.7 | 3.71E+05 | 0.00E+00 | 0.00E+00 | 123666.67 | 0.00E+00 | 0.00E+00 | 0.00E+00 | 0 | 0.00 | Q9C6M5.1 | RecName: Full=AP2/ERF and B3 domain-containing transcription repressor TEM1: AltName: Full=Protein TEMPRANILLO 1: AltName: Full=RAV1-like ethylene-responsive transcription factor TEM1 |
| 74 | 2 | 1 | 14.65 | 3.8 | 4.71E+05 | 0.00E+00 | 0.00E+00 | 157000 | 1.45E+06 | 0.00E+00 | 0.00E+00 | 483333.33 | 3.08 | P08697.3 | RecName: Full=Alpha-2-antiplasmin: Short=Alpha-2-AP: AltName: Full=Alpha-2-plasmin inhibitor: Short=Alpha-2-PI: AltName: Full=Serpin F2: Flags: Precursor |
| 75 | 1 | 1 | 12.99 | 9.7 | 0.00E+00 | 0.00E+00 | 0.00E+00 | 0 | 0.00E+00 | 0.00E+00 | 1.29E+06 | 430000 | #DIV/0! | P06310.1 | RecName: Full=Ig kappa chain V-II region RPMI 6410: Flags: Precursor |
| 76 | 6 | 1 | 12.92 | 7.2 | 0.00E+00 | 1.80E+05 | 3.42E+05 | 174000 | 0.00E+00 | 2.68E+06 | 9.16E+05 | 1198666.7 | 6.89 | P80748.1 | RecName: Full=Ig lambda chain V-III region LOI |
| 77 | 2 | 1 | 12.87 | 6.9 | 1.01E+05 | 0.00E+00 | 0.00E+00 | 33666.667 | 0.00E+00 | 0.00E+00 | 6.50E+05 | 216666.67 | 6.44 | P35542.2 | RecName: Full=Serum amyloid A-4 protein: AltName: Full=Constitutively expressed serum amyloid A protein: Short=C-SAA: Flags: Precursor |
| 78 | 9 | 1 | 12.71 | 16.1 | 4.60E+06 | 2.72E+06 | 4.27E+06 | 3863333.3 | 0.00E+00 | 0.00E+00 | 0.00E+00 | 0 | 0.00 | P02656.1 | RecName: Full=Apolipoprotein C-III: Short=Apo-CIII: Short=ApoC-III: AltName: Full=Apolipoprotein C3: Flags: Precursor |
| 79 | 3 | 1 | 12.39 | 0.9 | 0.00E+00 | 1.73E+06 | 1.83E+06 | 1186666.7 | 2.20E+06 | 0.00E+00 | 0.00E+00 | 733333.33 | 0.62 | O94804.1 | RecName: Full=Serine/threonine-protein kinase 10: AltName: Full=Lymphocyte-oriented kinase |
| 80 | 1 | 1 | 11.67 | 11.6 | 0.00E+00 | 0.00E+00 | 0.00E+00 | 0 | 3.86E+05 | 0.00E+00 | 0.00E+00 | 128666.67 | #DIV/0! | P01700.1 | RecName: Full=Ig lambda chain V-I region HA |
| 81 | 1 | 1 | 11.53 | 3.4 | 8.07E+06 | 0.00E+00 | 0.00E+00 | 2690000 | 0.00E+00 | 0.00E+00 | 0.00E+00 | 0 | 0.00 | A7HJX5.1 | RecName: Full=Valine--tRNA ligase: AltName: Full=Valyl-tRNA synthetase: Short=ValRS |
| 82 | 4 | 1 | 11.45 | 1.7 | 9.42E+05 | 7.39E+05 | 7.65E+05 | 815333.33 | 9.73E+05 | 0.00E+00 | 0.00E+00 | 324333.33 | 0.40 | Q28HX4.1 | RecName: Full=Intraflagellar transport protein 57 homolog |
| 83 | 7 | 1 | 11.21 | 3.7 | 4.86E+06 | 4.87E+06 | 8.98E+06 | 6236666.7 | 0.00E+00 | 0.00E+00 | 0.00E+00 | 0 | 0.00 | Q62558.2 | RecName: Full=Haptoglobin: AltName: Full=Zonulin: Contains: RecName: Full=Haptoglobin alpha chain: Contains: RecName: Full=Haptoglobin beta chain: Flags: Precursor |
| 84 | 4 | 1 | 11.07 | 0 | 3.69E+05 | 4.61E+05 | 6.08E+05 | 479333.33 | 3.95E+05 | 0.00E+00 | 0.00E+00 | 131666.67 | 0.27 | Q9I7U4.3 | RecName: Full=Titin: AltName: Full=D-Titin: AltName: Full=Kettin |
| 85 | 1 | 1 | 10.48 | 8.3 | 0.00E+00 | 0.00E+00 | 0.00E+00 | 0 | 0.00E+00 | 0.00E+00 | 5.01E+04 | 16700 | #DIV/0! | C1DRQ7.1 | RecName: Full=Methylthioribose-1-phosphate isomerase: Short=M1Pi: Short=MTR-1-P isomerase: AltName: Full=S-methyl-5-thioribose-1-phosphate isomerase |
| 86 | 1 | 1 | 10.43 | 13.2 | 0.00E+00 | 0.00E+00 | 0.00E+00 | 0 | 3.39E+05 | 0.00E+00 | 0.00E+00 | 113000 | #DIV/0! | P59093.1 | RecName: Full=Uncharacterized protein C21orf99: AltName: Full=Cancer/testis antigen 85: Short=CT85 |
| 87 | 1 | 1 | 10.39 | 1.1 | 0.00E+00 | 0.00E+00 | 5.77E+05 | 192333.33 | 0.00E+00 | 0.00E+00 | 0.00E+00 | 0 | 0.00 | A3PGJ0.1 | RecName: Full=DNA-directed RNA polymerase subunit beta': Short=RNAP subunit beta': AltName: Full=RNA polymerase subunit beta': AltName: Full=Transcriptase subunit beta' |
| 88 | 1 | 1 | 10.31 | 4.5 | 0.00E+00 | 1.65E+06 | 0.00E+00 | 550000 | 0.00E+00 | 0.00E+00 | 0.00E+00 | 0 | 0.00 | Q0ABG0.1 | RecName: Full=50S ribosomal protein L6 |
| 89 | 3 | 1 | 10.07 | 0.5 | 0.00E+00 | 1.45E+06 | 3.11E+06 | 1520000 | 0.00E+00 | 0.00E+00 | 0.00E+00 | 0 | 0.00 | P28685.1 | RecName: Full=Contactin-2: AltName: Full=Axonin-1: Flags: Precursor |
| 90 | 1 | 1 | 10.02 | 2.3 | 0.00E+00 | 2.57E+06 | 0.00E+00 | 856666.67 | 0.00E+00 | 0.00E+00 | 0.00E+00 | 0 | 0.00 | Q0P5E6.1 | RecName: Full=Alpha-1-syntrophin |
| 91 | 1 | 1 | 10 | 13.8 | 0.00E+00 | 0.00E+00 | 0.00E+00 | 0 | 4.14E+05 | 0.00E+00 | 0.00E+00 | 138000 | #DIV/0! | B4U6R8.1 | RecName: Full=Deoxyuridine 5'-triphosphate nucleotidohydrolase: Short=dUTPase: AltName: Full=dUTP pyrophosphatase |
| 92 | 1 | 1 | 9.89 | 0.6 | 0.00E+00 | 4.71E+05 | 0.00E+00 | 157000 | 0.00E+00 | 0.00E+00 | 0.00E+00 | 0 | 0.00 | Q07630.1 | RecName: Full=RNA replication protein: AltName: Full=165 kDa protein: AltName: Full=ORF1 protein: Includes: RecName: Full=RNA-directed RNA polymerase: Includes: RecName: Full=Helicase |
| 93 | 3 | 1 | 9.88 | 7.8 | 4.38E+05 | 0.00E+00 | 0.00E+00 | 146000 | 7.01E+05 | 0.00E+00 | 1.72E+06 | 807000 | 5.53 | B7VIS1.1 | RecName: Full=UPF0325 protein VS_2356 |
| 94 | 4 | 1 | 9.77 | 1.7 | 2.09E+06 | 0.00E+00 | 0.00E+00 | 696666.67 | 2.65E+06 | 0.00E+00 | 1.78E+07 | 6816666.7 | 9.78 | A6NDB9.2 | RecName: Full=Paralemmin-3: Flags: Precursor |
| 95 | 1 | 1 | 9.64 | 0.5 | 0.00E+00 | 0.00E+00 | 6.61E+05 | 220333.33 | 0.00E+00 | 0.00E+00 | 0.00E+00 | 0 | 0.00 | O51568.1 | RecName: Full=Transcription-repair-coupling factor: Short=TRCF: AltName: Full=ATP-dependent helicase mfd |
| 96 | 1 | 1 | 9.62 | 2.9 | 0.00E+00 | 0.00E+00 | 5.99E+05 | 199666.67 | 0.00E+00 | 0.00E+00 | 0.00E+00 | 0 | 0.00 | Q92BQ6.1 | RecName: Full=RNA polymerase sigma factor RpoD: AltName: Full=Sigma-43 |
| 97 | 1 | 1 | 9.54 | 15.8 | 0.00E+00 | 0.00E+00 | 2.57E+05 | 85666.667 | 0.00E+00 | 0.00E+00 | 0.00E+00 | 0 | 0.00 | B2HIJ2.1 | RecName: Full=Uracil-DNA glycosylase: Short=UDG |
| 98 | 3 | 1 | 9.54 | 3.7 | 6.91E+05 | 0.00E+00 | 0.00E+00 | 230333.33 | 0.00E+00 | 0.00E+00 | 1.44E+06 | 480000 | 2.08 | P05090.1 | RecName: Full=Apolipoprotein D: Short=Apo-D: Short=ApoD: Flags: Precursor |
| 99 | 1 | 1 | 9.44 | 7.7 | 0.00E+00 | 0.00E+00 | 0.00E+00 | 0 | 2.76E+06 | 0.00E+00 | 0.00E+00 | 920000 | #DIV/0! | Q9HIR2.1 | RecName: Full=50S ribosomal protein L2P |
| 100 | 1 | 1 | 9.2 | 0.2 | 0.00E+00 | 0.00E+00 | 0.00E+00 | 0 | 0.00E+00 | 3.83E+06 | 0.00E+00 | 1276666.7 | #DIV/0! | Q54IX3.1 | RecName: Full=Probable polyketide synthase 26: Short=dipks26 |
| 101 | 1 | 1 | 9.17 | 1.9 | 0.00E+00 | 0.00E+00 | 0.00E+00 | 0 | 0.00E+00 | 2.47E+06 | 0.00E+00 | 823333.33 | #DIV/0! | A1B495.1 | RecName: Full=NADH-quinone oxidoreductase subunit D: AltName: Full=NADH dehydrogenase I, subunit 4: AltName: Full=NADH dehydrogenase I, subunit D: AltName: Full=NADH-quinone oxidoreductase subunit 4: Short=NQO4: AltName: Full=NDH-1, subunit 4: AltName: Full=NDH-1, subunit D |
| 102 | 5 | 1 | 9.15 | 14.9 | 4.00E+06 | 0.00E+00 | 0.00E+00 | 1333333.3 | 1.01E+07 | 1.04E+07 | 1.20E+07 | 10833333 | 8.13 | A9GUX6.1 | RecName: Full=NADH-quinone oxidoreductase subunit B 1: AltName: Full=NADH dehydrogenase I subunit B 1: AltName: Full=NDH-1 subunit B 1 |
| 103 | 1 | 1 | 9.11 | 3.1 | 0.00E+00 | 0.00E+00 | 0.00E+00 | 0 | 0.00E+00 | 0.00E+00 | 4.10E+03 | 1366.6667 | #DIV/0! | Q5GH72.1 | RecName: Full=XK-related protein 7 |
| 104 | 3 | 1 | 9.1 | 2.4 | 0.00E+00 | 0.00E+00 | 0.00E+00 | 0 | 0.00E+00 | 4.94E+06 | 2.18E+06 | 2373333.3 | #DIV/0! | P0ACP6.1 | RecName: Full=HTH-type transcriptional regulator GntR: AltName: Full=Gluconate utilization system GNT-I transcriptional repressorgi\|82581670\|sp\|P0ACP5.1\|GNTR_ECOLI RecName: Full=HTH-type transcriptional regulator GntR: AltName: Full=Gluconate utilization system GNT-I transcriptional repressor |
| 105 | 2 | 1 | 9.03 | 1 | 4.35E+05 | 0.00E+00 | 6.52E+05 | 362333.33 | 0.00E+00 | 0.00E+00 | 0.00E+00 | 0 | 0.00 | O43345.1 | RecName: Full=Zinc finger protein 208: AltName: Full=Zinc finger protein 91-like |
| 106 | 1 | 1 | 8.86 | 3.6 | 0.00E+00 | 0.00E+00 | 0.00E+00 | 0 | 2.54E+05 | 0.00E+00 | 0.00E+00 | 84666.667 | #DIV/0! | O43374.2 | RecName: Full=Ras GTPase-activating protein 4: AltName: Full=Calcium-promoted Ras inactivator: AltName: Full=Ras p21 protein activator 4: AltName: Full=RasGAP-activating-like protein 2 |
| 107 | 5 | 1 | 8.81 | 14.2 | 0.00E+00 | 0.00E+00 | 0.00E+00 | 0 | 7.58E+06 | 7.33E+06 | 2.09E+06 | 5666666.7 | #DIV/0! | Q8RBK6.1 | RecName: Full=Phosphoribosylformylglycinamidine synthase 1: AltName: Full=Phosphoribosylformylglycinamidine synthase I: Short=FGAM synthase I |
| 108 | 2 | 1 | 8.75 | 2.3 | 0.00E+00 | 0.00E+00 | 7.84E+05 | 261333.33 | 6.23E+05 | 0.00E+00 | 0.00E+00 | 207666.67 | 0.79 | D2NT89.2 | RecName: Full=AAA ATPase forming ring-shaped complexes: Short=ARC |
| 109 | 1 | 1 | 8.74 | 18.6 | 0.00E+00 | 0.00E+00 | 0.00E+00 | 0 | 4.26E+05 | 0.00E+00 | 0.00E+00 | 142000 | #DIV/0! | O07006.1 | RecName: Full=Phenolic acid decarboxylase PadC: Short=PAD |
| 110 | 1 | 1 | 8.71 | 2.7 | 0.00E+00 | 0.00E+00 | 0.00E+00 | 0 | 0.00E+00 | 0.00E+00 | 4.27E+06 | 1423333.3 | #DIV/0! | Q5HPJ0.1 | RecName: Full=Aconitate hydratase: Short=Aconitase: AltName: Full=Citrate hydro-lyase |
| 111 | 1 | 1 | 8.68 | 5.1 | 0.00E+00 | 0.00E+00 | 0.00E+00 | 0 | 0.00E+00 | 0.00E+00 | 4.61E+06 | 1536666.7 | #DIV/0! | Q8VV84.1 | RecName: Full=60 kDa chaperonin: AltName: Full=GroEL protein: AltName: Full=Protein Cpn60 |
| 112 | 2 | 1 | 8.64 | 0.4 | 0.00E+00 | 0.00E+00 | 0.00E+00 | 0 | 0.00E+00 | 1.37E+06 | 2.70E+06 | 1356666.7 | #DIV/0! | Q8IYW2.3 | RecName: Full=Tetratricopeptide repeat protein 40 |
| 113 | 1 | 1 | 8.61 | 3 | 0.00E+00 | 0.00E+00 | 0.00E+00 | 0 | 0.00E+00 | 0.00E+00 | 1.47E+05 | 49000 | #DIV/0! | Q5RFM4.3 | RecName: Full=Centromere/kinetochore protein zw10 homolog |
| 114 | 5 | 1 | 8.58 | 10.9 | 3.39E+06 | 3.04E+06 | 2.72E+06 | 3050000 | 0.00E+00 | 2.71E+06 | 2.79E+06 | 1833333.3 | 0.60 | O57818.1 | RecName: Full=Uncharacterized HTH-type transcriptional regulator PH0045 |
| 115 | 2 | 1 | 8.57 | 63 | 0.00E+00 | 0.00E+00 | 8.98E+06 | 2993333.3 | 0.00E+00 | 0.00E+00 | 4.74E+06 | 1580000 | 0.53 | P01542.2 | RecName: Full=Crambin |
| 116 | 4 | 1 | 8.57 | 5.4 | 0.00E+00 | 8.65E+06 | 7.96E+06 | 5536666.7 | 0.00E+00 | 8.44E+06 | 1.57E+07 | 8046666.7 | 1.45 | P62258.1 | RecName: Full=14-3-3 protein epsilon: Short=14-3-3Egi\|60391192\|sp\|P62259.1\|1433E_MOUSE RecName: Full=14-3-3 protein epsilon: Short=14-3-3Egi\|61216932\|sp\|P62260.1\|1433E_RAT RecName: Full=14-3-3 protein epsilon: Short=14-3-3E: AltName: Full=Mitochondrial import stimulation factor L subunit: Short=MSF Lgi\|71153779\|sp\|P62261.1\|1433E_BOVIN RecName: Full=14-3-3 protein epsilon: Short=14-3-3Egi\|71153780\|sp\|P62262.1\|1433E_SHEEP RecName: Full=14-3-3 protein epsilon: Short=14-3-3E: AltName: Full=Protein kinase C inhibitor protein 1: Short=KCIP-1gi\|82197924\|sp\|Q5ZMT0.1\|1433E_CHICK RecName: Full=14-3-3 protein epsilon: Short=14-3-3E |
| 117 | 3 | 1 | 8.56 | 1.2 | 5.30E+05 | 0.00E+00 | 0.00E+00 | 176666.67 | 0.00E+00 | 1.04E+06 | 1.27E+06 | 770000 | 4.36 | O74458.1 | RecName: Full=Transcription factor tau subunit sfc4: AltName: Full=TFIIIC subunit sfc4: AltName: Full=Transcription factor C subunit 4 |
| 118 | 1 | 1 | 8.56 | 3.9 | 0.00E+00 | 0.00E+00 | 0.00E+00 | 0 | 0.00E+00 | 8.65E+03 | 0.00E+00 | 2883.3333 | #DIV/0! | P46557.1 | RecName: Full=Matrix non-peptidase homolog 1: Flags: Precursor |
| 119 | 3 | 1 | 8.56 | 18.2 | 0.00E+00 | 0.00E+00 | 0.00E+00 | 0 | 3.57E+05 | 1.35E+05 | 7.60E+04 | 189333.33 | #DIV/0! | Q9BYA7.2 | RecName: Full=Putative proline dehydrogenase-like protein |
| 120 | 1 | 1 | 8.5 | 8.9 | 0.00E+00 | 0.00E+00 | 0.00E+00 | 0 | 2.20E+06 | 0.00E+00 | 0.00E+00 | 733333.33 | #DIV/0! | Q7TTU9.1 | RecName: Full=Tryptophan--tRNA ligase: AltName: Full=Tryptophanyl-tRNA synthetase: Short=TrpRS |
| 121 | 1 | 1 | 8.49 | 5.2 | 0.00E+00 | 0.00E+00 | 0.00E+00 | 0 | 2.46E+05 | 0.00E+00 | 0.00E+00 | 82000 | #DIV/0! | Q1MPL6.1 | RecName: Full=Aspartyl/glutamyl-tRNA(Asn/Gln) amidotransferase subunit B: Short=Asp/Glu-ADT subunit B |
| 122 | 1 | 1 | 8.45 | 2.3 | 0.00E+00 | 1.05E+06 | 0.00E+00 | 350000 | 0.00E+00 | 0.00E+00 | 0.00E+00 | 0 | 0.00 | Q9UJX2.3 | RecName: Full=Cell division cycle protein 23 homolog: AltName: Full=Anaphase-promoting complex subunit 8: Short=APC8: AltName: Full=Cyclosome subunit 8 |
| 123 | 1 | 1 | 8.44 | 0.5 | 3.66E+05 | 0.00E+00 | 0.00E+00 | 122000 | 0.00E+00 | 0.00E+00 | 0.00E+00 | 0 | 0.00 | P55383.1 | RecName: Full=Uncharacterized protein y4cA |
| 124 | 1 | 1 | 8.41 | 0.9 | 0.00E+00 | 0.00E+00 | 0.00E+00 | 0 | 5.13E+06 | 0.00E+00 | 0.00E+00 | 1710000 | #DIV/0! | Q13523.3 | RecName: Full=Serine/threonine-protein kinase PRP4 homolog: AltName: Full=PRP4 kinase: AltName: Full=PRP4 pre-mRNA-processing factor 4 homolog |
| 125 | 1 | 1 | 8.38 | 2.3 | 0.00E+00 | 0.00E+00 | 0.00E+00 | 0 | 0.00E+00 | 0.00E+00 | 3.47E+05 | 115666.67 | #DIV/0! | Q87RJ4.1 | RecName: Full=DNA ligase: AltName: Full=Polydeoxyribonucleotide synthase [NAD(+)] |
| 126 | 6 | 1 | 8.31 | 34.8 | 2.04E+06 | 2.64E+06 | 6.14E+06 | 3606666.7 | 7.16E+06 | 6.70E+06 | 6.70E+06 | 6853333.3 | 1.90 | C4L2G6.1 | RecName: Full=Putative membrane protein insertion efficiency factor |
| 127 | 1 | 1 | 8.31 | 1.3 | 0.00E+00 | 0.00E+00 | 0.00E+00 | 0 | 1.51E+06 | 0.00E+00 | 0.00E+00 | 503333.33 | #DIV/0! | Q7TQ07.2 | RecName: Full=DNA polymerase nu |
| 128 | 3 | 1 | 8.29 | 0.3 | 0.00E+00 | 0.00E+00 | 0.00E+00 | 0 | 7.81E+05 | 1.35E+06 | 2.26E+06 | 1463666.7 | #DIV/0! | Q6BNL1.2 | RecName: Full=Actin cytoskeleton-regulatory complex protein PAN1 |
| 129 | 2 | 1 | 8.28 | 8.7 | 0.00E+00 | 6.84E+05 | 7.74E+05 | 486000 | 0.00E+00 | 0.00E+00 | 0.00E+00 | 0 | 0.00 | P55361.1 | RecName: Full=Probable transcriptional regulator SyrB |
| 130 | 1 | 1 | 8.21 | 3 | 0.00E+00 | 0.00E+00 | 0.00E+00 | 0 | 0.00E+00 | 0.00E+00 | 4.67E+04 | 15566.667 | #DIV/0! | Q9Y4D2.3 | RecName: Full=Sn1-specific diacylglycerol lipase alpha: Short=DGL-alpha: AltName: Full=Neural stem cell-derived dendrite regulator |
| 131 | 2 | 1 | 8.17 | 0.3 | 0.00E+00 | 0.00E+00 | 0.00E+00 | 0 | 3.56E+05 | 0.00E+00 | 2.06E+06 | 805333.33 | #DIV/0! | P52746.4 | RecName: Full=Zinc finger protein 142: AltName: Full=HA4654 |
| 132 | 1 | 1 | 8.15 | 2.9 | 2.40E+05 | 0.00E+00 | 0.00E+00 | 80000 | 0.00E+00 | 0.00E+00 | 0.00E+00 | 0 | 0.00 | P42768.4 | RecName: Full=Wiskott-Aldrich syndrome protein: Short=WASp |
| 133 | 2 | 1 | 8.13 | 5.4 | 0.00E+00 | 0.00E+00 | 0.00E+00 | 0 | 1.98E+05 | 4.93E+05 | 0.00E+00 | 230333.33 | #DIV/0! | Q96AE4.3 | RecName: Full=Far upstream element-binding protein 1: Short=FBP: Short=FUSE-binding protein 1: AltName: Full=DNA helicase V: Short=hDH V |
| 134 | 1 | 1 | 8.08 | 1.9 | 0.00E+00 | 9.22E+05 | 0.00E+00 | 307333.33 | 0.00E+00 | 0.00E+00 | 0.00E+00 | 0 | 0.00 | Q6AE64.1 | RecName: Full=UDP-N-acetylglucosamine--N-acetylmuramyl-(pentapeptide) pyrophosphoryl-undecaprenol N-acetylglucosamine transferase: AltName: Full=Undecaprenyl-PP-MurNAc-pentapeptide-UDPGlcNAc GlcNAc transferase |
| 135 | 1 | 1 | 8.06 | 2.7 | 0.00E+00 | 0.00E+00 | 0.00E+00 | 0 | 1.73E+06 | 0.00E+00 | 0.00E+00 | 576666.67 | #DIV/0! | P10244.1 | RecName: Full=Myb-related protein B: Short=B-Myb: AltName: Full=Myb-like protein 2 |
| 136 | 1 | 1 | 8.05 | 0.4 | 0.00E+00 | 3.06E+05 | 0.00E+00 | 102000 | 0.00E+00 | 0.00E+00 | 0.00E+00 | 0 | 0.00 | Q9BYW2.3 | RecName: Full=Histone-lysine N-methyltransferase SETD2: AltName: Full=HIF-1: AltName: Full=Huntingtin yeast partner B: AltName: Full=Huntingtin-interacting protein 1: Short=HIP-1: AltName: Full=Huntingtin-interacting protein B: AltName: Full=Lysine N-methyltransferase 3A: AltName: Full=SET domain-containing protein 2: Short=hSET2: AltName: Full=p231HBP |
| 137 | 4 | 1 | 8.04 | 2.5 | 0.00E+00 | 3.56E+05 | 4.49E+05 | 268333.33 | 4.29E+05 | 0.00E+00 | 4.84E+05 | 304333.33 | 1.13 | P07629.2 | RecName: Full=Serum amyloid P-component: AltName: Full=Female protein: Short=FP: AltName: Full=SAP(FP): Flags: Precursor |
| 138 | 1 | 1 | 8.03 | 3.7 | 0.00E+00 | 0.00E+00 | 0.00E+00 | 0 | 0.00E+00 | 0.00E+00 | 4.29E+05 | 143000 | #DIV/0! | P35913.2 | RecName: Full=Rod cGMP-specific 3',5'-cyclic phosphodiesterase subunit beta: Short=GMP-PDE beta: Flags: Precursor |
| 139 | 1 | 1 | 8.02 | 3.7 | 0.00E+00 | 0.00E+00 | 0.00E+00 | 0 | 3.77E+04 | 0.00E+00 | 0.00E+00 | 12566.667 | #DIV/0! | Q86UP0.1 | RecName: Full=Cadherin-24: Flags: Precursor |
| 140 | 3 | 1 | 8.01 | 3.1 | 0.00E+00 | 0.00E+00 | 0.00E+00 | 0 | 8.06E+05 | 1.10E+06 | 0.00E+00 | 635333.33 | #DIV/0! | Q9NTN9.1 | RecName: Full=Semaphorin-4G: Flags: Precursor |
| 141 | 2 | 1 | 7.96 | 1.1 | 0.00E+00 | 4.71E+05 | 0.00E+00 | 157000 | 5.75E+05 | 0.00E+00 | 0.00E+00 | 191666.67 | 1.22 | P32316.2 | RecName: Full=Acetyl-CoA hydrolase: AltName: Full=Acetyl-CoA deacylase: Short=Acetyl-CoA acylase |
| 142 | 1 | 1 | 7.95 | 9.5 | 0.00E+00 | 0.00E+00 | 0.00E+00 | 0 | 0.00E+00 | 0.00E+00 | 1.87E+06 | 623333.33 | #DIV/0! | O60096.1 | RecName: Full=37S ribosomal protein S35, mitochondrial: Flags: Precursor |
| 143 | 1 | 1 | 7.91 | 4.2 | 0.00E+00 | 0.00E+00 | 0.00E+00 | 0 | 0.00E+00 | 0.00E+00 | 2.15E+06 | 716666.67 | #DIV/0! | A3LQ86.2 | RecName: Full=Ribosome biogenesis protein YTM1 |
| 144 | 1 | 1 | 7.89 | 1 | 0.00E+00 | 0.00E+00 | 0.00E+00 | 0 | 0.00E+00 | 2.73E+06 | 0.00E+00 | 910000 | #DIV/0! | A4YXQ9.1 | RecName: Full=Glycine dehydrogenase [decarboxylating]: AltName: Full=Glycine cleavage system P-protein: AltName: Full=Glycine decarboxylase |
| 145 | 1 | 1 | 7.88 | 1.7 | 0.00E+00 | 0.00E+00 | 6.99E+05 | 233000 | 0.00E+00 | 0.00E+00 | 0.00E+00 | 0 | 0.00 | A6NNM3.3 | RecName: Full=RIMS-binding protein 3B: Short=RIM-BP3.B: AltName: Full=RIMS-binding protein 3.2: Short=RIM-BP3.2 |
| 146 | 1 | 1 | 7.86 | 1.6 | 0.00E+00 | 0.00E+00 | 0.00E+00 | 0 | 0.00E+00 | 0.00E+00 | 2.18E+06 | 726666.67 | #DIV/0! | Q42191.2 | RecName: Full=Mitochondrial inner membrane protein OXA1: AltName: Full=Oxidase assembly 1 protein: Short=AtOXA1: Flags: Precursor |
| 147 | 1 | 1 | 7.86 | 0.1 | 0.00E+00 | 0.00E+00 | 0.00E+00 | 0 | 3.41E+06 | 0.00E+00 | 0.00E+00 | 1136666.7 | #DIV/0! | Q8NF91.4 | RecName: Full=Nesprin-1: AltName: Full=Enaptin: AltName: Full=Myocyte nuclear envelope protein 1: Short=Myne-1: AltName: Full=Nuclear envelope spectrin repeat protein 1: AltName: Full=Synaptic nuclear envelope protein 1: Short=Syne-1 |
| 148 | 1 | 1 | 7.83 | 1.9 | 0.00E+00 | 0.00E+00 | 0.00E+00 | 0 | 0.00E+00 | 0.00E+00 | 6.49E+05 | 216333.33 | #DIV/0! | P02457.3 | RecName: Full=Collagen alpha-1(I) chain: AltName: Full=Alpha-1 type I collagen: Flags: Precursor |
| 149 | 1 | 1 | 7.81 | 5.3 | 0.00E+00 | 0.00E+00 | 0.00E+00 | 0 | 0.00E+00 | 0.00E+00 | 4.28E+05 | 142666.67 | #DIV/0! | B1YIT8.1 | RecName: Full=UDP-N-acetylmuramoylalanine--D-glutamate ligase: AltName: Full=D-glutamic acid-adding enzyme: AltName: Full=UDP-N-acetylmuramoyl-L-alanyl-D-glutamate synthetase |
| 150 | 1 | 1 | 7.81 | 2.9 | 0.00E+00 | 0.00E+00 | 0.00E+00 | 0 | 0.00E+00 | 0.00E+00 | 6.01E+05 | 200333.33 | #DIV/0! | Q7V051.1 | RecName: Full=2,3-bisphosphoglycerate-independent phosphoglycerate mutase: Short=BPG-independent PGAM: Short=Phosphoglyceromutase: Short=iPGM |
| 151 | 2 | 1 | 7.81 | 3 | 0.00E+00 | 0.00E+00 | 5.40E+05 | 180000 | 0.00E+00 | 0.00E+00 | 2.02E+06 | 673333.33 | 3.74 | Q8IXQ6.2 | RecName: Full=Poly [ADP-ribose] polymerase 9: Short=PARP-9: AltName: Full=ADP-ribosyltransferase diphtheria toxin-like 9: Short=ARTD9: AltName: Full=B aggressive lymphoma protein |
| 152 | 1 | 1 | 7.8 | 4.9 | 0.00E+00 | 0.00E+00 | 0.00E+00 | 0 | 5.31E+05 | 0.00E+00 | 0.00E+00 | 177000 | #DIV/0! | Q68DY1.2 | RecName: Full=Zinc finger protein 626 |
| 153 | 1 | 1 | 7.74 | 0.8 | 0.00E+00 | 0.00E+00 | 1.91E+06 | 636666.67 | 0.00E+00 | 0.00E+00 | 0.00E+00 | 0 | 0.00 | P22516.1 | RecName: Full=ATP-dependent RNA helicase CHL1: AltName: Full=Chromosome loss protein 1: AltName: Full=Chromosome transmission fidelity protein 1 |
| 154 | 1 | 1 | 7.73 | 5.1 | 0.00E+00 | 7.06E+06 | 0.00E+00 | 2353333.3 | 0.00E+00 | 0.00E+00 | 0.00E+00 | 0 | 0.00 | F2Z699.1 | RecName: Full=External alternative NADH-ubiquinone oxidoreductase, mitochondrial: AltName: Full=External alternative NADH dehydrogenase: AltName: Full=NADH:ubiquinone reductase (non-electrogenic): Flags: Precursor |
| 155 | 1 | 1 | 7.7 | 0.7 | 0.00E+00 | 0.00E+00 | 0.00E+00 | 0 | 0.00E+00 | 4.65E+06 | 0.00E+00 | 1550000 | #DIV/0! | Q04217.1 | RecName: Full=Probable ATP-dependent RNA helicase DHR1: AltName: Full=DEAH box RNA helicase DHR1: AltName: Full=Extracellular mutant protein 16 |
| 156 | 1 | 1 | 7.68 | 0.6 | 0.00E+00 | 0.00E+00 | 5.57E+05 | 185666.67 | 0.00E+00 | 0.00E+00 | 0.00E+00 | 0 | 0.00 | O60303.4 | RecName: Full=Uncharacterized protein KIAA0556 |
| 157 | 1 | 1 | 7.6 | 2.5 | 0.00E+00 | 0.00E+00 | 0.00E+00 | 0 | 0.00E+00 | 0.00E+00 | 1.48E+06 | 493333.33 | #DIV/0! | O14133.3 | RecName: Full=Arginyl-tRNA--protein transferase 1: Short=Arginyltransferase 1: Short=R-transferase 1: AltName: Full=Arginine-tRNA--protein transferase 1 |
| 158 | 1 | 1 | 7.59 | 1 | 0.00E+00 | 0.00E+00 | 0.00E+00 | 0 | 0.00E+00 | 1.47E+06 | 0.00E+00 | 490000 | #DIV/0! | C3PFR3.1 | RecName: Full=ATP synthase subunit alpha: AltName: Full=ATP synthase F1 sector subunit alpha: AltName: Full=F-ATPase subunit alpha |
| 159 | 1 | 1 | 7.58 | 8.1 | 0.00E+00 | 0.00E+00 | 0.00E+00 | 0 | 0.00E+00 | 0.00E+00 | 2.28E+06 | 760000 | #DIV/0! | O00170.2 | RecName: Full=AH receptor-interacting protein: Short=AIP: AltName: Full=Aryl-hydrocarbon receptor-interacting protein: AltName: Full=HBV X-associated protein 2: Short=XAP-2: AltName: Full=Immunophilin homolog ARA9 |
| 160 | 1 | 1 | 7.57 | 0.6 | 3.02E+05 | 0.00E+00 | 0.00E+00 | 100666.67 | 0.00E+00 | 0.00E+00 | 0.00E+00 | 0 | 0.00 | C3PH19.1 | RecName: Full=Translation initiation factor IF-2 |
| 161 | 1 | 1 | 7.56 | 8 | 0.00E+00 | 0.00E+00 | 0.00E+00 | 0 | 7.74E+06 | 0.00E+00 | 0.00E+00 | 2580000 | #DIV/0! | Q7Z6M1.1 | RecName: Full=Rab9 effector protein with kelch motifs: AltName: Full=40 kDa Rab9 effector protein: AltName: Full=p40 |
| 162 | 1 | 1 | 7.52 | 3.6 | 5.97E+05 | 0.00E+00 | 0.00E+00 | 199000 | 0.00E+00 | 0.00E+00 | 0.00E+00 | 0 | 0.00 | Q8YQ88.1 | RecName: Full=Putative ABC transporter ATP-binding protein alr3946 |
| 163 | 1 | 1 | 7.51 | 5.7 | 0.00E+00 | 0.00E+00 | 0.00E+00 | 0 | 0.00E+00 | 2.38E+06 | 0.00E+00 | 793333.33 | #DIV/0! | Q03924.5 | RecName: Full=Zinc finger protein 117: AltName: Full=Provirus-linked krueppel: Short=h-PLK: AltName: Full=Zinc finger protein HPF9 |
| 164 | 1 | 1 | 7.51 | 1.3 | 0.00E+00 | 0.00E+00 | 0.00E+00 | 0 | 0.00E+00 | 1.01E+06 | 0.00E+00 | 336666.67 | #DIV/0! | Q0AC83.1 | RecName: Full=LPS-assembly protein LptD: AltName: Full=Organic solvent tolerance protein: Flags: Precursor |
| 165 | 1 | 1 | 7.51 | 1.3 | 2.97E+07 | 0.00E+00 | 0.00E+00 | 9900000 | 0.00E+00 | 0.00E+00 | 0.00E+00 | 0 | 0.00 | Q5FWF4.2 | RecName: Full=DNA annealing helicase and endonuclease ZRANB3: AltName: Full=Annealing helicase 2: Short=AH2: AltName: Full=Zinc finger Ran-binding domain-containing protein 3: Includes: RecName: Full=DNA annealing helicase ZRANB3: Includes: RecName: Full=Endonuclease ZRANB3 |
| 166 | 1 | 1 | 7.48 | 1.3 | 0.00E+00 | 0.00E+00 | 0.00E+00 | 0 | 0.00E+00 | 0.00E+00 | 5.18E+06 | 1726666.7 | #DIV/0! | Q68CP9.2 | RecName: Full=AT-rich interactive domain-containing protein 2: Short=ARID domain-containing protein 2: AltName: Full=BRG1-associated factor 200: Short=BAF200: AltName: Full=Zinc finger protein with activation potential: AltName: Full=Zipzap/p200 |
| 167 | 1 | 1 | 7.48 | 4.4 | 0.00E+00 | 0.00E+00 | 0.00E+00 | 0 | 7.79E+06 | 0.00E+00 | 0.00E+00 | 2596666.7 | #DIV/0! | Q8J0D2.1 | RecName: Full=Oligoxyloglucan reducing end-specific cellobiohydrolase: Short=OXG-RCBH: Flags: Precursor |
| 168 | 1 | 1 | 7.41 | 0.7 | 0.00E+00 | 0.00E+00 | 1.07E+06 | 356666.67 | 0.00E+00 | 0.00E+00 | 0.00E+00 | 0 | 0.00 | Q72KS4.1 | RecName: Full=Lon protease 1: AltName: Full=ATP-dependent protease La 1 |
| 169 | 1 | 1 | 7.4 | 7 | 0.00E+00 | 0.00E+00 | 0.00E+00 | 0 | 0.00E+00 | 0.00E+00 | 1.41E+06 | 470000 | #DIV/0! | Q2G3S9.1 | RecName: Full=Trigger factor: Short=TF: AltName: Full=PPIase |
| 170 | 1 | 1 | 7.38 | 6.5 | 6.02E+06 | 0.00E+00 | 0.00E+00 | 2006666.7 | 0.00E+00 | 0.00E+00 | 0.00E+00 | 0 | 0.00 | Q6VVB1.2 | RecName: Full=E3 ubiquitin-protein ligase NHLRC1: AltName: Full=Malin: AltName: Full=NHL repeat-containing protein 1 |
| 171 | 1 | 1 | 7.37 | 11.3 | 0.00E+00 | 0.00E+00 | 0.00E+00 | 0 | 0.00E+00 | 1.73E+05 | 0.00E+00 | 57666.667 | #DIV/0! | Q13790.2 | RecName: Full=Apolipoprotein F: Short=Apo-F: AltName: Full=Lipid transfer inhibitor protein: Short=LTIP: Flags: Precursor |
| 172 | 1 | 1 | 7.37 | 2.9 | 0.00E+00 | 0.00E+00 | 6.82E+05 | 227333.33 | 0.00E+00 | 0.00E+00 | 0.00E+00 | 0 | 0.00 | Q5XHC1.1 | RecName: Full=UPF0602 protein C4orf47 homolog |
| 173 | 1 | 1 | 7.37 | 6.7 | 0.00E+00 | 0.00E+00 | 0.00E+00 | 0 | 0.00E+00 | 3.25E+06 | 0.00E+00 | 1083333.3 | #DIV/0! | Q9CBS7.1 | RecName: Full=3-oxoacyl-[acyl-carrier-protein] synthase 1: AltName: Full=Beta-ketoacyl-ACP synthase 1: Short=KAS 1 |
| 174 | 1 | 1 | 7.35 | 2.1 | 0.00E+00 | 0.00E+00 | 0.00E+00 | 0 | 0.00E+00 | 0.00E+00 | 5.77E+06 | 1923333.3 | #DIV/0! | O94525.1 | RecName: Full=Uncharacterized ribonuclease C609.01 |
| 175 | 1 | 1 | 7.33 | 2.6 | 0.00E+00 | 5.97E+06 | 0.00E+00 | 1990000 | 0.00E+00 | 0.00E+00 | 0.00E+00 | 0 | 0.00 | P51784.3 | RecName: Full=Ubiquitin carboxyl-terminal hydrolase 11: AltName: Full=Deubiquitinating enzyme 11: AltName: Full=Ubiquitin thioesterase 11: AltName: Full=Ubiquitin-specific-processing protease 11 |
| 176 | 1 | 1 | 7.32 | 0.8 | 1.00E+06 | 0.00E+00 | 0.00E+00 | 333333.33 | 0.00E+00 | 0.00E+00 | 0.00E+00 | 0 | 0.00 | Q8J1G4.1 | RecName: Full=Kinesin-like protein KIP1 |
| 177 | 2 | 1 | 7.3 | 3.8 | 0.00E+00 | 0.00E+00 | 0.00E+00 | 0 | 1.16E+06 | 1.03E+06 | 0.00E+00 | 730000 | #DIV/0! | P39820.3 | RecName: Full=Glutamate 5-kinase 1: AltName: Full=Gamma-glutamyl kinase 1: Short=GK 1 |
| 178 | 1 | 1 | 7.29 | 6.3 | 0.00E+00 | 0.00E+00 | 0.00E+00 | 0 | 0.00E+00 | 4.92E+05 | 0.00E+00 | 164000 | #DIV/0! | O94805.1 | RecName: Full=Actin-like protein 6B: AltName: Full=53 kDa BRG1-associated factor B: AltName: Full=Actin-related protein Baf53b: AltName: Full=ArpNalpha: AltName: Full=BRG1-associated factor 53B: Short=BAF53B |
| 179 | 1 | 1 | 7.28 | 1.6 | 0.00E+00 | 4.28E+05 | 0.00E+00 | 142666.67 | 0.00E+00 | 0.00E+00 | 0.00E+00 | 0 | 0.00 | Q98JM5.1 | RecName: Full=DNA polymerase IV 2: Short=Pol IV 2 |
| 180 | 1 | 1 | 7.25 | 3.3 | 0.00E+00 | 0.00E+00 | 0.00E+00 | 0 | 0.00E+00 | 1.97E+06 | 0.00E+00 | 656666.67 | #DIV/0! | P43284.1 | RecName: Full=Tryptophan synthase beta chain 2, chloroplastic: AltName: Full=Orange pericarp 2: Flags: Precursor |
| 181 | 3 | 1 | 7.25 | 6.7 | 5.50E+05 | 0.00E+00 | 7.35E+05 | 428333.33 | 0.00E+00 | 0.00E+00 | 1.58E+06 | 526666.67 | 1.23 | Q6P4M0.1 | RecName: Full=7-dehydrocholesterol reductase: Short=7-DHC reductase: AltName: Full=Sterol Delta(7)-reductase |
| 182 | 3 | 1 | 7.25 | 4 | 0.00E+00 | 5.99E+05 | 5.53E+05 | 384000 | 1.09E+06 | 0.00E+00 | 0.00E+00 | 363333.33 | 0.95 | Q6P5Z2.1 | RecName: Full=Serine/threonine-protein kinase N3: AltName: Full=Protein kinase PKN-beta: AltName: Full=Protein-kinase C-related kinase 3 |
| 183 | 1 | 1 | 7.23 | 2.6 | 0.00E+00 | 0.00E+00 | 0.00E+00 | 0 | 2.75E+05 | 0.00E+00 | 0.00E+00 | 91666.667 | #DIV/0! | O44199.1 | RecName: Full=DNA repair protein rad-50 |
| 184 | 1 | 1 | 7.23 | 3 | 0.00E+00 | 0.00E+00 | 0.00E+00 | 0 | 3.13E+06 | 0.00E+00 | 0.00E+00 | 1043333.3 | #DIV/0! | Q02928.1 | RecName: Full=Cytochrome P450 4A11: AltName: Full=20-hydroxyeicosatetraenoic acid synthase: Short=20-HETE synthase: AltName: Full=CYP4AII: AltName: Full=CYPIVA11: AltName: Full=Cytochrome P-450HK-omega: AltName: Full=Cytochrome P450HL-omega: AltName: Full=Fatty acid omega-hydroxylase: AltName: Full=Lauric acid omega-hydroxylase: Flags: Precursor |
| 185 | 3 | 1 | 7.22 | 2.1 | 0.00E+00 | 6.31E+06 | 0.00E+00 | 2103333.3 | 2.82E+06 | 2.64E+06 | 0.00E+00 | 1820000 | 0.87 | Q96J65.2 | RecName: Full=Multidrug resistance-associated protein 9: AltName: Full=ATP-binding cassette sub-family C member 12 |
| 186 | 1 | 1 | 7.19 | 3.7 | 0.00E+00 | 0.00E+00 | 0.00E+00 | 0 | 7.83E+05 | 0.00E+00 | 0.00E+00 | 261000 | #DIV/0! | Q7SHU8.1 | RecName: Full=Probable dipeptidyl-aminopeptidase B: Short=DPAP B |
| 187 | 1 | 1 | 7.18 | 26.8 | 1.45E+05 | 0.00E+00 | 0.00E+00 | 48333.333 | 0.00E+00 | 0.00E+00 | 0.00E+00 | 0 | 0.00 | A0KEK5.1 | RecName: Full=Sulfurtransferase TusA homolog |
| 188 | 1 | 1 | 7.15 | 14.6 | 0.00E+00 | 0.00E+00 | 0.00E+00 | 0 | 0.00E+00 | 9.46E+05 | 0.00E+00 | 315333.33 | #DIV/0! | O95816.1 | RecName: Full=BAG family molecular chaperone regulator 2: Short=BAG-2: AltName: Full=Bcl-2-associated athanogene 2 |
| 189 | 1 | 1 | 7.15 | 10.3 | 0.00E+00 | 0.00E+00 | 0.00E+00 | 0 | 0.00E+00 | 1.71E+06 | 0.00E+00 | 570000 | #DIV/0! | P52954.2 | RecName: Full=Transcription factor LBX1: AltName: Full=Ladybird homeobox protein homolog 1 |
| 190 | 1 | 1 | 7.15 | 2.7 | 0.00E+00 | 1.29E+06 | 0.00E+00 | 430000 | 0.00E+00 | 0.00E+00 | 0.00E+00 | 0 | 0.00 | Q0P4K8.1 | RecName: Full=NFATC2-interacting protein: AltName: Full=Nuclear factor of activated T-cells, cytoplasmic 2-interacting protein |
| 191 | 2 | 1 | 7.09 | 1 | 1.04E+06 | 0.00E+00 | 1.53E+06 | 856666.67 | 0.00E+00 | 0.00E+00 | 0.00E+00 | 0 | 0.00 | Q3YK19.1 | RecName: Full=Fanconi anemia group J protein homolog: Short=Protein FACJ: AltName: Full=ATP-dependent RNA helicase BRIP1 |
| 192 | 1 | 1 | 7.07 | 2.5 | 0.00E+00 | 0.00E+00 | 0.00E+00 | 0 | 0.00E+00 | 1.92E+04 | 0.00E+00 | 6400 | #DIV/0! | E1BP36.3 | RecName: Full=MMS19 nucleotide excision repair protein homolog: AltName: Full=MMS19-like protein |
| 193 | 1 | 1 | 7.04 | 6.2 | 0.00E+00 | 7.39E+05 | 0.00E+00 | 246333.33 | 0.00E+00 | 0.00E+00 | 0.00E+00 | 0 | 0.00 | P18738.1 | RecName: Full=Gastrula zinc finger protein XlCGF9.1 |
| 194 | 1 | 1 | 7.03 | 6 | 0.00E+00 | 0.00E+00 | 0.00E+00 | 0 | 0.00E+00 | 2.62E+06 | 0.00E+00 | 873333.33 | #DIV/0! | P0DD26.1 | RecName: Full=Probable dipeptidase Bgi\|342165246\|sp\|P0DD27.1\|PEPDB_STRPQ RecName: Full=Probable dipeptidase B |
| 195 | 1 | 1 | 7.01 | 3.5 | 0.00E+00 | 0.00E+00 | 0.00E+00 | 0 | 0.00E+00 | 3.16E+05 | 0.00E+00 | 105333.33 | #DIV/0! | Q12874.1 | RecName: Full=Splicing factor 3A subunit 3: AltName: Full=SF3a60: AltName: Full=Spliceosome-associated protein 61: Short=SAP 61 |
| 196 | 1 | 1 | 6.94 | 1.4 | 0.00E+00 | 0.00E+00 | 0.00E+00 | 0 | 0.00E+00 | 0.00E+00 | 1.03E+07 | 3433333.3 | #DIV/0! | B1LS97.1 | RecName: Full=Sulfite reductase [NADPH] hemoprotein beta-component: Short=SiR-HP: Short=SiRHP |
| 197 | 1 | 1 | 6.89 | 1.6 | 0.00E+00 | 0.00E+00 | 0.00E+00 | 0 | 0.00E+00 | 2.82E+05 | 0.00E+00 | 94000 | #DIV/0! | Q9SD67.1 | RecName: Full=ATP-dependent zinc metalloprotease FTSH 7, chloroplastic: Short=AtFTSH7: Flags: Precursor |
| 198 | 1 | 1 | 6.88 | 5.1 | 0.00E+00 | 0.00E+00 | 3.35E+05 | 111666.67 | 0.00E+00 | 0.00E+00 | 0.00E+00 | 0 | 0.00 | Q03LV6.1 | RecName: Full=Glycine--tRNA ligase beta subunit: AltName: Full=Glycyl-tRNA synthetase beta subunit: Short=GlyRS |
| 199 | 3 | 1 | 6.88 | 9.7 | 0.00E+00 | 0.00E+00 | 2.26E+06 | 753333.33 | 7.31E+04 | 8.86E+06 | 0.00E+00 | 2977700 | 3.95 | Q3ZWY8.1 | RecName: Full=30S ribosomal protein S16gi\|189044378\|sp\|A5FRP7.1\|RS16_DEHSB RecName: Full=30S ribosomal protein S16 |
| 200 | 1 | 1 | 6.86 | 3.1 | 0.00E+00 | 0.00E+00 | 8.98E+06 | 2993333.3 | 0.00E+00 | 0.00E+00 | 0.00E+00 | 0 | 0.00 | Q12851.2 | RecName: Full=Mitogen-activated protein kinase kinase kinase kinase 2: AltName: Full=B lymphocyte serine/threonine-protein kinase: AltName: Full=Germinal center kinase: Short=GC kinase: AltName: Full=MAPK/ERK kinase kinase kinase 2: Short=MEK kinase kinase 2: Short=MEKKK 2: AltName: Full=Rab8-interacting protein |
| 201 | 1 | 1 | 6.82 | 0.5 | 0.00E+00 | 0.00E+00 | 0.00E+00 | 0 | 0.00E+00 | 0.00E+00 | 4.62E+07 | 15400000 | #DIV/0! | Q96M86.2 | RecName: Full=Dynein heavy chain domain-containing protein 1: AltName: Full=Dynein heavy chain domain 1-like protein: AltName: Full=Protein CCDC35 |
| 202 | 1 | 1 | 6.82 | 17.1 | 0.00E+00 | 0.00E+00 | 0.00E+00 | 0 | 0.00E+00 | 0.00E+00 | 1.05E+04 | 3500 | #DIV/0! | Q9BTA0.2 | RecName: Full=Protein FAM167B |
| 203 | 1 | 1 | 6.78 | 2.8 | 0.00E+00 | 0.00E+00 | 0.00E+00 | 0 | 2.56E+06 | 0.00E+00 | 0.00E+00 | 853333.33 | #DIV/0! | Q12564.1 | RecName: Full=Chitin synthase A: AltName: Full=Chitin-UDP acetyl-glucosaminyl transferase A: AltName: Full=Class-I chitin synthase A |
| 204 | 3 | 1 | 6.72 | 17.6 | 0.00E+00 | 1.34E+05 | 0.00E+00 | 44666.667 | 0.00E+00 | 5.78E+05 | 0.00E+00 | 192666.67 | 4.31 | A1L1C2.1 | RecName: Full=Mitochondrial cardiolipin hydrolase: AltName: Full=Choline phosphatase 6: AltName: Full=Mitochondrial phospholipase: Short=MitoPLD: AltName: Full=Phosphatidylcholine-hydrolyzing phospholipase D6: AltName: Full=Phospholipase D6: Short=PLD 6 |
| 205 | 2 | 1 | 6.71 | 2.6 | 4.60E+05 | 0.00E+00 | 0.00E+00 | 153333.33 | 2.93E+06 | 0.00E+00 | 0.00E+00 | 976666.67 | 6.37 | Q31V62.1 | RecName: Full=Glycine--tRNA ligase beta subunit: AltName: Full=Glycyl-tRNA synthetase beta subunit: Short=GlyRS |
| 206 | 1 | 1 | 6.71 | 6 | 0.00E+00 | 0.00E+00 | 0.00E+00 | 0 | 1.05E+05 | 0.00E+00 | 0.00E+00 | 35000 | #DIV/0! | Q9VAS7.1 | RecName: Full=Innexin inx3: Short=Innexin-3 |
| 207 | 1 | 1 | 6.7 | 2.8 | 0.00E+00 | 0.00E+00 | 0.00E+00 | 0 | 6.26E+05 | 0.00E+00 | 0.00E+00 | 208666.67 | #DIV/0! | Q6TN15.1 | RecName: Full=Interferon-induced GTP-binding protein Mx: AltName: Full=Interferon-inducible Mx protein |
| 208 | 1 | 1 | 6.69 | 4.9 | 3.84E+05 | 0.00E+00 | 0.00E+00 | 128000 | 0.00E+00 | 0.00E+00 | 0.00E+00 | 0 | 0.00 | P59828.1 | RecName: Full=Poly(beta-D-mannuronate) C5 epimerase: Flags: Precursor |
| 209 | 1 | 1 | 6.67 | 0.6 | 0.00E+00 | 0.00E+00 | 0.00E+00 | 0 | 0.00E+00 | 1.84E+06 | 0.00E+00 | 613333.33 | #DIV/0! | Q8TDJ6.2 | RecName: Full=DmX-like protein 2: AltName: Full=Rabconnectin-3 |
| 210 | 1 | 1 | 6.61 | 1.1 | 1.07E+06 | 0.00E+00 | 0.00E+00 | 356666.67 | 0.00E+00 | 0.00E+00 | 0.00E+00 | 0 | 0.00 | Q562F6.2 | RecName: Full=Shugoshin-like 2: AltName: Full=Shugoshin-2: Short=Sgo2: AltName: Full=Tripin |
| 211 | 1 | 1 | 6.59 | 6.3 | 0.00E+00 | 0.00E+00 | 0.00E+00 | 0 | 0.00E+00 | 0.00E+00 | 3.81E+05 | 127000 | #DIV/0! | Q8EVD0.1 | RecName: Full=Glycerol kinase: AltName: Full=ATP:glycerol 3-phosphotransferase: AltName: Full=Glycerokinase: Short=GK |
| 212 | 3 | 1 | 6.56 | 6.7 | 0.00E+00 | 1.92E+06 | 1.95E+06 | 1290000 | 0.00E+00 | 0.00E+00 | 2.20E+06 | 733333.33 | 0.57 | A3CT71.1 | RecName: Full=Probable endonuclease 4: AltName: Full=Endodeoxyribonuclease IV: AltName: Full=Endonuclease IV |
| 213 | 1 | 1 | 6.56 | 1.6 | 0.00E+00 | 0.00E+00 | 0.00E+00 | 0 | 5.10E+06 | 0.00E+00 | 0.00E+00 | 1700000 | #DIV/0! | Q7Z392.2 | RecName: Full=Trafficking protein particle complex subunit 11 |
| 214 | 2 | 1 | 6.56 | 1.2 | 3.51E+05 | 0.00E+00 | 5.65E+05 | 305333.33 | 0.00E+00 | 0.00E+00 | 0.00E+00 | 0 | 0.00 | Q9C099.2 | RecName: Full=Leucine-rich repeat and coiled-coil domain-containing protein 1: AltName: Full=Centrosomal leucine-rich repeat and coiled-coil domain-containing protein |
| 215 | 1 | 1 | 6.55 | 7.5 | 0.00E+00 | 0.00E+00 | 0.00E+00 | 0 | 0.00E+00 | 0.00E+00 | 7.18E+05 | 239333.33 | #DIV/0! | C1AUB5.1 | RecName: Full=Probable cytosol aminopeptidase: AltName: Full=Leucine aminopeptidase: Short=LAP: AltName: Full=Leucyl aminopeptidase |
| 216 | 1 | 1 | 6.54 | 0.5 | 0.00E+00 | 0.00E+00 | 1.91E+06 | 636666.67 | 0.00E+00 | 0.00E+00 | 0.00E+00 | 0 | 0.00 | O75128.2 | RecName: Full=Protein cordon-bleu |
| 217 | 1 | 1 | 6.54 | 0.8 | 0.00E+00 | 0.00E+00 | 5.80E+05 | 193333.33 | 0.00E+00 | 0.00E+00 | 0.00E+00 | 0 | 0.00 | Q8NDV3.2 | RecName: Full=Structural maintenance of chromosomes protein 1B: Short=SMC protein 1B: Short=SMC-1-beta: Short=SMC-1B |
| 218 | 1 | 1 | 6.54 | 0.3 | 1.10E+06 | 0.00E+00 | 0.00E+00 | 366666.67 | 0.00E+00 | 0.00E+00 | 0.00E+00 | 0 | 0.00 | Q9SIV5.3 | RecName: Full=Zinc finger CCCH domain-containing protein 19: Short=AtC3H19: AltName: Full=Protein Needed for RDR2-independent DNA methylation |
| 219 | 1 | 1 | 6.53 | 4.5 | 0.00E+00 | 0.00E+00 | 6.04E+05 | 201333.33 | 0.00E+00 | 0.00E+00 | 0.00E+00 | 0 | 0.00 | P50768.1 | RecName: Full=Regulatory protein E2 |
| 220 | 1 | 1 | 6.5 | 1.2 | 0.00E+00 | 0.00E+00 | 0.00E+00 | 0 | 0.00E+00 | 1.79E+06 | 0.00E+00 | 596666.67 | #DIV/0! | Q5JSH3.1 | RecName: Full=WD repeat-containing protein 44: AltName: Full=Rabphilin-11 |
| 221 | 1 | 1 | 6.45 | 1.1 | 3.84E+05 | 0.00E+00 | 0.00E+00 | 128000 | 0.00E+00 | 0.00E+00 | 0.00E+00 | 0 | 0.00 | P89105.3 | RecName: Full=RNA polymerase-associated protein CTR9: AltName: Full=Centromere-binding factor 1-dependent protein 1: AltName: Full=Cln three-requiring protein 9 |
| 222 | 1 | 1 | 6.44 | 0.4 | 0.00E+00 | 0.00E+00 | 0.00E+00 | 0 | 2.12E+05 | 0.00E+00 | 0.00E+00 | 70666.667 | #DIV/0! | Q75JN1.1 | RecName: Full=Probable serine/threonine-protein kinase ifkC: AltName: Full=Initiation factor kinase C |
| 223 | 1 | 1 | 6.43 | 1.7 | 0.00E+00 | 4.17E+06 | 0.00E+00 | 1390000 | 0.00E+00 | 0.00E+00 | 0.00E+00 | 0 | 0.00 | Q14188.2 | RecName: Full=Transcription factor Dp-2: AltName: Full=E2F dimerization partner 2 |
| 224 | 2 | 1 | 6.42 | 5.4 | 4.66E+05 | 8.45E+05 | 0.00E+00 | 437000 | 0.00E+00 | 0.00E+00 | 0.00E+00 | 0 | 0.00 | A1RY72.1 | RecName: Full=DNA ligase 2: AltName: Full=Polydeoxyribonucleotide synthase [ATP] 2 |
| 225 | 1 | 1 | 6.42 | 1.4 | 4.23E+05 | 0.00E+00 | 0.00E+00 | 141000 | 0.00E+00 | 0.00E+00 | 0.00E+00 | 0 | 0.00 | Q8BTM8.5 | RecName: Full=Filamin-A: Short=FLN-A: AltName: Full=Actin-binding protein 280: Short=ABP-280: AltName: Full=Alpha-filamin: AltName: Full=Endothelial actin-binding protein: AltName: Full=Filamin-1: AltName: Full=Non-muscle filamin |
| 226 | 1 | 1 | 6.42 | 1.7 | 4.81E+05 | 0.00E+00 | 0.00E+00 | 160333.33 | 0.00E+00 | 0.00E+00 | 0.00E+00 | 0 | 0.00 | Q7UZL6.1 | RecName: Full=DNA mismatch repair protein MutS |
| 227 | 1 | 1 | 6.42 | 0.8 | 0.00E+00 | 0.00E+00 | 0.00E+00 | 0 | 0.00E+00 | 8.51E+06 | 0.00E+00 | 2836666.7 | #DIV/0! | Q9UPN4.3 | RecName: Full=5-azacytidine-induced protein 1: AltName: Full=Centrosomal protein of 131 kDa: Short=Cep131: AltName: Full=Pre-acrosome localization protein 1 |
| 228 | 1 | 1 | 6.41 | 3.9 | 0.00E+00 | 0.00E+00 | 0.00E+00 | 0 | 3.15E+06 | 0.00E+00 | 0.00E+00 | 1050000 | #DIV/0! | C4K4F9.1 | RecName: Full=Elongation factor G: Short=EF-G |
| 229 | 2 | 1 | 6.39 | 17.9 | 0.00E+00 | 0.00E+00 | 0.00E+00 | 0 | 0.00E+00 | 1.45E+06 | 1.37E+06 | 940000 | #DIV/0! | O06589.1 | RecName: Full=Imidazole glycerol phosphate synthase subunit HisH: AltName: Full=IGP synthase glutamine amidotransferase subunit: AltName: Full=IGP synthase subunit HisH: AltName: Full=ImGP synthase subunit HisH: Short=IGPS subunit HisH |
| 230 | 3 | 1 | 6.39 | 0.8 | 0.00E+00 | 0.00E+00 | 7.88E+05 | 262666.67 | 0.00E+00 | 0.00E+00 | 5.18E+05 | 172666.67 | 0.66 | Q7VNT4.1 | RecName: Full=Electron transport complex protein RnfC |
| 231 | 2 | 1 | 6.38 | 1.8 | 0.00E+00 | 1.12E+07 | 0.00E+00 | 3733333.3 | 0.00E+00 | 0.00E+00 | 0.00E+00 | 0 | 0.00 | O76083.1 | RecName: Full=High affinity cGMP-specific 3',5'-cyclic phosphodiesterase 9A |
| 232 | 1 | 1 | 6.38 | 5.2 | 0.00E+00 | 0.00E+00 | 0.00E+00 | 0 | 0.00E+00 | 0.00E+00 | 1.02E+05 | 34000 | #DIV/0! | Q54SA1.1 | RecName: Full=Phospholipase D Z: AltName: Full=Phosphatase D3: Short=PLD 3: Flags: Precursor |
| 233 | 1 | 1 | 6.37 | 1.1 | 0.00E+00 | 0.00E+00 | 0.00E+00 | 0 | 1.51E+06 | 0.00E+00 | 0.00E+00 | 503333.33 | #DIV/0! | P61134.1 | RecName: Full=Complement component C6: Flags: Precursor |
| 234 | 1 | 1 | 6.37 | 1.7 | 3.83E+05 | 0.00E+00 | 0.00E+00 | 127666.67 | 0.00E+00 | 0.00E+00 | 0.00E+00 | 0 | 0.00 | Q752Q3.2 | RecName: Full=Enhancer of polycomb-like protein 1 |
| 235 | 1 | 1 | 6.33 | 7.7 | 0.00E+00 | 0.00E+00 | 0.00E+00 | 0 | 0.00E+00 | 6.95E+05 | 0.00E+00 | 231666.67 | #DIV/0! | Q00325.2 | RecName: Full=Phosphate carrier protein, mitochondrial: AltName: Full=Phosphate transport protein: Short=PTP: AltName: Full=Solute carrier family 25 member 3: Flags: Precursor |
| 236 | 1 | 1 | 6.32 | 3.8 | 1.59E+05 | 0.00E+00 | 0.00E+00 | 53000 | 0.00E+00 | 0.00E+00 | 0.00E+00 | 0 | 0.00 | Q9KUR9.2 | RecName: Full=LPS-assembly protein LptD: AltName: Full=Organic solvent tolerance protein: Flags: Precursor |
| 237 | 1 | 1 | 6.31 | 4 | 0.00E+00 | 0.00E+00 | 0.00E+00 | 0 | 7.73E+05 | 0.00E+00 | 0.00E+00 | 257666.67 | #DIV/0! | B3L2G0.1 | RecName: Full=tRNA (guanine(37)-N1)-methyltransferase: AltName: Full=M1G-methyltransferase: AltName: Full=tRNA [GM37] methyltransferase: AltName: Full=tRNA methyltransferase 5 homolog |
| 238 | 1 | 1 | 6.31 | 1.8 | 6.79E+06 | 0.00E+00 | 0.00E+00 | 2263333.3 | 0.00E+00 | 0.00E+00 | 0.00E+00 | 0 | 0.00 | Q96Q42.2 | RecName: Full=Alsin: AltName: Full=Amyotrophic lateral sclerosis 2 chromosomal region candidate gene 6 protein: AltName: Full=Amyotrophic lateral sclerosis 2 protein |
| 239 | 2 | 1 | 6.29 | 4.2 | 0.00E+00 | 0.00E+00 | 0.00E+00 | 0 | 2.09E+06 | 0.00E+00 | 1.94E+06 | 1343333.3 | #DIV/0! | Q6XPS3.2 | RecName: Full=Phosphatidylinositol 3,4,5-trisphosphate 3-phosphatase TPTE2: AltName: Full=Lipid phosphatase TPIP: AltName: Full=TPTE and PTEN homologous inositol lipid phosphatase |
| 240 | 1 | 1 | 6.29 | 4.5 | 0.00E+00 | 0.00E+00 | 0.00E+00 | 0 | 0.00E+00 | 0.00E+00 | 2.08E+05 | 69333.333 | #DIV/0! | Q8NFI3.1 | RecName: Full=Cytosolic endo-beta-N-acetylglucosaminidase: Short=ENGase |
| 241 | 1 | 1 | 6.24 | 6.7 | 0.00E+00 | 0.00E+00 | 0.00E+00 | 0 | 4.48E+05 | 0.00E+00 | 0.00E+00 | 149333.33 | #DIV/0! | Q49842.2 | RecName: Full=(Dimethylallyl)adenosine tRNA methylthiotransferase MiaB: AltName: Full=tRNA-i(6)A37 methylthiotransferase |
| 242 | 1 | 1 | 6.24 | 2.3 | 0.00E+00 | 2.81E+04 | 0.00E+00 | 9366.6667 | 0.00E+00 | 0.00E+00 | 0.00E+00 | 0 | 0.00 | Q92995.2 | RecName: Full=Ubiquitin carboxyl-terminal hydrolase 13: AltName: Full=Deubiquitinating enzyme 13: AltName: Full=Isopeptidase T-3: Short=ISOT-3: AltName: Full=Ubiquitin thioesterase 13: AltName: Full=Ubiquitin-specific-processing protease 13 |
| 243 | 1 | 1 | 6.2 | 2.1 | 0.00E+00 | 0.00E+00 | 0.00E+00 | 0 | 8.38E+05 | 0.00E+00 | 0.00E+00 | 279333.33 | #DIV/0! | Q68DV7.1 | RecName: Full=E3 ubiquitin-protein ligase RNF43: AltName: Full=RING finger protein 43: Flags: Precursor |
| 244 | 1 | 1 | 6.2 | 3.7 | 0.00E+00 | 0.00E+00 | 0.00E+00 | 0 | 0.00E+00 | 7.34E+05 | 0.00E+00 | 244666.67 | #DIV/0! | Q9ZDC9.1 | RecName: Full=Putative carboxypeptidase RP402 |
| 245 | 1 | 1 | 6.19 | 2 | 0.00E+00 | 0.00E+00 | 0.00E+00 | 0 | 5.83E+05 | 0.00E+00 | 0.00E+00 | 194333.33 | #DIV/0! | Q6ZV73.2 | RecName: Full=FYVE, RhoGEF and PH domain-containing protein 6: AltName: Full=Zinc finger FYVE domain-containing protein 24 |
| 246 | 1 | 1 | 6.16 | 1.5 | 0.00E+00 | 3.72E+05 | 0.00E+00 | 124000 | 0.00E+00 | 0.00E+00 | 0.00E+00 | 0 | 0.00 | O82139.1 | RecName: Full=Cycloartenol Synthase |
| 247 | 1 | 1 | 6.15 | 2.2 | 0.00E+00 | 0.00E+00 | 0.00E+00 | 0 | 0.00E+00 | 3.83E+05 | 0.00E+00 | 127666.67 | #DIV/0! | Q02512.2 | RecName: Full=Replication protein E1: AltName: Full=ATP-dependent helicase E1 |
| 248 | 1 | 1 | 6.13 | 3.8 | 0.00E+00 | 4.49E+05 | 0.00E+00 | 149666.67 | 0.00E+00 | 0.00E+00 | 0.00E+00 | 0 | 0.00 | B4U9K9.1 | RecName: Full=Adenylosuccinate synthetase: Short=AMPSase: Short=AdSS: AltName: Full=IMP--aspartate ligase |
| 249 | 1 | 1 | 6.1 | 13.6 | 0.00E+00 | 2.17E+05 | 0.00E+00 | 72333.333 | 0.00E+00 | 0.00E+00 | 0.00E+00 | 0 | 0.00 | A4XTF3.1 | RecName: Full=Integration host factor subunit beta: Short=IHF-beta |
| 250 | 1 | 1 | 6.1 | 0.3 | 0.00E+00 | 0.00E+00 | 0.00E+00 | 0 | 4.73E+05 | 0.00E+00 | 0.00E+00 | 157666.67 | #DIV/0! | O75592.3 | RecName: Full=Probable E3 ubiquitin-protein ligase MYCBP2: AltName: Full=Myc-binding protein 2: AltName: Full=Pam/highwire/rpm-1 protein: AltName: Full=Protein associated with Myc |
| 251 | 1 | 1 | 6.07 | 1.6 | 0.00E+00 | 0.00E+00 | 0.00E+00 | 0 | 6.62E+05 | 0.00E+00 | 0.00E+00 | 220666.67 | #DIV/0! | Q9Y2H2.3 | RecName: Full=Phosphatidylinositide phosphatase SAC2: AltName: Full=Inositol polyphosphate 5-phosphatase F: AltName: Full=Sac domain-containing inositol phosphatase 2: AltName: Full=Sac domain-containing phosphoinositide 5-phosphatase 2: Short=hSAC2 |
| 252 | 1 | 1 | 6.06 | 1.4 | 7.07E+05 | 0.00E+00 | 0.00E+00 | 235666.67 | 0.00E+00 | 0.00E+00 | 0.00E+00 | 0 | 0.00 | Q9LVR3.1 | RecName: Full=UPF0496 protein At5g66670 |
| 253 | 1 | 1 | 6.05 | 4.6 | 0.00E+00 | 0.00E+00 | 0.00E+00 | 0 | 4.47E+05 | 0.00E+00 | 0.00E+00 | 149000 | #DIV/0! | P35249.2 | RecName: Full=Replication factor C subunit 4: AltName: Full=Activator 1 37 kDa subunit: Short=A1 37 kDa subunit: AltName: Full=Activator 1 subunit 4: AltName: Full=Replication factor C 37 kDa subunit: Short=RF-C 37 kDa subunit: Short=RFC37 |
| 254 | 1 | 1 | 6 | 2.5 | 0.00E+00 | 0.00E+00 | 6.11E+05 | 203666.67 | 0.00E+00 | 0.00E+00 | 0.00E+00 | 0 | 0.00 | Q7UA23.1 | RecName: Full=DNA mismatch repair protein MutS |
| 255 | 1 | 1 | 5.99 | 2.1 | 0.00E+00 | 0.00E+00 | 0.00E+00 | 0 | 0.00E+00 | 0.00E+00 | 8.30E+05 | 276666.67 | #DIV/0! | O15067.4 | RecName: Full=Phosphoribosylformylglycinamidine synthase: Short=FGAM synthase: Short=FGAMS: AltName: Full=Formylglycinamide ribotide amidotransferase: Short=FGARAT: AltName: Full=Formylglycinamide ribotide synthetase |
| 256 | 1 | 1 | 5.98 | 1.7 | 0.00E+00 | 0.00E+00 | 0.00E+00 | 0 | 1.13E+06 | 0.00E+00 | 0.00E+00 | 376666.67 | #DIV/0! | Q8WTR7.1 | RecName: Full=Zinc finger protein 473: AltName: Full=Zinc finger protein 100 homolog: Short=Zfp-100 |
| 257 | 1 | 1 | 5.97 | 5.3 | 0.00E+00 | 0.00E+00 | 0.00E+00 | 0 | 0.00E+00 | 0.00E+00 | 3.81E+05 | 127000 | #DIV/0! | Q4G176.3 | RecName: Full=Acyl-CoA synthetase family member 3, mitochondrial: Flags: Precursor |
| 258 | 1 | 1 | 5.97 | 1.3 | 0.00E+00 | 0.00E+00 | 0.00E+00 | 0 | 3.62E+05 | 0.00E+00 | 0.00E+00 | 120666.67 | #DIV/0! | Q5VXU9.1 | RecName: Full=Uncharacterized protein C9orf84 |
| 259 | 1 | 1 | 5.97 | 2.4 | 0.00E+00 | 0.00E+00 | 0.00E+00 | 0 | 4.27E+06 | 0.00E+00 | 0.00E+00 | 1423333.3 | #DIV/0! | Q6UXZ4.1 | RecName: Full=Netrin receptor UNC5D: AltName: Full=Protein unc-5 homolog 4: AltName: Full=Protein unc-5 homolog D: Flags: Precursor |
| 260 | 1 | 1 | 5.96 | 1.8 | 8.50E+05 | 0.00E+00 | 0.00E+00 | 283333.33 | 0.00E+00 | 0.00E+00 | 0.00E+00 | 0 | 0.00 | P26358.2 | RecName: Full=DNA (cytosine-5)-methyltransferase 1: Short=Dnmt1: AltName: Full=CXXC-type zinc finger protein 9: AltName: Full=DNA methyltransferase HsaI: Short=DNA MTase HsaI: Short=M.HsaI: AltName: Full=MCMT |
| 261 | 1 | 1 | 5.95 | 9.9 | 0.00E+00 | 0.00E+00 | 0.00E+00 | 0 | 0.00E+00 | 0.00E+00 | 2.77E+06 | 923333.33 | #DIV/0! | O43709.2 | RecName: Full=Uncharacterized methyltransferase WBSCR22: AltName: Full=Williams-Beuren syndrome chromosomal region 22 protein |
| 262 | 1 | 1 | 5.95 | 1.5 | 3.23E+06 | 0.00E+00 | 0.00E+00 | 1076666.7 | 0.00E+00 | 0.00E+00 | 0.00E+00 | 0 | 0.00 | P32386.2 | RecName: Full=ATP-dependent bile acid permease |
| 263 | 1 | 1 | 5.95 | 8.5 | 1.59E+05 | 0.00E+00 | 0.00E+00 | 53000 | 0.00E+00 | 0.00E+00 | 0.00E+00 | 0 | 0.00 | P36781.1 | RecName: Full=Regulatory protein E2 |
| 264 | 1 | 1 | 5.94 | 10.7 | 0.00E+00 | 0.00E+00 | 0.00E+00 | 0 | 0.00E+00 | 0.00E+00 | 3.55E+05 | 118333.33 | #DIV/0! | P09124.2 | RecName: Full=Glyceraldehyde-3-phosphate dehydrogenase 1: AltName: Full=NAD-dependent glyceraldehyde-3-phosphate dehydrogenase: Short=GAPDH |
| 265 | 1 | 1 | 5.92 | 3.8 | 0.00E+00 | 0.00E+00 | 0.00E+00 | 0 | 0.00E+00 | 3.54E+05 | 0.00E+00 | 118000 | #DIV/0! | Q0WVN5.2 | RecName: Full=Cellulose synthase-like protein G3: Short=AtCslG3 |
| 266 | 1 | 1 | 5.92 | 12.6 | 0.00E+00 | 0.00E+00 | 0.00E+00 | 0 | 0.00E+00 | 3.83E+05 | 0.00E+00 | 127666.67 | #DIV/0! | Q1MPQ7.1 | RecName: Full=30S ribosomal protein S3 |
| 267 | 1 | 1 | 5.9 | 2.6 | 0.00E+00 | 0.00E+00 | 0.00E+00 | 0 | 0.00E+00 | 1.70E+06 | 0.00E+00 | 566666.67 | #DIV/0! | Q5DRC2.1 | RecName: Full=Protocadherin gamma-A1: Short=PCDH-gamma-A1: Flags: Precursor |
| 268 | 1 | 1 | 5.88 | 20.8 | 0.00E+00 | 0.00E+00 | 0.00E+00 | 0 | 4.35E+04 | 0.00E+00 | 0.00E+00 | 14500 | #DIV/0! | Q9BPH3.1 | RecName: Full=Conotoxin ArMLKM-01: Flags: Precursor |
| 269 | 1 | 1 | 5.87 | 3.2 | 1.52E+07 | 0.00E+00 | 0.00E+00 | 5066666.7 | 0.00E+00 | 0.00E+00 | 0.00E+00 | 0 | 0.00 | Q6ZW76.1 | RecName: Full=Ankyrin repeat and SAM domain-containing protein 3 |
| 270 | 1 | 1 | 5.86 | 4.1 | 0.00E+00 | 0.00E+00 | 6.30E+05 | 210000 | 0.00E+00 | 0.00E+00 | 0.00E+00 | 0 | 0.00 | Q8K9B2.1 | RecName: Full=Fructose-bisphosphate aldolase class 2: Short=FBP aldolase: Short=FBPA: AltName: Full=Fructose-1,6-bisphosphate aldolase: AltName: Full=Fructose-bisphosphate aldolase class II |
| 271 | 1 | 1 | 5.85 | 4.9 | 0.00E+00 | 0.00E+00 | 0.00E+00 | 0 | 0.00E+00 | 0.00E+00 | 1.79E+05 | 59666.667 | #DIV/0! | O94776.1 | RecName: Full=Metastasis-associated protein MTA2: AltName: Full=Metastasis-associated 1-like 1: Short=MTA1-L1 protein: AltName: Full=p53 target protein in deacetylase complex |
| 272 | 1 | 1 | 5.85 | 3.3 | 0.00E+00 | 0.00E+00 | 0.00E+00 | 0 | 4.83E+05 | 0.00E+00 | 0.00E+00 | 161000 | #DIV/0! | Q09501.1 | RecName: Full=Intermediate filament protein ifp-1: AltName: Full=Cel IF E1: AltName: Full=Intermediate filament protein E1: Short=IF-E1 |
| 273 | 1 | 1 | 5.85 | 1.1 | 0.00E+00 | 4.72E+05 | 0.00E+00 | 157333.33 | 0.00E+00 | 0.00E+00 | 0.00E+00 | 0 | 0.00 | Q14678.3 | RecName: Full=KN motif and ankyrin repeat domain-containing protein 1: AltName: Full=Ankyrin repeat domain-containing protein 15: AltName: Full=Kidney ankyrin repeat-containing protein |
| 274 | 1 | 1 | 5.84 | 3 | 0.00E+00 | 0.00E+00 | 0.00E+00 | 0 | 0.00E+00 | 0.00E+00 | 5.91E+05 | 197000 | #DIV/0! | P30760.2 | RecName: Full=DNA-directed RNA polymerase subunit beta: Short=RNAP subunit beta: AltName: Full=RNA polymerase subunit beta: AltName: Full=Transcriptase subunit betagi\|254765297\|sp\|B8ZSC7.1\|RPOB_MYCLB RecName: Full=DNA-directed RNA polymerase subunit beta: Short=RNAP subunit beta: AltName: Full=RNA polymerase subunit beta: AltName: Full=Transcriptase subunit beta |
| 275 | 1 | 1 | 5.82 | 8.5 | 0.00E+00 | 0.00E+00 | 0.00E+00 | 0 | 0.00E+00 | 0.00E+00 | 3.55E+05 | 118333.33 | #DIV/0! | C3K334.1 | RecName: Full=Multifunctional CCA protein: Includes: RecName: Full=CCA-adding enzyme: AltName: Full=CCA tRNA nucleotidyltransferase: AltName: Full=tRNA CCA-pyrophosphorylase: AltName: Full=tRNA adenylyl-/cytidylyl-transferase: AltName: Full=tRNA nucleotidyltransferase: AltName: Full=tRNA-NT: Includes: RecName: Full=2'-nucleotidase: Includes: RecName: Full=2',3'-cyclic phosphodiesterase: Includes: RecName: Full=Phosphatase |
| 276 | 1 | 1 | 5.82 | 6.6 | 2.51E+06 | 0.00E+00 | 0.00E+00 | 836666.67 | 0.00E+00 | 0.00E+00 | 0.00E+00 | 0 | 0.00 | Q9LV41.1 | RecName: Full=Putative F-box/kelch-repeat protein At3g24610 |
| 277 | 1 | 1 | 5.76 | 0.7 | 1.85E+05 | 0.00E+00 | 0.00E+00 | 61666.667 | 0.00E+00 | 0.00E+00 | 0.00E+00 | 0 | 0.00 | Q1J9C1.1 | RecName: Full=DNA mismatch repair protein MutSgi\|166232143\|sp\|Q1JJH0.1\|MUTS_STRPC RecName: Full=DNA mismatch repair protein MutS |
| 278 | 1 | 1 | 5.76 | 0.8 | 0.00E+00 | 0.00E+00 | 0.00E+00 | 0 | 5.87E+05 | 0.00E+00 | 0.00E+00 | 195666.67 | #DIV/0! | Q8IYA2.3 | RecName: Full=Coiled-coil domain-containing protein 144C |
| 279 | 1 | 1 | 5.74 | 0.5 | 0.00E+00 | 0.00E+00 | 0.00E+00 | 0 | 0.00E+00 | 0.00E+00 | 1.26E+06 | 420000 | #DIV/0! | P35724.1 | RecName: Full=Manganese resistance protein MNR2 |
| 280 | 1 | 1 | 5.74 | 5.2 | 0.00E+00 | 0.00E+00 | 0.00E+00 | 0 | 0.00E+00 | 1.71E+06 | 0.00E+00 | 570000 | #DIV/0! | Q5HZH2.1 | RecName: Full=Ribosome biogenesis protein TSR3 homolog |
| 281 | 2 | 1 | 5.73 | 6.4 | 0.00E+00 | 0.00E+00 | 0.00E+00 | 0 | 0.00E+00 | 7.73E+05 | 6.88E+05 | 487000 | #DIV/0! | Q9NS85.1 | RecName: Full=Carbonic anhydrase-related protein 10: AltName: Full=Carbonic anhydrase-related protein X: Short=CA-RP X: Short=CARP X: AltName: Full=Cerebral protein 15gi\|47115606\|sp\|P61215.1\|CAH10_MOUSE RecName: Full=Carbonic anhydrase-related protein 10: AltName: Full=Carbonic anhydrase-related protein X: Short=CA-RP X: Short=CARP Xgi\|52000736\|sp\|Q9N085.1\|CAH10_MACFA RecName: Full=Carbonic anhydrase-related protein 10gi\|75041009\|sp\|Q5R4U0.1\|CAH10_PONAB RecName: Full=Carbonic anhydrase-related protein 10 |
| 282 | 1 | 1 | 5.34 | 6.4 | 0.00E+00 | 0.00E+00 | 0.00E+00 | 0 | 0.00E+00 | 7.73E+05 | 0.00E+00 | 257666.67 | #DIV/0! | A0JN41.1 | RecName: Full=Carbonic anhydrase-related protein 10 |
| 283 | 1 | 1 | 5.71 | 3 | 1.20E+06 | 0.00E+00 | 0.00E+00 | 400000 | 0.00E+00 | 0.00E+00 | 0.00E+00 | 0 | 0.00 | O43159.2 | RecName: Full=Ribosomal RNA-processing protein 8: AltName: Full=Cerebral protein 1: AltName: Full=Nucleomethylin |
| 284 | 1 | 1 | 5.71 | 2.4 | 0.00E+00 | 0.00E+00 | 0.00E+00 | 0 | 0.00E+00 | 0.00E+00 | 2.62E+05 | 87333.333 | #DIV/0! | P30501.1 | RecName: Full=HLA class I histocompatibility antigen, Cw-2 alpha chain: AltName: Full=MHC class I antigen Cw*2: Flags: Precursor |
| 285 | 1 | 1 | 5.7 | 8.2 | 0.00E+00 | 0.00E+00 | 0.00E+00 | 0 | 0.00E+00 | 0.00E+00 | 3.16E+05 | 105333.33 | #DIV/0! | O48915.1 | RecName: Full=Protein NDR1: AltName: Full=Non-race specific disease resistance protein 1: Short=AtNDR1: Flags: Precursor |
| 286 | 2 | 1 | 5.69 | 1.6 | 0.00E+00 | 0.00E+00 | 0.00E+00 | 0 | 4.66E+06 | 3.63E+06 | 0.00E+00 | 2763333.3 | #DIV/0! | Q5IF00.1 | RecName: Full=Autophagy-related protein 28 |
| 287 | 1 | 1 | 5.68 | 0.7 | 0.00E+00 | 0.00E+00 | 0.00E+00 | 0 | 0.00E+00 | 0.00E+00 | 5.63E+06 | 1876666.7 | #DIV/0! | Q8R508.1 | RecName: Full=Protocadherin Fat 3: AltName: Full=FAT tumor suppressor homolog 3: Flags: Precursor |
| 288 | 1 | 1 | 5.67 | 1.2 | 0.00E+00 | 0.00E+00 | 0.00E+00 | 0 | 0.00E+00 | 6.27E+06 | 0.00E+00 | 2090000 | #DIV/0! | P35556.3 | RecName: Full=Fibrillin-2: Flags: Precursor |
| 289 | 1 | 1 | 5.65 | 2.4 | 0.00E+00 | 6.87E+05 | 0.00E+00 | 229000 | 0.00E+00 | 0.00E+00 | 0.00E+00 | 0 | 0.00 | B3W6N4.1 | RecName: Full=Chromosomal replication initiator protein DnaA |
| 290 | 1 | 1 | 5.64 | 6.8 | 0.00E+00 | 0.00E+00 | 4.83E+05 | 161000 | 0.00E+00 | 0.00E+00 | 0.00E+00 | 0 | 0.00 | Q49420.1 | RecName: Full=Probable cysteine desulfurase |
| 291 | 1 | 1 | 5.64 | 2.4 | 0.00E+00 | 0.00E+00 | 0.00E+00 | 0 | 0.00E+00 | 3.12E+06 | 0.00E+00 | 1040000 | #DIV/0! | Q5R4P8.1 | RecName: Full=Zinc finger protein 574 |
| 292 | 1 | 1 | 5.63 | 4.4 | 0.00E+00 | 1.15E+06 | 0.00E+00 | 383333.33 | 0.00E+00 | 0.00E+00 | 0.00E+00 | 0 | 0.00 | Q09179.2 | RecName: Full=Glutamine synthetase: Short=GS: AltName: Full=Glutamate--ammonia ligase |
| 293 | 1 | 1 | 5.63 | 3.9 | 0.00E+00 | 0.00E+00 | 0.00E+00 | 0 | 3.56E+05 | 0.00E+00 | 0.00E+00 | 118666.67 | #DIV/0! | Q8CXQ6.1 | RecName: Full=tRNA (guanine-N(1)-)-methyltransferase: AltName: Full=M1G-methyltransferase: AltName: Full=tRNA [GM37] methyltransferase |
| 294 | 1 | 1 | 5.62 | 1.3 | 0.00E+00 | 0.00E+00 | 0.00E+00 | 0 | 7.53E+06 | 0.00E+00 | 0.00E+00 | 2510000 | #DIV/0! | A1L020.1 | RecName: Full=RNA-binding protein MEX3A: AltName: Full=RING finger and KH domain-containing protein 4 |
| 295 | 1 | 1 | 5.61 | 3 | 0.00E+00 | 0.00E+00 | 0.00E+00 | 0 | 5.08E+05 | 0.00E+00 | 0.00E+00 | 169333.33 | #DIV/0! | Q15NV5.1 | RecName: Full=3-methyl-2-oxobutanoate hydroxymethyltransferase: AltName: Full=Ketopantoate hydroxymethyltransferase: Short=KPHMT |
| 296 | 1 | 1 | 5.6 | 2 | 0.00E+00 | 0.00E+00 | 0.00E+00 | 0 | 4.68E+05 | 0.00E+00 | 0.00E+00 | 156000 | #DIV/0! | A4W5D0.1 | RecName: Full=Isocitrate dehydrogenase kinase/phosphatase: Short=IDH kinase/phosphatase: Short=IDHK/P |
| 297 | 1 | 1 | 5.59 | 5.1 | 0.00E+00 | 2.74E+05 | 0.00E+00 | 91333.333 | 0.00E+00 | 0.00E+00 | 0.00E+00 | 0 | 0.00 | O27698.1 | RecName: Full=Anthranilate phosphoribosyltransferase |
| 298 | 1 | 1 | 5.59 | 2.8 | 0.00E+00 | 0.00E+00 | 5.82E+05 | 194000 | 0.00E+00 | 0.00E+00 | 0.00E+00 | 0 | 0.00 | Q68DI1.2 | RecName: Full=Zinc finger protein 776 |
| 299 | 1 | 1 | 5.57 | 2.3 | 2.39E+06 | 0.00E+00 | 0.00E+00 | 796666.67 | 0.00E+00 | 0.00E+00 | 0.00E+00 | 0 | 0.00 | Q5XG99.2 | RecName: Full=LysM and putative peptidoglycan-binding domain-containing protein 4 |
| 300 | 1 | 1 | 5.57 | 6.5 | 0.00E+00 | 0.00E+00 | 0.00E+00 | 0 | 9.69E+05 | 0.00E+00 | 0.00E+00 | 323000 | #DIV/0! | Q9BQY9.3 | RecName: Full=Dysbindin domain-containing protein 2: Short=Casein kinase-1 binding protein: AltName: Full=CK1BP: AltName: Full=HSMNP1 |
| 301 | 1 | 1 | 5.56 | 1.7 | 0.00E+00 | 1.15E+06 | 0.00E+00 | 383333.33 | 0.00E+00 | 0.00E+00 | 0.00E+00 | 0 | 0.00 | A4S6Y4.1 | RecName: Full=Lon protease homolog, mitochondrial: Flags: Precursor |
| 302 | 1 | 1 | 5.56 | 0.4 | 0.00E+00 | 0.00E+00 | 0.00E+00 | 0 | 3.22E+06 | 0.00E+00 | 0.00E+00 | 1073333.3 | #DIV/0! | P98158.1 | RecName: Full=Low-density lipoprotein receptor-related protein 2: Short=LRP-2: AltName: Full=Glycoprotein 330: Short=gp330: AltName: Full=Megalin: Flags: Precursor |
| 303 | 1 | 1 | 5.53 | 0.8 | 0.00E+00 | 0.00E+00 | 0.00E+00 | 0 | 2.23E+06 | 0.00E+00 | 0.00E+00 | 743333.33 | #DIV/0! | Q8IYB8.1 | RecName: Full=ATP-dependent RNA helicase SUPV3L1, mitochondrial: AltName: Full=Suppressor of var1 3-like protein 1: Short=SUV3-like protein 1: Flags: Precursor |
| 304 | 1 | 1 | 5.52 | 0.7 | 0.00E+00 | 0.00E+00 | 0.00E+00 | 0 | 0.00E+00 | 1.10E+06 | 0.00E+00 | 366666.67 | #DIV/0! | O14924.1 | RecName: Full=Regulator of G-protein signaling 12: Short=RGS12 |
| 305 | 1 | 1 | 5.51 | 2.7 | 0.00E+00 | 0.00E+00 | 0.00E+00 | 0 | 2.61E+05 | 0.00E+00 | 0.00E+00 | 87000 | #DIV/0! | B2IC30.1 | RecName: Full=Formate--tetrahydrofolate ligase: AltName: Full=Formyltetrahydrofolate synthetase: Short=FHS: Short=FTHFS |
| 306 | 1 | 1 | 5.51 | 31.1 | 0.00E+00 | 4.70E+06 | 0.00E+00 | 1566666.7 | 0.00E+00 | 0.00E+00 | 0.00E+00 | 0 | 0.00 | P05109.1 | RecName: Full=Protein S100-A8: AltName: Full=Calgranulin-A: AltName: Full=Calprotectin L1L subunit: AltName: Full=Cystic fibrosis antigen: Short=CFAG: AltName: Full=Leukocyte L1 complex light chain: AltName: Full=Migration inhibitory factor-related protein 8: Short=MRP-8: Short=p8: AltName: Full=S100 calcium-binding protein A8: AltName: Full=Urinary stone protein band A |
| 307 | 1 | 1 | 5.48 | 1.6 | 0.00E+00 | 0.00E+00 | 0.00E+00 | 0 | 0.00E+00 | 7.22E+05 | 0.00E+00 | 240666.67 | #DIV/0! | P48736.3 | RecName: Full=Phosphatidylinositol 4,5-bisphosphate 3-kinase catalytic subunit gamma isoform: Short=PI3-kinase subunit gamma: Short=PI3K-gamma: Short=PI3Kgamma: Short=PtdIns-3-kinase subunit gamma: AltName: Full=Phosphatidylinositol 4,5-bisphosphate 3-kinase 110 kDa catalytic subunit gamma: Short=PtdIns-3-kinase subunit p110-gamma: Short=p110gamma: AltName: Full=Phosphoinositide-3-kinase catalytic gamma polypeptide: AltName: Full=Serine/threonine protein kinase PIK3CG: AltName: Full=p120-PI3K |
| 308 | 1 | 1 | 5.48 | 5.4 | 0.00E+00 | 0.00E+00 | 0.00E+00 | 0 | 5.10E+06 | 0.00E+00 | 0.00E+00 | 1700000 | #DIV/0! | P52424.1 | RecName: Full=Phosphoribosylformylglycinamidine cyclo-ligase, chloroplastic/mitochondrial: AltName: Full=AIR synthase: Short=AIRS: AltName: Full=Phosphoribosyl-aminoimidazole synthetase: AltName: Full=VUpur5: Flags: Precursor |
| 309 | 1 | 1 | 5.48 | 4.4 | 0.00E+00 | 0.00E+00 | 0.00E+00 | 0 | 1.58E+05 | 0.00E+00 | 0.00E+00 | 52666.667 | #DIV/0! | Q58175.2 | RecName: Full=Hydroxylamine reductase: AltName: Full=Hybrid-cluster protein: Short=HCP |
| 310 | 1 | 1 | 5.48 | 0.7 | 4.73E+06 | 0.00E+00 | 0.00E+00 | 1576666.7 | 0.00E+00 | 0.00E+00 | 0.00E+00 | 0 | 0.00 | Q5THR3.1 | RecName: Full=EF-hand calcium-binding domain-containing protein 6: AltName: Full=CAP-binding protein complex-interacting protein 1: AltName: Full=DJ-1-binding protein: Short=DJBP |
| 311 | 1 | 1 | 5.48 | 6.9 | 0.00E+00 | 0.00E+00 | 0.00E+00 | 0 | 0.00E+00 | 0.00E+00 | 3.10E+05 | 103333.33 | #DIV/0! | Q969X2.1 | RecName: Full=Alpha-N-acetylgalactosaminide alpha-2,6-sialyltransferase 6: AltName: Full=GalNAc alpha-2,6-sialyltransferase VI: AltName: Full=ST6GalNAc VI: Short=ST6GalNAcVI: Short=hST6GalNAc VI: AltName: Full=Sialyltransferase 7F: Short=SIAT7-F |
| 312 | 1 | 1 | 5.47 | 5.2 | 2.59E+05 | 0.00E+00 | 0.00E+00 | 86333.333 | 0.00E+00 | 0.00E+00 | 0.00E+00 | 0 | 0.00 | Q9NQ79.2 | RecName: Full=Cartilage acidic protein 1: AltName: Full=68 kDa chondrocyte-expressed protein: Short=CEP-68: AltName: Full=ASPIC: Flags: Precursor |
| 313 | 1 | 1 | 5.46 | 2.8 | 0.00E+00 | 0.00E+00 | 2.59E+05 | 86333.333 | 0.00E+00 | 0.00E+00 | 0.00E+00 | 0 | 0.00 | A4WTP4.1 | RecName: Full=GMP synthase [glutamine-hydrolyzing]: AltName: Full=GMP synthetase: AltName: Full=Glutamine amidotransferase |
| 314 | 1 | 1 | 5.46 | 4.9 | 0.00E+00 | 0.00E+00 | 2.94E+05 | 98000 | 0.00E+00 | 0.00E+00 | 0.00E+00 | 0 | 0.00 | Q38WL5.1 | RecName: Full=Methionine import ATP-binding protein MetN |
| 315 | 1 | 1 | 5.46 | 4.2 | 0.00E+00 | 0.00E+00 | 0.00E+00 | 0 | 1.80E+06 | 0.00E+00 | 0.00E+00 | 600000 | #DIV/0! | Q6MFS1.1 | RecName: Full=Exocyst complex protein EXO70 |
| 316 | 2 | 1 | 5.45 | 6.1 | 2.35E+05 | 2.30E+05 | 0.00E+00 | 155000 | 0.00E+00 | 0.00E+00 | 0.00E+00 | 0 | 0.00 | D2Y283.1 | RecName: Full=Hainantoxin-XVI-13: Short=HNTX-XVI-13: Flags: Precursor |
| 317 | 1 | 1 | 5.45 | 12.4 | 0.00E+00 | 0.00E+00 | 0.00E+00 | 0 | 0.00E+00 | 6.22E+05 | 0.00E+00 | 207333.33 | #DIV/0! | Q57133.1 | RecName: Full=Uncharacterized protein HI_0973 |
| 318 | 1 | 1 | 5.43 | 1.8 | 0.00E+00 | 1.19E+06 | 0.00E+00 | 396666.67 | 0.00E+00 | 0.00E+00 | 0.00E+00 | 0 | 0.00 | B7IFR8.1 | RecName: Full=tRNA(Ile)-lysidine synthase: AltName: Full=tRNA(Ile)-2-lysyl-cytidine synthase: AltName: Full=tRNA(Ile)-lysidine synthetase |
| 319 | 1 | 1 | 5.43 | 7.1 | 0.00E+00 | 0.00E+00 | 0.00E+00 | 0 | 1.51E+06 | 0.00E+00 | 0.00E+00 | 503333.33 | #DIV/0! | O19074.1 | RecName: Full=Cytidine monophosphate-N-acetylneuraminic acid hydroxylase: Short=CMP-N-acetylneuraminic acid hydroxylase: AltName: Full=CMP-N-acetylneuraminate monooxygenase: AltName: Full=CMP-Neu5Ac hydroxylase: AltName: Full=CMP-NeuAc hydroxylase |
| 320 | 1 | 1 | 5.42 | 3.6 | 0.00E+00 | 0.00E+00 | 0.00E+00 | 0 | 0.00E+00 | 0.00E+00 | 2.68E+05 | 89333.333 | #DIV/0! | P28028.3 | RecName: Full=Serine/threonine-protein kinase B-raf: AltName: Full=Proto-oncogene B-Raf |
| 321 | 1 | 1 | 5.42 | 0.1 | 0.00E+00 | 0.00E+00 | 2.67E+06 | 890000 | 0.00E+00 | 0.00E+00 | 0.00E+00 | 0 | 0.00 | P78527.3 | RecName: Full=DNA-dependent protein kinase catalytic subunit: Short=DNA-PK catalytic subunit: Short=DNA-PKcs: AltName: Full=DNPK1: AltName: Full=p460 |
| 322 | 1 | 1 | 5.39 | 5.3 | 0.00E+00 | 0.00E+00 | 0.00E+00 | 0 | 2.28E+06 | 0.00E+00 | 0.00E+00 | 760000 | #DIV/0! | Q8NGM1.1 | RecName: Full=Olfactory receptor 4C15: AltName: Full=Olfactory receptor OR11-127: AltName: Full=Olfactory receptor OR11-134 |
| 323 | 1 | 1 | 5.38 | 2.8 | 0.00E+00 | 0.00E+00 | 0.00E+00 | 0 | 0.00E+00 | 0.00E+00 | 2.87E+06 | 956666.67 | #DIV/0! | Q8YH20.1 | RecName: Full=Beta-(1-->2)glucan export ATP-binding/permease protein NdvA |
| 324 | 1 | 1 | 5.37 | 0.8 | 0.00E+00 | 0.00E+00 | 0.00E+00 | 0 | 5.22E+05 | 0.00E+00 | 0.00E+00 | 174000 | #DIV/0! | B4GBA9.2 | RecName: Full=Protein teflon |
| 325 | 1 | 1 | 5.37 | 1.3 | 0.00E+00 | 0.00E+00 | 0.00E+00 | 0 | 6.64E+06 | 0.00E+00 | 0.00E+00 | 2213333.3 | #DIV/0! | Q6Q759.1 | RecName: Full=Sperm-associated antigen 17: AltName: Full=Projection protein PF6 homolog |
| 326 | 1 | 1 | 5.32 | 6.7 | 0.00E+00 | 0.00E+00 | 0.00E+00 | 0 | 0.00E+00 | 0.00E+00 | 9.63E+05 | 321000 | #DIV/0! | P0DI62.1 | RecName: Full=Casparian strip membrane protein 1 |
| 327 | 1 | 1 | 5.32 | 1.1 | 0.00E+00 | 0.00E+00 | 0.00E+00 | 0 | 6.50E+05 | 0.00E+00 | 0.00E+00 | 216666.67 | #DIV/0! | Q9USI6.1 | RecName: Full=Myosin type-2 heavy chain 1: AltName: Full=Myosin type II heavy chain 1 |
| 328 | 1 | 1 | 5.31 | 3.4 | 0.00E+00 | 0.00E+00 | 0.00E+00 | 0 | 4.60E+05 | 0.00E+00 | 0.00E+00 | 153333.33 | #DIV/0! | Q2NNU1.1 | RecName: Full=E3 ubiquitin-protein ligase IE2: AltName: Full=Immediate-early protein IE2 |
| 329 | 1 | 1 | 5.31 | 0 | 1.10E+06 | 0.00E+00 | 0.00E+00 | 366666.67 | 0.00E+00 | 0.00E+00 | 0.00E+00 | 0 | 0.00 | Q91ZU6.1 | RecName: Full=Dystonin: AltName: Full=Bullous pemphigoid antigen 1: Short=BPA: AltName: Full=Dystonia musculorum protein: AltName: Full=Hemidesmosomal plaque protein: AltName: Full=Microtubule actin cross-linking factor 2 |
| 330 | 1 | 1 | 5.31 | 3.5 | 0.00E+00 | 0.00E+00 | 0.00E+00 | 0 | 5.02E+06 | 0.00E+00 | 0.00E+00 | 1673333.3 | #DIV/0! | Q92783.3 | RecName: Full=Signal transducing adapter molecule 1: Short=STAM-1 |
| 331 | 1 | 1 | 5.3 | 7.9 | 0.00E+00 | 0.00E+00 | 0.00E+00 | 0 | 0.00E+00 | 0.00E+00 | 4.62E+07 | 15400000 | #DIV/0! | P70902.1 | RecName: Full=Variable large protein 15/16: Flags: Precursor |
| 332 | 1 | 1 | 5.29 | 8.9 | 0.00E+00 | 0.00E+00 | 0.00E+00 | 0 | 0.00E+00 | 3.92E+04 | 0.00E+00 | 13066.667 | #DIV/0! | A6Q599.1 | RecName: Full=UPF0102 protein NIS_1551 |
| 333 | 1 | 1 | 5.29 | 5.7 | 0.00E+00 | 0.00E+00 | 0.00E+00 | 0 | 1.14E+05 | 0.00E+00 | 0.00E+00 | 38000 | #DIV/0! | Q9BV10.1 | RecName: Full=Dol-P-Man:Man(7)GlcNAc(2)-PP-Dol alpha-1,6-mannosyltransferase: AltName: Full=Asparagine-linked glycosylation protein 12 homolog: Short=hALG12: AltName: Full=Dolichyl-P-Man:Man(7)GlcNAc(2)-PP-dolichyl-alpha-1,6-mannosyltransferase: AltName: Full=Mannosyltransferase ALG12 homolog: AltName: Full=Membrane protein SB87 |
| 334 | 2 | 1 | 5.28 | 0 | 4.26E+05 | 0.00E+00 | 0.00E+00 | 142000 | 4.36E+05 | 0.00E+00 | 0.00E+00 | 145333.33 | 1.02 | A2ASS6.1 | RecName: Full=Titin: AltName: Full=Connectin |
| 335 | 1 | 1 | 5.28 | 8.2 | 0.00E+00 | 0.00E+00 | 0.00E+00 | 0 | 0.00E+00 | 3.16E+05 | 0.00E+00 | 105333.33 | #DIV/0! | P46439.3 | RecName: Full=Glutathione S-transferase Mu 5: AltName: Full=GST class-mu 5: AltName: Full=GSTM5-5 |
| 336 | 2 | 1 | 5.28 | 0.5 | 0.00E+00 | 1.06E+06 | 0.00E+00 | 353333.33 | 0.00E+00 | 2.65E+06 | 0.00E+00 | 883333.33 | 2.50 | Q86UR5.1 | RecName: Full=Regulating synaptic membrane exocytosis protein 1: AltName: Full=Rab-3-interacting molecule 1: Short=RIM 1: AltName: Full=Rab-3-interacting protein 2 |
| 337 | 1 | 1 | 5.27 | 4.9 | 0.00E+00 | 0.00E+00 | 5.97E+05 | 199000 | 0.00E+00 | 0.00E+00 | 0.00E+00 | 0 | 0.00 | Q09544.1 | RecName: Full=ATP synthase subunit delta, mitochondrial: AltName: Full=F-ATPase delta subunit: Flags: Precursor |
| 338 | 1 | 1 | 5.26 | 5.6 | 0.00E+00 | 0.00E+00 | 0.00E+00 | 0 | 5.11E+05 | 0.00E+00 | 0.00E+00 | 170333.33 | #DIV/0! | B7L043.1 | RecName: Full=3-deoxy-manno-octulosonate cytidylyltransferase: AltName: Full=CMP-2-keto-3-deoxyoctulosonic acid synthase: Short=CKS: Short=CMP-KDO synthase |
| 339 | 1 | 1 | 5.25 | 13.5 | 0.00E+00 | 5.40E+05 | 0.00E+00 | 180000 | 0.00E+00 | 0.00E+00 | 0.00E+00 | 0 | 0.00 | Q7V6D4.1 | RecName: Full=Aldehyde decarbonylase: Short=AD: AltName: Full=Fatty aldehyde decarbonylase |
| 340 | 1 | 1 | 5.24 | 3.8 | 0.00E+00 | 0.00E+00 | 2.79E+05 | 93000 | 0.00E+00 | 0.00E+00 | 0.00E+00 | 0 | 0.00 | Q96R27.2 | RecName: Full=Olfactory receptor 2M4: AltName: Full=HTPCRX18: AltName: Full=OST710: AltName: Full=Olfactory receptor OR1-55: AltName: Full=Olfactory receptor TPCR100 |
| 341 | 1 | 1 | 5.22 | 2.8 | 0.00E+00 | 0.00E+00 | 1.10E+06 | 366666.67 | 0.00E+00 | 0.00E+00 | 0.00E+00 | 0 | 0.00 | P13682.4 | RecName: Full=Zinc finger protein 35: AltName: Full=Zinc finger protein HF.10 |
| 342 | 1 | 1 | 5.2 | 1.2 | 0.00E+00 | 0.00E+00 | 0.00E+00 | 0 | 0.00E+00 | 2.37E+06 | 0.00E+00 | 790000 | #DIV/0! | P51160.2 | RecName: Full=Cone cGMP-specific 3',5'-cyclic phosphodiesterase subunit alpha': AltName: Full=cGMP phosphodiesterase 6C: Flags: Precursor |
| 343 | 1 | 1 | 5.2 | 4.2 | 0.00E+00 | 1.41E+06 | 0.00E+00 | 470000 | 0.00E+00 | 0.00E+00 | 0.00E+00 | 0 | 0.00 | Q96SK2.2 | RecName: Full=Transmembrane protein 209 |
| 344 | 1 | 1 | 5.19 | 7.4 | 0.00E+00 | 0.00E+00 | 0.00E+00 | 0 | 6.33E+05 | 0.00E+00 | 0.00E+00 | 211000 | #DIV/0! | C5A4B7.1 | RecName: Full=S-adenosylmethionine synthase: Short=AdoMet synthase: AltName: Full=Methionine adenosyltransferase |
| 345 | 1 | 1 | 5.17 | 5.8 | 0.00E+00 | 0.00E+00 | 3.76E+05 | 125333.33 | 0.00E+00 | 0.00E+00 | 0.00E+00 | 0 | 0.00 | P46718.2 | RecName: Full=Programmed cell death protein 2: AltName: Full=Zinc finger protein Rp-8 |
| 346 | 1 | 1 | 5.17 | 2 | 0.00E+00 | 9.07E+05 | 0.00E+00 | 302333.33 | 0.00E+00 | 0.00E+00 | 0.00E+00 | 0 | 0.00 | Q9US47.2 | RecName: Full=Putative succinate-semialdehyde dehydrogenase C1002.12c [NADP(+)]: Short=SSDH |
| 347 | 1 | 1 | 5.15 | 7 | 0.00E+00 | 0.00E+00 | 0.00E+00 | 0 | 3.85E+06 | 0.00E+00 | 0.00E+00 | 1283333.3 | #DIV/0! | Q5B131.1 | RecName: Full=Probable carboxypeptidase AN5749: AltName: Full=Peptidase M20 domain-containing protein AN5749: Flags: Precursor |
| 348 | 1 | 1 | 5.15 | 6 | 0.00E+00 | 0.00E+00 | 4.72E+05 | 157333.33 | 0.00E+00 | 0.00E+00 | 0.00E+00 | 0 | 0.00 | Q9BXC0.1 | RecName: Full=Hydroxycarboxylic acid receptor 1: AltName: Full=G-protein coupled receptor 104: AltName: Full=G-protein coupled receptor 81 |
| 349 | 1 | 1 | 5.14 | 0.3 | 0.00E+00 | 0.00E+00 | 0.00E+00 | 0 | 0.00E+00 | 2.39E+06 | 0.00E+00 | 796666.67 | #DIV/0! | P08F94.1 | RecName: Full=Fibrocystin: AltName: Full=Polycystic kidney and hepatic disease 1 protein: AltName: Full=Polyductin: AltName: Full=Tigmin: Flags: Precursor |
| 350 | 1 | 1 | 5.14 | 11.4 | 2.35E+05 | 0.00E+00 | 0.00E+00 | 78333.333 | 0.00E+00 | 0.00E+00 | 0.00E+00 | 0 | 0.00 | Q9NX76.1 | RecName: Full=CKLF-like MARVEL transmembrane domain-containing protein 6: AltName: Full=Chemokine-like factor superfamily member 6 |
| 351 | 1 | 1 | 5.13 | 6.8 | 0.00E+00 | 0.00E+00 | 0.00E+00 | 0 | 6.49E+06 | 0.00E+00 | 0.00E+00 | 2163333.3 | #DIV/0! | Q58144.1 | RecName: Full=Putative rubrerythrin |
| 352 | 1 | 1 | 5.12 | 2.5 | 0.00E+00 | 0.00E+00 | 0.00E+00 | 0 | 6.85E+05 | 0.00E+00 | 0.00E+00 | 228333.33 | #DIV/0! | Q0W486.1 | RecName: Full=O-phospho-L-seryl-tRNA:Cys-tRNA synthase 2: AltName: Full=Sep-tRNA:Cys-tRNA synthase 2: Short=SepCysS 2 |
| 353 | 1 | 1 | 5.12 | 2.8 | 0.00E+00 | 0.00E+00 | 0.00E+00 | 0 | 4.95E+05 | 0.00E+00 | 0.00E+00 | 165000 | #DIV/0! | Q6GQV1.1 | RecName: Full=Protein FAM196B |
| 354 | 1 | 1 | 5.12 | 4.4 | 0.00E+00 | 0.00E+00 | 0.00E+00 | 0 | 1.15E+05 | 0.00E+00 | 0.00E+00 | 38333.333 | #DIV/0! | Q8TDR2.2 | RecName: Full=Serine/threonine-protein kinase 35: AltName: Full=CLP-36-interacting kinase 1: Short=CLIK-1: AltName: Full=PDLIM1-interacting kinase 1: AltName: Full=Serine/threonine-protein kinase 35 L1 |
| 355 | 1 | 1 | 5.09 | 1.3 | 0.00E+00 | 0.00E+00 | 0.00E+00 | 0 | 0.00E+00 | 2.79E+06 | 0.00E+00 | 930000 | #DIV/0! | Q14031.3 | RecName: Full=Collagen alpha-6(IV) chain: Flags: Precursor |
| 356 | 1 | 1 | 5.07 | 7.3 | 0.00E+00 | 0.00E+00 | 0.00E+00 | 0 | 9.34E+04 | 0.00E+00 | 0.00E+00 | 31133.333 | #DIV/0! | C6DF34.1 | RecName: Full=Ribose-5-phosphate isomerase A: AltName: Full=Phosphoriboisomerase A: Short=PRI |
| 357 | 1 | 1 | 5.06 | 2.5 | 0.00E+00 | 0.00E+00 | 0.00E+00 | 0 | 2.08E+06 | 0.00E+00 | 0.00E+00 | 693333.33 | #DIV/0! | Q9NSB2.2 | RecName: Full=Keratin, type II cuticular Hb4: AltName: Full=Keratin-84: Short=K84: AltName: Full=Type II hair keratin Hb4: AltName: Full=Type-II keratin Kb24 |
| 358 | 1 | 1 | 5.05 | 9.5 | 0.00E+00 | 0.00E+00 | 2.61E+05 | 87000 | 0.00E+00 | 0.00E+00 | 0.00E+00 | 0 | 0.00 | E9DBV9.1 | RecName: Full=Leucine aminopeptidase 1: AltName: Full=Leucyl aminopeptidase 1: Short=LAP1: Flags: Precursor |
| 359 | 1 | 1 | 5.05 | 9.3 | 2.13E+05 | 0.00E+00 | 0.00E+00 | 71000 | 0.00E+00 | 0.00E+00 | 0.00E+00 | 0 | 0.00 | Q48445.1 | RecName: Full=UPF0053 protein in cps region: AltName: Full=ORF19 |
| 360 | 1 | 1 | 5.05 | 0.6 | 0.00E+00 | 0.00E+00 | 0.00E+00 | 0 | 0.00E+00 | 6.65E+05 | 0.00E+00 | 221666.67 | #DIV/0! | Q8NEZ4.3 | RecName: Full=Histone-lysine N-methyltransferase MLL3: AltName: Full=Homologous to ALR protein: AltName: Full=Lysine N-methyltransferase 2C: Short=KMT2C: AltName: Full=Myeloid/lymphoid or mixed-lineage leukemia protein 3 |
| 361 | 1 | 1 | 5.04 | 2.6 | 0.00E+00 | 0.00E+00 | 0.00E+00 | 0 | 0.00E+00 | 1.26E+06 | 0.00E+00 | 420000 | #DIV/0! | O14730.2 | RecName: Full=Serine/threonine-protein kinase RIO3: AltName: Full=RIO kinase 3: AltName: Full=sudD homolog |
| 362 | 1 | 1 | 5.02 | 4.8 | 0.00E+00 | 0.00E+00 | 0.00E+00 | 0 | 2.07E+05 | 0.00E+00 | 0.00E+00 | 69000 | #DIV/0! | A4G2T7.1 | RecName: Full=tRNA (guanine-N(1)-)-methyltransferase: AltName: Full=M1G-methyltransferase: AltName: Full=tRNA [GM37] methyltransferase |
| 363 | 1 | 1 | 5.02 | 9.6 | 6.96E+05 | 0.00E+00 | 0.00E+00 | 232000 | 0.00E+00 | 0.00E+00 | 0.00E+00 | 0 | 0.00 | C5BHP3.1 | RecName: Full=DNA gyrase inhibitor |

**Table S8.** Details of the pathways associated with the differentially expressed proteins identified in meningiomas defined by DAVID (A) and PANTHER (B) analysis

1. **Summary of DAVID Analysis**

| **BIOCARTA** | | | | | | | | | | | | |
| --- | --- | --- | --- | --- | --- | --- | --- | --- | --- | --- | --- | --- |
| **Category** | **Term** | **Count** | **%** | ***p-*Value** | **Genes** | **List Total** | **Pop Hits** | **Pop Total** | **Fold Enrichment** | **Bonferroni** | **Benjamini** | **FDR** |
| **BIOCARTA** | h_intrinsicPathway:Intrinsic Prothrombin Activation Pathway | 11 | 7.746479 | 7.83E-14 | P02671, P00742, P01042, P00740, P05155, P04070, P01008, P03952, P00748, P00734, P07225 | 32 | 17 | 1437 | 29.05698529 | 2.19E-12 | 2.19E-12 | 6.55E-11 |
| **BIOCARTA** | h_alternativePathway:Alternative Complement Pathway | 9 | 6.338028 | 7.80E-13 | P01031, P07357, P00751, P02748, P13671, P01024, P10643, P27918, P06681 | 32 | 10 | 1437 | 40.415625 | 2.18E-11 | 1.09E-11 | 6.52E-10 |
| **BIOCARTA** | h_compPathway:Complement Pathway | 10 | 7.042254 | 6.24E-12 | P01031, P07357, P00751, P02748, P09871, P13671, P01024, P10643, P0C0L5, P06681 | 32 | 17 | 1437 | 26.41544118 | 1.75E-10 | 5.82E-11 | 5.22E-09 |
| **BIOCARTA** | h_classicPathway:Classical Complement Pathway | 9 | 6.338028 | 8.34E-12 | P01031, P07357, P02748, P09871, P13671, P01024, P10643, P0C0L5, P06681 | 32 | 12 | 1437 | 33.6796875 | 2.33E-10 | 5.83E-11 | 6.97E-09 |
| **BIOCARTA** | h_lectinPathway:Lectin Induced Complement Pathway | 8 | 5.633803 | 7.82E-10 | P01031, P07357, P02748, P13671, P01024, P10643, P0C0L5, P06681 | 32 | 12 | 1437 | 29.9375 | 2.19E-08 | 4.38E-09 | 6.54E-07 |
| **BIOCARTA** | h_amiPathway:Acute Myocardial Infarction | 7 | 4.929577 | 9.39E-08 | P02671, P00742, P04070, P01008, P00747, P00734, P07225 | 32 | 13 | 1437 | 24.18028846 | 2.63E-06 | 4.38E-07 | 7.86E-05 |
| **BIOCARTA** | h_extrinsicPathway:Extrinsic Prothrombin Activation Pathway | 6 | 4.225352 | 1.41E-06 | P02671, P00742, P04070, P01008, P00734, P07225 | 32 | 11 | 1437 | 24.49431818 | 3.96E-05 | 5.65E-06 | 0.0011818 |
| **BIOCARTA** | h_LairPathway:Cells and Molecules involved in local acute inflammatory response | 4 | 2.816901 | 0.004212541 | P01031, P13671, P01024, P10643 | 32 | 16 | 1437 | 11.2265625 | 0.111481934 | 0.014666422 | 3.4698726 |
| **BIOCARTA** | h_plateletAppPathway:Platelet Amyloid Precursor Protein Pathway | 3 | 2.112676 | 0.014760043 | P00740, P00747, P00734 | 32 | 9 | 1437 | 14.96875 | 0.340558211 | 0.045208605 | 11.697122 |
| **BIOCARTA** | h_fibrinolysisPathway:Fibrinolysis Pathway | 3 | 2.112676 | 0.021952 | P02671, P00747, P00734 | 32 | 11 | 1437 | 12.24715909 | 0.462863402 | 0.060258352 | 16.94674 |
| **KEGG_PATHWAY** | | | | | | | | | | | | |
| **Category** | **Term** | **Count** | **%** | ***p-*Value** | **Genes** | **List Total** | **Pop Hits** | **Pop Total** | **Fold Enrichment** | **Bonferroni** | **Benjamini** | **FDR** |
| **KEGG_PATHWAY** | hsa04610:Complement and coagulation cascades | 36 | 25.35211 | 1.45E-55 | P01031, P07357, P07358, P04003, P05546, P04070, P08603, P09871, P13671, P10643, P00751, P01008, P01009, P08697, P01024, P05160, P00734, P01023, P07225, P06681, P02671, P01042, P02748, P07360, P20851, P00742, P05154, P05155, P00740, P05156, P04275, P03952, P00748, P00747, P0C0L5, Q96IY4 | 57 | 69 | 5085 | 46.54462243 | 4.64E-54 | 4.64E-54 | 1.26E-52 |
| **KEGG_PATHWAY** | hsa05020:Prion diseases | 7 | 4.929577 | 1.72E-06 | P01031, P07357, P07358, P02748, P07360, P13671, P10643 | 57 | 35 | 5085 | 17.84210526 | 5.51E-05 | 1.84E-05 | 0.0014912 |
| **KEGG_PATHWAY** | hsa04512:ECM-receptor interaction | 4 | 2.816901 | 0.01550365 | P02751, P04275, P04004, P07996 | 57 | 84 | 5085 | 4.248120301 | 0.881601249 | 0.413407129 | 43.869258 |
| **BBID** | | | | | | | | | | | | |
| **Category** | **Term** | **Count** | **%** | ***p*-Value** | **Genes** | **List Total** | **Pop Hits** | **Pop Total** | **Fold Enrichment** | **Bonferroni** | **Benjamini** | **FDR** |
| **BBID** | 79.B_cell_Activation | 2 | 1.408451 | 0.07374001 | P01871, P01857, P01854 | 4 | 9 | 358 | 19.88888889 | 0.498124327 | 0.498124327 | 35.650122 |
| **PANTHER_PATHWAY** | | | | | | | | | | | | |
| **Category** | **Term** | **Count** | **%** | ***p*-Value** | **Genes** | **List Total** | **Pop Hits** | **Pop Total** | **Fold Enrichment** | **Bonferroni** | **Benjamini** | **FDR** |
| **PANTHER_PATHWAY** | P00011:Blood coagulation | 19 | 13.38028 | 6.96E-26 | P02671, P01042, P05546, P04070, P08519, P00742, P36955, P00740, P01008, P01009, P04275, P08697, P03952, P00748, P05160, P01023, P00747, P00734, P07225 | 29 | 55 | 2857 | 34.03322884 | 1.04E-24 | 1.04E-24 | 4.84E-23 |
| **PANTHER_PATHWAY** | P00050:Plasminogen activating cascade | 6 | 4.225352 | 1.46E-06 | P02671, P36955, P08697, P00747, P08519, Q96IY4 | 29 | 22 | 2857 | 26.86833856 | 2.19E-05 | 1.09E-05 | 0.001015 |
| **REACTOME_PATHWAY** | | | | | | | | | | | | |
| **Category** | **Term** | **Count** | **%** | **PValue** | **Genes** | **List Total** | **Pop Hits** | **Pop Total** | **Fold Enrichment** | **Bonferroni** | **Benjamini** | **FDR** |
| **REACTOME_PATHWAY** | REACT_604:Hemostasis | 27 | 19.01408 | 3.08E-17 | P04196, P02751, P04114, P04070, P02775, P01008, P09486, P01009, P08697, P05160, P07996, P00734, P01023, P07225, P02671, P02787, P01042, P02768, P00742, P05155, P00740, P10909, P04275, P03952, P00748, P00747, P02647 | 54 | 235 | 3398 | 7.229787234 | 6.17E-16 | 6.17E-16 | 2.35E-14 |
| **REACTOME_PATHWAY** | REACT_6900:Signaling in Immune system | 17 | 11.97183 | 3.54E-06 | P02751, P01031, P07357, P07358, P04114, P02748, P04070, P07360, P09871, P13671, P10643, P01834, P00751, P01024, P00734, P06681, P07225 | 54 | 286 | 3398 | 3.74035224 | 7.09E-05 | 3.54E-05 | 0.0026962 |
| **REACTOME_PATHWAY** | REACT_602:Metabolism of lipids and lipoproteins | 8 | 5.633803 | 0.007928057 | P04114, P02649, P02768, P02655, P06727, P02656, P02652, P02647 | 54 | 150 | 3398 | 3.356049383 | 0.147168269 | 0.051681004 | 5.8781772 |
| **REACTOME_PATHWAY** | REACT_13552:Integrin cell surface interactions | 5 | 3.521127 | 0.036270994 | P02671, P02751, P04275, P04004, P07996 | 54 | 81 | 3398 | 3.884316415 | 0.522362292 | 0.168667706 | 24.511242 |

1. **Summary of PANTHER Analysis**

| **Pathways** | **List Total** | **Count** | **Expected** | **+/-** | ***p*-value** |
| --- | --- | --- | --- | --- | --- |
| Unclassified | 17174 | 99 | 109.05 | - | 1.00E+00 |
| Blood coagulation | 57 | 17 | 0.36 | + | 3.79E-21 |
| Plasminogen activating cascade | 18 | 5 | 0.11 | + | 2.43E-05 |
| Angiotensin II-stimulated signaling through G proteins and beta-arrestin | 38 | 1 | 0.24 | + | 4.30E-02 |
| FAS signaling pathway | 38 | 1 | 0.24 | + | 4.50E-02 |
| Integrin signalling pathway | 209 | 1 | 1.33 | - | 3.90E-02 |
| p53 pathway | 109 | 1 | 0.69 | + | 1.00E+00 |
| Transcription regulation by bZIP transcription factor | 58 | 1 | 0.37 | + | 1.00E+00 |
| Nicotinic acetylcholine receptor signaling pathway | 105 | 1 | 0.67 | + | 1.00E+00 |
| Muscarinic acetylcholine receptor 2 and 4 signaling pathway | 63 | 1 | 0.4 | + | 1.00E+00 |
| Muscarinic acetylcholine receptor 1 and 3 signaling pathway | 60 | 1 | 0.38 | + | 1.00E+00 |
| Arginine biosynthesis | 9 | 1 | 0.06 | + | 1.00E+00 |
| Gonadotropin releasing hormone receptor pathway | 282 | 1 | 1.79 | - | 1.00E+00 |
| Inflammation mediated by chemokine and cytokine signaling pathway | 289 | 1 | 1.84 | - | 1.00E+00 |
| Huntington disease | 164 | 1 | 1.04 | - | 1.00E+00 |
| General transcription regulation | 41 | 1 | 0.26 | + | 1.00E+00 |
|  |  |  |  |  |  |
| **Biological Process** | **List Total** | **Count** | **Expected** | **+/-** | ***p*- value** |
| Proteolysis | 1131 | 53 | 7.18 | + | 4.04E-30 |
| Complement activation | 99 | 22 | 0.63 | + | 4.92E-25 |
| Blood coagulation | 271 | 28 | 1.72 | + | 2.43E-23 |
| Response to external stimulus | 271 | 28 | 1.72 | + | 2.43E-23 |
| Immune system process | 2480 | 53 | 15.75 | + | 2.24E-14 |
| Unclassified | 6816 | 6 | 43.28 | - | 1.93E-13 |
| Immune response | 725 | 28 | 4.6 | + | 2.46E-12 |
| Response to stimulus | 1767 | 42 | 11.22 | + | 3.40E-12 |
| Protein metabolic process | 3178 | 55 | 20.18 | + | 3.90E-11 |
| Cell-cell adhesion | 724 | 24 | 4.6 | + | 5.69E-09 |
| Cell adhesion | 1301 | 28 | 8.26 | + | 1.79E-06 |
| Nucleobase, nucleoside, nucleotide and nucleic acid metabolic process | 3782 | 5 | 24.02 | - | 9.89E-05 |
| Metabolic process | 8127 | 77 | 51.61 | + | 7.45E-04 |
| Lipid transport | 220 | 9 | 1.4 | + | 2.23E-03 |
| Primary metabolic process | 7813 | 73 | 49.61 | + | 3.52E-03 |
| Transcription | 2224 | 2 | 14.12 | - | 7.65E-03 |
| Transcription from RNA polymerase II promoter | 2214 | 2 | 14.06 | - | 8.14E-03 |
|  |  |  |  |  |  |
| **Molecular Function** | **List Total** | **Count** | **Expected** | **+/-** | ***p*- value** |
| Lipid transporter activity | 102 | 24 | 0.65 | + | 4.23E-28 |
| Serine-type peptidase activity | 350 | 30 | 2.22 | + | 6.57E-23 |
| Serine-type endopeptidase inhibitor activity | 125 | 21 | 0.79 | + | 2.10E-21 |
| Peptidase inhibitor activity | 196 | 22 | 1.24 | + | 8.82E-19 |
| Peptidase activity | 717 | 33 | 4.55 | + | 3.40E-17 |
| Enzyme inhibitor activity | 389 | 22 | 2.47 | + | 1.17E-12 |
| Metallopeptidase activity | 233 | 17 | 1.48 | + | 3.16E-11 |
| Hydrolase activity | 2223 | 40 | 14.12 | + | 8.57E-08 |
| Unclassified | 7724 | 18 | 49.05 | - | 1.87E-07 |
| Transporter activity | 1049 | 25 | 6.66 | + | 1.55E-06 |
| Protein binding | 3073 | 44 | 19.51 | + | 9.98E-06 |
| Nucleic acid binding | 3715 | 4 | 23.59 | - | 2.24E-05 |
| Enzyme regulator activity | 1216 | 23 | 7.72 | + | 3.66E-04 |
| Calmodulin binding | 279 | 10 | 1.77 | + | 1.97E-03 |
| DNA binding | 2309 | 2 | 14.66 | - | 3.93E-03 |
| Calcium-dependent phospholipid binding | 139 | 7 | 0.88 | + | 5.04E-03 |
| Transcription factor activity | 2041 | 2 | 12.96 | - | 2.05E-02 |
| Transcription regulator activity | 2041 | 2 | 12.96 | - | 2.05E-02 |
| Transferase activity | 1601 | 1 | 10.17 | - | 4.49E-02 |
|  |  |  |  |  |  |
| Cellular Component | **List Total** | **Count** | **Expected** | **+/-** | ***p*- value** |
| Extracellular region | 601 | 18 | 3.82 | + | 2.04E-06 |
| Intermediate filament cytoskeleton | 87 | 5 | 0.55 | + | 9.18E-03 |
| Extracellular matrix | 558 | 11 | 3.54 | + | 3.28E-02 |
| Unclassified | 17814 | 101 | 113.12 | - | 4.34E-02 |
|  |  |  |  |  |  |
| **PANTHER Protein Class** | **List Total** | **Count** | **Expected** | **+/-** | ***p*- value** |
| Apolipoprotein | 102 | 24 | 0.65 | + | 5.10E-28 |
| Serine protease | 350 | 30 | 2.22 | + | 7.94E-23 |
| Complement component | 90 | 20 | 0.57 | + | 1.33E-22 |
| Transfer/carrier protein | 475 | 32 | 3.02 | + | 2.30E-21 |
| Serine protease inhibitor | 125 | 21 | 0.79 | + | 2.54E-21 |
| Protease inhibitor | 196 | 22 | 1.24 | + | 1.07E-18 |
| Protease | 717 | 33 | 4.55 | + | 4.11E-17 |
| Defense/immunity protein | 694 | 30 | 4.41 | + | 1.26E-14 |
| Metalloprotease | 233 | 17 | 1.48 | + | 3.82E-11 |
| Hydrolase | 1852 | 40 | 11.76 | + | 3.95E-10 |
| Unclassified | 6898 | 11 | 43.8 | - | 1.96E-09 |
| Cell adhesion molecule | 668 | 19 | 4.24 | + | 9.40E-06 |
| Nucleic acid binding | 2724 | 3 | 17.3 | - | 2.31E-03 |
| Intracellular calcium-sensing protein | 279 | 10 | 1.77 | + | 2.38E-03 |
| Calmodulin | 279 | 10 | 1.77 | + | 2.38E-03 |
| Annexin | 139 | 7 | 0.88 | + | 6.09E-03 |
| Enzyme modulator | 1540 | 23 | 9.78 | + | 1.85E-02 |
| Transcription factor | 2041 | 2 | 12.96 | - | 2.47E-02 |
| Intermediate filament | 87 | 5 | 0.55 | + | 4.59E-02 |

**Table S9.** ELISA-based measurement of serum HPX, Apo E, Apo A1 and RBP in healthy controls and different grades of meningioma patients

**A. ELISA-based measurement of serum HPX**

| **HC** | **Conc.**  **(g/L)** | **HC** | **Conc.**  **(g/L)** | **MG I** | **Conc.**  **(g/L)** | **MG II** | **Conc. (g/L)** |
| --- | --- | --- | --- | --- | --- | --- | --- |
| HC 01 | 1.21 | HC 24 | 1.18 | MG I (1) | 1.71 | MG II (1) | 1.04 |
| HC 02 | 1.09 | HC 25 | 1.07 | MG I (2) | 1.03 | MG II (2) | 2.63 |
| HC 03 | 0.89 | HC 26 | 1.02 | MG I (3) | 1.16 | MG II (3) | 2.68 |
| HC 04 | 0.78 | HC 27 | 0.97 | MG I (4) | 0.59 | MG II (4) | 2.43 |
| HC 05 | 0.83 | HC 28 | 0.69 | MG I (5) | 2.19 | MG II (5) | 3.71 |
| HC 06 | 0.68 | HC 29 | 0.85 | MG I (6) | 0.56 | MG II (1) | 1.04 |
| HC 07 | 1.23 | HC 30 | 0.7 | MG I (7) | 0.91 |  |  |
| HC 08 | 1.07 | HC 31 | 0.49 | MG I (8) | 1.8 | **MG III*** | **Conc. (g/L)** |
| HC 09 | 1.06 | HC 32 | 0.93 | MG I (9) | 1.43 | MG III (1) | 3.7 |
| HC 10 | 1.31 | HC 33 | 1.11 | MG I (10) | 1.37 | MG III (2) | 3.65 |
| HC 11 | 0.82 | HC 34 | 1.23 | MG I (11) | 0.67 | MG III (3) | 3.82 |
| HC 12 | 0.67 | HC 35 | 1.05 | MG I (12) | 1.78 |  |  |
| HC 13 | 1.33 | HC 36 | 0.89 | MG I (13) | 1.87 |  |  |
| HC 14 | 1.32 | HC 37 | 1.07 | MG I (14) | 1.61 |  |  |
| HC 15 | 0.98 | HC 38 | 1.02 |  |  |  |  |
| HC 16 | 0.49 | HC 39 | 0.68 |  |  |  |  |
| HC 17 | 1.06 | HC 40 | 0.7 |  |  |  |  |
| HC 18 | 1.28 | HC 41 | 0.67 |  |  |  |  |
| HC 19 | 0.84 | HC 42 | 0.91 |  |  |  |  |
| HC 20 | 0.93 | HC 43 | 1.23 |  |  |  |  |
| HC 21 | 1.18 | HC 44 | 1.17 |  |  |  |  |
| HC 22 | 1.21 | HC 45 | 1.08 |  |  |  |  |
| HC 23 | 0.97 |  |  |  |  |  |  |

|  | **HC** | **MG I** | **MG II** | **MG III*** |
| --- | --- | --- | --- | --- |
| **Number of subjects** | 45 | 14 | 5 | 3 |
| **Minimum** | 0.49 | 0.56 | 1.04 | 3.65 |
| **25% Percentile** | 0.83 | 0.85 | 1.74 | 3.65 |
| **Median** | 1.02 | 1.4 | 2.63 | 3.7 |
| **75% Percentile** | 1.18 | 1.78 | 3.19 | 3.82 |

| **Maximum** | 1.33 | 2.19 | 3.71 | 3.82 |
| --- | --- | --- | --- | --- |
| **Mean** | 0.97 | 1.33 | 2.49 | 3.72 |
| **Std. Deviation** | 0.22 | 0.52 | 0.95 | 0.09 |
| **Std. Error of Mean** | 0.03 | 0.14 | 0.43 | 0.05 |

**B. ELISA-based measurement of serum Apo E**

| **HC** | **Conc.**  **(mg/L)** | **HC** | **Conc.**  **(mg/L)** | **MG I** | **Conc.**  **(mg/L)** | **MG II** | **Conc. (mg/L)** |
| --- | --- | --- | --- | --- | --- | --- | --- |
| HC 01 | 42.78 | HC 24 | 49.48 | MG I (1) | 120.56 | MG II (1) | 106.23 |
| HC 02 | 56.09 | HC 25 | 50.9 | MG I (2) | 109.45 | MG II (2) | 176.05 |
| HC 03 | 146.1 | HC 26 | 59.4 | MG I (3) | 98.58 | MG II (3) | 61.87 |
| HC 04 | 65.09 | HC 27 | 50.62 | MG I (4) | 167.89 | MG II (4) | 92.99 |
| HC 05 | 151.24 | HC 28 | 60.62 | MG I (5) | 122.74 | MG II (5) | 183.77 |
| HC 06 | 49.87 | HC 29 | 44.89 | MG I (6) | 112.03 | MG II (1) | 106.23 |
| HC 07 | 157.67 | HC 30 | 47.98 | MG I (7) | 72.61 |  |  |
| HC 08 | 102.6 | HC 31 | 102.9 | MG I (8) | 83.26 | **MG III*** | **Conc. (mg/L)** |
| HC 09 | 43.89 | HC 32 | 101.78 | MG I (9) | 184.35 | MG III (1) | 109.67 |
| HC 10 | 149.08 | HC 33 | 98.09 | MG I (10) | 138.76 | MG III (2) | 112.78 |
| HC 11 | 60.45 | HC 34 | 45.02 | MG I (11) | 148.03 | MG III (3) | 119.67 |
| HC 12 | 147.09 | HC 35 | 126.9 | MG I (12) | 157.3 |  |  |
| HC 13 | 42.89 | HC 36 | 62.8 | MG I (13) | 43.29 |  |  |
| HC 14 | 40.87 | HC 37 | 50.75 | MG I (14) | 155.04 |  |  |
| HC 15 | 60.46 | HC 38 | 49.3 |  |  |  |  |
| HC 16 | 77.89 | HC 39 | 52.87 |  |  |  |  |
| HC 17 | 89.76 | HC 40 | 70.64 |  |  |  |  |
| HC 18 | 49.8 | HC 41 | 80.67 |  |  |  |  |
| HC 19 | 56.03 | HC 42 | 180.91 |  |  |  |  |
| HC 20 | 48.1 | HC 43 | 121.23 |  |  |  |  |
| HC 21 | 53.95 | HC 44 | 51.17 |  |  |  |  |
| HC 22 | 48.03 | HC 45 | 101.08 |  |  |  |  |
| HC 23 | 65.23 |  |  |  |  |  |  |

|  | **HC** | **MG I** | **MG II** | **MG III*** |
| --- | --- | --- | --- | --- |
| **Number of subjects** | 45 | 14 | 5 | 3 |
| **Minimum** | 40.87 | 43.29 | 61.87 | 109.7 |
| **25% Percentile** | 49.64 | 94.75 | 77.43 | 109.7 |

| **Median** | 60.45 | 121.7 | 106.2 | 112.8 |
| --- | --- | --- | --- | --- |
| **75% Percentile** | 101.4 | 155.6 | 179.9 | 119.7 |
| **Maximum** | 180.9 | 184.4 | 183.8 | 119.7 |
| **Mean** | 77 | 122.4 | 124.2 | 114 |
| **Std. Deviation** | 38.13 | 39.45 | 53.43 | 5.118 |
| **Std. Error of Mean** | 5.684 | 10.54 | 23.89 | 2.955 |

**C. ELISA-based measurement of serum Apo AI**

| **HC** | **Conc. (g/L)** | **HC** | **Conc. (g/L)** | **MG I** | **Conc. (g/L)** | **MG II** | **Conc. (g/L)** |
| --- | --- | --- | --- | --- | --- | --- | --- |
| HC 01 | 1.5 | HC 24 | 0.56 | MG I (1) | 3.19 | MG II (1) | 8.04 |
| HC 02 | 1.32 | HC 25 | 1.26 | MG I (2) | 2.53 | MG II (2) | 2.63 |
| HC 03 | 1.11 | HC 26 | 1.45 | MG I (3) | 3.82 | MG II (3) | 7.68 |
| HC 04 | 0.98 | HC 27 | 1.34 | MG I (4) | 0.92 | MG II (4) | 4.43 |
| HC 05 | 1.54 | HC 28 | 1.29 | MG I (5) | 0.95 | MG II (5) | 6.71 |
| HC 06 | 1.26 | HC 29 | 1.41 | MG I (6) | 1.52 |  |  |
| HC 07 | 0.94 | HC 30 | 1.17 | MG I (7) | 0.81 | **MG III*** | **Conc. (g/L)** |
| HC 08 | 1.37 | HC 31 | 1.21 | MG I (8) | 0.58 | MG III (1) | 15.67 |
| HC 09 | 1.27 | HC 32 | 1.39 | MG I (9) | 2.12 | MG III (2) | 14.98 |
| HC 10 | 1.18 | HC 33 | 1.22 | MG I (10) | 2.31 | MG III (3) | 15.78 |
| HC 11 | 0.78 | HC 34 | 1.18 | MG I (11) | 0.48 |  |  |
| HC 12 | 1.17 | HC 35 | 1.27 | MG I (12) | 0.75 |  |  |
| HC 13 | 1.16 | HC 36 | 1.15 | MG I (13) | 2.21 |  |  |
| HC 14 | 1.03 | HC 37 | 0.73 | MG I (14) | 2.91 |  |  |
| HC 15 | 1.42 | HC 38 | 0.94 |  |  |  |  |
| HC 16 | 1.35 | HC 39 | 1.03 |  |  |  |  |
| HC 17 | 0.86 | HC 40 | 1.12 |  |  |  |  |
| HC 18 | 0.52 | HC 41 | 0.67 |  |  |  |  |
| HC 19 | 1.32 | HC 42 | 0.91 |  |  |  |  |
| HC 20 | 1.18 | HC 43 | 1.23 |  |  |  |  |
| HC 21 | 1.67 | HC 44 | 1.17 |  |  |  |  |
| HC 22 | 1.03 | HC 45 | 1.08 |  |  |  |  |
| HC 23 | 0.79 |  |  |  |  |  |  |

|  | **HC** | **MG I** | **MG II** | **MG III*** |
| --- | --- | --- | --- | --- |
| **Number of subjects** | 45 | 14 | 5 | 3 |
| **Minimum** | 0.52 | 0.48 | 2.63 | 14.98 |
| **25% Percentile** | 1.005 | 0.795 | 3.53 | 14.98 |
| **Median** | 1.18 | 1.82 | 6.71 | 15.67 |
| **75% Percentile** | 1.32 | 2.63 | 7.86 | 15.78 |
| **Maximum** | 1.67 | 3.82 | 8.04 | 15.78 |
| **Mean** | 1.15 | 1.79 | 5.89 | 15.48 |
| **Std. Deviation** | 0.25 | 1.08 | 2.30 | 0.43 |
| **Std. Error of Mean** | 0.038 | 0.29 | 1.03 | 0.25 |

**D. ELISA-based measurement of serum plasma RBP**

| **HC** | **Conc.**  **(mg/L)** | **HC** | **Conc.**  **(mg/L)** | **MG I** | **Conc.**  **(mg/L)** | **MG II** | **Conc. (mg/L)** |
| --- | --- | --- | --- | --- | --- | --- | --- |
| HC 01 | 24.05 | HC 24 | 39.78 | MG I (1) | 36.56 | MG II (1) | 56.23 |
| HC 02 | 29.8 | HC 25 | 50.02 | MG I (2) | 31.9 | MG II (2) | 36.05 |
| HC 03 | 45.98 | HC 26 | 47.43 | MG I (3) | 29.78 | MG II (3) | 61.87 |
| HC 04 | 52.56 | HC 27 | 36.56 | MG I (4) | 32.78 | MG II (4) | 62.99 |
| HC 05 | 33.98 | HC 28 | 31.9 | MG I (5) | 45.09 | MG II (5) | 53.77 |
| HC 06 | 21.9 | HC 29 | 29.78 | MG I (6) | 48.67 | MG II (1) | 56.23 |
| HC 07 | 29.89 | HC 30 | 32.78 | MG I (7) | 56.9 |  |  |
| HC 08 | 37.02 | HC 31 | 45.09 | MG I (8) | 65.09 | **MG III*** | **Conc. (mg/L)** |
| HC 09 | 67.89 | HC 32 | 48.67 | MG I (9) | 45.9 | MG III (1) | 43.56 |
| HC 10 | 55.43 | HC 33 | 56.9 | MG I (10) | 43.01 | MG III (2) | 35.89 |
| HC 11 | 27.56 | HC 34 | 65.09 | MG I (11) | 36.89 | MG III (3) | 41.67 |
| HC 12 | 23.98 | HC 35 | 45.9 | MG I (12) | 38.32 |  |  |
| HC 13 | 33.05 | HC 36 | 43.01 | MG I (13) | 49.45 |  |  |
| HC 14 | 31.78 | HC 37 | 36.89 | MG I (14) | 52.31 |  |  |
| HC 15 | 33.02 | HC 38 | 38.32 |  |  |  |  |
| HC 16 | 28.97 | HC 39 | 49.45 |  |  |  |  |
| HC 17 | 12.78 | HC 40 | 52.31 |  |  |  |  |
| HC 18 | 13.09 | HC 41 | 80.67 |  |  |  |  |
| HC 19 | 41.94 | HC 42 | 80.91 |  |  |  |  |
| HC 20 | 18.56 | HC 43 | 21.23 |  |  |  |  |
| HC 21 | 32.93 | HC 44 | 51.17 |  |  |  |  |

| HC 22 | 36.73 | HC 45 | 31.08 |  |  |  |  |
| --- | --- | --- | --- | --- | --- | --- | --- |
| HC 23 | 41.78 |  |  |  |  |  |  |

|  | **HC** | **MG I** | **MG II** | **MG III*** |
| --- | --- | --- | --- | --- |
| **Number of subjects** | 45 | 14 | 5 | 3 |
| **Minimum** | 12.78 | 29.78 | 36.05 | 35.89 |
| **25% Percentile** | 29.85 | 35.62 | 44.91 | 35.89 |
| **Median** | 36.89 | 44.05 | 56.23 | 41.67 |
| **75% Percentile** | 49.06 | 50.17 | 62.43 | 43.56 |
| **Maximum** | 80.91 | 65.09 | 62.99 | 43.56 |
| **Mean** | 39.77 | 43.76 | 54.18 | 40.37 |
| **Std. Deviation** | 15.31 | 10.19 | 10.84 | 4.00 |
| **Std. Error of Mean** | 2.28 | 2.72 | 4.85 | 2.31 |

* MG III sample was analyzed in three technical replicates

**Table S10.** Statistical summary of ROC curve analysis for evaluating performance of different serum proteins for prediction of grade I and grade II meningiomas

| **Classifier protein** | **HC vs. MG I** | | **HC vs. MG II** | | **MG I vs. MG II** | |
| --- | --- | --- | --- | --- | --- | --- |
|  | **AUC** | **95% CI** | **AUC** | **95% CI** | **AUC** | **95% CI** |
| **Apolipoprotein E (Apo E)** | 0.794 | 0.654 - 0.933 | 0.813 | 0.647-0.979 | 0.514 | 0.164-0.864 |
| **Hemopexin (HPX)** | 0.699 | 0.492 - 0.906 | 0.906 | 0.738 - 1.07 | 0.871 | 0.636-0.987 |
| **Apolipoprotein A-I (Apo A-I)** | 0.616 | 0.383 - 0.849 | 0.937 | 0.783 - 0.987 | 0.957 | 0.862-1.052 |
| **Plasma retinol-**  **binding protein**  **(RBP4)** | 0.608 | 0.457 - 0.759 | 0.804 | 0.624 - 0.985 | 0.757 | 0.479- 0.94 |

**Table S11.** Comparison of the fold changes of the differentially expressed proteins identified from iTRAQ data with proteins reported in published literature in tissue and CSF samples

| **Name of Protein** | **iTRAQ results obtained from Q-TOF data** | | | **Meningioma tissue proteomics data^17^** | | |
| --- | --- | --- | --- | --- | --- | --- |
|  | Benign | Atypical | Anaplastic | Benign | Atypical | Anaplastic |
| Vitamin D-binding protein | 0.85 | 1.2 | 0.78 | 0.735 | 0.374 | 0.628 |
| Apolipoprotein A-I | 2.28 | 7.09 | 13.26 | 3.951 | 1.87 | 1.937 |
| α-1-antitrypsin | 1.69 | 6.24 | 7.33 | 1.841 | 1.067 | 2.349 |

| **Name of Protein** | **iTRAQ results obtained from Q-TOF data** | | | **Meningioma CSF proteomics data^20^** |
| --- | --- | --- | --- | --- |
|  | Benign | Atypical | Anaplastic | Meningioma *vs.* Non brain tumor samples |
| Serum albumin precursor | 0.63 | 1.05 | 0.61 | 4.51 |
| Apolipoprotein E | 2.39 | 1.48 | 2.03 | 2.87 |
| α-1-antitrypsin | 1 | 6.24 | 7.33 | 2.73 |
| Transthyretin | 1 | 1.42 | 1.86 | -3.1 |
